# Investigating C_7_ modified tetrandrine derivatives for synthesis anti-hepatocellular carcinoma activity and mechanistic insights

**DOI:** 10.1038/s41598-025-18875-1

**Published:** 2025-09-29

**Authors:** Taibai Jiang, Lihong Shi, Xueke Peng, Shan Zheng, Qian Chen, Junjie Lan, Weidong Pan

**Affiliations:** 1 Guizhou Province Engineering Research Center of Medical Resourceful Healthcare Products, College of Pharmacy, Guiyang Healthcare Vocational University, Guiyang, 550081 People’s Republic of China; 2 School of Pharmaceutical Sciences, Guizhou University, Guiyang, 550025 People’s Republic of China; 3 Department of Pharmacy, Guizhou Provincial People’s Hospital, Guiyang, 550002 People’s Republic of China; 4 Eugenics Research Center, The First Affiliated Hospital of Guizhou University of Traditional Chinese Medicine, Guiyang, 550002 People’s Republic of China

**Keywords:** Tetrandrine, Sulfonate derivatives, HCC cells, Caspase-3-dependent apoptotic pathway, Candidate drug, Liver cancer, Drug screening

## Abstract

Hepatocellular carcinoma (HCC) remains a significant global health challenge, with drug therapy being a critical treatment approach. However, the development of drug resistance and the occurrence of adverse reactions to existing medications have made the creation of new HCC treatments urgent. Tetrandrine, a bisbenzylisoquinoline alkaloid from natural products, has shown great potential in this area. Yet, systematic comparative studies on different tetrandrine modification sites (such as C-7 and C-14) are lacking, which has hindered the simultaneous optimization of its antitumor activity and synthetic feasibility. In this study, we focused on a C-7 modification strategy and designed and synthesized 38 novel tetrandrine sulfonate derivatives (including 31 new compounds) through an efficient one-step reaction. The in vitro antiproliferative activities of these derivatives were evaluated against four HCC cell lines (HepG-2, SMMC-7721, SK-Hep-1, and QGY-7701), and a systematic comparison with previously reported C-14 sulfonate derivatives was conducted. The results showed that all the C-7 derivatives exhibited good antiproliferative activities against HCC. Notably, compound **15** demonstrated significant antiproliferative activity against all tested cell lines, with IC_50_ values ranging from 3.28 to 6.16 µM, which is superior to the first-line clinical drug 5-fluorouracil. Mechanistic studies revealed that compound **15** exerts its antitumor effects by regulating the balance of Bcl-2 and Bax proteins, triggering the release of cytochrome C from mitochondria, and subsequently activating the caspase-3-dependent apoptosis pathway. The results of target prediction showed that PDGFRA, FLT1, PIK3CD and MET might be the main targets of compound **15** against hepatocellular carcinoma. Although C-14 sulfonate derivatives showed slightly higher in vitro activity, C-7 derivatives (particularly compound **15**) exhibited significant advantages in synthetic efficiency (one-step reaction), ease of purification, environmental friendliness, and scalability for industrial production. In conclusion, this study has established C-7 sulfonation as an efficient strategy for developing tetrandrine-based anti-HCC agents. With its remarkable antitumor activity, excellent synthetic process characteristics, and clear apoptosis-inducing mechanism, compound **15** has emerged as a highly promising clinical candidate. This C-7 modification platform provides a viable path for transforming natural product derivatives into clinical therapies for HCC treatment.

**Supplementary Information:**

The online version contains supplementary material available at 10.1038/s41598-025-18875-1.

## Introduction

Liver cancer remains a widespread malignant tumor globally, with persistently high rates of occurrence and mortality^1,2^. Recent data on global cancer epidemiology show that hepatocellular carcinoma (HCC) ranks as the sixth most frequently diagnosed cancer and the third deadliest cancer worldwide^3,4^. Pharmacological intervention, as a principal therapeutic modality for HCC, is predominantly employed in clinical settings for patients deemed ineligible for surgical resection^5,6^. Although 5-fluorouracil (5-FU), sorafenib, lenvatinib, regorafenib, ramucirumab, and cabozantinib could decrease the incidence and mortality of liver cancer^7–9^, they cause fatigue, nausea, emesis, diarrhoea, cutaneous eruptions, hypertension induction, coagulopathies, and hepatic dysfunction^10–14^. Meanwhile, their long-term use resulted in drug resistance and reduction of effectiveness, which drastically limits their clinical application^15–17^. Therefore, the development of new chemical drugs against HCC for preventing liver cancer is imminent.

Natural products are one of the key drivers of new drug discovery due to their biophilicity and structural diversity^18–23^. Among them, the natural bisbenzylisoquinoline alkaloid tetrandrine has attracted much attention from medicinal chemists because of its unique structure and remarkable biological activity. Studies have shown that tetrandrine is highly promising in inhibiting the growth of tumour cells, which has inspired researchers to structurally modify it to develop anti-tumour drug candidates^24–27^. In the early stage, researchers introduced halogens at the C-5 position of tetrandrine, and then used Sonogashira or Suzuki-Miyaura reactions to access aryl or alkynyl groups, and the anti-hepatocarcinogenic activity of some of the derivatives was thus enhanced^28–30^. Meanwhile, some researchers have also obtained derivatives with better anticancer activity by introducing ester groups at the C-7 position^31^. Our group has adopted a new strategy to introduce an amino group at the C-14 position of tetrandrine and constructed a series of C-14 derivatives containing urea, thiourea, amide and other groups based on this, and some of these compounds showed good inhibitory effects on HCC cells^32–35^ .

Derivatisation studies of tetrandrine have demonstrated that modifications at C-5 (via C-C bonds), C-7 (via C-O bonds), and C-14 (via C-N bonds) all enhance inhibitory effects on HCC cells. However, existing studies have not been able to determine which derivatization is more favourable for improving the anti-HCC activity. Recently, the authors modified the C-14 amino tetrandrine by diazotization and Sandmeyer reaction to synthesize C14-OH tetrandrine for the first time, and further introduced a sulfonate ester moiety at the C-14 position by esterification reaction, and successfully prepared 40 tetrandrine sulfonate esterified derivatives, which were tested for their anti-HCC activities^36^. This result lays a foundation for exploring the HCC inhibitory effects of tetrandrine derivatives with the same moiety at different sites. Although tetrandrine C-7 sulfonate derivatives have been reported to have antitumor activities, only 8 derivatives are currently publicly available, with sulfonate substituents mainly being alkyl chains (3 types) and substituted benzene rings (5 types). Due to insufficient structural diversity, comprehensive structure-activity relationship studies are difficult to conduct systematically. Therefore, in the present study, we designed and synthesized an expanded series of C-7 sulfonate ester derivatives of tetrandrine. Systematic comparison of their anti-HCC activity with our previously reported C-14 analogues enabled direct evaluation of positional effects on pharmacological efficacy. Derivatives exhibiting superior activity underwent preliminary mechanistic studies to explore their anti - HCC action. This work aims to provide experimental data to support the design and synthesis of tetrandrine derivatives with improved anti-HCC activity.

## Results and discussion

### Chemistry

**Fig. 1:**
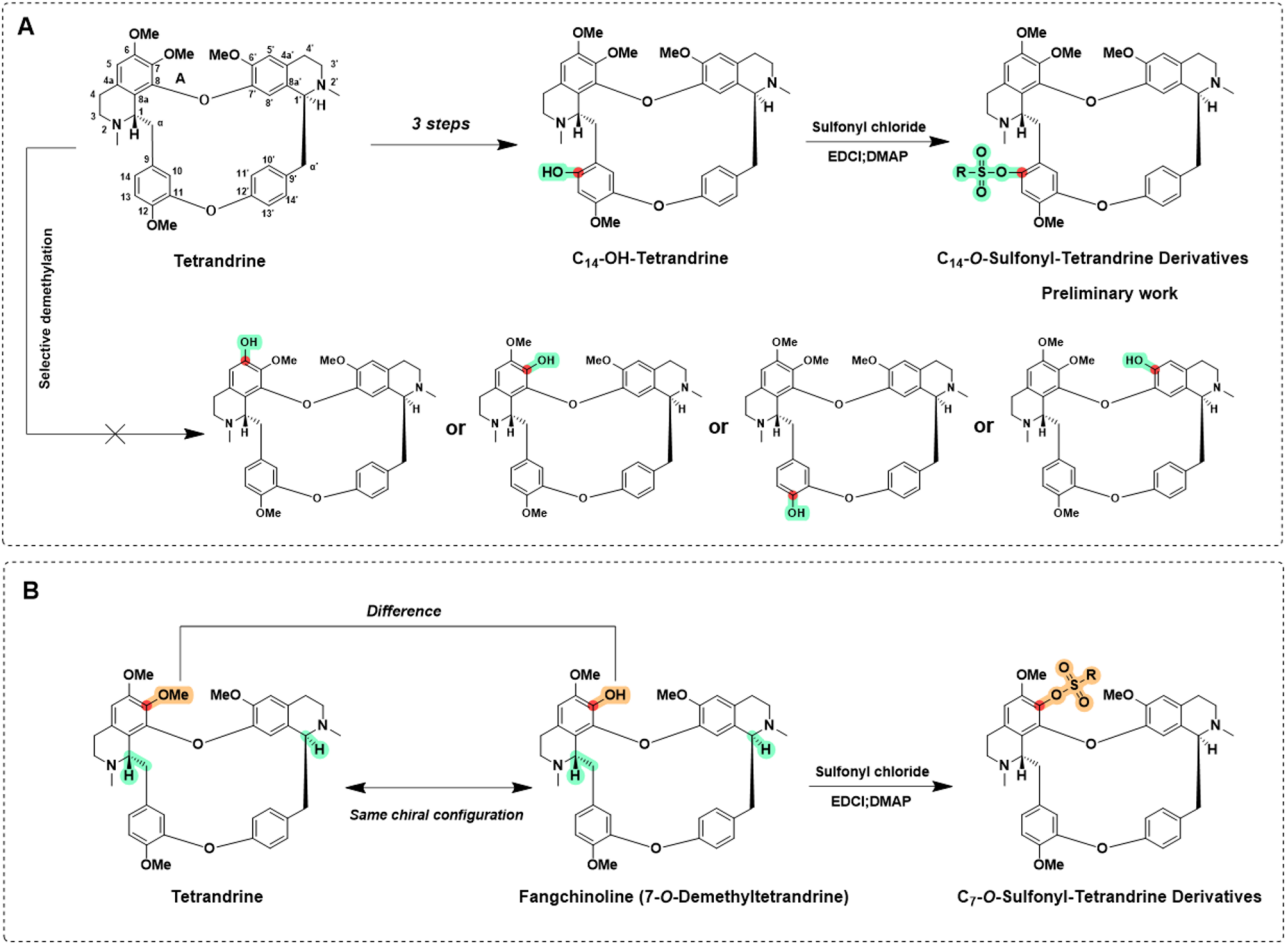
Synthetic strategy for C-7 sulfonate derivatives of tetrandrine. (**A**) Selective removal of a single methyl group from the tetrandrine scaffold is highly challenging; (**B**) General synthetic route of C7-tetrandrine derivatives. RSO_2_Cl, EDCI, DMAP, CH_2_Cl_2_, 0 °C., 8 h.

Tetrandrine, an eighteen-membered macrocyclic compound, is formed by the ether linkage of two benzylisoquinoline units. Its structure is characterised by the presence of four methoxy (-OCH_3_) and two methyl (-CH_3_) substituents, conferring overall molecu-lar stability. Although our previous studies successfully synthesized C-14 sulfonate substituted tetrandrine derivatives, the introduction of sulfonate groups at other positions, such as C-7, presented significant challenges. To address this, we attempted selective demethylation of the methoxy groups in tetrandrine, aiming to generate re-active hydroxyl groups for subsequent sulfonate modification. However, LC-MS analysis revealed the presence of multiple demethylation by-products (e.g., mono-demethylated and bis-demethylated isomers) in the reaction system, indicating that traditional chemical methods are inadequate for achieving site-selective modification, as shown in Fig. 1A.

Notably, the natural homologue 7-O-Demethyltetrandrine (Fangchinoline) has provided a novel approach to addressing this challenge. The structures of tetrandrine and fangchinoline are highly homologous, differing only at the C-7 substituent^37^: tetrandrine features a methoxy group, whereas fangchinoline possesses a hydroxyl group (-OH), with the remaining structural features, including the configuration of chiral centers, being identical. Taking advantage of the high reactivity of the C-7 hydroxyl group in fangchinoline, we have successfully constructed 38 structurally well-defined C-7 sulfonate derivatives, including 31 new compounds, by nucleophilic substitution reactions using various substituted sulfonyl chlorides (detailed structures are shown in Fig. 1B). This strategy effectively circumvents the complex selective demethylation steps, offering a streamlined route to functionalization.

### Biological evaluation

#### In vitro cytotoxicity assay

This study utilized 5-fluorouracil (5-Fu), a drug commonly used in clinical practice, as a positive control to measure the inhibitory activity of the synthesized derivatives against 4 HCC cell lines with the MTT colorimetric assay. The results, as presented in the Table 1, demonstrated that the sulfonate derivatives exhibited higher inhibitory activity against HCC cells compared to Fan and 5-fluorouracil. However, some derivatives showed reduced activity compared to the parent compound. It is noteworthy that, compared with the other derivatives, derivative 15 showed the most significant inhibitory effect on four types of liver cancer cells, and its in vitro anticancer activity against HCC far exceeded that of the commonly used anticancer drug 5-fluorouracil by a factor of more than 10. Taking these key findings into account, compound **15** has been selected as a prime candidate for further investigation of its biological activity due to its outstanding advantages.

**Table 1:**
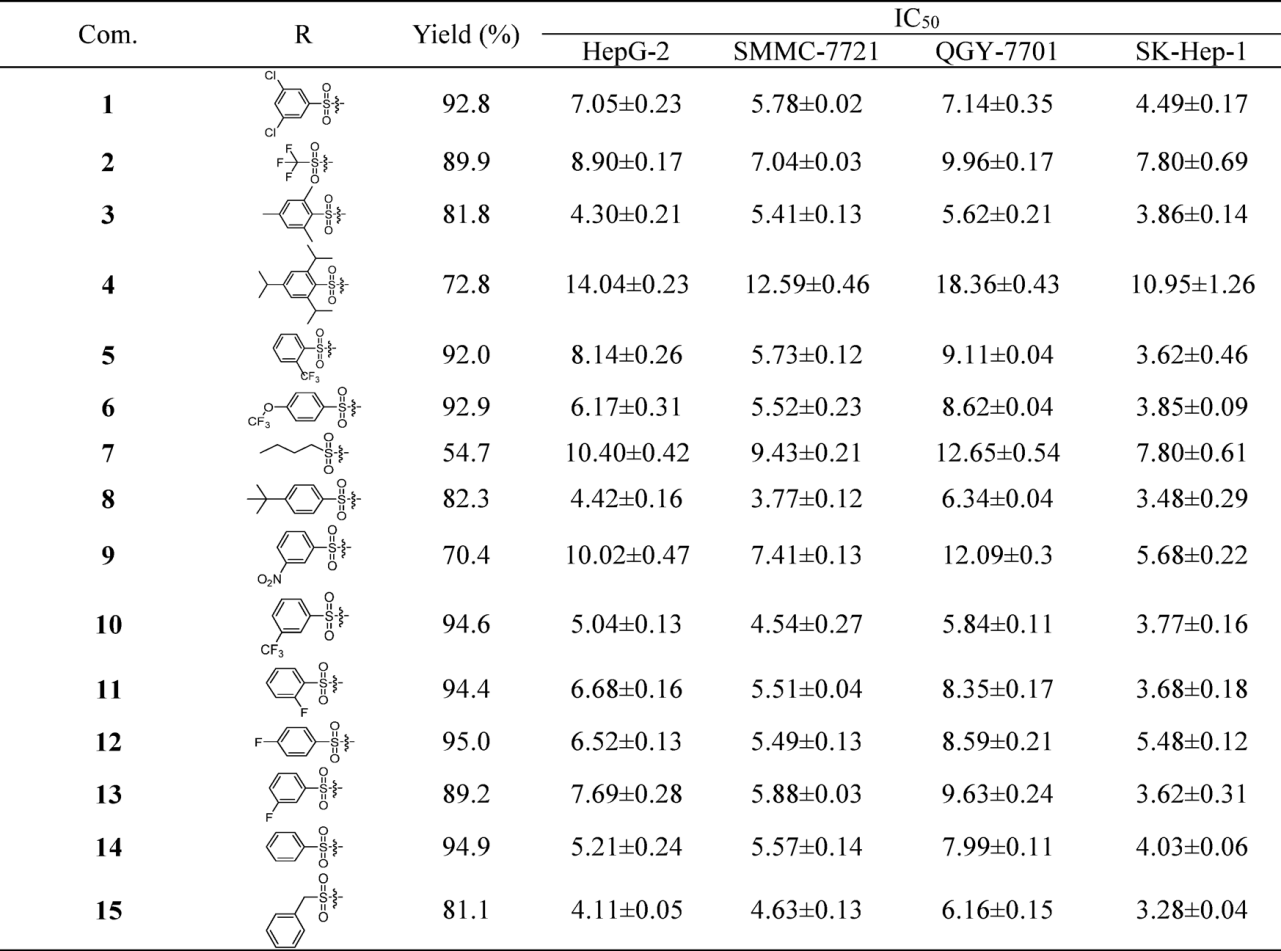

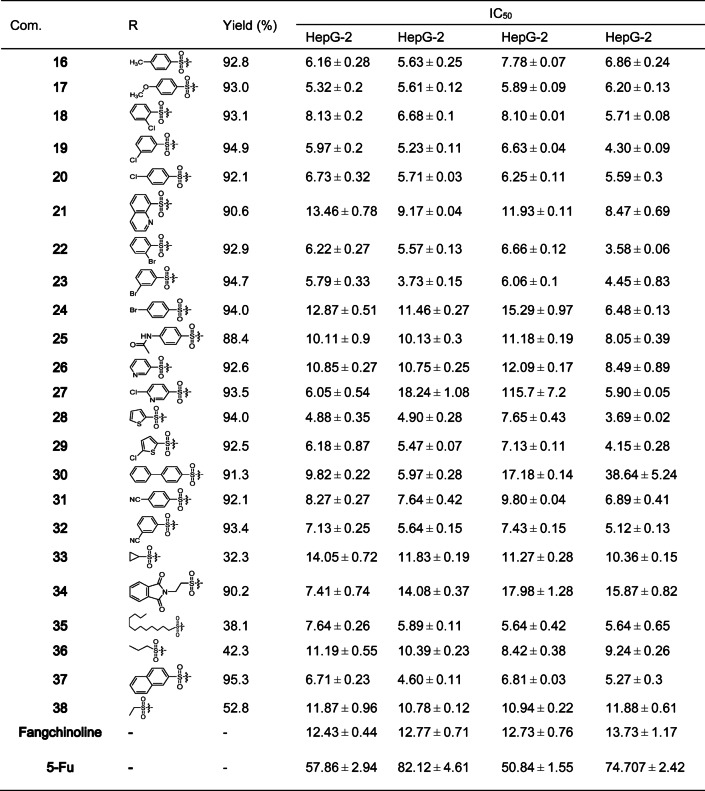
Cytotoxic effects of the test compound on HepG-2, SMMC-7721, QGY-7701, and SK-Hep-1 hepatocellular carcinoma cell lines after 48 h of exposure.

#### Structure-activity relationship

**Fig. 2:**
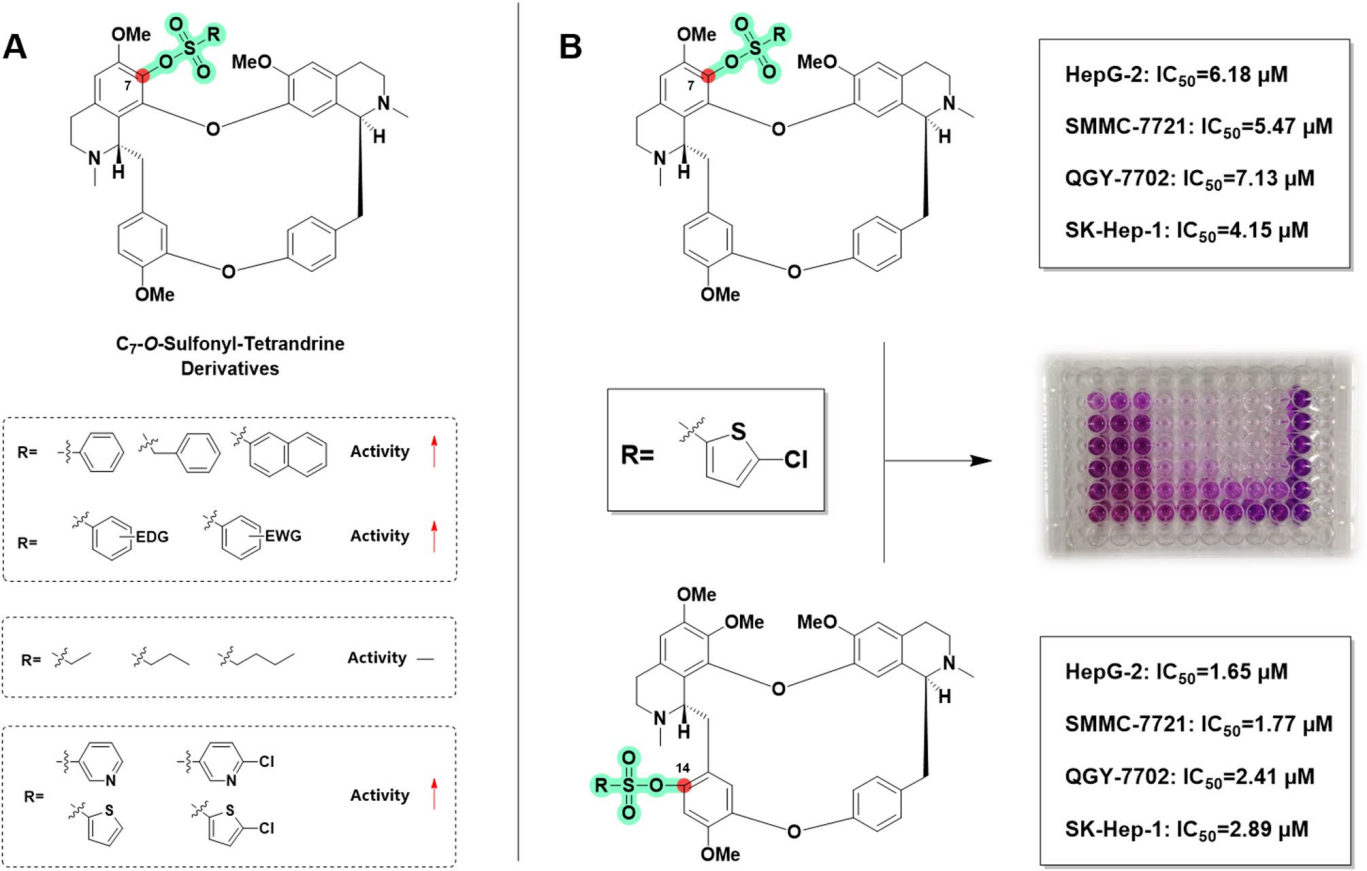
Analysis of the relationship between structure and activity for C-7 sulfonate tetrandrine derivatives. (**A**) Contribution of C-7 substituted groups in tetrandrine to anti-HCC activity; (**B**) IC₅₀ of 5-chlorothiophene-2-sulfonyl substituted at C-7 and C-14 positions of tetrandrine against four HCC cell lines.

This study systematically investigated the structure-activity relationship (SAR) of different sulfonate group substitutions on the antitumor activity based on the IC_50_ results of 38 C-7 tetrandrine derivatives. Experiments involving the introduction of aliphatic, aromatic, and heterocyclic sulfonate groups at the C-7 position of tetrandrine revealed that aromatic rings (phenyl, benzyl, naphthyl) and heterocyclic substituents significantly enhanced the anti-HCC activity of the parent compound, with IC_50_ values consistently around 5 µM. In contrast, the introduction of aliphatic sulfonate groups did not exhibit any activity enhancement. Notably, in the aromatic sulfonate series, which accounted for 70% of the synthesized substances, the inhibitory potential against the four HCC cell lines (IC_50_: 4–8 µM) was comparable to that of the unsubstituted phenyl sulfonate derivative tetrandrine, regardless of whether the benzene ring substituents were electron-withdrawing groups (-X, -NO_2_, -CN, -CF_3_) or electron-donating groups (-CH_3_, -OCH_3_). This finding is highly consistent with our previous research conclusions, further confirming that the type of aromatic ring substituent has no significant impact on activity, as shown in Fig. 2A.

In the comparison of heterocyclic systems, five-membered heterocycles exhibited stronger biological activity than six-membered heterocycles, a trend that aligns with previous research. Notably, although earlier literature reported that tetrandrine derivatives with 5-chlorothiazole sulfonate introduced at the C-14 position demonstrated significant inhibitory effects against the four HCC cell lines (IC_50_: 1.65–2.89 µM), the same substituent introduced at the C-7 position in this study did not exhibit equally prominent activity, as shown in Fig. 2B. Based on previous research findings^36^, we have compiled the IC_50_ of tetrandrine derivatives bearing various sulfonate ester substitutions at the C-7 and C-14 positions against four HCC cell lines. The comparative analysis showed that most C14-substituted tetrandrine derivatives had much stronger inhibitory effects on HCC cells, with IC_50_ values between 1 and 8 µM. In contrast, C-7 substituted derivatives demonstrated comparatively weaker activity, with IC_50_ values between 4 and 12 µM. These results suggest that the C-14 position of tetrandrine holds greater potential for structural modification. This finding offers crucial insights for optimizing the molecular design of lead compounds, highlighting its importance in advancing future research.

#### Compound **15** inhibits HepG-2 cells colony formation

**Fig. 3:**
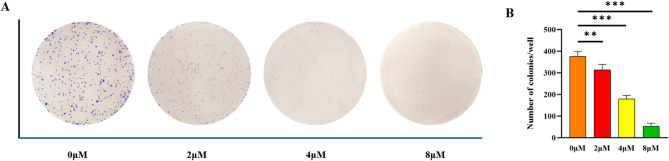
Compound **15** exhibits inhibitory effects on the proliferation of HepG-2 cells in vitro. (**A**) Compound **15** was tested on HepG-2 cells at concentrations of 0, 2, 4, and 8 µM to evaluate its effect on colony formation; (**B**) Data are expressed as the mean ± SD from three independent trials, where **p < 0.01 and ***p < 0.001 signify statistical significance.

To confirm the inhibitory capability of compound **15** on the growth of liver cancer cells, we used HepG-2 cells for the colony formation test. As shown in Fig. 3, compound **15** demonstrated an effective inhibition of HCC proliferation that varied with dose. Treatment of HepG-2 cells with 4 µM concentration of **15** for 48 h reduced cell viability by 50%. Follow-up plate colony formation assay showed that in the presence of **15**, the HepG-2 cells’ capacity to form colonies diminished as the concentration of **15** rose, resulting in only a small number of colonies at 8 µM.

#### Compound **15** inhibits HepG-2 cell migration

**Fig. 4:**
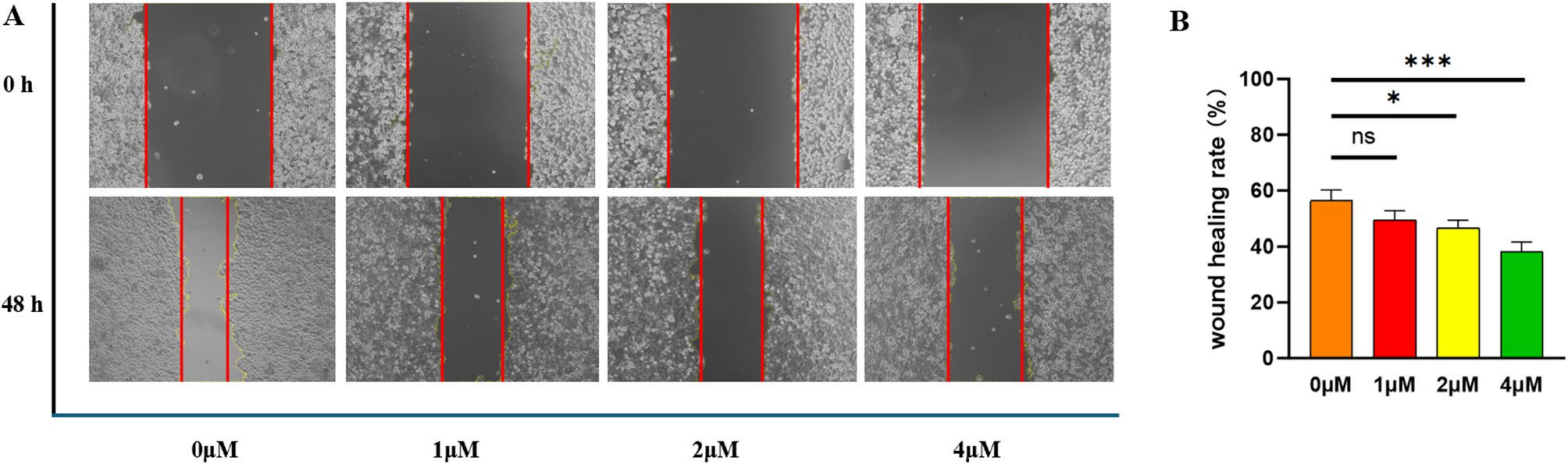
Compound **15** exhibits inhibitory effects on the wound healing of HepG-2 cells in vitro. (**A**) Compound **15** was tested on HepG-2 cells at concentrations of 0, 1, 2, and 4 µM to evaluate its effect on wound healing; (**B**) The results are presented as the mean ± SD from three independent experiments, with *p < 0.05 and ***p < 0.001 indicating statistical significance.

A scratch wound assay was conducted to study the influence of compound **15** on the migration of HepG-2 cells. Experimental results elucidated that in control groups, the scratch area was nearly fully repopulated after 48 h, with wound closure rates of 56.4 ± 3.7%. Upon treatment with varying concentrations of compound **15** (1, 2, and 4 µM), the wound closure rate was significantly lowered, and cellular migration was greatly inhibited as the levels of compound **15** went up. By comparing the experimental results of the control group with those of the treatment groups at varying concentrations, it can be concluded that compound **15** exhibits a significant inhibitory effect on the migration of HepG-2 cells under in vitro conditions, as shown in Fig. 4.

#### Induction of apoptosis by compound **15** in HepG-2 cells

**Fig. 5:**
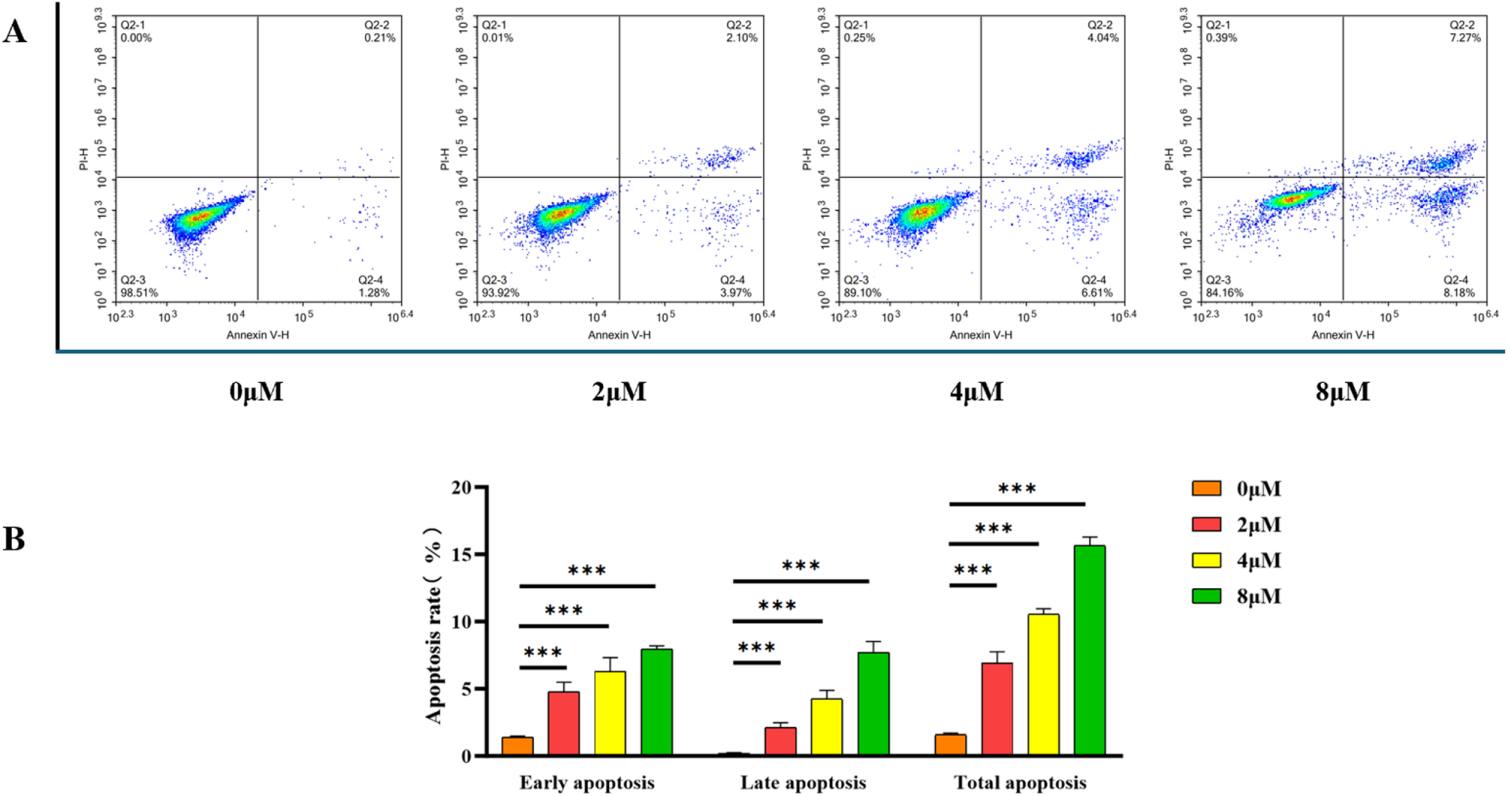
In vitro studies demonstrate that compound **15** triggers apoptosis in HepG-2 cells. (**A**) The apoptosis of HepG-2 cells exposed to different doses of compound **15** (0, 2, 4, and 8 µM) was examined using flow cytometry; (**B**) results are reported as the mean ± SD from three independent studies, with ***p < 0.001 indicating a statistically significant difference.

In order to assess the effect of compound **15** on apoptosis in HepG-2 cells, this study employed the Annexin V-FITC/PI double staining method. This assay, widely recognized for its efficacy in detecting apoptosis, enables the clear distinction between early apoptotic and necrotic cells, thereby enhancing the accuracy and reliability of the experimental findings. The results of the experiment demonstrated a significant rise in the apoptosis rate of HepG-2 cells after 48 h of exposure to Compound **15**, which varied with concentration. Specifically, in the control group (0 µM), the early and late apoptosis rates were 1.28% and 0.21%, respectively. When the concentration increased to 2 µM, the apoptosis rates rose to 3.97% and 2.10%. At 4 µM concentration, the apoptosis rates further increased to 6.61% and 4.04%, respectively. When the concentration reached 8 µM, the apoptosis rates reached 8.18% and 7.27%, as shown in Fig. 5. These findings indicate that apoptosis in HepG-2 cells can be caused by compound **15** in a concentration-dependent way.

#### Compound **15** induces apoptosis in HepG-2 cells via the mitochondrial pathway

**Fig. 6:**
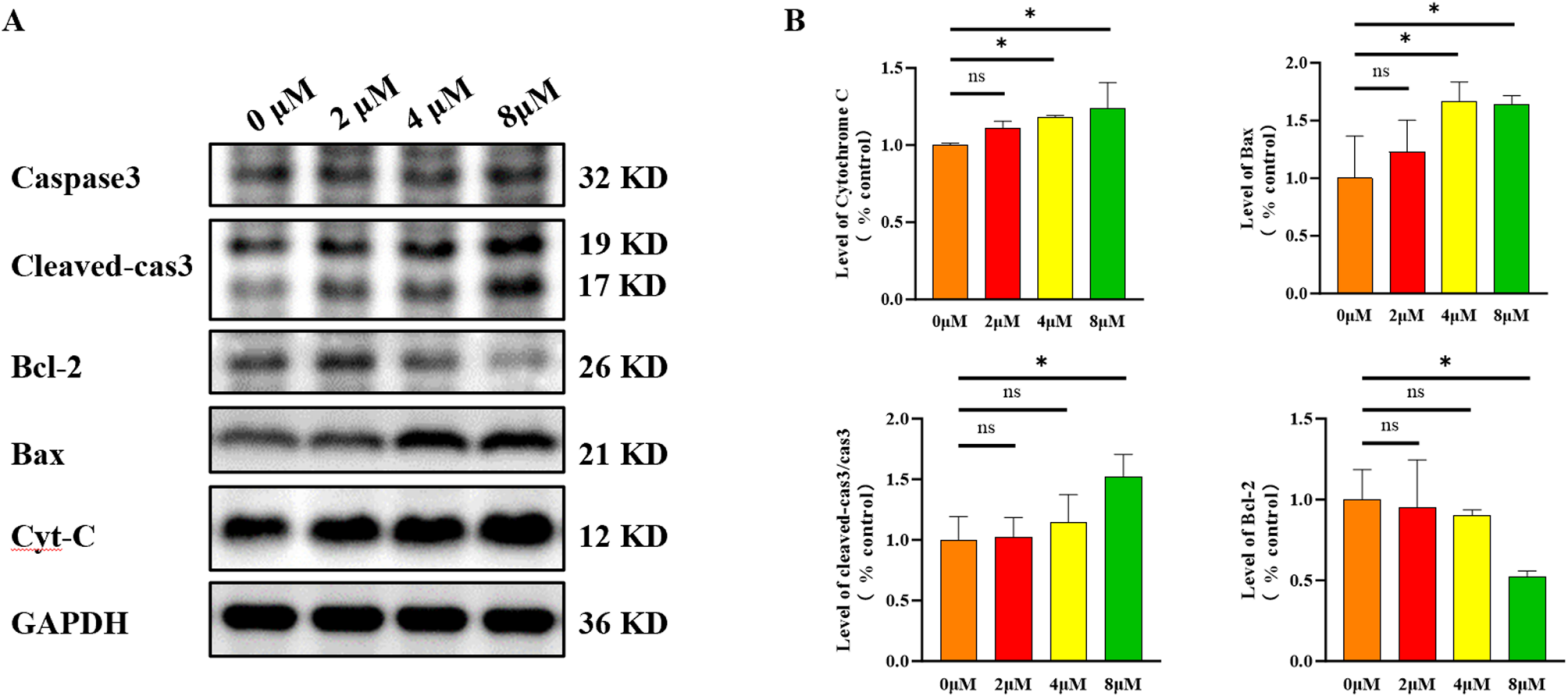
Compound **15** triggers apoptosis in HepG-2 cells through the mitochondrial pathway. (**A**) Western blotting demonstrated that compound **15** upregulates the expression of mitochondrial pathway proteins in HepG-2 cells; (**B**) The results are presented as the mean ± SD from three independent experiments, with *p < 0.05 indicating statistical significance.

To clarify the molecular pathways through which compound **15** causes apoptosis in HepG-2 cells, this research used Western blot analysis to examine the levels of proteins involved in apoptosis. HepG-2 cells were exposed to compound **15** at concentrations of 0, 2, 4, and 8 µM for 48 h, followed by preparation of total cell lysates for Western blot analysis. As shown in Fig. 6, a significant drop in Bcl-2 protein expression was observed after 48 h of treatment with 8 µM of compound **15**, accompanied by an increase in Bax and cytochrome C protein levels. This observation suggests that compound **15** may influence mitochondrial and endoplasmic reticulum function by modulating the expression of Bax and Bcl-2, thereby facilitating the release of cytochrome C. The release of cytochrome C is a pivotal step in the mitochondria-mediated apoptotic pathway. Cytochrome C, once it is released, can attach to apoptotic protease-activating factor-1 (Apaf-1)., thereby initiating the downstream caspase cascade, which ultimately leads to apoptosis^38^. Furthermore, we measured cleaved caspase-3 levels, which demonstrated a dose-dependent rise with increasing amounts of compound **15**. This indicates that compound **15** induced apoptosis in HepG-2 cells is indeed mediated through the caspase cascade. In summary, compound **15** induces apoptosis in HepG-2 cells via the mitochondrial pathway, with its mechanism likely involving the modulation of Bax and Bcl-2 expression, the promotion of cytochrome C release, and the activation of downstream molecules such as caspase-3, ultimately bringing about cell apoptosis.

#### Prediction of the anti-HCC target of compound **15**

**Fig. 7:**
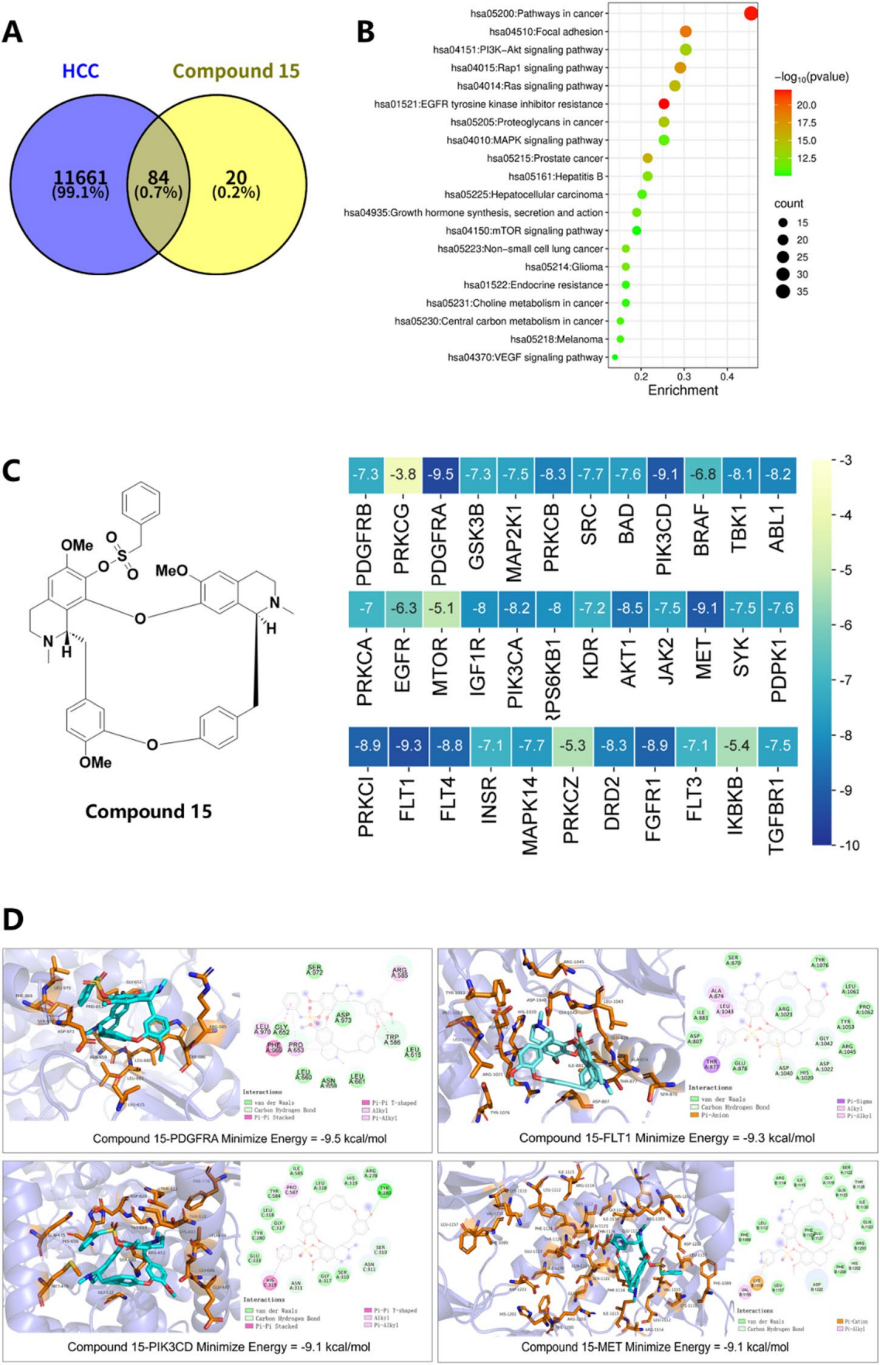
Target prediction and molecular docking simulation of compound **15** against HCC. (**A**) Compound **15** potential active target and HCC target intersection Venn diagram; (**B**) the result of KEGG enrichment analysis^39^; (**C**) molecular docking heatmap of the compound **15** and key targets for the treatment of HCC (Kcal/mol); (**D**) visualization of the molecular docking results of PDGFRA (− 9.5 kcal/mol), FLT1 (− 9.3 kcal/mol), PIK3CD (− 9.1 kcal/mol), and MET (− 9.1 kcal/mol).

To investigate potential targets of compound **15** against HCC, 11,745 HCC-related genes were retrieved from the GeneCards and OMIM databases using the keyword “Hepatocellular Carcinoma (HCC)”. A total of 104 candidate targets of compound **15** were identified using the SwissTargetPrediciton database. An intersection analysis between the HCC and compound **15**-related targets identified 84 overlapping key targets (Fig. 7A). KEGG pathway enrichment analysis of these targets (*P* < 0.05) revealed 145 significantly enriched signaling pathways. Among the top 20 enriched pathways (Fig. 7B), the PI3K-Akt, EGFR, MAPK, mTOR, Rap1, and Ras signaling pathways were prominent, suggesting compound **15**’s anti-HCC effects may involve modulation of these pathways. After removing duplicates, 35 unique core targets associated with the enriched pathways were selected for molecular docking analysis. The binding energy heatmap (Fig. 7C) showed that compound **15** exhibited binding energies below − 5 kcal/mol with more than 95% of selected targets. According to molecular docking theory, negative binding free energy values indicate spontaneous ligand-receptor interactions; and greater absolute values correspond to more stable complexes. These findings suggest that compound **15** has strong binding affinity for key therapeutic targets in HCC.

Visualization analysis of targets with binding energies below − 9 kcal/mol is presented in Fig. 7D. Compound **15** exhibited the strongest binding affinities with PDGFRA (− 9.5 kcal/mol), FLT1 (− 9.3 kcal/mol), PIK3CD (− 9.1 kcal/mol), and MET (− 9.1 kcal/mol). These high affinities primarily arises from van der Waals (vdW) interactions, which enhance ligand-receptor binding by filling structural voids at the protein-ligand interface. Key interacting residues include: SER972, ASP973 and GLY652 in PDGFRA; ARG1021, SER870 and TYR1076 in FLT1; TYR280, ARG278 and HIS319 in PIK3CD; SER1122, GLY1119 and ARG1203 in MET.

Additionally, C-H bonds was observed across all targets (e.g. TRP586 in PDGFRA; GLY1042, ASP1022 and ASP1040 in FLT1; ASN311 and SER310 in PIK3CD; THR1126, ASP1222 and HIS1202 in MET), facilitating ligand embedding into vdW-driven hydrophobic pockets, thereby minimizing solvent exposure and stabilizing binding conformations. Ubiquitous π-alkyl interactions (e.g. LEU970, PRO653 and ARG585 in PDGFRA; ALA874 and LEU1043 in FLT1; PRO587 in PIK3CD; VAL1155 in MET) further contributed to hydrophobic stabilization and complex rigidity.

Beyond general hydrophobic contacts, benzene ring-mediated specific interactions significantly enhanced targets selectivity. In PDGFRA and PIK3CD, T-shaped π-π stacking with PHE969 and HIS319, respectively, constrained by spatial architecture of the binding pockets, helped reduce off-target binding. This structural alignment synergized with surrounding hydrogen bonds, π-alkyl contact interface and improve both binding and specificity. In FLT1, π-σ interactions with THR877 further enhanced binding through vdW-mediated aromatic stabilization. For MET, an electrostatic π-cation interaction with LYS1110 played a pivotal role in ligand recognition and affinity enhancement.

In summary, compound **15** demonstrated favorable binding profiles with all 35 evaluated targets, with PDGFRA, FLT1, PIK3CD, and MET emerging as the most promising pharmacological targets underlying its anti-HCC activity.

## Materials and methods

### Materials and instruments

Fangchinoline, having a purity of at least 97%, was obtained from Nanjing Jingzhu Biotechnology Company, based in Nanjing, China. The chemicals and solvents used were obtained from commercial vendors, J&K Scientific, based in Beijing, China. These materials were utilized without further purification, except where specified. Dichloromethane needs to be dried using calcium hydride and subsequently distilled in an argon environment before use. Thin layer chromatography on silica gel GF-254 was used to track the reaction’s progress, while column chromatography with silica gel (300–400 mesh, Haohong Biomedical Technology Co., Ltd) was employed for separation and purification. Visualization was achieved using UV light at a wavelength of 254 nm or a 10% solution of phosphomolybdic acid hydrate in ethanol. ^1^H NMR, ^13^C NMR, and ^19^F NMR spectra were recorded using Bruker AVANCE III 600 MHz spectrometers in CDCl_3_. HRMS data were obtained with the aid of a Thermo Scientific Q Exactive Focus instrument, produced by Thermo Fisher Scientific in the USA. A YRT-3 micro melting point instrument from JingTuo in Tianjin, China, was used to determine the melting points.

### Synthesis of C-7 tetrandrine derivatives

In the process of synthesizing derivatives, 0.164 mmol of Fangchinoline (1.0 equivalent), 0.246 mmol of EDCI (1.5 equivalents), and 0.0328 mmol of DMAP (0.2 equivalent) were dissolved in 2 mL of anhydrous dichloromethane with argon atmosphere. The mixture was rapidly stirred in an ice bath and cooled to 0 °C, followed by the gradual addition of 0.246 mmol of sulfonyl chloride (1.5 equivalents) at this temperature. Then raised to 25 °C and stirred for a further 8 h. Upon completion of the reaction (monitored by TLC), the mixture was quenched with ice-cold water (5 mL). The organic phase was separated using a separating funnel, dried over Na_2_SO_4_, filtered, and concentrated under reduced pressure to afford the crude product. The target derivative was purified by flash column chromatography on silica gel, using a solvent system of dichloromethane (80 vol.) and methanol (1 vol.) containing 1% triethylamine. Comprehensive NMR (^1^H, ^13^C, and ^19^F) and HRMS data for the 38 C-7 tetrandrine derivatives synthesized are provided in the Supplementary Material.

### Cell lines and cell culture

The cell lines used in this study were sourced from Procell Life Science and Technology Company in Wuhan, China. All cell lines were cultured in Dulbecco’s modified Eagle’s medium (Gibco, USA) with 10% fetal bovine serum and a mix of streptomycin and penicillin (Solarbio, Beijing, China). A humidified incubator (Thermo Scientific, USA) at 37 °C with 5% CO₂ was used to culture the cells.

### In vitro cytotoxicity assay

The MTT assay was adopted to determine the IC_50_ values of the compounds against four cell lines. In brief, 5,000 cells were seeded into each well of a 96-well plate. After 12 h, the cells were treated with different concentrations of the compound. Following a 48-hour incubation at 37 °C, 20 µL of MTT (5 mg/mL) solution was added to each well, and the plates were incubated for an additional 4 h. The supernatant was then removed, and 150 µL of DMSO was added to dissolve the crystals. The absorbance was measured at 490 nm using a microplate reader. The calculation of the IC_50_ value was performed with GraphPad Prism 8 software, utilizing a nonlinear regression and a dose-response inhibition equation.

### Colony formation assay

Log-phase HepG-2 cells (1,000/well) were seeded in six-well plates. The plates were kept at 37 °C in a humid environment with 5% CO_2_ for 7 days until visible colonies developed. Then, they were treated with compound **15** (0, 2, 4 and 8 µM) for 10 days. Subsequently, the plates were washed with PBS, fixed with 4% paraformaldehyde (Servicebio, Wuhan, China) solution for 15 min, and stained with 0.04% Giemsa stain for 15 min.

### Wound healing assay

A uniform distribution of HepG-2 cells was achieved in six-well plates, and they were cultured until a monolayer developed. The monolayer was scratched with a sterile pipette tip to create a wound, and initial images were obtained using an inverted microscope (Olympus, Tokyo, Japan). Following a wash with phosphate-buffered saline (PBS) to clear away cellular debris, the cells received treatment with compound **15** at 0, 1, 2, and 4 µM concentrations. After 48 h of incubation with the drug-containing medium, additional images were taken to assess wound closure.

### Flow cytometry analysis

HepG-2 cells, while in their exponential growth phase, were introduced into six-well plates and cultured to 80% confluence. The cells were then treated with varying concentrations (0, 2, 4, and 8 µM) of compound **15** for 48 h. Following incubation, the cells were gently washed three times with phosphate-buffered saline (PBS) and subsequently detached using EDTA-free trypsin. The cell suspension was adjusted to a density of (1–5) × 10⁶ cells/mL, followed by centrifugation at 800 rpm for 5 min. After discarding the supernatant, the cell pellet was resuspended in binding buffer. Apoptotic cells were then stained with Annexin V-FITC and propidium iodide (PI) according to the manufacturer’s instructions and analysed by flow cytometry using an Agilent Technologies instrument (Agilent, USA). All cultures and experiments were repeated three times. Quantitative analysis was performed using NovoExpress software (version 1.6.0).

### Western blotting

After total protein extraction from the cells, protein quantification was performed, and the protein concentration was adjusted to ensure a final concentration of 5 µg/µL for all experimental samples. Subsequently, the samples were processed following the previously described experimental method^32^. Proteins were separated by SDS-PAGE (Servicebio, China) and transferred onto PVDF (Merck Millipore, Germany) membranes. The membranes were incubated with primary antibodies specific to apoptosis-related proteins, followed by incubation with corresponding secondary antibodies. Bax antibody (60267-1-Ig), Bcl-2 antibody (68103-1-Ig), Caspase-3 antibody(66470-2-Ig), and cytochrome C antibody (66264-1-Ig) were purchased from Proteintech (Wuhan, China). Specific protein bands were visualized using an ECL substrate kit (Merck Millipore, Germany), and the density of the bands was analyzed. GAPDH was used as a housekeeping protein to serve as a loading control for normalization.

### Target prediction

Potential targets of compound **15** in homo sapiens were predicted using Swiss Target Prediction database (http://www.swisstargetprediction.ch/index.php), followed by standardization of gene symbols to official nomenclature via UniProt. Then, the keyword “Hepatocellular Carcinoma” was searched in Gene Cards and OMIM with the species restricted to Homo sapiens; the retrieved entries were merged and duplicates were removed to establish a disease-related target set. The intersection between the predicted targets of compound **15** and the disease-target set was subjected to KEGG pathway enrichment analysis and visualization in DAVID 6.8 (https://davidbioinformatics.nih.gov/) using the human background. Crystal structures of the HCC candidate proteins were retrieved from the RCSB PDB database (http://www.pdb.org/). All protein structures were pre-processed in AutoDock Tools 1.5.7 by adding hydrogen atoms, removing crystallographic water molecules, deleting non-essential ligands, and adjusting the conformations of key active-site residues; the processed structures were saved in PDBQT format. The 3D structure of compound **15** was optimized for energy using the MMFF94 force field, and the conformation with the minimum energy was saved in MOL2 format. All rotatable bonds were retained and their parameters defined in AutoDock Tools 1.5.7 (Scripps Research, USA) before conversion to the PDBQT format required for docking. Molecular docking was performed with AutoDock Vina 1.1.2 (Scripps Research, USA), and the resulting complexes were visualized using PyMOL 2.5.2 (Schrödinger, USA) and Discovery Studio 2019 (Dassault Systèmes BIOVIA, France).

### Statistical analysis

The results of all experiments are expressed as mean ± standard deviation (SD) of three independent replicates. GraphPad Prism software (version 8.0.2) was used to conduct the statistical analysis. Normality of all experimental datasets was confirmed via the Shapiro-Wilk test prior to employing one-way ANOVA to statistically analyze differences between experimental and control groups. Statistical significance was determined by a p-value under 0.05.

## Conclusions

In this study, 38 C-7 tetrandrine derivatives (including 31 novel compounds) were successfully synthesized, and their growth inhibitory effects against four human HCC cell lines were systematically evaluated through in vitro cytotoxicity assays. The study revealed that, despite the C-7 sulfonate derivatives exhibiting slightly lower overall anti-HCC activity in comparison to the C-14 sulfonate derivatives, their synthetic route offered remarkable benefits. Specifically, the reaction process was condensed into a single step, and the subsequent post-processing was more straightforward and environmentally benign, rendering it highly appropriate for large-scale industrial production. Notably, compound **15** was outstanding among all derivatives, with IC_50_ values of 4.12, 4.63, 6.16, and 3.28 µM against HepG-2, SMMC-7721, QGY-7701, and SK-Hep-1 cells, respectively, which was more than a several-fold enhancement in activity over the clinical first-line drug 5-fluorouracil. Mechanism studies have shown that the compound works by modulating the Bcl-2/Bax protein balance. This action drives the release of mitochondrial cytochrome C, thereby activating the caspase-3-dependent apoptotic pathway, which is similar to the previously reported derivative at C-14 position. Target prediction results indicated that PDGFRA, Flt1, PIK3CD and MET could be the primary targets of compound **15** in treating HCC. While the C-14 position derivatives exhibited slightly higher in vitro activity, the C-7 position tetrandrine derivatives, especially compound **15**, showed greater clinical potential when synthetic efficiency and industrial feasibility were considered. Follow-up studies will focus on further validating its potential in anti-HCC applications through in vivo pharmacodynamic evaluation and target identification.

## Supplementary Information

Below is the link to the electronic supplementary material.

Supplementary Material 1

Supplementary Material 2

## Data Availability

The NMR and HRMS data for all 38 derivatives are provided in the supplementary materials, further inquiries can be directed to the corresponding author/s.
