# Supplementary figures and images for: Investigating C7 modified tetrandrine derivatives for synthesis anti-hepatocellular carcinoma activity and mechanistic insights
Source: Sci Rep. 2025 Sep 29;15:33601. doi: 10.1038/s41598-025-18875-1 (PMC12480866; doi:10.1038/s41598-025-18875-1)

A-1

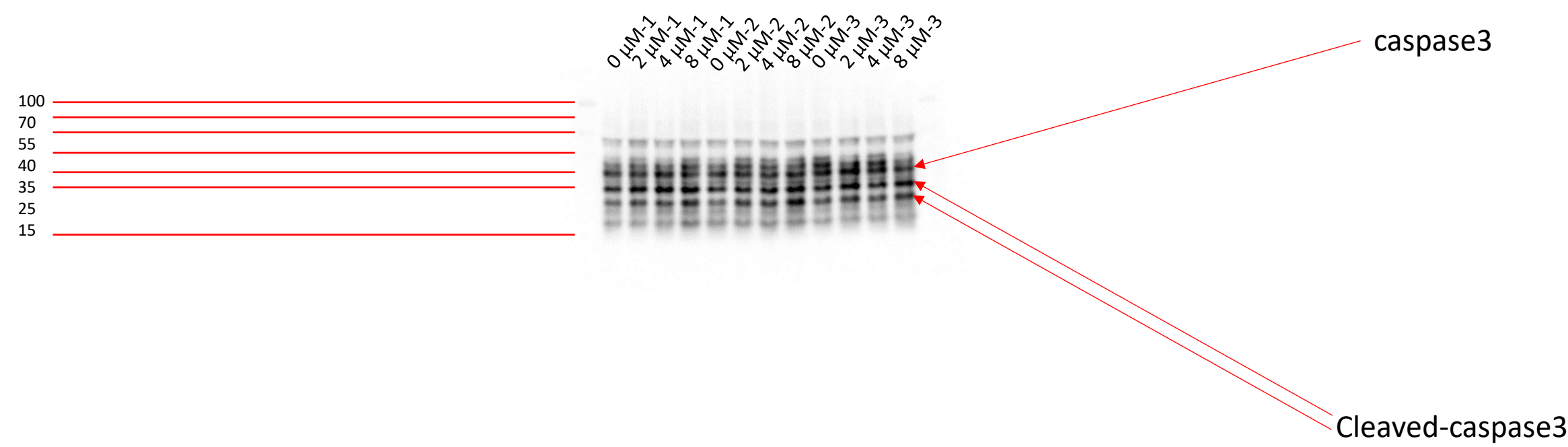

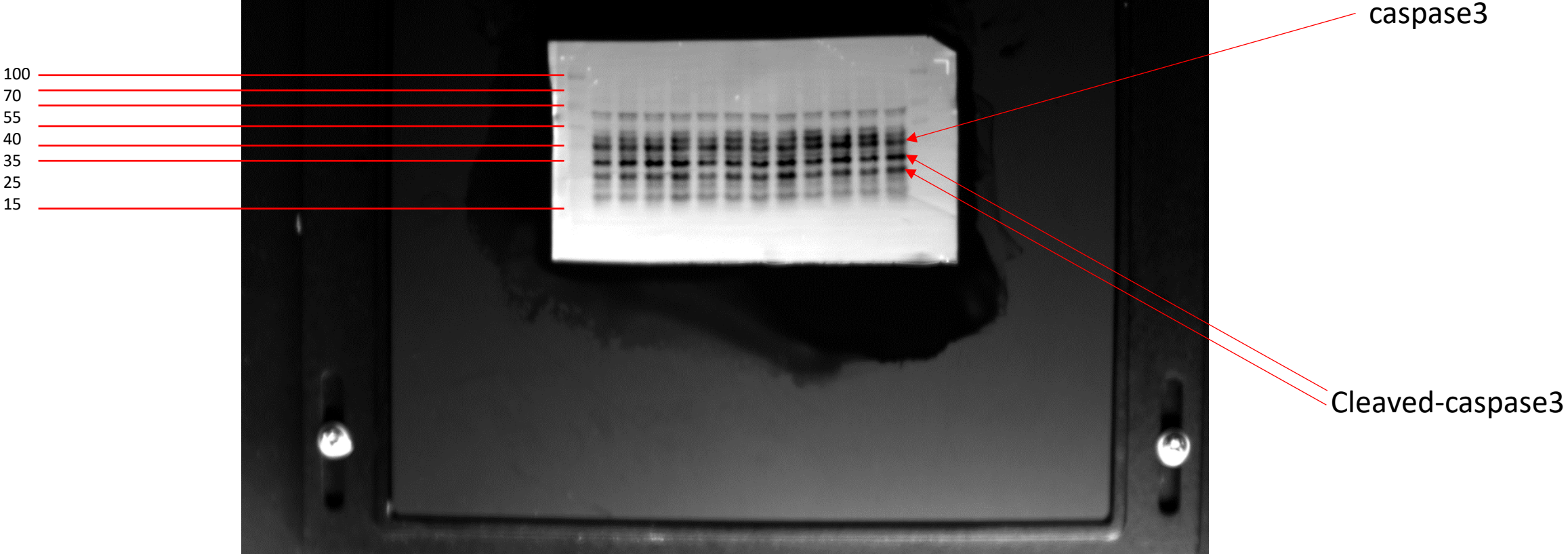

B-1

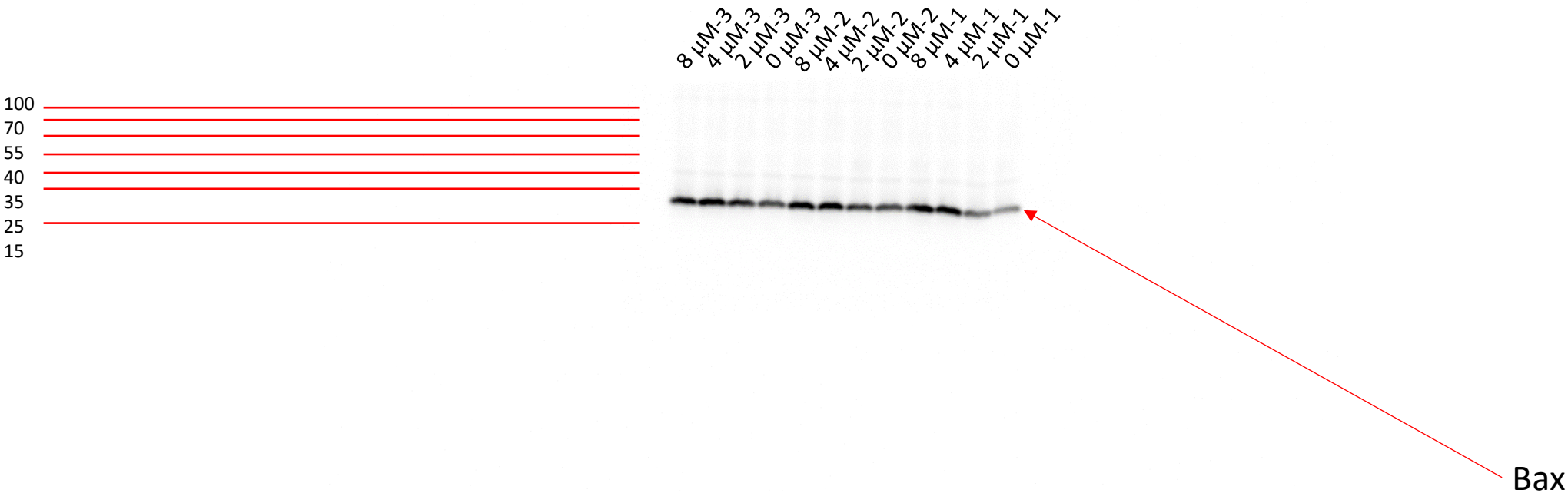

100  
70  
55  
40  
35  
25  
15

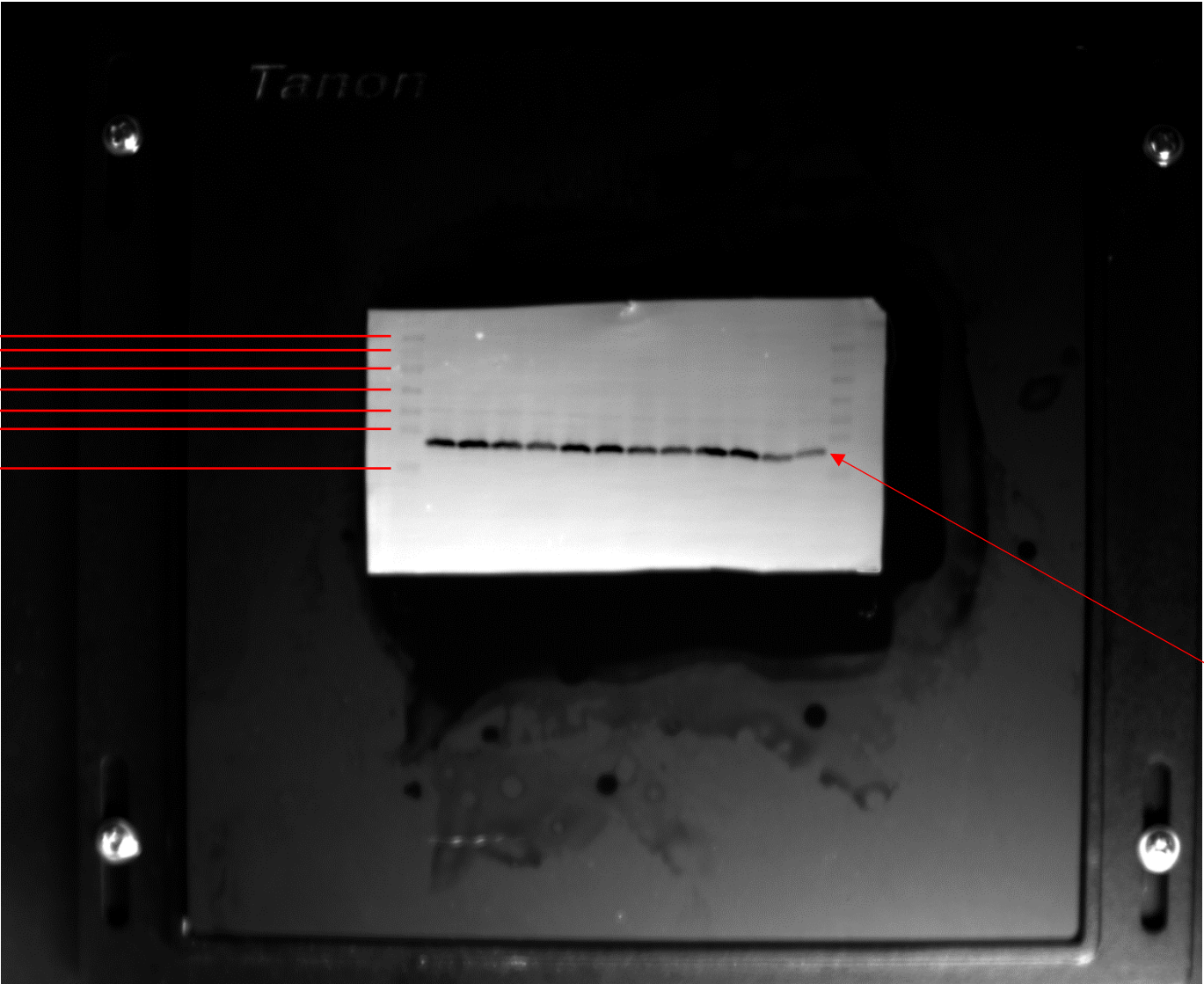

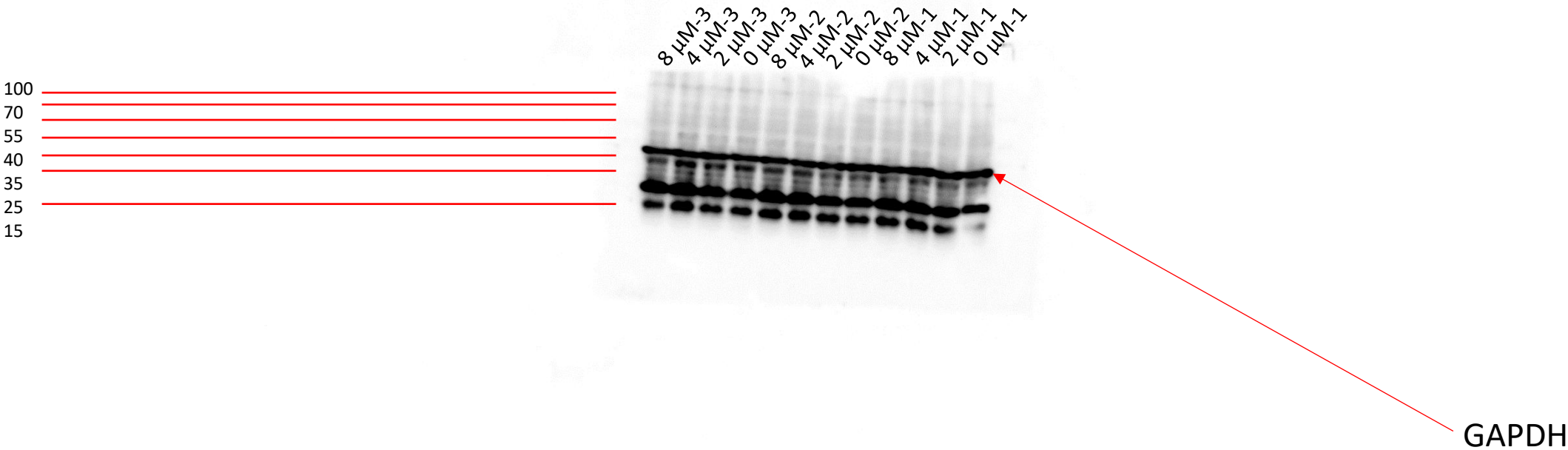

100  
70  
55  
40  
35  
25  
15

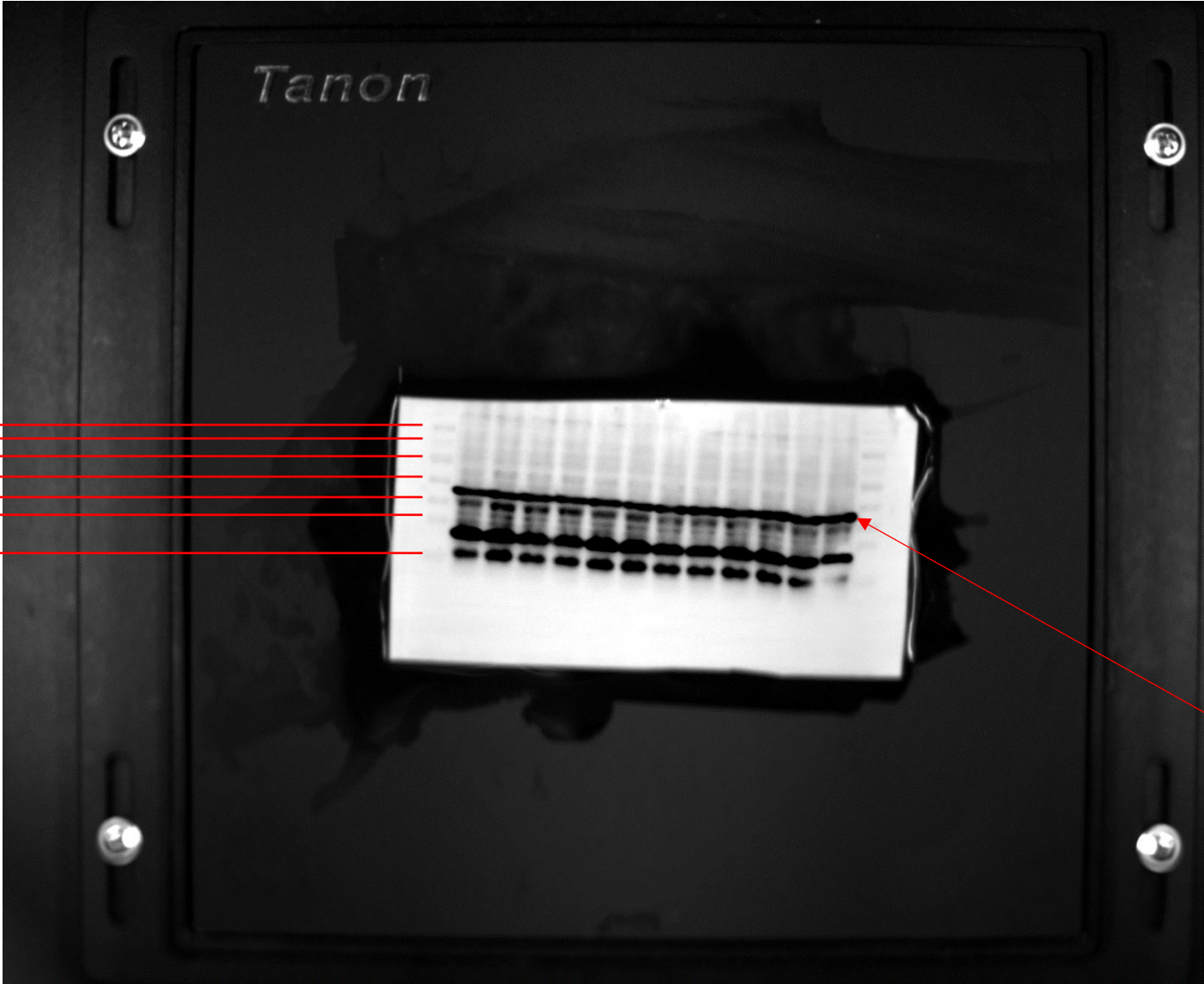

GAPDH

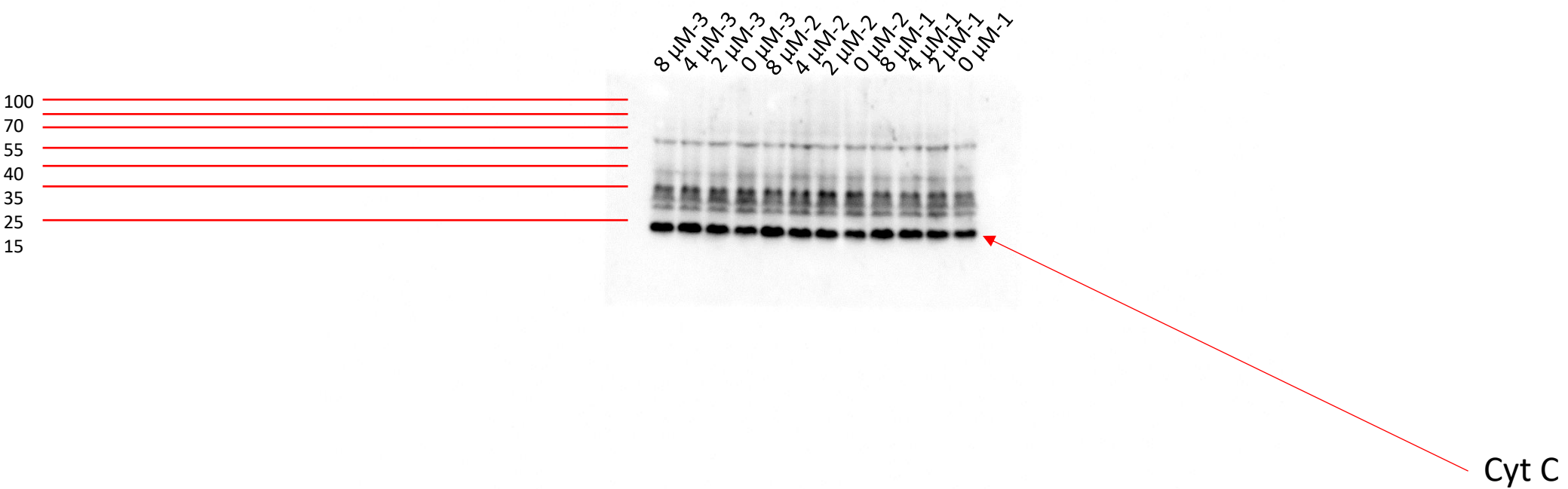

100  
70  
55  
40  
35  
25  
15

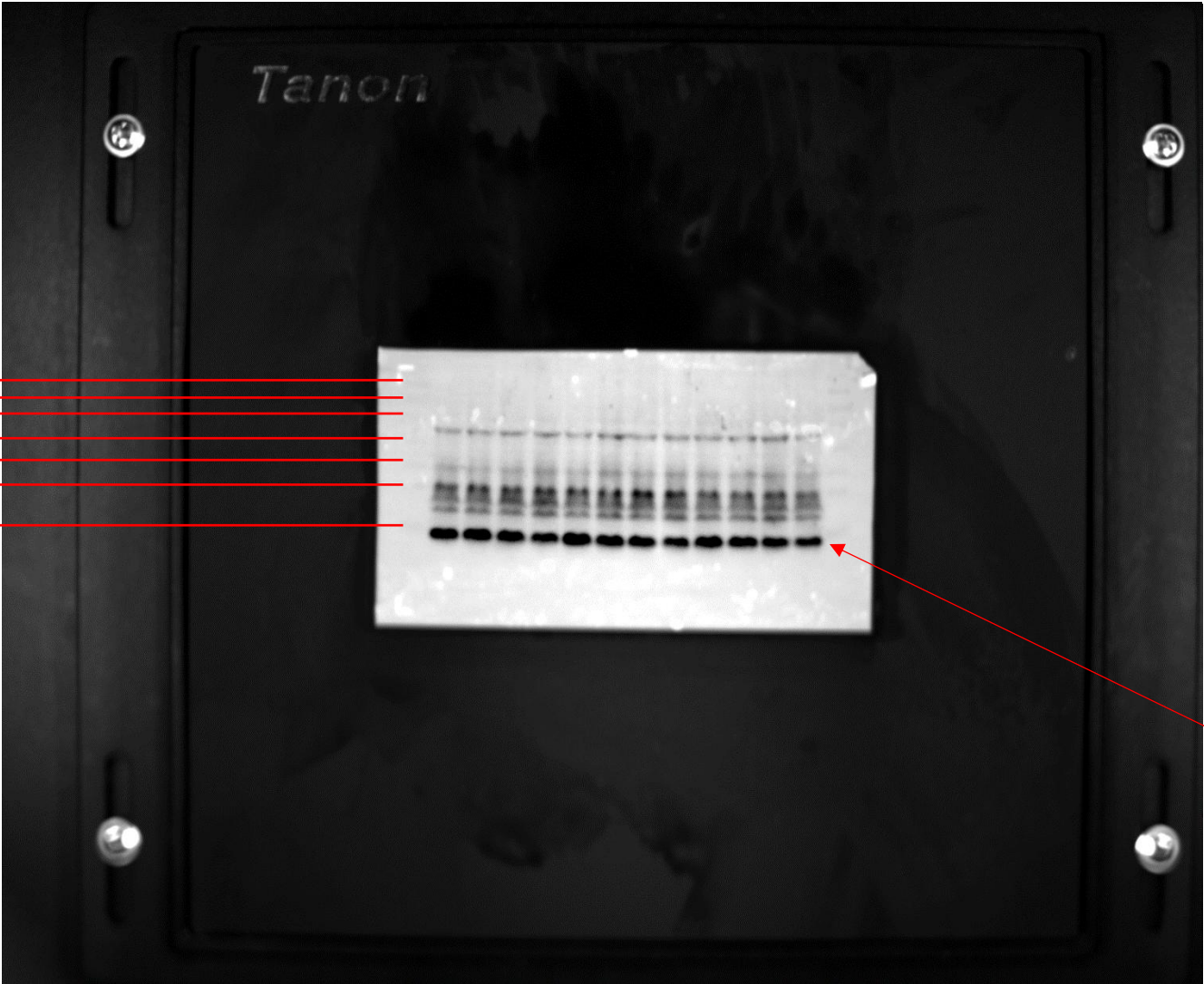

Cyt C

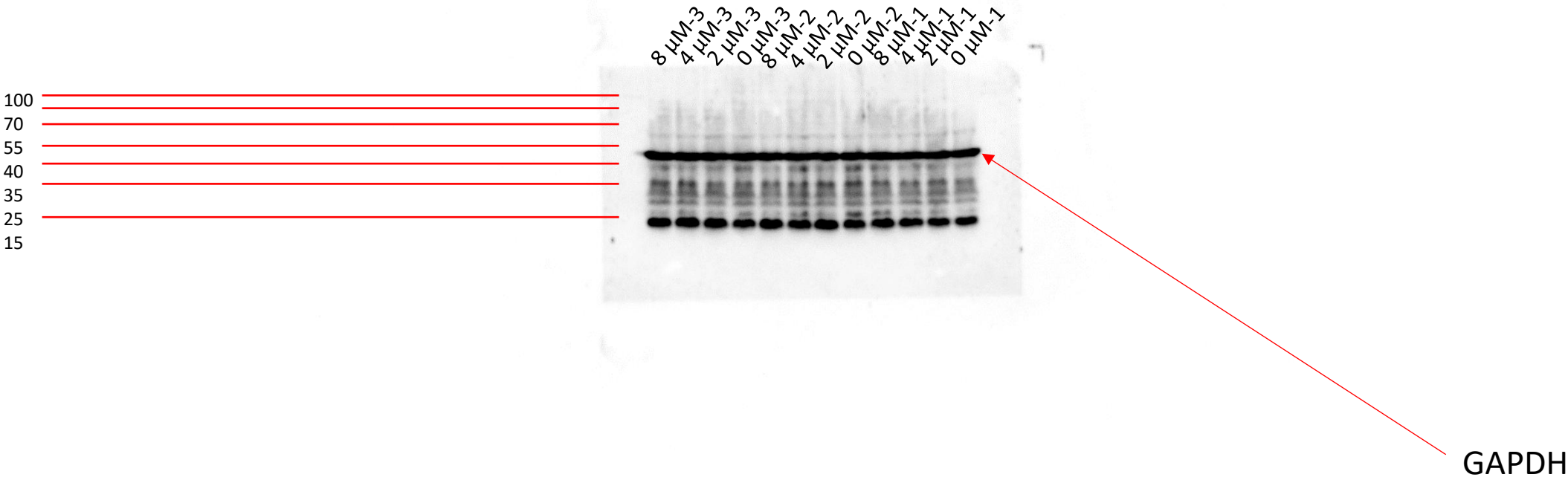

100  
70  
55  
40  
35  
25  
15

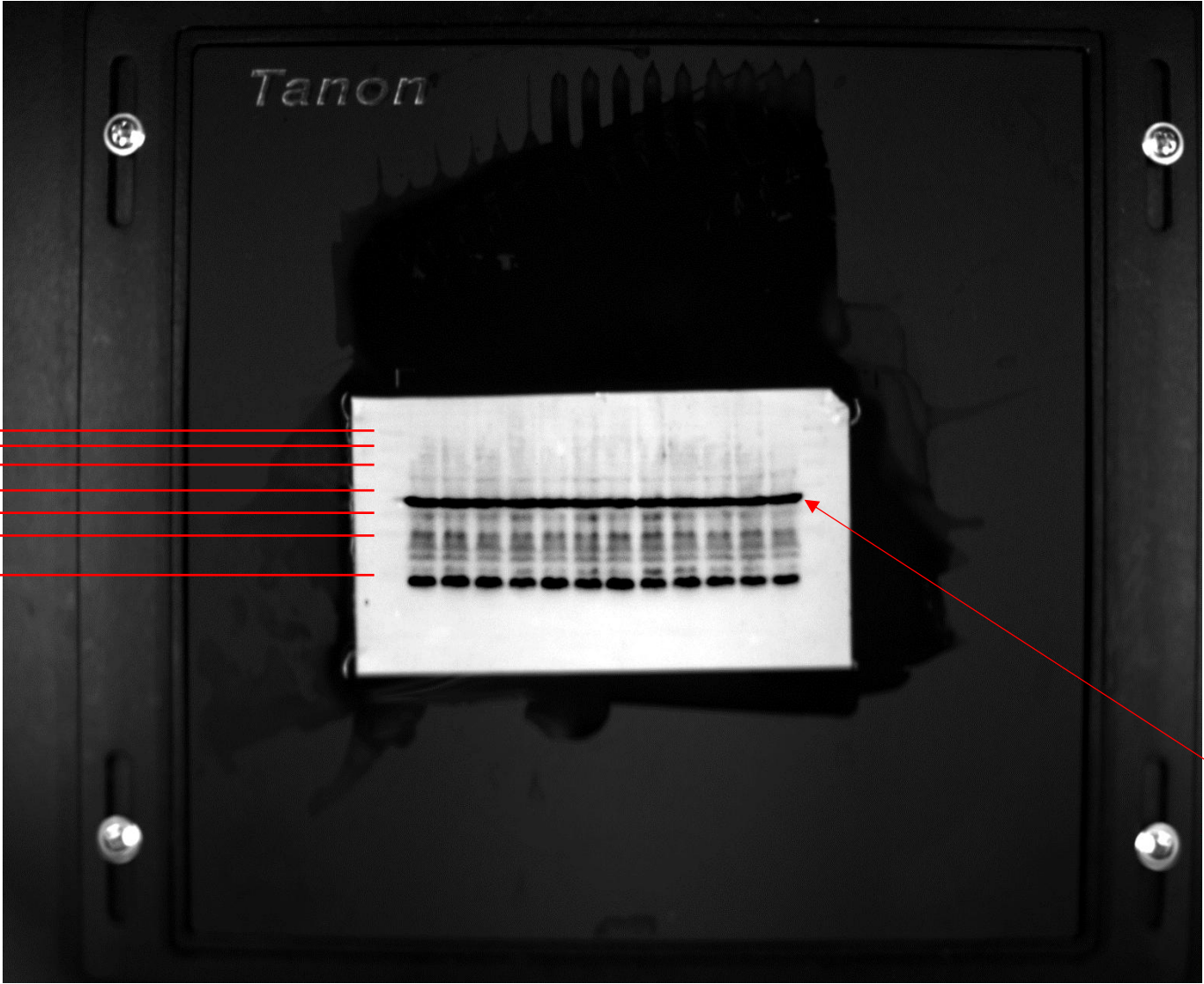

GAPDH

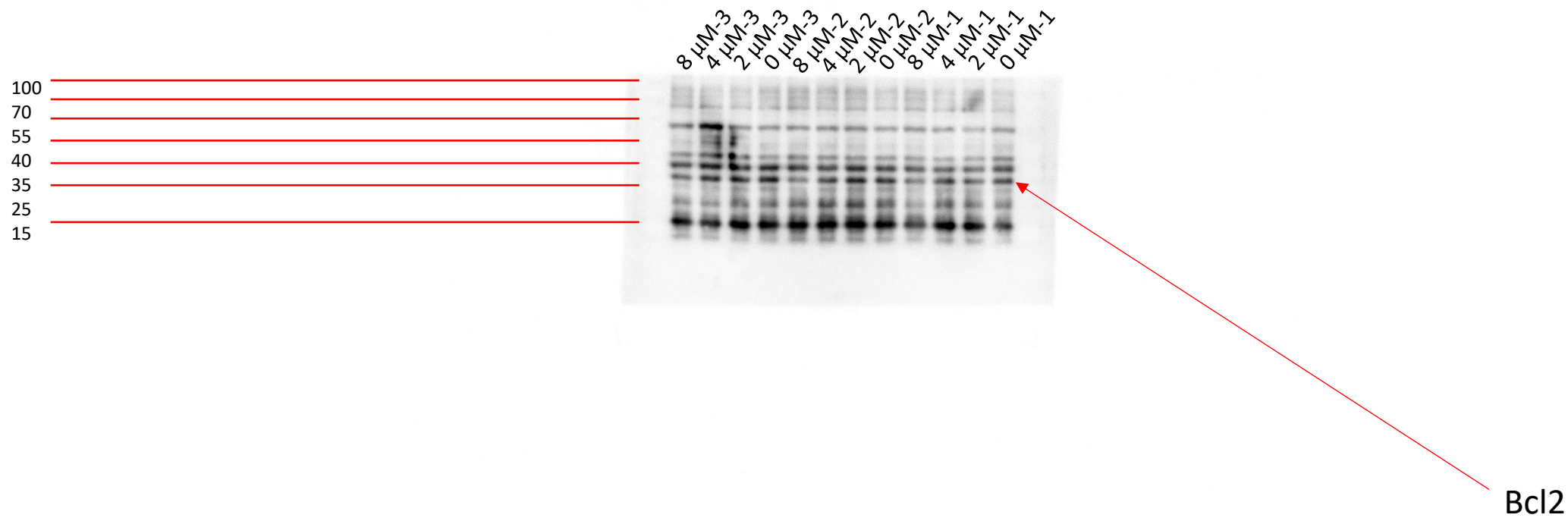

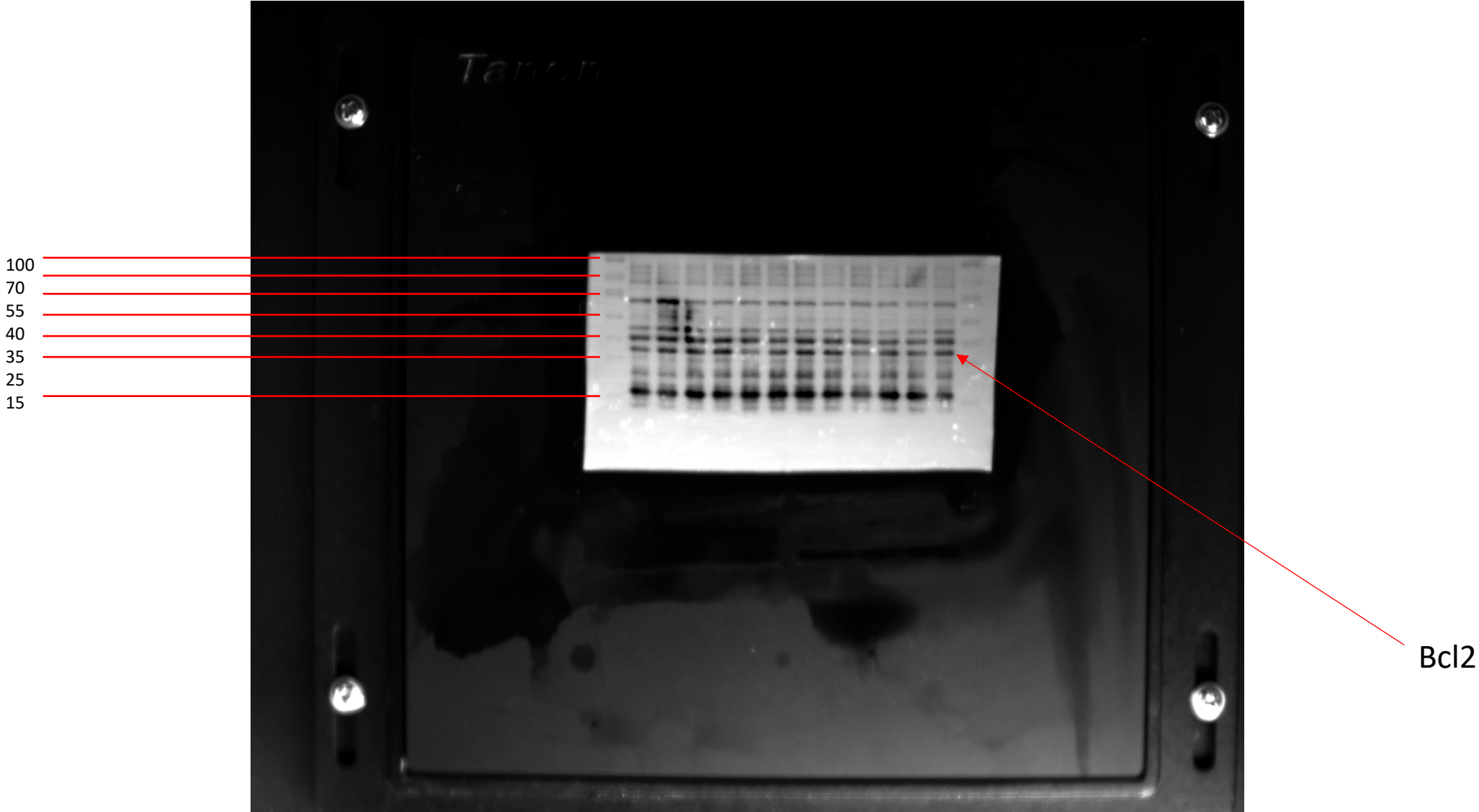

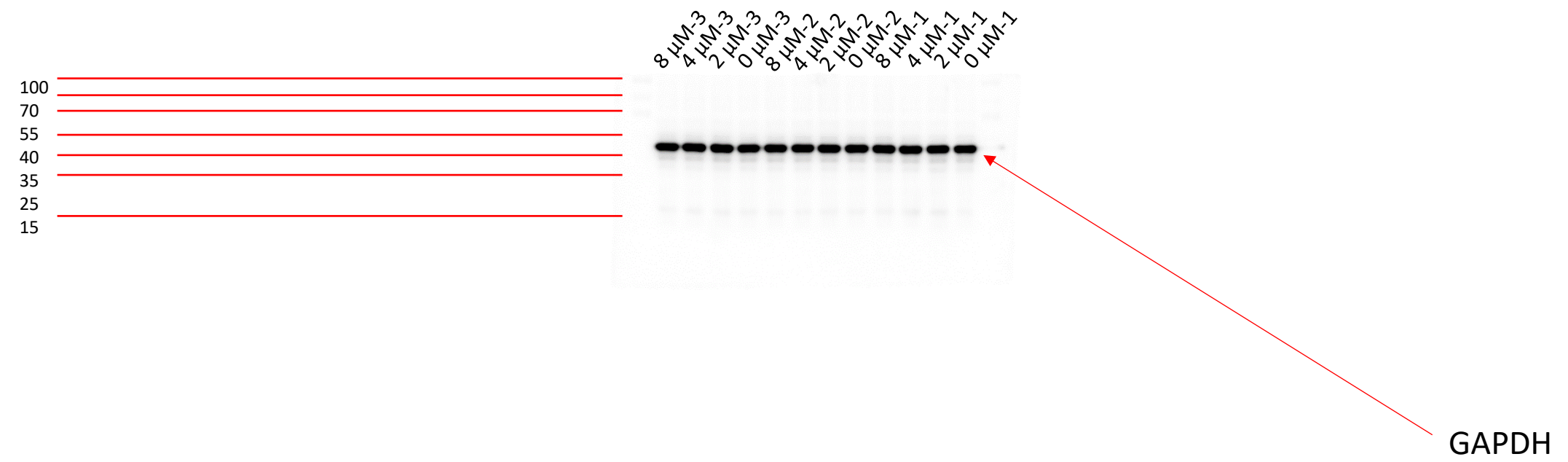

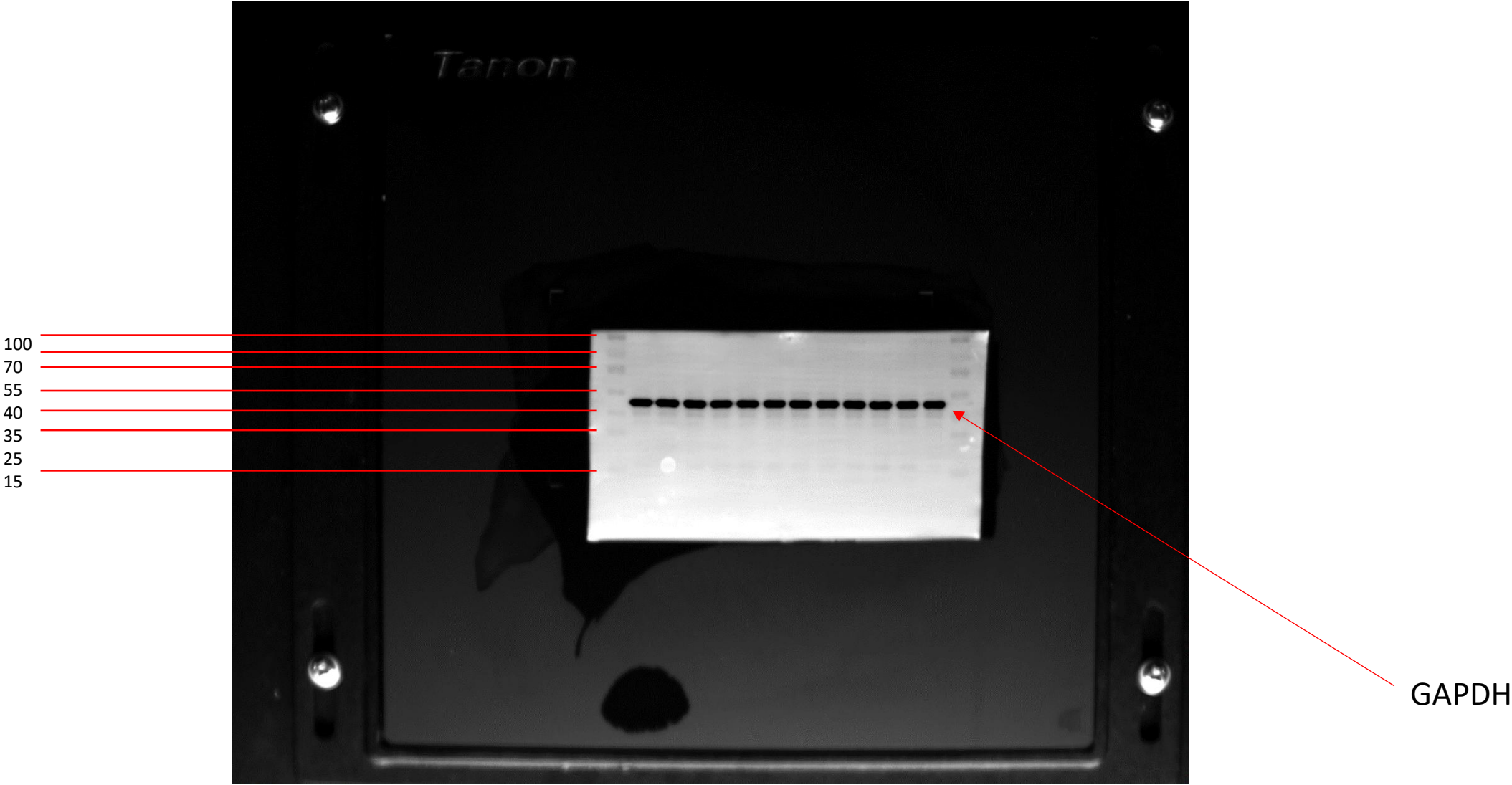

Supplement: Supplementary file 1 — Supplementary Material 1 [file 41598_2025_18875_MOESM1_ESM.pdf]
