# Supplementary material for: Investigating C7 modified tetrandrine derivatives for synthesis anti-hepatocellular carcinoma activity and mechanistic insights
Source: Sci Rep. 2025 Sep 29;15:33601. doi: 10.1038/s41598-025-18875-1 (PMC12480866; doi:10.1038/s41598-025-18875-1)
Supplement: Supplementary file 2 — Supplementary Material 2 [file 41598_2025_18875_MOESM2_ESM.docx]

**Supplementary Material**

Investigating C7 Modified Tetrandrine Derivatives for Synthesis Anti-Hepatocellular Carcinoma Activity and Mechanistic Insights

**Taibai Jiang ^1,‡^, Lihong Shi ^1‡^, Xueke Peng ^1^,Shan Zheng ^1^, Qian Chen ^4^, Junjie Lan ^3,*^ and Weidong Pan ^2,*^**

**1.Guizhou Province Engineering Research Center of Medical Resourceful Healthcare Products, College of Pharmacy, Guiyang Healthcare Vocational University, Guiyang 550081, PR China**

**2.School of Pharmaceutical Sciences, Guizhou University, Guiyang 550025, PR China**

**3.Department of Pharmacy, Guizhou Provincial People’s Hospital, Guiyang 550002, PR China**

**4.Eugenics Research Center, The First Affiliated Hospital of Guizhou University of Traditional Chinese Medicine, 550002, PR China**

*** Correspondence: wdpan@163.com (W.P.); lanjunjie2007@163.com (J.L.).**

**‡ These authors contributed equally to this work.**

Contents

[Characterization information of C_7_-*O*-Sulfonyl-Tetrandrine Derivatives 1-38 3](#_Toc193894058)

[NMR and MS Spectra 16](#_Toc193894059)

# Characterization information of C_7_-*O*-Sulfonyl-Tetrandrine Derivatives 1-38

Compound **1** 7-*O*-(3,5-Dichlorobenzenesulfonyl)-tetrandrine

Beige solid, yield: 92.8%; m.p.: 119.2-121.2 ℃; ^1^H NMR (600 MHz, CDCl_3_) *δ* (ppm): 7.64 (t, *J* = 1.9 Hz, 1H), 7.59 (d, *J* = 1.9 Hz, 2H), 7.35 (dd, *J* = 8.2, 2.2 Hz, 1H), 7.19 (dd, *J* = 8.2, 2.6 Hz, 1H), 6.88-6.85 (m, 2H), 6.74 (dd, *J* = 8.2, 2.6 Hz, 1H), 6.48 (d, *J* = 1.6 Hz, 1H), 6.47 (s, 1H), 6.35 (s, 1H), 6.25 (dd, *J* = 8.2, 2.2 Hz, 1H), 5.11 (s, 1H), 3.93 (s, 3H), 3.78 (dd, *J* = 11.3, 5.4 Hz, 1H), 3.66 (d, *J* = 10.0 Hz, 1H), 3.58 (s, 3H), 3.56-3.50 (m, 1H), 3.48-3.43 (m, 1H), 3.29 (s, 3H), 3.26 (dd, *J* = 12.4, 5.4 Hz, 1H), 2.98-2.87 (m, 4H), 2.80-2.72 (m, 2H), 2.68-2.64 (m, 4H), 2.54 (d, *J* = 13.6 Hz, 1H), 2.49-2.45 (m, 1H), 2.29 (s, 3H). ^13C NMR^ (150 MHz, CDCl_3_) *δ* (ppm): 153.9, 150.8, 149.2, 148.6, 147.6, 147.2, 142.3, 140.3, 135.5, 134.9, 134.4, 133.5, 133.0, 132.6, 130.1, 128.6, 128.4, 128.0, 126.9, 124.2, 122.8, 121.9, 121.7, 120.5, 116.5, 112.9, 111.5, 106.3, 63.7, 61.4, 56.1, 55.8, 55.8, 44.9, 43.8, 42.3, 42.3, 42.1, 39.2, 24.4, 22.1. HR-ESIMS(*m/z*) : 817.2112 [M + H]^+,^ calculated for C_43_H_43_Cl_2_N_2_O_8_S: 817.2112.

Compound **2** 7-*O*-Trifluoromathanesulfonyl- tetrandrine

Beige solid, yield: 89.9%; m.p.: 205.1-207.2 ℃; ^1^H NMR (600 MHz, CDCl_3_) *δ* (ppm): 7.40 (dd, *J* = 8.2, 2.2 Hz, 1H), 7.17 (dd, *J* = 8.2, 2.6 Hz, 1H), 6.87-6.83 (m, 2H), 6.81 (dd, *J* = 8.2, 2.6 Hz, 1H), 6.53 (s, 1H), 6.49 (d, *J* = 1.7 Hz, 1H), 6.40 (s, 1H), 6.35 (dd, *J* = 8.2, 2.2 Hz, 1H), 5.99 (s, 1H), 3.93 (s, 3Hz), 3.82 (dd, *J* = 11.3, 5.4 Hz, 1H), 3.78 (s, 3H), 3.69 (d, *J* = 10.0 Hz, 1H), 3.57-3.48 (m, 2H), 3.36 (s, 3H), 3.31 (dd, *J* = 12.4, 5.3 Hz, 1H), 2.99-2.87 (m, 4H), 2.82 (t, *J* = 11.9 Hz, 1H), 2.72-2.66 (m, 2H), 2.58 (s, 3H), 2.55 (d, *J* = 13.1 Hz, 1H), 2.47 (dd, *J* = 15.9, 4.8 Hz, 1H), 2.29 (s, 3H). ^13C NMR^ (150 MHz, CDCl_3_) *δ* (ppm): 153.8, 149.5, 149.4, 148.0, 147.7, 147.1, 141.7, 135.0, 134.3, 133.8, 132.5, 130.4, 129.1, 128.4, 128.2, 124.0, 122.8, 122.2, 121.9, 121.6, 118.4 (q, ^1^*J*_C-F_ = 319.3 Hz), 115.8 , 112.7, 111.5, 106.1, 64.3, 61.4, 56.1, 56.0, 55.8, 45.1, 43.7, 42.3, 41.6, 40.8, 23.9, 22.1. HR-ESIMS(*m/z*) : 741.2449 [M + H]^+^, calculated for C_38_H_40_F_3_N_2_O_8_S: 741.2452.

Compound **3** 7-*O*-(2,4,6-Trimethylbenzenesulfonyl)- tetrandrine

Beige solid, yield: 81.8%; m.p.: 144.8-145.0 ℃; ^1^H NMR (600 MHz, CDCl_3_) *δ* (ppm): 7.30 (dd, *J* = 8.2, 2.2 Hz, 1H), 7.14 (dd, *J* = 8.2, 2.5 Hz, 1H), 6.89 (s, 2H), 6.86 (s, 2H), 6.76 (dd, *J* = 8.2, 2.5 Hz, 1H), 6.50 (s, 1H), 6.48 (s, 1H), 6.29-6.27 (m, 2H), 5.50 (s, 1H), 3.93 (s, 3H), 3.73 (dd, *J* = 11.2, 5.3 Hz, 1H), 3.68 (d, *J* = 10.1 Hz, 1H), 3.53-3.45 (m, 2H), 3.43 (s, 3H), 3.32 (s, 3H), 3.30-3.28 (m, 1H), 2.96-2.86 (m, 4H), 2.76-2.69 (m, 3H), 2.68 (s, 3H), 2.54 (d, *J* = 13.8 Hz, 1H), 2.47-2.43 (m, 1H), 2.35 (s, 3H), 2.29 (s, 6H), 2.28 (s, 3H). ^13C NMR^ (150 MHz, CDCl_3_) *δ* (ppm): 153.8, 150.9, 149.4, 148.8, 148.0, 147.1, 142.8, 142.3, 139.8, 134.8, 134.6, 133.7, 132.5, 131.9, 131.2, 130.1, 128.6, 128.1, 127.8, 123.6, 122.8, 121.8, 121.8, 121.1, 116.0, 112.6, 111.6, 105.8, 64.0, 61.4, 56.1, 55.9, 55.5, 45.5, 43.9, 42.6, 42.4, 41.7, 39.3, 24.9, 22.7, 22.2, 21.2. HR-ESIMS(*m/z*) : 791.3361 [M + H]^+^, calculated for C_46_H_51_N_2_O_8_S: 791.3361.

Compound **4** 7-*O*-(2,4,6-Triisopropylbenzenesulfonyl)-tetrandrine

Light brown solid, yield: 72.8%; m.p.: 140.0-141.4 ℃; ^1^H NMR (600 MHz, CDCl_3_) *δ* (ppm): 7.38 (dd, *J* = 8.2, 2.2 Hz, 1H), 7.15 (dd, *J* = 8.2, 2.6 Hz, 1H), 7.09 (s, 2H), 6.89-6.85 (m, 2H), 6.81 (dd, *J* = 8.2, 2.6 Hz, 1H), 6.54-6.53 (m, 2H), 6.34 (dd, *J* = 8.2, 2.2 Hz, 1H), 6.15 (s, 1H), 6.07 (s, 1H), 3.93 (s, 3H), 3.91 (dd, *J* = 11.2, 5.6 Hz, 1H), 3.74 (d, *J* = 10.0 Hz, 1H), 3.64 (p, *J* = 6.7 Hz, 2H), 3.60-3.55 (m, 1H), 3.53-3.48 (m, 1H), 3.36 (s, 3H), 3.33 (dd, *J* = 12.4, 5.5 Hz, 1H), 3.07 (s, 3H), 3.02-2.77 (m, 7H), 2.72 (dd, *J* = 14.0, 10.1 Hz, 1H), 2.64-2.60 (m, 4H), 2.41 (dd, *J* = 15.6, 5.4 Hz, 1H), 2.31 (s, 3H), 1.24 (dd, *J* = 6.9, 4.5 Hz, 6H), 1.10 (d, *J* = 6.8 Hz, 6H), 1.06 (d, *J* = 6.7 Hz, 6H). ^13C NMR^ (150 MHz, CDCl_3_) *δ* (ppm): 153.8, 152.5, 150.3, 149.4, 149.2, 149.2, 148.4, 147.0, 142.9, 134.8, 134.7, 133.8, 132.5, 131.8, 130.5, 128.5, 127.9, 127.4, 123.0, 122.9, 122.8, 122.4, 122.1, 121.9, 116.1, 112.0, 111.5, 105.1, 64.0, 61.4, 56.1, 55.8, 54.9, 45.3, 43.8, 42.3, 42.2, 41.5, 40.0, 34.2, 29.7, 24.7, 24.3, 24.2, 23.7, 23.7, 22.1. HR-ESIMS(*m/z*) : 875.4298 [M + H]^+^, calculated for C_52_H_63_N_2_O_8_S: 875.4210.

PJ-0512 Compound **5** 7-*O*-(2-(Trifluoromethyl)benzenesulfonyl)-tetrandrine

Light brown solid, yield: 92.0%; m.p.: 135.9-136.1 ℃; ^1^H NMR (600 MHz, CDCl_3_) *δ* (ppm): 7.91 (d, *J* = 7.8 Hz, 1H), 7.85 (d, *J* = 7.8 Hz, 1H), 7.69 (t, *J* = 7.6 Hz, 1H), 7.60 (t, *J* = 7.6 Hz, 1H), 7.36 (dd, *J* = 8.2, 2.2 Hz, 1H), 7.15 (dd, *J* = 8.2, 2.6 Hz, 1H), 6.86 (s, 2H), 6.79 (dd, *J* = 8.2, 2.6 Hz, 1H), 6.53 (s, 1H), 6.50 (s, 1H), 6.33 (dd, *J* = 8.2, 2.2 Hz, 1H), 6.20 (s, 1H), 5.78 (s, 1H), 3.93 (s, 3H), 3.77 (dd, *J* = 11.3, 5.3 Hz, 1H), 3.71 (d, *J* = 9.9 Hz, 1H), 3.54-3.47 (m, 2H), 3.35 (s, 3H), 3.30 (dd, *J* = 12.4, 5.3 Hz, 1H), 3.22 (s, 3H), 3.01-2.88 (m, 4H), 2.78 (t, *J* = 11.9 Hz, 1H), 2.74-2.68 (m, 2H), 2.63 (s, 3H), 2.59 (d, *J* = 13.1 Hz, 1H), 2.44-2.39 (m, 1H), 2.28 (s, 3H). ^13C NMR^ (150 MHz, CDCl_3_) *δ* (ppm): 153.7, 150.1, 149.43\, 148.6, 148.0, 147.1, 142.3, 136.6, 135.1, 134.6, 133.0, 132.6, 132.5, 131.6, 131.6, 130.3, 128.7 (q, 2JC-F = 33.7 Hz), 128.6, 128.3, 128.0, 127.9 (q, 3JC-F = 6.0 Hz), 123.6, 122.8, 122.2 (q, 1JC-F = 272.7 Hz), 122.0, 121.9, 121.3, 116.0, 112.7, 111.5, 105.5, 64.1, 61.3, 56.1, 55.8, 55.2, 45.3, 43.8, 42.4, 42.3, 41.6, 40.1, 24.4, 22.2. HR-ESIMS(*m/z*) : 817.2768 [M + H]^+^, calculated for C_44_H_44_F_3_N_2_O_8_S: 817.2765.

Compound **6** 7-*O*-(4-(Trifluoromethoxy)benzenesulfonyl)-tetrandrine

Light brown solid, yield: 92.9%; m.p.: 93.3-95.2 ℃; ^1^H NMR (600 MHz, CDCl_3_) *δ* (ppm): 7.72 (d, *J* = 8.9 Hz, 2H), 7.36 (dd, *J* = 8.2, 2.2 Hz, 1H), 7.29 (d, *J* = 8.4 Hz, 2H), 7.16 (dd, *J* = 8.2, 2.6 Hz, 1H), 6.87 (d, *J* = 1.4 Hz, 2H), 6.79 (dd, *J* = 8.2, 2.6 Hz, 1H), 6.52 (s, 1H), 6.50 (s, 1H), 6.30 (dd, *J* = 8.2, 2.2 Hz, 1H), 6.23 (s, 1H), 5.66 (s, 1H), 3.93 (s, 3H), 3.82 (dd, *J* = 11.2, 5.2 Hz, 1H), 3.71 (d, *J* = 10.1 Hz, 1H), 3.54-3.44 (m, 2H), 3.34-3.32 (m, 6H), 3.30 (dd, *J* = 12.3, 5.3 Hz, 1H), 3.02-2.89 (m, 4H), 2.79-2.72 (m, 3H), 2.67 (s, 3H), 2.59 (d, *J* = 13.5 Hz, 1H), 2.47-2.43 (m, 1H), 2.28 (s, 3H). ^13^C NMR (150 MHz, CDCl_3_) *δ* (ppm): 153.7, 152.7, 150.4, 149.4, 149.0, 148.1, 147.1, 142.3, 135.6, 134.9, 134.5, 132.8, 132.5, 130.7, 130.3, 128.3, 127.9, 127.6, 123.7, 122.8, 122.0, 121.9, 121.2, 120.4, 120.2 (q, ^1^*J*_C-F_ = 258.2 Hz), 115.9, 112.6, 111.5, 105.7, 105.7, 63.9, 61.4, 56.1, 55.8, 55.3, 45.0, 43.8, 42.3, 42.3, 41.7, 38.5, 38.4, 24.7, 24.71, 22.2. HR-ESIMS(*m/z*) : 833.2718 [M + H]^+^, calculated for C_44_H_44_F_3_N_2_O_9_S: 833.2714.

Compound **7** 7-*O*-(1-Butanesulfonyl)-tetrandrine

Beige solid, yield: 54.7%; m.p.: 106.5-108.7 ℃; ^1^H NMR (600 MHz, CDCl_3_) *δ* (ppm): 7.37 (dd, *J* = 8.2, 2.2 Hz, 1H), 7.15 (dd, *J* = 8.2, 2.6 Hz, 1H), 6.86-6.80 (m, 2H), 6.81 (dd, *J* = 8.2, 2.6 Hz, 1H), 6.54 (s, 1H), 6.49 (s, 1H), 6.38 (s, 1H), 6.34 (dd, *J* = 8.2, 2.2 Hz, 1H), 6.04 (s, 1H), 3.93 (s, 3H), 3.87 (dd, *J* = 11.3, 5.3 Hz, 1H), 3.78 (s, 3H), 3.71 (d, *J* = 9.8 Hz, 1H), 3.53-3.47 (m, 2H), 3.37 (s, 3H), 3.32 (dd, *J* = 12.4, 5.3 Hz, 1H), 3.01-2.89 (m, 6H), 2.80 (t, *J* = 11.9 Hz, 1H), 2.77-2.73 (m, 1H), 2.69 (dd, *J* = 14.0, 10.4 Hz, 1H), 2.63 (s, 3H), 2.58 (d, *J* = 13.0 Hz, 1H), 2.48-2.43 (m, 1H), 2.29 (s, 3H), 1.79-1.66 (m, 2H), 1.37 (h, *J* = 7.5 Hz, 2H), 0.90 (t, *J* = 7.4 Hz, 3H). ^13^C NMR (150 MHz, CDCl_3_) *δ* (ppm): 153.7, 150.4, 149.4, 148.9, 148.3, 147.0, 142.3, 134.8, 134.5, 132.5, 132.4, 130.4, 128.2, 127.9, 127.8, 123.7, 122.8, 122.2, 122.0, 121.7, 115.9, 112.6, 111.5, 106.1, 64.1, 61.4, 56.1, 56.1, 55.9, 51.7, 45.1, 43.8, 42.4, 42.3, 41.5, 39.0, 25.3, 24.6, 22.2, 21.5, 13.5. HR-ESIMS(*m/z*) : 729.3203 [M + H]^+^, calculated for C_41_H_49_N_2_O_8_S: 729.3204.

Compound **8** 7-*O*-(4-tert-Butylbenzenesulfonyl)-tetrandrine

Beige solid, yield: 82.3%; m.p.: 141.9-143.1 ℃; ^1^H NMR (600 MHz, CDCl_3_) *δ* (ppm): 7.57 (dt, *J* = 8.6, 2.2 Hz, 2H), 7.43 (dt, *J* = 8.6, 2.2 Hz, 2H), 7.36 (dd, *J* = 8.2, 2.2 Hz, 1H), 7.13 (dd, *J* = 8.2, 2.6 Hz, 1H), 6.86 (s, 2H), 6.79 (dd, *J* = 8.2, 2.6 Hz, 1H), 6.51 (s, 1H), 6.50 (s, 1H), 6.28 (dd, *J* = 8.2, 2.2 Hz, 1H), 6.20 (s, 1H), 5.82 (s, 1H), 3.93 (s, 3H), 3.83 (dd, *J* = 11.2, 5.4 Hz, 1H), 3.68 (d, *J* = 9.9 Hz, 1H), 3.55-3.48 (m, 2H), 3.32 (s, 3H), 3.30-3.28 (m, 1H), 3.27 (s, 3H), 3.00-2.89 (m, 4H), 2.81 (t, *J* = 11.8 Hz, 1H), 2.72-2.69 (m, 2H), 2.66 (s, 3H), 2.59 (d, *J* = 13.0 Hz, 1H), 2.45-2.41 (m, 1H), 2.28 (s, 3H), 1.32 (s, 9H). ^13^C NMR (150 MHz, CDCl_3_) *δ* (ppm): 157.3, 153.6, 150.6, 149.3, 149.3, 148.1, 147.0, 142.5, 135.0, 134.6, 134.1, 132.5, 132.4, 130.4, 128.4, 128.3, 127.8, 127.7, 125.4, 123.5, 122.8, 122.0, 121.8, 121.8, 115.9, 112.6, 111.4, 105.5, 64.2, 61.5, 56.1, 55.8, 55.3, 45.2, 43.8, 42.5, 42.3, 41.6, 39.9, 35.2, 31.1, 24.3, 22.1. HR-ESIMS(*m/z*) : 805.3519 [M + H]^+^, calculated for C_47_H_53_N_2_O_8_S: 805.3517.

Compound **9** 7-*O*-(3-Nitrobenzenesulfonyl)-tetrandrine

Orange solid, yield: 70.4%; m.p.: 120.5-121.6 ℃; ^1^H NMR (600 MHz, CDCl_3_) *δ* (ppm): 8.58 (t, *J* = 2.0 Hz, 1H), 8.47 (ddd, *J* = 8.2, 2.2, 1.1 Hz, 1H), 7.97 (d, *J* = 7.9 Hz, 1H), 7.66 (t, *J* = 8.0 Hz, 1H), 7.33 (dd, *J* = 8.2, 2.2 Hz, 1H), 7.17 (dd, *J* = 8.2, 2.6 Hz, 1H), 6.87 (s, 2H), 6.76 (dd, *J* = 8.2, 2.6 Hz, 1H), 6.50 (s, 1H), 6.43 (s, 1H), 6.30 (s, 1H), 6.27 (dd, *J* = 8.2, 2.2 Hz, 1H), 5.44 (s, 1H), 3.93 (s, 3H), 3.70-3.66 (m, 2H), 3.55-3.50 (m, 1H), 3.44 (s, 4H), 3.32 (s, 3H), 3.26 (dd, *J* = 12.4, 5.3 Hz, 1H), 2.97-2.87 (m, 4H), 2.77-2.67 (m, 3H), 2.64 (s, 3H), 2.53 (d, *J* = 13.6 Hz, 1H), 2.48-2.44 (m, 1H), 2.28 (s, 3H). ^13^C NMR (150 MHz, CDCl_3_) *δ* (ppm): 153.8, 150.3, 149.4, 148.7, 147.9, 147.8, 147.1, 142.1, 139.4, 134.9, 134.4, 133.9, 133.1, 132.5, 130.2, 129.8, 128.6, 128.1, 127.8, 124.1, 123.7, 122.8, 122.0, 121.8, 120.7, 116.0, 112.8, 111.6, 106.1, 64.0, 61.4, 56.1, 55.8, 55.6, 45.1, 43.8, 42.4, 42.3, 41.9, 38.8, 24.7, 22.2. HR-ESIMS(*m/z*) : 794.2747 [M + H]^+^, calculated for C_43_H_44_N_3_O_10_S: 794.2742.

Compound **10** 7-*O*-(3-(Trifluoromethyl)benzenesulfonyl)-tetrandrine

Beige solid, yield: 94.6%; m.p.: 131.7-132.8 ℃; ^1^H NMR (600 MHz, CDCl_3_) *δ* (ppm): 8.01 (s, 1H), 7.86 (dd, *J* = 12.9, 7.9 Hz, 2H), 7.60 (t, *J* = 7.9 Hz, 1H), 7.34 (dd, *J* = 8.2, 2.2 Hz, 1H), 7.16 (dd, *J* = 8.2, 2.6 Hz, 1H), 6.87 (s, 2H), 6.77 (dd, *J* = 8.2, 2.6 Hz, 1H), 6.51 (s, 1H), 6.49 (s, 1H), 6.29 (dd, *J* = 8.2, 2.2 Hz, 1H), 6.24 (s, 1H), 5.59 (s, 1H), 3.93 (s, 3H), 3.78 (dd, *J* = 11.2, 5.4 Hz, 1H), 3.69 (d, *J* = 10.0 Hz, 1H), 3.55-3.46 (m, 2H), 3.32 (d, *J* = 1.3 Hz, 6H), 3.27 (dd, *J* = 12.4, 5.4 Hz, 1H), 3.00-2.89 (m, 4H), 2.80-2.68 (m, 3H), 2.65 (s, 3H), 2.58 (d, *J* = 13.2 Hz, 1H), 2.46-2.42 (m, 1H), 2.28 (s, 3H). ^13^C NMR (150 MHz, CDCl_3_) *δ* (ppm): 153.8, 150.3, 149.4, 149.0, 148.0, 147.1, 142.3, 138.7, 135.0, 134.5, 132.9, 132.5, 131.8, 131.3 (q, ^2^*J*_C-F_ = 33.4 Hz), 130.3, 129.9 (q, ^3^*J*_C-F_ = 3.6 Hz), 129.3, 128.5, 128.0, 127.9, 125.5 (q, ^3^*J*_C-F_ = 3.6 Hz), 123.9, 123.1 (q, ^1^*J*_C-F_ = 271.4 Hz), 122.8, 122.0, 121.8, 121.3, 116.0, 112.7, 111.5, 105.8, 63.9, 61.4, 56.1, 55.8, 55.3, 45.1, 43.8, 42.4, 42.3, 41.8, 39.3, 24.5, 22.1. HR-ESIMS(*m/z*) : 817.2768 [M + H]^+^, calculated for C_44_H_44_F_3_N_2_O_8_S: 817.2765.

Compound **11** 7-*O*-(2-Fluorobenzenesulfonyl)-tetrandrine

Beige solid, yield: 94.4%; m.p.: 156.7-157.8 ℃; ^1^H NMR (600 MHz, CDCl_3_) *δ* (ppm): 7.67-7.60 (m, 2H), 7.33 (dd, *J* = 8.2, 2.2 Hz, 1H), 7.22 (td, *J* = 7.6, 1.1 Hz, 1H), 7.19-7.14 (m, 2H), 6.89-6.86 (m, 2H), 6.77 (dd, *J* = 8.3, 2.6 Hz, 1H), 6.51 (s, 1H), 6.50 (d, *J* =1.1 Hz, 1H), 6.28 (dd, *J* = 8.3, 2.2 Hz, 1H), 6.25 (s, 1H), 5.43 (s, 1H), 3.93 (s, 3H), 3.72-3.69 (m, 2H), 3.53-3.47 (m, 2H), 3.39 (s, 3H), 3.33 (s, 3H), 3.28 (dd, *J* = 12.4, 5.3 Hz, 1H), 2.99-2.94 (m, 1H), 2.92-2.89 (m, 3H), 2.78-2.67 (m, 3H), 2.63 (s, 3H), 2.59 (d, *J* = 13.3 Hz, 1H), 2.45-2.41 (m, 1H), 2.29 (s, 3H). ^13^C NMR (150 MHz, CDCl_3_) *δ* (ppm): 159.7 (q, ^1^*J*_C-F_ = 259.2 Hz), 153.9, 150.5, 149.3, 148.9, 148.0, 147.1, 142.4, 135.7 (q, ^3^*J*_C-F_ = 8.2 Hz), 134.8, 134.7, 132.6, 132.5, 131.1, 130.3, 128.3, 128.3, 127.5, 126.0 (q, ^3^*J*_C-F_ = 318.4 Hz), 123.7, 123.7, 122.9, 121.8, 121.7, 121.1, 117.0 (q, ^2^*J*_C-F_ = 13.7 Hz), 116.1, 112.8, 111.6, 105.8, 64.0, 61.4, 56.1, 55.8, 55.5, 45.0, 43.8, 42.3, 41.7, 39.7, 39.7, 24.1, 22.1. HR-ESIMS(*m/z*) : 767.2800 [M + H]^+^, calculated for C_43_H_44_FN_2_O_8_S: 767.2797.

Compound **12** 7-*O*-(4-Fluorobenzenesulfonyl)-tetrandrine

Beige solid, yield: 95.0%; m.p.: 128.8-130.8 ℃; ^1^H NMR (600 MHz, CDCl_3_) *δ* (ppm): 7.69-7.67 (m, 2H), 7.35 (dd, *J* = 8.2, 2.2 Hz, 1H), 7.18 (dd, *J* = 8.2, 2.6 Hz, 1H), 7.17-7.13 (m, 2H), 6.88-6.86 (m, 2H), 6.76 (dd, *J* = 8.2, 2.6 Hz, 1H), 6.47 (s, 1H), 6.46 (s, 1H), 6.30 (s, 1H), 6.28 (dd, *J* = 8.3, 2.2 Hz, 1H), 5.25 (s, 1H), 3.93 (s, 3H), 3.71 (dd, *J* = 11.3, 5.3 Hz, 1H), 3.67 (d, *J* = 10.0 Hz, 1H), 3.54-3.49 (m, 4H), 3.46-3.41 (m, 1H), 3.29 (s, 3H), 3.26 (dd, *J* = 12.3, 5.3 Hz, 1H), 2.97-2.87 (m, 4H), 2.77-2.71 (m, 2H), 2.69-2.66 (m, 1H), 2.65 (s, 3H), 2.54 (d, *J* = 13.2 Hz, 1H), 2.47-2.43 (m, 1H), 2.28 (s, 3H). ^13^C NMR (150 MHz, CDCl_3_) *δ* (ppm): 165.7 (q, ^1^*J*_C-F_ = 254.3 Hz), 153.8, 150.9, 149.4, 148.7, 147.8, 147.2, 142.3, 135.0, 134.5, 133.5 (q, ^4^*J*_C-F_ = 3.0 Hz), 132.6, 132.6, 131.4 (q, ^3^*J*_C-F_ = 9.4 Hz), 130.2, 128.3, 128.1, 127.9, 123.9, 122.8, 121.9, 121.8, 120.7, 116.0, 115.9 (q, ^2^*J*_C-F_ = 22.3 Hz), 112.8, 111.6, 106.1, 63.9, 61.3, 56.1, 55.8, 55.7, 45.1, 43.9, 42.4, 42.3, 42.1, 38.9, 24.6, 22.2. HR-ESIMS(*m/z*) : 767.2796 [M + H]^+^, calculated for C_43_H_44_FN_2_O_8_S: 767.2797.

Compound **13** 7-*O*-(3-Fluorobenzenesulfonyl)-tetrandrine

Beige solid, yield: 89.2%; m.p.: 109.6-110.6 ℃; ^1^H NMR (600 MHz, CDCl_3_) *δ* (ppm): 7.46-7.31(m, 3H), 7.36-7.33 (m, 2H), 7.17 (dd, *J* = 8.2, 2.6 Hz, 1H), 6.89-6.86 (m, 2H), 6.75 (dd, *J* = 8.2, 2.6 Hz, 1H), 6.48 (s, 1H), 6.45 (s, 1H), 6.30 (s, 1H), 6.28 (dd, *J* = 8.3, 2.2 Hz, 1H), 5.32 (s, 1H), 3.93 (s, 3H), 3.74 (dd, *J* = 11.3, 5.3 Hz, 2H), 3.66 (d, *J* = 10.1 Hz, 1H), 3.54-3.48 (m, 1H), 3.47 (s, 4H), 3.30 (s, 3H), 3.27 (dd, *J* = 12.4, 5.3 Hz, 1H), 2.98-2.88 (m, 4H), 2.77-2.73 (m, 2H), 2.69-2.65 (m, 1H), 2.64 (s, 3H), 2.53 (d, *J* = 13.3 Hz, 1H), 2.47-2.43 (m, 1H), 2.28 (s, 3H). ^13^C NMR (150 MHz, CDCl_3_) *δ* (ppm): 161.94 (q, ^1^*J*_C-F_ = 249.7 Hz), 153.8, 150.8, 149.4, 148.8, 147.8, 147.2, 142.3, 139.2 (q, ^3^*J*_C-F_ = 7.3 Hz), 135.0, 134.5, 132.7, 132.6, 130.3, 130.2, 128.5, 128.1, 127.9, 124.4 (q, ^4^*J*_C-F_ = 3.2 Hz), 123.9, 122.8, 121.8, 121.7, 121.0, 120.8 (q, ^2^*J*_C-F_ = 20.9 Hz), 116.1, 116.0 (q, ^2^*J*_C-F_ = 24.5 Hz), 112.8, 111.6, 106.1, 63.9, 61.4, 56.1, 55.8, 55.7, 45.1, 43.9, 42.4, 42.3, 42.0, 39.6, 24.4, 22.1. HR-ESIMS(*m/z*) : 767.2802 [M + H]^+^, calculated for C_43_H_44_FN_2_O_8_S: 767.2797.

Compound **14** 7-*O*-Benzenesulfonyl-tetrandrine

Beige solid, yield: 94.9%; m.p.: 153.0-155.1 ℃; ^1^H NMR (600 MHz, CDCl_3_) *δ* (ppm): 7.68-7.66 (m, 2H), 7.65-7.62 (m, 1H), 7.48-7.45 (m, 2H), 7.32 (dd, *J* = 8.2, 2.2 Hz, 1H), 7.16 (dd, *J* = 8.2, 2.6 Hz, 1H), 6.89-6.86 (m, 2H), 6.74 (dd, *J* = 8.2, 2.6 Hz, 1H), 6.47-6.46 (m, 2H), 6.28 (s, 1H), 6.26 (dd, *J* = 8.2, 2.2 Hz, 1H), 5.22 (s, 1H), 3.93 (s, 3H), 3.70 (dd, *J* = 11.3, 5.3 Hz, 1H), 3.67 (d, *J* = 10.0 Hz, 1H), 3.54-3.46 (m, 2H), 3.44 (s, 3H), 3.29 (s, 3H), 3.25 (dd, *J* = 12.4, 5.3 Hz, 1H), 2.96-2.87 (m, 4H), 2.77-2.72 (m, 2H), 2.67-2.66 (m, 1H), 2.64 (s, 3H), 2.55 (d, *J* = 13.3 Hz, 1H), 2.46-2.42 (m, 1H), 2.28 (s, 3H). ^13^C NMR (150 MHz, CDCl_3_) *δ* (ppm): 153.8, 151.0, 149.3, 148.9, 147.8, 147.1, 142.4, 137.4, 134.9, 134.6, 133.5, 132.6, 132.4, 130.2, 128.6, 128.5, 128.3, 128.2, 127.7, 123.8, 122.8, 121.7, 121.7, 121.1, 116.1, 112.7, 111.6, 106.0, 63.9, 61.4, 56.1, 55.8, 55.7, 45.1, 43.9, 42.4, 42.3, 42.0, 39.7, 24.3, 22.1. HR-ESIMS(*m/z*) : 749.2891 [M + H]^+^, calculated for C_43_H_45_N_2_O_8_S: 749.2891.

Compound **15** 7-*O*-(alpha-Toluenesulfonyl)-tetrandrine

Beige solid, yield: 81.1%; m.p.: 130.4-131.4 ℃; ^1^H NMR (600 MHz, CDCl_3_) *δ* (ppm): 7.35-7.27 (m, 6H), 7.12 (dd, *J* = 8.2, 2.6 Hz, 1H), 6.86-6.83 (m, 2H), 6.80 (dd, *J* = 8.2, 2.6 Hz, 1H), 6.52 (s, 1H), 6.49 (s, 1H), 6.42 (s, 1H), 6.33 (dd, *J* = 8.2, 2.2 Hz, 1H), 6.04 (s, 1H), 4.14 (q, *J* = 13.7 Hz, 2H), 3.92 (s, 3H), 3.82-3.79 (m, 4H), 3.72 (d, *J* = 9.9 Hz, 1H), 3.55-3.43 (m, 2H), 3.38 (s, 3H), 3.26 (dd, *J* = 12.6, 5.3 Hz, 1H), 3.00-2.89 (m, 3H), 2.85-2.81 (m, 1H), 2.78 (t, *J* = 11.9 Hz, 1H), 2.72-2.68 (m, 2H), 2.60 (d, *J* = 12.9 Hz, 1H), 2.50 (s, 3H), 2.49-2.46 (m, 1H), 2.30 (s, 3H). ^13^C NMR (1510 MHz, CDCl_3_) *δ* (ppm): 153.7, 150.4, 149.4, 148.9, 148.2, 147.0, 142.3, 134.9, 134.5, 132.6, 132.5, 130.8, 130.4, 128.8, 128.7, 128.5, 128.1, 127.7, 127.5, 123.8, 122.8, 122.2, 121.9, 121.7, 115.9, 112.5, 111.5, 106.3, 64.0, 61.4, 57.4, 56.1, 56.1, 55.8, 45.2, 43.8, 42.3, 42.3, 41.5, 39.6, 24.5, 22.2. HR-ESIMS(*m/z*) : 763.3049 [M + H]^+^, calculated for C_44_H_47_N_2_O_8_S: 763.3048.

Compound **16** 7-*O*-(4-Toluenesulfonyl)-tetrandrine

Beige solid, yield: 92.8%; m.p.: 142.8-144.1 ℃; ^1^H NMR (600 MHz, CDCl_3_) *δ* (ppm): 7.53 (d, *J* = 8.3 Hz, 2H), 7.31 (dd, *J* = 8.1, 2.2 Hz, 1H), 7.24 (d, *J* = 8.1 Hz, 2H), 7.15 (dd, *J* = 8.1, 2.6 Hz, 1H), 6.90-6.84 (m, 2H), 6.74 (dd, *J* = 8.3, 2.6 Hz, 1H), 6.46 (s, 1H), 6.44 (s, 1H), 6.31 (s, 1H)z, 6.24 (dd, *J* = 8.3, 2.2 Hz, 1H), 5.19 (s, 1H), 3.93 (s, 3H), 3.66-3.63 (m, 2H), 3.54-3.46 (m, 5H), 3.28 (s, 3H), 3.24 (dd, *J* = 12.4, 5.3 Hz, 1H), 2.96-2.86 (m, 4H), 2.77-2.71 (m, 2H), 2.66-2.63 (m, 4H), 2.53 (d, *J* = 13.1 Hz, 1H), 2.48 (s, 3H), 2.47-2.43 (m, 1H), 2.28 (s, 3H). ^13^C NMR (150 MHz, CDCl_3_) *δ* (ppm): 153.8, 151.2, 149.3, 148.8, 147.8, 147.1, 144.4, 142.5, 134.9, 134.6, 134.5, 132.6, 132.4, 130.1, 129.1, 128.7, 128.3, 128.2, 127.6, 123.7, 122.9, 121.6, 121.6, 121.0, 116.0, 112.8, 111.6, 106.1, 64.2, 61.4, 56.1, 55.8, 55.8, 45.2, 43.9, 42.5, 42.3, 41.9, 40.1, 24.2, 22.1, 21.8. HR-ESIMS(*m/z*) : 763.3051 [M + H]^+^, calculated for C_44_H_47_N_2_O_8_S: 763.3048.

Compound **17** 7-*O*-(4-Methoxybenzenesulfonyl)-tetrandrine

Beige solid, yield: 93.0%; m.p.: 130.2-132.4 ℃; H NMR (600 MHz, CDCl_3_) *δ* (ppm): 7.58 (dt, *J* = 8.9, 2.9 Hz, 2H), 7.31 (dd, *J* = 8.2, 2.2 Hz, 1H), 7.14 (dd, *J* = 8.2, 2.6 Hz, 1H), 6.92 (dt, *J* = 8.9, 2.9 Hz, 2H), 6.89-6.86 (m, 2H), 6.73 (dd, *J* = 8.2, 2.6 Hz, 1H), 6.45 (s, 1H), 6.43 (s, 1H), 6.32 (s, 1H), 6.25 (dd, *J* = 8.2, 2.2 Hz, 1H), 5.15 (s, 1H), 3), 3.93 (s, 3H), 3.89 (s, 3H),3.68 (dd, *J* = 11.3, 5.3 Hz, 1H), 3.65 (d, *J* = 10.1 Hz, 1H), 3.56 (s, 3H), 3.53-3.43 (m, 2H), 3.27 (s, 3H), 3.24 (dd, *J* = 12.3, 5.3 Hz, 1H), 2.97-2.86 (m, 4H), 2.76-2.71 (m, 2H), 2.66-2.63 (m, 4H), 2.53 (d, *J* = 13.4 Hz, 1H), 2.47-2.43 (m, 1H), 2.28 (s, 3H). ^13^C NMR (150 MHz, CDCl_3_) *δ* (ppm): 163.6, 153.7, 151.3, 149.3, 148.8, 147.7, 147.2, 142.5, 135.0, 134.6, 132.6, 132.3, 130.8, 130.1, 129.0, 128.4, 128.2, 127.6, 123.8, 122.8, 121.7, 121.6, 120.9, 116.0, 113.7, 112.8, 111.6, 106.2, 64.0, 61.4, 56.1, 55.9, 55.8, 55.7, 45.1, 43.9, 42.4, 42.3, 42.1, 39.6, 24.3, 22.1. HR-ESIMS(*m/z*) : 779.3001 [M + H]^+^, calculated for C_44_H_47_N_2_O_9_S: 779.2997.

Compound **18** 7-*O*-(2-Chlorobenzenesulfonyl)-tetrandrine

Beige solid, yield: 93.1%; m.p.: 140.6-143.4 ℃; ^1^H NMR (600 MHz, CDCl_3_) *δ* (ppm): 7.79 (d, *J* = 7.9 Hz, 1H), 7.50 (d, *J* = 3.8 Hz, 2H), 7.35 (dd, *J* = 8.2, 2.2 Hz, 1H), 7.33-7.30 (m, 1H), 7.15 (dd, *J* = 8.2, 2.6 Hz, 1H), 6.86 (s, 2H), 6.79 (dd, *J* = 8.2, 2.6 Hz, 1H), 6.53 (s, 1H), 6.51 (s,1H), 6.31 (dd, *J* = 8.3, 2.2 Hz, 1H), 6.21 (s, 1H), 5.76 (s, 1H), 3.93 (s, 3H), 3.79 (dd, *J* = 11.3, 5.4 Hz, 1H), 3.72 (d, *J* = 10.1 Hz, 1H), 3.53-3.48 (m, 2H), 3.36 (s, 3H), 3.32 (dd, *J* = 12.6, 5.5 Hz, 1H), 3.29 (s, 3H), 3.01-2.88 (m, 4H), 2.78 (t, *J* = 11.9 Hz, 1H), 2.75-2.69 (m, 2H), 2.65 (s, 3H), 2.59 (d, *J* = 13.3 Hz, 1H), 2.44-2.40 (m, 1H), 2.29 (s, 3H). ^13^C NMR (150 MHz, CDCl_3_) *δ* (ppm): 153.8, 150.2, 149.4, 148.8, 148.2, 147.1, 142.5, 136.4, 134.8, 134.6, 133.9, 133.4, 132.6, 132.4, 131.6, 131.1, 130.3, 128.3, 128.3, 127.6, 126.3, 123.5, 122.8, 122.0, 121.8, 121.4, 116.1, 112.7, 111.5, 105.6, 64.0, 61.3, 56.1, 55.9, 55.3, 45.1, 43.8, 42.3, 41.6, 39.5, 39.5, 24.4, 22.2. HR-ESIMS(*m/z*) : 783.2504 [M + H]^+^, calculated for C_43_H_44_ClN_2_O_8_S: 783.2501.

Compound **19** 7-*O*-(3-Chlorobenzenesulfonyl)-tetrandrine

Beige solid, yield: 94.9%; m.p.: 132.4-134.8 ℃; ^1^H NMR (600 MHz, CDCl_3_) *δ* (ppm): 7.71 (t, *J* = 1.9 Hz, 1H), 7.62 (ddd, *J* = 8.0, 2.0, 1.0 Hz, 1H), 7.54 (ddd, *J* = 8.0, 1.8, 1.0 Hz, 1H), 7.40 (t, *J* = 8.0 Hz, 1H), 7.36 (dd, *J* = 8.2, 2.2 Hz, 1H), 7.18 (dd, *J* = 8.2, 2.6 Hz, 1H), 6.89-6.86 (m, 2H), 6.75 (dd, *J* = 8.2, 2.6 Hz, 1H), 6.48 (s, 1H), 6.47 (d, *J* = 1.7 Hz, 1H), 6.31 (s, 1H), 6.26 (dd, *J* = 8.3, 2.2 Hz, 1H), 5.23 (s, 1H), 3.93 (s, 3H), 3.78 (dd, *J* = 11.4, 5.3 Hz, 1H), 3.67 (d, *J* = 10.1 Hz, 1H), 3.55-3.51 (m, 1H), 3.50-3.45 (m, 4H), 3.30-3.27 (m, 4H), 2.98-2.89 (m, 4H), 2.79-2.72 (m, 2H), 2.70-2.67 (m, 1H), 2.66 (s, 3H), 2.55 (d, *J* = 13.5 Hz, 1H), 2.48-2.44 (m, 1H), 2.28 (s, 3H). ^13^C NMR (150 MHz, CDCl_3_) *δ* (ppm): 153.9, 150.8, 149.3, 148.7, 147.8, 147.2, 142.3, 139.0, 134.7, 134.7, 134.5, 133.7, 132.8, 132.6, 130.2, 129.8, 128.6, 128.2, 128.2, 127.6, 126.7, 123.9, 122.9, 121.9, 121.7, 120.8, 116.2, 112.8, 111.5, 106.1, 63.8, 61.4, 56.1, 55.8, 55.7, 44.9, 43.8, 42.3, 42.3, 42.0, 39.2, 24.3, 22.1. HR-ESIMS(*m/z*) : 783.2501 [M + H]^+^, calculated for C_43_H_44_ClN_2_O_8_S: 783.2501.

Compound **20** 7-*O*-(4-Chlorobenzenesulfonyl)-tetrandrine

Beige solid, yield: 92.1%; m.p.: 144.0-146.1 ℃; ^1^H NMR (600 MHz, CDCl_3_) *δ* (ppm): 7.59 (dt, *J* = 8.6, 2.4 Hz, 2H), 7.45 (dt, *J* = 8.6, 2.4 Hz, 2H), 7.38 (dd, *J* = 8.2, 2.2 Hz, 1H), 7.18 (dd, *J* = 8.2, 2.6 Hz, 1H), 6.87 (d, *J* = 1.1 Hz, 2H), 6.76 (dd, *J* = 8.2, 2.6 Hz, 1H), 6.48 (s, 1H), 6.45 (s, 1H), 6.30 (s, 1H), 6.28 (dd, *J* = 8.2, 2.2 Hz, 1H), 5.24 (s, 1H), 3.93 (s, 3H), 3.70 (dd, *J* = 11.3, 5.2 Hz, 1H), 3.66 (d, *J* = 10.2 Hz, 1H), 3.53-3.48 (m, 4H), 3.46-3.41 (m, 1H), 3.30 (s, 3H), 3.27 (dd, *J* = 12.4, 5.3 Hz, 1H), 2.97-2.88 (m, 4H), 2.77-2.69 (m, 3H), 2.66 (s, 3H), 2.53 (d, *J* = 13.4 Hz, 1H), 2.48-2.44 (m, 1H), 2.27 (s, 3H). ^13^C NMR (150 MHz, CDCl_3_) *δ* (ppm): 153.7, 150.8, 149.4, 148.6, 147.8, 147.1, 142.3, 140.1, 135.9, 134.9, 134.4, 132.7, 132.5, 130.3, 130.0, 128.9, 128.2, 128.1, 127.9, 123.8, 122.8, 121.9, 121.8, 120.5, 115.8, 112.8, 111.5, 106.1, 63.9, 63.9, 61.4, 56.1, 55.8, 55.7, 45.0, 43.8, 42.4, 42.3, 42.0, 38.8, 38.7, 24.7, 22.2. HR-ESIMS(*m/z*) : 783.2502 [M + H]^+^, calculated for C_43_H_44_ClN_2_O_8_S: 783.2501.

Compound **21** 7-*O*-(8-Quinolinesulfonyl)-tetrandrine

Beige solid, yield: 90.6%; m.p.: 156.3-157.4 ℃; ^1^H NMR (600 MHz, CDCl_3_) *δ* (ppm): 8.97 (dd, *J* = 4.2, 1.8 Hz, 1H), 8.25 (td, *J* = 8.0, 7.4, 1.6 Hz, 2H), 8.11 (dd, *J* = 8.2, 1.4 Hz, 1H), 7.57 (t, *J* = 7.8 Hz, 1H), 7.46 (dd, *J* = 8.2, 4.2 Hz, 1H), 7.24 (dd, *J* = 8.2, 2.2 Hz, 1H), 7.12 (dd, *J* = 8.2, 2.6 Hz, 1H), 6.91-6.86 (m, 2H), 6.69 (dd, *J* = 8.2, 2.6 Hz, 1H), 6.46 (s, 1H), 6.37 (d, *J* = 1.8 Hz, 1H), 6.20 (s, 1H), 6.18 (dd, *J* = 8.2, 2.2 Hz, 1H), 5.04 (s, 1H), 3.93 (s, 3H), 3.68 (d, *J* = 9.9 Hz, 1H), 3.54-3.49 (m, 2H), 3.45-3.40 (m, 1H), 3.30 (s, 3H), 3.22 (dd, *J* = 12.3, 5.4 Hz, 1H), 3.14 (s, 3H), 2.96-2.85 (m, 4H), 2.78 (dd, *J* = 14.0, 10.1 Hz, 1H), 2.71-2.66 (m, 2H), 2.61-2.59 (m, 4H), 2.43 (dd, *J* = 15.5, 5.3 Hz, 1H), 2.28 (s, 3H). ^13^C NMR (150 MHz, CDCl_3_) *δ* (ppm): 153.9, 151.8, 150.6, 149.2, 148.9, 148.2, 147.2, 144.3, 142.6, 136.0, 135.3, 134.7, 134.5, 134.2, 132.9, 132.5, 131.9, 130.0, 129.1, 128.7, 127.8, 127.1, 125.1, 123.9, 122.9, 122.1, 121.7, 121.6, 120.9, 116.3, 112.7, 111.6, 105.9, 63.5, 61.4, 56.1, 55.7, 55.3, 45.0, 43.9, 42.3, 42.3, 41.9, 38.6, 24.5, 22.0. HR-ESIMS(*m/z*) : 800.3002 [M + H]^+^, calculated for C_46_H_46_N_3_O_8_S: 800.3000.

Compound **22** 7-*O*-(2-Bromobenzenesulfonyl)-tetrandrine

Beige solid, yield: 92.9%; m.p.: 145.9-147.8 ℃; ^1^H NMR (600 MHz, CDCl_3_) *δ* (ppm): 7.82 (dd, *J* = 7.6, 2.0 Hz, 1H), 7.71 (dd, *J* = 7.6, 1.5 Hz, 1H), 7.40-7.34 (m, 3H), 7.15 (dd, *J* = 8.2, 2.6 Hz, 1H), 6.86 (s, 2H), 6.79 (dd, *J* = 8.2, 2.6 Hz, 1H), 6.53 (s, 1H), 6.52 (s, 1H), 6.32 (dd, *J* = 8.2, 2.2 Hz, 1H), 6.21 (s, 1H), 5.82 (s, 1H), 3.93 (s, 3H), 3.80 (dd, *J* = 11.2, 5.4 Hz, 1H), 3.72 (d, *J* = 9.8 Hz, 1H), 3.53-3.47 (m, 2H), 3.36 (s, 3H), 3.32-3.29 (m, 4H), 2.98-2.88 (m, 4zH), 2.78 (t, *J* = 11.8 Hz, 1H), 2.75-2.69 (m, 2H), 2.65 (s, 3H), 2.60 (d, *J* = 13.1 Hz, 1H), 2.44-2.40 (m, 1H), 2.29 (s, 3H). ^13^C NMR (150 MHz, CDCl_3_) *δ* (ppm): 153.8, 150.2, 149.4, 148.8, 148.2, 147.1, 142.5, 138.4, 135.1, 135.0, 134.6, 133.7, 132.6, 132.4, 131.2, 130.3, 128.4, 128.3, 127.9, 126.8, 123.6, 122.8, 122.0, 121.8, 121.5, 121.3, 116.1, 112.7, 111.5, 105.6, 64.0, 61.3, 56.1, 55.9, 55.3, 45.3, 43.8, 42.5, 42.3, 41.6, 39.6, 24.6, 22.1. HR-ESIMS(*m/z*) : 827.1997 [M + H]^+^, calculated for C_43_H_44_BrN_2_O_8_S: 827.1996.

Compound **23** 7-*O*-(3-Bromobenzenesulfonyl)-tetrandrine

Beige solid, yield: 94.7%; m.p.: 126.9-128.1 ℃; ^1^H NMR (600 MHz, CDCl_3_) *δ* (ppm): 7.87 (t, *J* = 1.8 Hz, 1H), 7.77 (ddd, *J* = 8.0, 2.0, 1.0 Hz, 1H), 7.59 (ddd, *J* = 8.0, 2.1, 1.0 Hz,1H), 7.36 (dd, *J* = 8.2, 2.2 Hz, 1H), 7.33 (t, *J* = 8.0 Hz, 1H), 7.18 (dd, *J* = 8.2, 2.6 Hz, 1H), 6.89-6.86 (m, 2H), 6.74 (dd, *J* = 8.2, 2.6 Hz, 1H), 6.48-6.47 (m, 2H), 6.32 (s, 1H), 6.25 (dd, *J* = 8.2, 2.2 Hz, 1H), 5.16 (s, 1H), 3.93 (s, 3H), 3.76 (dd, *J* = 11.3, 5.3 Hz, 1H), 3.66 (d, *J* = 10.1 Hz, 1H), 3.55-3.45 (m, 5H), 3.29-3.26 (m, 4H), 2.97-2.88 (m, 4H), 2.79-2.72 (m, 2H), 2.68-2.64 (m, 4H), 2.55 (d, *J* = 13.8 Hz, 1H), 2.48-2.44 (m, 1H), 2.28 (s, 3H). ^13^C NMR (150 MHz, CDCl_3_) *δ* (ppm): 153.8, 150.9, 149.3, 148.7, 147.8, 147.1, 142.3, 139.2, 136.5, 134.8, 134.5, 132.8, 132.6, 131.4, 130.2, 130.0, 128.3, 128.3, 127.7, 127.2, 123.9, 122.8, 122.4, 121.9, 121.7, 120.7, 116.2, 112.8, 111.5, 106.2, 63.8, 61.4, 56.1, 55.8, 55.8, 45.0, 43.8, 42.3, 42.3, 42.0, 39.5, 24.3, 22.1. HR-ESIMS(*m/z*) : 827.2002 [M + H]^+^, calculated for C_43_H_44_BrN_2_O_8_S: 827.1996.

Compound **24** 7-*O*-(4-Bromobenzenesulfonyl)-tetrandrine

Beige solid, yield: 94.0%; m.p.: 145.7-147.6 ℃; ^1^H NMR (600 MHz, CDCl_3_) *δ* (ppm): 7.62 (dt, *J* = 8.6, 2.4 Hz, 2H), 7.52 (dt, *J* = 8.6, 2.4 Hz, 2H), 7.41 (dd, *J* = 8.2, 2.2 Hz, 1H), 7.19 (dd, *J* = 8.2, 2.6 Hz, 1H), 6.87 (s, 2H), 6.76 (dd, *J* = 8.2, 2.6 Hz, 1H), 6.48 (s, 1H), 6.44 (s, 1H), 6.31 (s, 1H), 6.27 (dd, *J* = 8.2, 2.2 Hz, 1H), 5.24 (s, 1H), 3.93 (s, 3H), 3.70 (dd, *J* = 11.3, 5.2 Hz, 1H), 3.65 (d, *J* = 10.2 Hz, 1H), 3.53-3.43 (m, 5H), 3.30-3.27 (m, 4H), 2.97-2.88 (m, 4H), 2.76-2.68 (m, 3H), 2.67 (s, 3H), 2.53 (d, *J* = 13.3 Hz, 1H), 2.48-2.44 (m, 1H), 2.27 (s, 3H). ^13^C NMR (150 MHz, CDCl_3_) *δ* (ppm): 153.7, 150.8, 149.4, 148.6, 147.8, 147.1, 142.3, 136.4, 134.9, 134.4, 132.7, 132.5, 131.9, 130.4, 130.0, 128.7, 128.2, 128.2, 127.8, 123.7, 122.8, 122.0, 121.8, 120.5, 115.8, 112.8, 111.5, 106.1, 64.0, 61.4, 56.1, 55.8, 55.7, 45.1, 43.9, 42.5, 42.3, 41.9, 39.0, 24.6, 22.2. HR-ESIMS(*m/z*) : 827.1997 [M + H]^+^, calculated for C_43_H_44_BrN_2_O_8_S: 827.1996.

Compound **25** 7-*O*-(4-Acetamidobenzenesulfonyl)-tetrandrine

Beige solid, yield: 88.4%; m.p.: 155.1-157.1 ℃; ^1^H NMR (600 MHz, CDCl_3_) *δ* (ppm): 8.80 (s, 1H), 7.66 (d, *J* = 8.7 Hz, 2H), 7.52 (d, *J* = 8.9 Hz, 2H), 7.31 (dd, *J* = 8.2, 2.2 Hz, 1H), 7.11 (dd, *J* = 8.2, 2.6 Hz, 1H), 6.86 (s, 2H), 6.75 (dd, *J* = 8.2, 2.6 Hz, 1H), 6.48 (s, 1H), 6.45 (s, 1H), 6.26 (dd, *J* = 8.2, 2.2 Hz, 1H), 6.24 (s, 1H), 5.45 (s, 1H), 3.91 (s, 3H), 3.85 (dd, *J* = 11.3, 5.3 Hz, 1H), 3.66 (d, *J* = 10.1 Hz, 1H), 3.52-3.47 (m, 2H), 3.45-3.40 (m, 1H), 3.37 (s, 3H), 3.29-3.26 (m, 4H), 3.00-2.94 (m, 1H), 2.92-2.88 (m, 2H), 2.75-2.69 (m, 3H), 2.66 (s, 3H), 2.54 (d, *J* = 13.5 Hz, 1H), 2.45-2.41 (m, 1H), 2.27 (s, 3H), 2.20 (s,z 3H). ^13^C NMR (150 MHz, CDCl_3_) *δ*(ppm): 169.2, 153.7, 150.8, 149.3, 148.9, 148.1, 147.1, 143.5, 142.5, 134.7, 134.6, 132.6, 132.5, 131.0, 130.2, 129.7, 128.0, 127.8, 127.4, 123.6, 122.9, 121.8, 121.8, 121.1, 118.6, 116.0, 112.6, 111.6, 106.0, 63.6, 61.4, 56.1, 55.8, 55.6, 45.0, 43.8, 42.3, 42.2, 41.8, 38.0, 24.8, 24.6, 22.1. HR-ESIMS(*m/z*) : 806.3104 [M + H]^+^, calculated for C_45_H_48_N_3_O_9_S: 806.3106.

Compound **26** 7-*O*-(Pyridine-3-sulfonyl)-tetrandrine

Beige solid, yield: 92.6%; m.p.: 162.4-165.4 ℃; ^1^H NMR (600 MHz, CDCl_3_) *δ* (ppm): 1H NMR (600 MHz, CDCl_3_) *δ*: 8.85 (d, *J* = 1.9 Hz, 1H), 8.82 (dd, *J* = 4.8, 1.3 Hz, 1H), 7.93 (dt, *J* = 8.1, 1.9 Hz, 1H), 7.40-7.38 (m, 2H), 7.17 (dd, *J* = 8.2, 2.6 Hz, 1H), 6.87 (s, 2H), 6.78 (dd, *J* = 8.2, 2.6 Hz, 1H), 6.52 (s, 1H), 6.47 (s, 1H), 6.30 (dd, *J* = 8.2, 2.2 Hz, 1H), 6.25 (s, 1H), 5.49 (s, 1H), 3.94 (s, 3H), 3.86 (dd, *J* = 11.3, 5.3 Hz, 1H), 3.69 (d, *J* = 10.1 Hz, 1H), 3.54-3.47 (m, 2H), 3.36 (s, 3H), 3.33-3.30 (m, 4H), 3.03-2.90 (m, 4H), 2.79-2.70 (m, 3H), 2.68 (s, 3H), 2.55 (d, *J* = 13.6 Hz, 1H), 2.47-2.43 (m, 1H), 2.28 (s, 3H). ^13^C NMR (150 MHz, CDCl_3_) *δ* (ppm): 153.8, 150.3, 149.4, 149.1, 148.8, 148.0, 147.2, 142.2, 136.0, 134.8, 134.4, 134.0, 133.0, 132.6, 130.4, 128.4, 127.7, 123.8, 123.2, 122.9, 122.0, 121.8, 121.1, 115.9, 112.7, 111.5, 105.9, 63.8, 61.3, 56.1, 55.8, 55.5, 44.9, 43.8, 42.3, 42.2, 41.8, 39.1, 24.4, 22.2. HR-ESIMS(*m/z*) : 750.2839 [M + H]^+^, calculated for C_42_H_44_N_3_O_8_S: 750.2844.

Compound **27** 7-*O*-(2-Chloropyridine-5-sulfonyl)-tetrandrine

Beige solid, yield: 93.5%; m.p.: 118.6-121.6 ℃; ^1^H NMR (600 MHz, CDCl_3_) *δ* (ppm): 8.61 (d, *J* = 2.4 Hz, 1H), 7.84 (dd, *J* = 8.4, 2.4 Hz, 1H), 7.43-7.40 (m, 2H), 7.18 (dd, *J* = 8.2, 2.5 Hz, 1H), 6.86 (s, 2H), 6.78 (dd, *J* = 8.2, 2.5 Hz, 1H), 6.51 (s, 1H), 6.45 (s, 1H), 6.31 (dd, *J* = 8.4, 2.2 Hz, 1H), 6.29 (s, 1H), 5.44 (s, 1H), 3.93 (s, 3H), 3.81 (dd, *J* = 11.3, 5.2 Hz, 1H), 3.67 (d, *J* = 10.2 Hz, 1H), 3.53-3.47 (m, 1H), 3.46-3.42 (m, 4H), 3.32 (s, 3H), 3.28 (dd, *J* = 12.3, 5.2 Hz, 1H), 3.01-2.89 (m, 4H), 2.75(t, *J* = 10.5 Hz, 1H), 2.73-2.68 (m, 2H), 2.67 (s, 3H), 2.52 (d, *J* = 13.8 Hz, 1H), 2.48-2.43 (m, 1H), 2.27 (s, 3H). ^13^C NMR (150 MHz, CDCl_3_) *δ* (ppm): 156.2, 153.6, 150.2, 149.5, 149.4, 148.6, 147.8, 147.1, 142.0, 138.5, 135.0, 134.3, 133.2, 132.9, 132.5, 130.4, 128.6, 128.2, 127.7, 124.2, 123.9, 122.8, 122.1, 121.8, 120.6, 115.7, 112.8, 111.5, 106.1, 63.8, 61.3, 56.1, 55.8, 55.6, 45.0, 43.7, 42.3, 42.3, 41.8, 38.8, 38.7, 24.7, 22.2. HR-ESIMS(*m/z*) : 784.2455 [M + H]^+^, calculated for C_42_H_43_ClN_3_O_8_S: 784.2454.

Compound **28** 7-*O*-(2-Thiophenesulfonyl)-tetrandrine

Beige solid, yield: 94.0%; m.p.: 145.8-148.6 ℃; ^1^H NMR (600 MHz, CDCl_3_) *δ* (ppm): 7.64 (dd, *J* = 5.0, 1.2 Hz, 1H), 7.47-7.46 (m, 1H), 7.35 (dd, *J* = 8.2, 2.2 Hz, 1H), 7.15 (dd, *J* = 8.1, 2.4 Hz, 1H), 7.08-7.06 (m, 1H), 6.88-6.85 (m, 2H), 6.78 (dd, *J* = 8.2, 2.6 Hz, 1H), 6.50 (s, 1H), 6.49 (s, 1H), 6.29 (dd, *J* = 8.2, 2.2 Hz, 1H), 6.27 (s, 1H), 5.53 (s, 1H), 3.93 (d, *J* = 1.2 Hz, 3H), 3.80 (dd, *J* = 11.3, 5.3 Hz, 1H), 3.71 (d, *J* = 10.0 Hz, 1H), 3.51-3.48 (m, 1H), 3.46 (s, 1H), 3.32 (s, 3H), 3.29 (dd, *J* = 12.4, 5.4 Hz, 1H), 3.00-2.89 (m, 4H), 2.79-2.74 (m, 2H), 2.72-2.68 (m, 1H), 2.65 (s, 3H), 2.59 (d, *J* = 13.8 Hz, 1H), 2.47-2.43 (m, 1H), 2.29 (s, 3H). ^13^C NMR (150 MHz, CDCl_3_) *δ* (ppm): 153.8, 150.9, 149.4, 148.9, 148.0, 147.1, 142.4, 136.9, 134.8, 134.6, 134.6, 133.7, 132.6, 132.6, 130.3, 128.2, 127.9, 127.6, 127.0, 123.7, 122.8, 121.8, 121.8, 121.3, 116.0, 112.6, 111.5, 105.8, 63.9, 61.3, 56.1, 55.8, 55.7, 45.0, 43.8, 42.3, 42.3, 41.8, 39.3, 24.3, 22.2. HR-ESIMS(*m/z*) : 755.2452 [M + H]^+^, calculated for C_41_H_43_N_2_O_8_S_2_: 755.2455.

Compound **29** 7-*O*-(5-Chlorothiophene-2-sulfonyl)-tetrandrine

Beige solid, yield: 92.5%; m.p.: 115.1-118.4 ℃; ^1^H NMR (600 MHz, CDCl_3_) *δ* (ppm): 7.38 (dd, *J* = 8.2, 2.2 Hz, 1H), 7.24 (d, *J* = 4.1 Hz, 1H), 7.17 (dd, *J* = 8.2, 2.6 Hz, 1H), 6.92 (d, *J* = 4.1 Hz, 1H), 6.87 (s, 2H), 6.78 (dd, *J* = 8.2, 2.6 Hz, 1H), 6.50 (s, 1H), 6.48 (s, 1H), 6.31-6.30 (m, 2H), 5.53 (s, 1H), 3.93 (s, 3H), 3.81 (dd, *J* = 11.3, 5.3 Hz, 1H), 3.69 (d, *J* = 10.0 Hz, 1H), 3.55 (s, 3H), 3.54-3.47 (m, 2H), 3.33-3.30 (m, 4H), 2.99-2.90 (m, 4H), 2.79-2.69 (m, 3H), 2.66 (s, 3H), 2.56 (d, *J* = 13.4 Hz, 1H), 2.48-2.44 (m, 1H), 2.29 (s, 3H). ^13^C NMR (150 MHz, CDCl_3_) *δ* (ppm): 153.8, 150.8, 149.4, 148.8, 148.0, 147.1, 142.3, 139.1, 134.8, 134.5, 134.0, 132.9, 132.6, 130.3, 128.3, 127.8, 127.6, 126.6, 123.8, 122.8, 122.0, 121.8, 121.0, 116.0, 112.7, 111.5, 105.9, 64.1, 61.3, 56.1, 55.8, 55.8, 45.0, 43.8, 42.4, 42.3, 41.9, 39.5, 24.3, 22.2. HR-ESIMS(*m/z*) : 789.2064 [M + H]^+^, calculated for C_41_H_44_ClN_2_O_8_S_2_: 789.2066.

Compound **30** 7-*O*-(Biphenyl-4-sulfonyl)-tetrandrine

Beige solid, yield: 91.3%; m.p.: 139.1-141.3 ℃; ^1^H NMR (600 MHz, CDCl_3_) *δ* (ppm): 7.72-7.68 (m, 4H), 7.64-7.62 (m, 2H), 7.52-7.49 (m, 2H), 7.47-7.44 (m, 1H), 7.02 (dd, *J* = 8.2, 2.2 Hz, 1H), 6.88 (s, 2H), 6.86 (dd, *J* = 8.2, 2.6 Hz, 1H), 6.72 (dd, *J* = 8.2, 2.6 Hz, 1H), 6.49 (s, 1H), 6.44 (s, 1H), 6.31 (s, 1H), 6.22 (dd, *J* = 8.2, 2.2 Hz, 1H), 5.32 (s, 1H), 3.93 (s, 3H), 3.69-3.66 (m, 2H), 3.54-3.49 (m, 1H), 3.48-3.42 (m, 4H), 3.31 (s, 3H), 3.18 (dd, *J* = 12.2, 5.3 Hz, 1H), 2.99-2.89 (m, 4H), 2.77 (dd, *J* = 14.0, 10.3 Hz, 1H), 2.72-2.66 (m, 2H), 2.64 (s, 3H), 2.59 (d, *J* = 13.3 Hz, 1H), 2.49-2.45 (m, 1H), 2.28 (s, 3H). ^13^C NMR (150 MHz, CDCl_3_) *δ* (ppm): 153.5, 151.0, 149.4, 148.7, 147.9, 147.1, 146.1, 142.4, 138.7, 135.8, 134.7, 134.5, 132.5, 132.4, 130.2, 129.2, 129.1, 129.0, 128.2, 128.1, 127.6, 127.2, 126.8, 123.6, 122.8, 121.8, 121.7, 120.9, 115.8, 112.8, 111.5, 106.1, 63.9, 61.4, 56.1, 55.8, 55.7, 45.0, 43.9, 42.4, 42.3, 41.9, 39.0, 24.4, 22.2. HR-ESIMS(*m/z*) : 825.3199 [M + H]^+^, calculated for C_49_H_49_N_2_O_8_S: 825.3204.

Compound **31** 7-*O*-(4-Cyanobenzenesulfonyl)-tetrandrine

Beige solid, yield: 92.1%; m.p.: 129.5-130.6 ℃; ^1^H NMR (600 MHz, CDCl_3_) *δ* (ppm): 1H NMR (600 MHz, CDCl_3_) *δ*: 7.80-7.75 (m, 4H), 7.40 (dd, *J* = 8.2, 2.2 Hz, 1H), 7.20 (dd, *J* = 8.2, 2.6 Hz, 1H), 6.87 (s, 2H), 6.78 (dd, *J* = 8.2, 2.6 Hz, 1H), 6.51 (s, 1H), 6.46 (s, 1H), 6.29 (dd, *J* = 8.2, 2.2 Hz, 1H), 6.27 (s, 1H), 5.46 (s, 1H), 3.94 (s, 3H), 3.75 (dd, *J* = 11.3, 5.2 Hz, 1H), 3.68 (d, *J* = 10.2 Hz, 1H), 3.54-3.48 (m, 1H), 3.46-3.41 (m, 1H), 3.39 (s, 3H), 3.33 (s, 3H), 3.29 (dd, *J* = 12.3, 5.2 Hz, 1H), 3.00-2.89 (m, 4H), 2.76-2.72 (m, 3H), 2.68 (s, 3H), 2.55 (d, *J* = 13.4 Hz, 1H), 2.48-2.44 (m, 1H), 2.28 (s, 3H). ^13^C NMR (150 MHz, CDCl_3_) *δ* (ppm): 153.7, 150.3, 149.5, 148.7, 148.0, 147.2, 142.1, 141.4, 134.9, 134.3, 133.0, 132.5, 132.3, 130.3, 129.1, 128.4, 128.1, 127.7, 123.9, 122.8, 122.1, 121.9, 120.7, 117.2, 117.0, 115.8, 112.7, 111.6, 106.0, 64.0, 61.3, 56.1, 55.8, 55.5, 45.0, 43.8, 42.5, 42.3, 41.8, 38.1, 24.9, 22.2. HR-ESIMS(*m/z*) : 774.2840 [M + H]^+^, calculated for C_44_H_44_N_3_O_8_S: 774.2844.

Compound **32** 7-*O*-(3-Cyanobenzenesulfonyl)-tetrandrine

Beige solid, yield: 93.4%; m.p.: 136.5-138.7 ℃; ^1^H NMR (600 MHz, CDCl_3_) *δ* (ppm): 1H NMR (600 MHz, CDCl_3_) *δ*: 8.00 (t, *J* = 1.7 Hz, 1H), 7.89-7.87 (m, 2H), 7.58 (t, *J* = 7.9 Hz, 1H), 7.39 (dd, *J* = 8.2, 2.2 Hz, 1H), 7.18 (dd, *J* = 8.2, 2.6 Hz, 1H), 6.87 (s, 2H), 6.78 (dd, *J* = 8.2, 2.6 Hz, 1H), 6.52 (s, 1H), 6.47 (s, 1H), 6.30 (dd, *J* = 8.2, 2.2 Hz, 1H), 6.25 (s, 1H), 5.60 (s, 1H), 3.94 (s, 3H), 3.85 (dd, *J* = 11.3, 5.3 Hz, 1H), 3.69 (d, *J* = 10.1 Hz, 1H), 3.55-3.44 (m, 2H), 3.35 (s, 3H), 3.33-3.30 (m, 4H), 3.03-2.90 (m, 4H), 2.79-2.72 (m, 3H), 2.68 (s, 3H), 2.55 (d, *J* = 13.4 Hz, 1H), 2.48-2.44 (m, 1H), 2.28 (s, 3H). ^13^C NMR (150 MHz, CDCl_3_) *δ* (ppm): 153.8, 150.1, 149.4, 148.9, 148.0, 147.1, 142.1, 139.0, 136.4, 134.9, 134.4, 133.1, 132.6, 132.4, 132.0, 130.3, 129.6, 128.5, 128.0, 127.6, 123.9, 122.9, 122.0, 121.8, 121.2, 117.0, 116.0, 113.1, 112.7, 111.5, 105.9, 63.9, 61.3, 56.1, 55.8, 55.5, 45.0, 43.7, 42.3, 42.3, 41.8, 38.5, 24.7, 22.2. HR-ESIMS(*m/z*) : 774.2842 [M + H]^+^, calculated for C_44_H_44_N_3_O_8_S: 774.2844.

Compound **33** 7-*O*-(Cyclopropanesulfonyl)-tetrandrine

Beige solid, yield: 32.3%; m.p.: 136.1-138.1 ℃; ^1^H NMR (600 MHz, CDCl_3_) *δ* (ppm): 1H NMR (600 MHz, CDCl_3_) *δ*: 7.36 (dd, *J* = 8.2, 2.2 Hz, 1H), 7.14 (dd, *J* = 8.2, 2.6 Hz, 1H), 6.85 (d, *J* = 1.1 Hz, 2H), 6.81 (dd, *J* = 8.2, 2.6 Hz, 1H), 6.55 (s, 1H), 6.49 (s, 1H), 6.38 (s, 1H), 6.33 (dd, *J* = 8.2, 2.2 Hz, 1H), 6.03 (s, 1H), 3.93 (s, 3H), 3.88 (dd, *J* = 11.3, 5.3 Hz, 1H), 3.78 (s, 3H), 3.72 (d, *J* = 9.9 Hz, 1H), 3.54-3.49 (m, 2H), 3.40-3.35 (m, 4H), 2.99-2.90 (m, 4H), 2.82-2.77 (m, 2H), 2.70 (dd, *J* = 14.0, 10.3 Hz, 1H), 2.62 (s, 3H), 2.58 (d, *J* = 13.5 Hz, 1H), 2.48 (dd, *J* = 16.0, 5.1 Hz, 1H), 2.33-2.28 (m, 4H), 1.08-1.02 (m, 2H), 0.93-0.86 (m, 2H). ^13^C NMR (150 MHz, CDCl_3_) *δ* (ppm): 153.8, 150.6, 149.4, 148.7, 148.5, 147.1, 142.6, 134.5, 132.5, 132.3, 130.4, 128.8, 128.1, 127.9, 123.6, 122.8, 122.2, 122.0, 121.7, 115.9, 112.6, 111.5, 106.1, 64.2, 61.4, 56.1, 56.1, 55.8, 45.2, 43.8, 42.3, 42.2, 41.5, 39.2, 28.9, 24.4, 22.2, 6.4, 5.8. HR-ESIMS(*m/z*) : 713.2889 [M + H]^+^, calculated for C_40_H_45_N_2_O_8_S: 713.2891.

Compound **34** 7-*O*-(2-Phthalimidoethanesulfonyl)-tetrandrine

Beige solid, yield: 90.2%; m.p.: 126.6-128.8 ℃; ^1^H NMR (600 MHz, CDCl_3_) *δ* (ppm): 1H NMR (600 MHz, CDCl_3_) *δ*: 7.83 (dd, *J* = 5.4, 3.0 Hz, 2H), 7.72 (dd, *J* = 5.4, 3.0 Hz, 2H), 7.37 (dd, *J* = 8.2, 2.2 Hz, 1H), 7.14 (dd, *J* = 8.2, 2.6 Hz, 1H), 6.84 (s, 2H), 6.80 (dd, *J* = 8.2, 2.6 Hz, 1H), 6.54 (s, 1H), 6.47 (s, 1H), 6.37 (s, 1H), 6.32 (dd, *J* = 8.2, 2.2 Hz, 1H), 6.05 (s, 1H), 4.11-4.02 (m, 2H), 3.92 (s, 3H), 3.89 (dd, *J* = 11.3, 5.4 Hz, 1H), 3.80 (s, 3H), 3.70 (d, *J* = 10.2 Hz, 1H), 3.53-3.47 (m, 3H), 3.44-3.39 (m, 1H), 3.37 (s, 3H), 3.33 (dd, *J* = 12.4, 5.4 Hz, 1H), 3.00-2.83 (m, 4H), 2.80-2.73 (m, 2H), 2.67 (dd, *J* = 14.0, 10.3 Hz, 1H), 2.55 (d, *J* = 13.6 Hz, 1H), 2.51 (s, 3H), 2.45 (dd, *J* = 15.8, 5.1 Hz, 1H), 2.28 (s, 3H). ^13^C NMR (150 MHz, CDCl_3_) *δ* (ppm): 167.4, 153.7, 150.1, 149.4, 148.9, 148.3, 147.0, 142.1, 134.7, 134.5, 134.2, 132.7, 132.4, 131.8, 130.5, 128.1, 127.5, 123.6, 123.4, 122.8, 122.2, 121.9, 121.8, 115.8, 112.5, 111.4, 106.0, 63.8, 61.3, 56.1, 56.0, 55.8, 49.2, 44.9, 43.7, 42.3, 42.0, 41.3, 38.8, 32.5, 24.3, 22.2. HR-ESIMS(*m/z*) : 846.3052 [M + H]^+^, calculated for C_47_H_48_N_3_O_10_S: 846.3055.

Compound **35** 7-*O*-(1-Dodecanesulfonyl)-tetrandrine

Beige solid, yield: 38.1%; m.p.: 83.4-84.3 ℃; ^1^H NMR (600 MHz, CDCl_3_) *δ* (ppm): 1H NMR (600 MHz, CDCl_3_) *δ*: 7.36 (dd, *J* = 8.2, 2.2 Hz, 1H), 7.14 (dd, *J* = 8.2, 2.6 Hz, 1H), 6.86-6.81 (m, 2H), 6.81 (dd, *J* = 8.2, 2.6 Hz, 1H), 6.53 (s, 1H), 6.49 (d, *J* = 1.6 Hz, 1H), 6.38 (s, 1H), 6.34 (dd, *J* = 8.2, 2.2 Hz, 1H), 6.03 (s, 1H), 3.93 (s, 3H), 3.85 (dd, *J* = 11.2, 5.3 Hz, 1H), 3.77 (s, 3H), 3.70 (d, *J* = 10.0 Hz, 1H), 3.53-3.44 (m, 2H), 3.36 (s, 3H), 3.28 (dd, *J* = 12.5, 5.3 Hz, 1H), 2.98-2.87 (m, 6H), 2.80 (t, *J* = 11.8 Hz, 1H), 2.75-2.67 (m, 2H), 2.61 (s, 3H), 2.58 (d, *J* = 13.5 Hz, 1H), 2.48-2.44 (m, 1H), 2.29 (s, 3H), 1.79-1.65 (m, 2H), 1.32-1.24 (m, 18H), 0.88 (t, *J* = 7.0 Hz, 3H). ^13^C NMR (150 MHz, CDCl_3_) *δ* (ppm): 153.7, 150.4, 149.4, 148.9, 148.1, 147.0, 142.3, 135.0, 134.5, 132.5, 132.4, 130.4, 128.4, 128.2, 127.8, 123.7, 122.8, 122.2, 121.9, 121.6, 115.9, 112.6, 111.4, 106.2, 64.1, 61.4, 56.1, 56.1, 55.9, 51.9, 45.2, 43.8, 42.5, 42.3, 41.5, 39.0, 31.9, 29.6, 29.5, 29.3, 29.1, 28.3, 24.7, 23.3, 22.7, 22.2, 14.1. HR-ESIMS(*m/z*) : 841.4453 [M + H]^+^, calculated for C_49_H_65_N_2_O_8_S: 841.4456.

Compound **36** 7-*O*-(1-Propanesulfonyl)-tetrandrine

Beige solid, yield: 42.3%; m.p.: 122.7-125.3 ℃; ^1^H NMR (600 MHz, CDCl_3_) *δ* (ppm): 1H NMR (600 MHz, CDCl_3_) *δ*: 7.36 (dd, *J* = 8.2, 2.2 Hz, 1H), 7.14 (dd, *J* = 8.2, 2.5 Hz, 1H), 6.86-6.82 (m, 2H), 6.81 (dd, *J* = 8.2, 2.6 Hz, 1H), 6.53 (s, 1H), 6.50 (d, *J* = 1.7 Hz, 1H), 6.38 (s, 1H), 6.34 (dd, *J* = 8.2, 2.2 Hz, 1H), 6.04 (s, 1H), 3.93 (s, 3H), 3.85 (dd, *J* = 11.2, 5.4 Hz, 1H), 3.78 (s, 3H), 3.70 (d, *J* = 10.0 Hz, 1H), 3.53-3.44 (m, 2H), 3.36 (s, 3H), 3.28 (dd, *J* = 12.4, 5.3 Hz, 1H), 2.99-2.85 (m, 6H), 2.79 (t, *J* = 11.8 Hz, 1H), 2.75-2.67 (m, 2H), 2.63-2.57 (m, 4H), 2.48-2.44 (m, 1H), 2.29 (s, 3H), 1.83-1.70 (m, 2H), 0.97 (t, *J* = 7.5 Hz, 3H). ^13^C NMR (150 MHz, CDCl_3_) *δ* (ppm): 153.7, 150.4, 149.4, 148.9, 148.1, 147.0, 142.3, 135.1, 134.5, 132.5, 132.4, 130.4, 128.4, 128.3, 127.7, 123.7, 122.8, 122.2, 121.9, 121.6, 115.9, 112.6, 111.4, 106.2, 64.1, 61.4, 56.1, 56.1, 55.9, 53.5, 45.2, 43.8, 42.5, 42.3, 41.5, 39.1, 24.7, 22.2, 17.1, 12.9. HR-ESIMS(*m/z*) : 715.3042 [M + H]^+^, calculated for C_40_H_47_N_2_O_8_S: 715.3048.

PJ-0548 7-*O*-(2-Naphthalenesulfonyl)-tetrandrine

Beige solid, yield: 95.3%; m.p.: 116.1-117.2 ℃; ^1^H NMR (600 MHz, CDCl_3_) *δ* (ppm): 1H NMR (600 MHz, CDCl_3_) *δ*: 8.31 (s, 1H), 8.02 (d, *J* = 8.2 Hz, 1H), 7.96 (d, *J* = 8.2 Hz, 1H), 7.91 (d, *J* = 8.7 Hz, 1H), 7.79 (ddd, *J* = 8.2, 6.9, 1.2 Hz, 1H), 7.67 (ddd, *J* = 8.2, 6.9, 1.2 Hz, 1H), 7.65 (dd, *J* = 8.7, 1.8 Hz, 1H), 6.92 (dd, *J* = 8.2, 2.6 Hz, 1H), 6.90-6.88 (m, 2H), 6.75 (dd, *J* = 8.2, 2.2 Hz, 1H), 6.65 (dd, *J* = 8.2, 2.6 Hz, 1H), 6.45 (s, 1H), 6.36 (s, 1H), 6.32 (d, *J* = 1.8 Hz, 1H), 6.14 (dd, *J* = 8.2, 2.2 Hz, 1H), 4.60 (s, 1H), 3.94 (s, 3H), 3.64 (d, *J* = 10.1 Hz, 1H), 3.56-3.53 (m, 1H), 3.51 (s, 3H), 3.41-3.36 (m, 1H), 3.29-3.26 (m, 4H), 3.04 (dd, *J* = 12.3, 5.3 Hz, 1H), 2.99-2.86 (m, 5H), 2.82 (dd, *J* = 14.0, 10.3 Hz, 1H), 2.66-2.63 (m, 4H), 2.62-2.56 (m, 2H), 2.51-2.47 (m, 1H), 2.28 (s, 3H). ^13^C NMR (150 MHz, CDCl_3_) *δ* (ppm): 153.5, 151.4, 149.3, 148.5, 147.6, 147.1, 142.4, 135.3, 134.6, 134.5, 134.4, 132.4, 132.3, 131.9, 130.0, 129.9, 129.6, 129.4, 128.7, 128.6, 128.1, 127.9, 127.7, 127.5, 123.8, 122.8, 121.6, 121.5, 120.1, 115.9, 112.8, 111.5, 106.4, 63.4, 61.4, 56.1, 55.9, 55.8, 44.9, 43.9, 42.3, 42.1, 38.6, 24.5, 22.1. HR-ESIMS(*m/z*) : 799.3041 [M + H]^+^, calculated for C_47_H_47_N_2_O_8_S: 799.3048.

PJ-0549 7-*O*-(Ethanesulfonyl)-tetrandrine

Beige solid, yield: 52.8%; m.p.: 128.1-129.3 ℃; ^1^H NMR (600 MHz, CDCl_3_) *δ* (ppm): 1H NMR (600 MHz, CDCl_3_) *δ*: 7.38 (dd, *J* = 8.2, 2.2 Hz, 1H), 7.15 (dd, *J* = 8.2, 2.6 Hz, 1H), 6.86-6.83 (m, 2H), 6.81 (dd, *J* = 8.2, 2.6 Hz, 1H), 6.55 (s, 1H), 6.49 (s, 1H), 6.38 (s, 1H), 6.34 (dd, *J* = 8.2, 2.2 Hz, 1H), 6.05 (s, 1H), 3.93 (s, 3H), 3.91 (dd, *J* = 11.3, 5.4 Hz, 1H), 3.78 (s, 3H), 3.72 (d, *J* = 10.0 Hz, 1H), 3.54-3.48 (m, 2H), 3.38-3.34 (m, 4H), 3.03-2.89 (m, 6H), 2.8-2.76 (m, 2H), 2.70 (dd, *J* = 14.0, 10.3 Hz, 1H), 2.64 (s, 3H), 2.58 (d, *J* = 13.2 Hz, 1H), 2.47 (dd, *J* = 16.3, 5.1 Hz, 1H), 2.29 (s, 3H), 1.28 (t, *J* = 7.4 Hz, 3H). ^13^C NMR (150 MHz, CDCl_3_) *δ* (ppm): 145.6, 142.2, 141.3, 140.8, 140.3, 138.9, 134.2, 126.5, 126.3, 124.4, 124.3, 122.3, 119.9, 119.6, 119.4, 115.6, 114.7, 114.1, 113.9, 113.5, 107.7, 104.4, 103.3, 98.0, 55.9, 53.3, 48.0, 47.9, 47.7, 38.4, 37.0, 35.7, 34.2, 34.1, 33.3, 30.7, 21.6, 16.4, 14.1. HR-ESIMS(*m/z*) : 701.2886 [M + H]^+^, calculated for C_39_H_45_N_2_O_8_S: 701.2891.

# NMR and MS Spectra

**Compound 1. 7-*O*-(3,5-Dichlorobenzenesulfonyl)-tetrandrine: ^1^H-NMR spectrum**


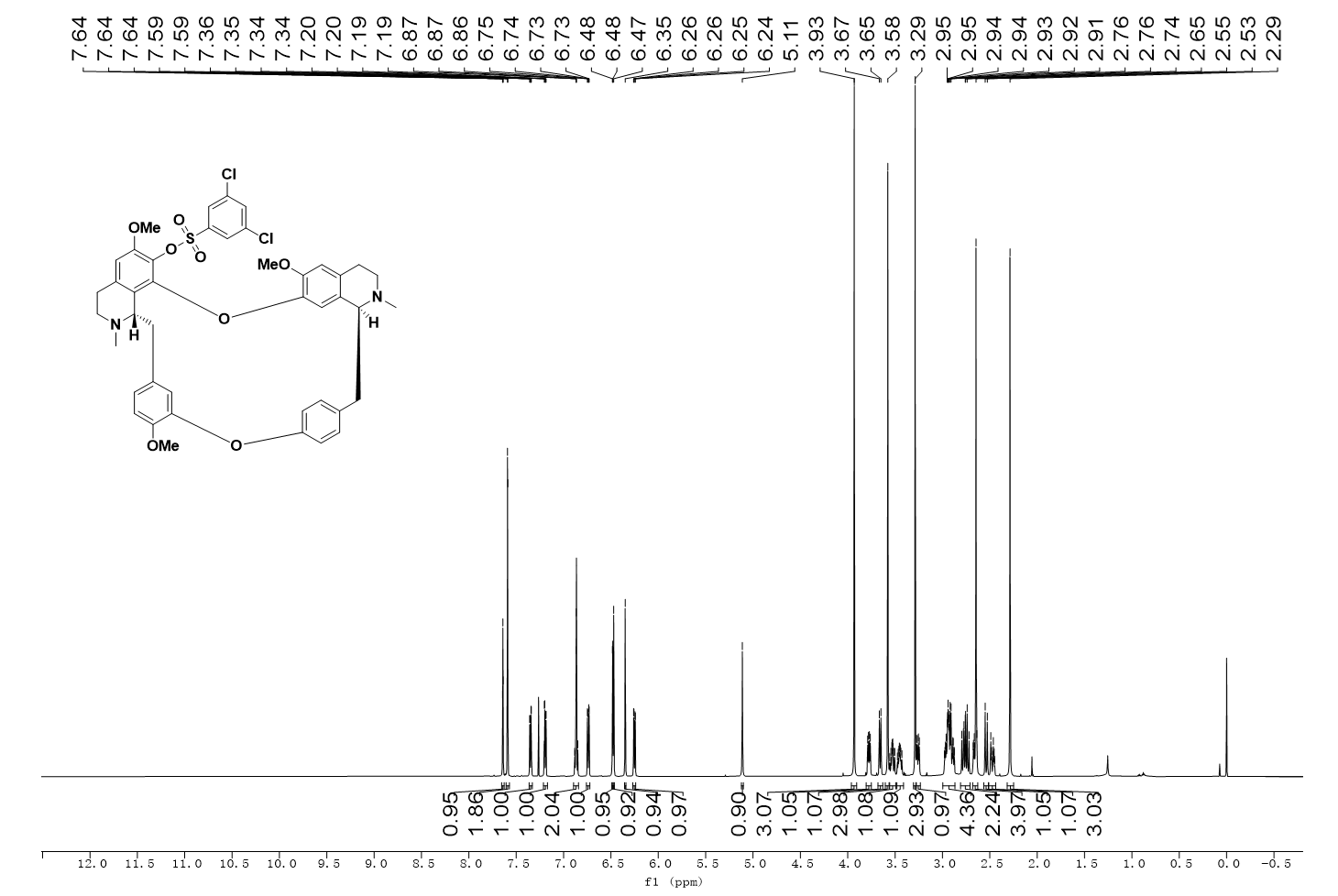


**Compound 1. 7-*O*-(3,5-Dichlorobenzenesulfonyl)-tetrandrine: ^13^C-NMR spectrum**


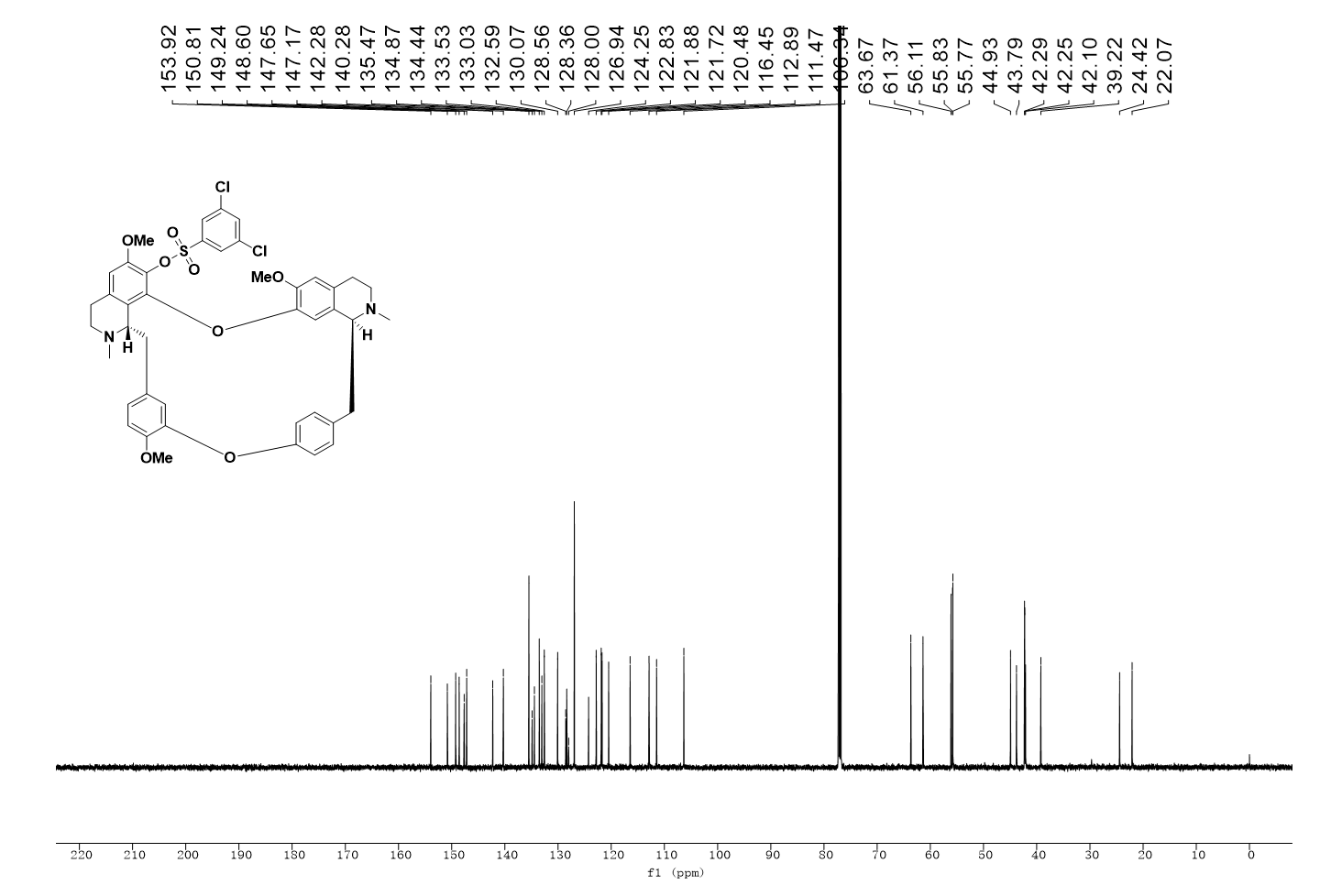


**Compound 1. 7-*O*-(3,5-Dichlorobenzenesulfonyl)-tetrandrine: HR-ESIMS spectrum**


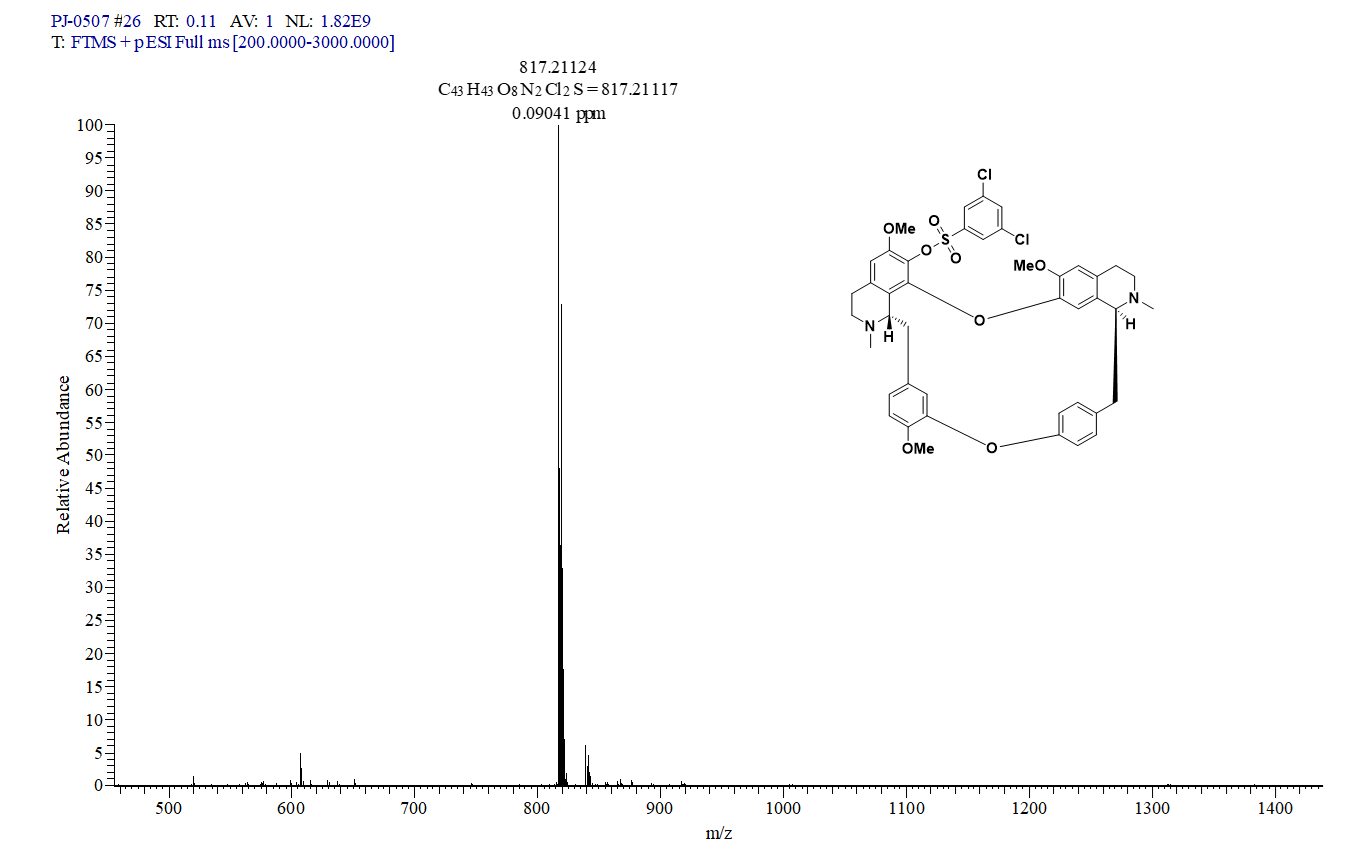


**Compound 2. 7-*O*-Trifluoromathanesulfonyl- tetrandrine: ^1^H-NMR spectrum**


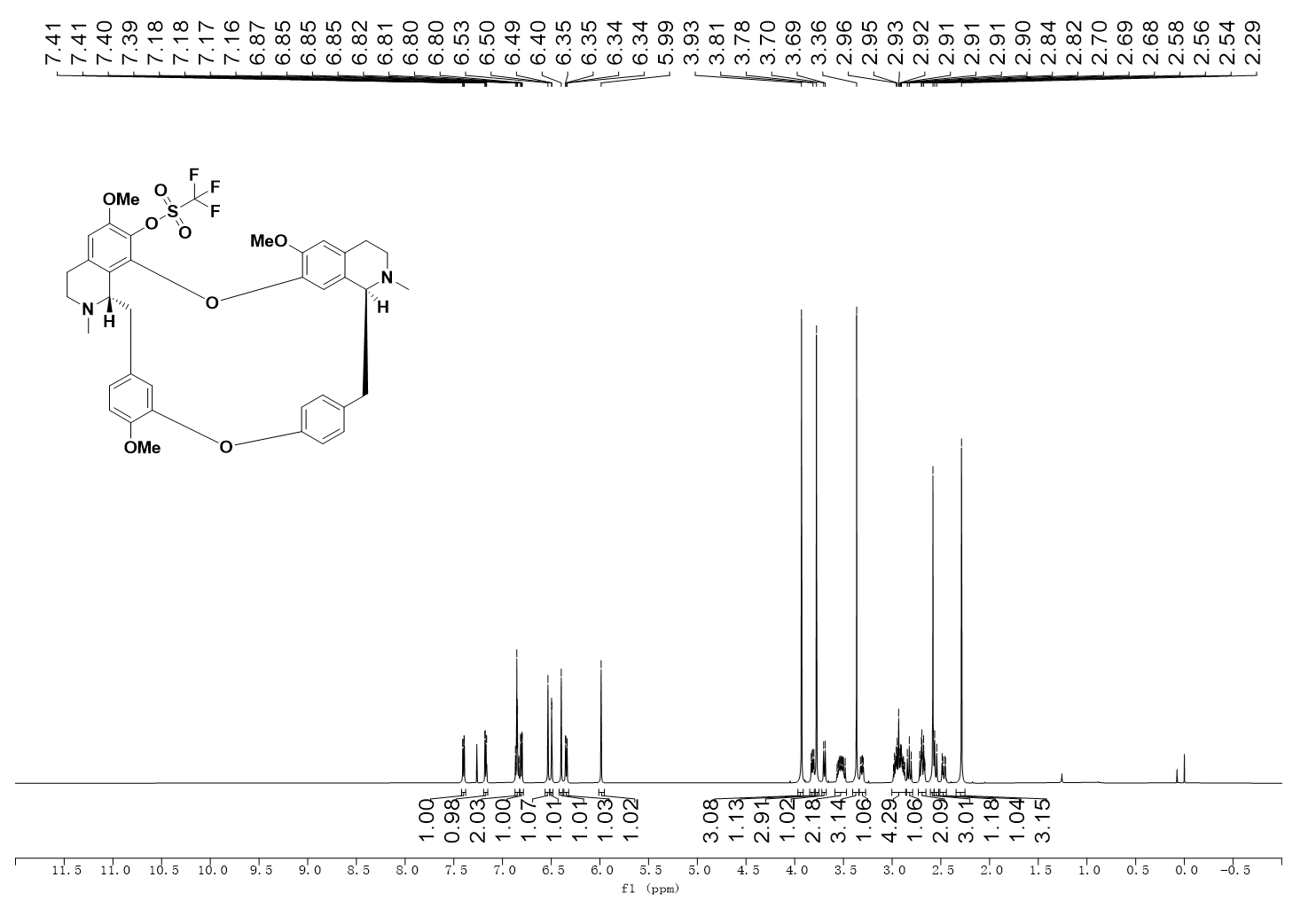


**Compound 2. 7-*O*-Trifluoromathanesulfonyl- tetrandrine: ^13^C-NMR spectrum**


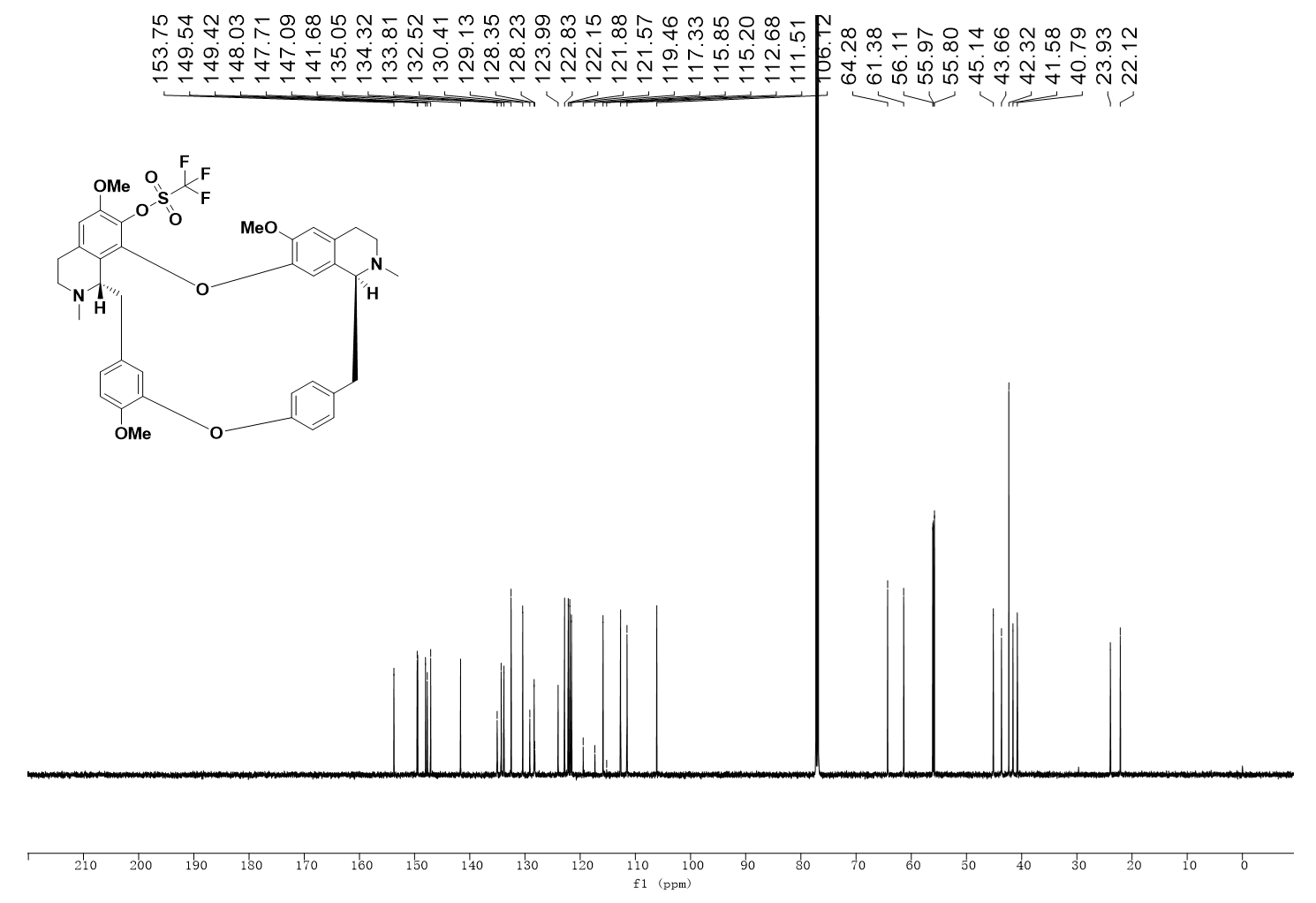


**Compound 2. 7-*O*-Trifluoromathanesulfonyl- tetrandrine: ^19^F-NMR spectrum**


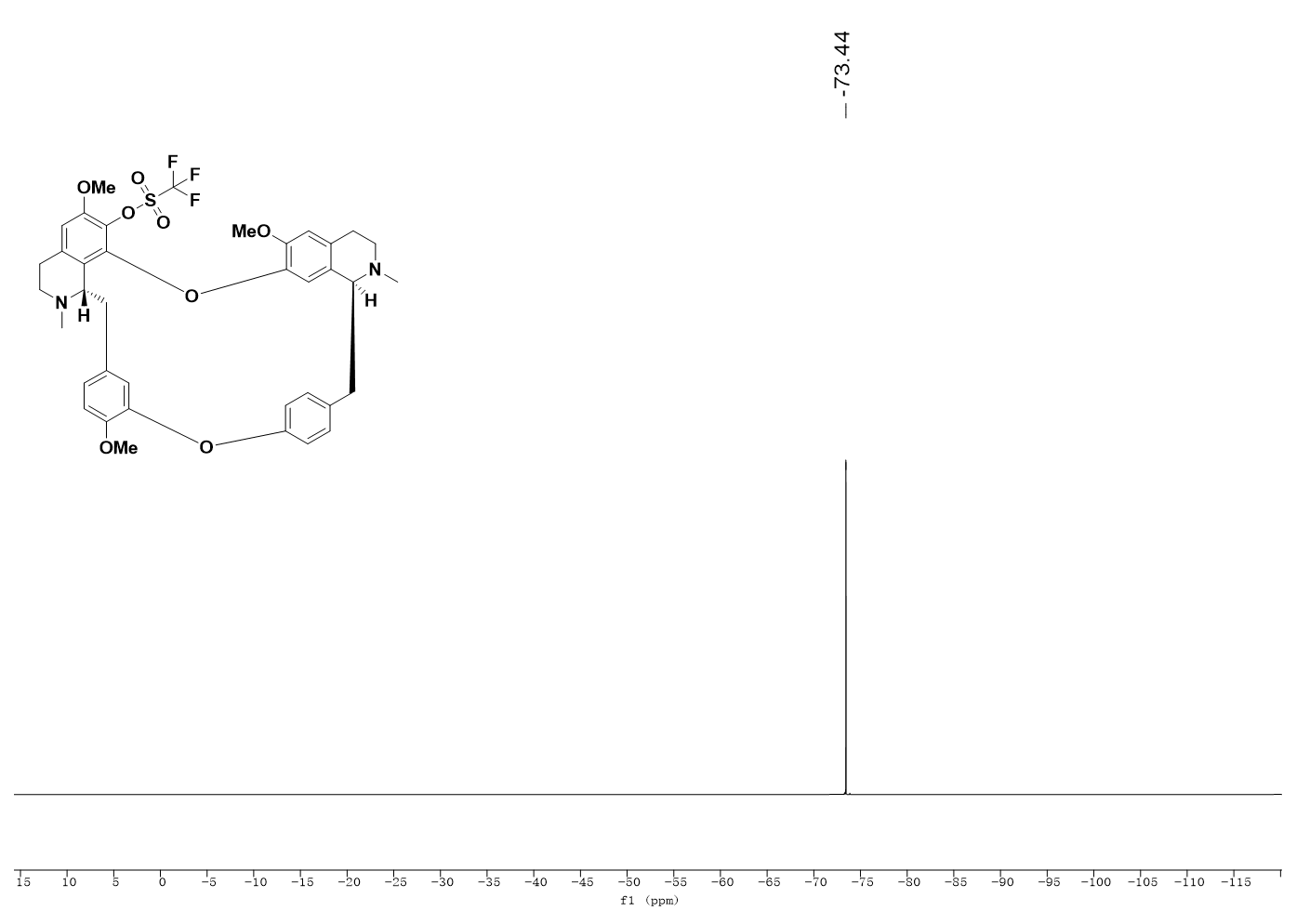


**Compound 2. 7-*O*-Trifluoromathanesulfonyl- tetrandrine: HR-ESIMS spectrum**


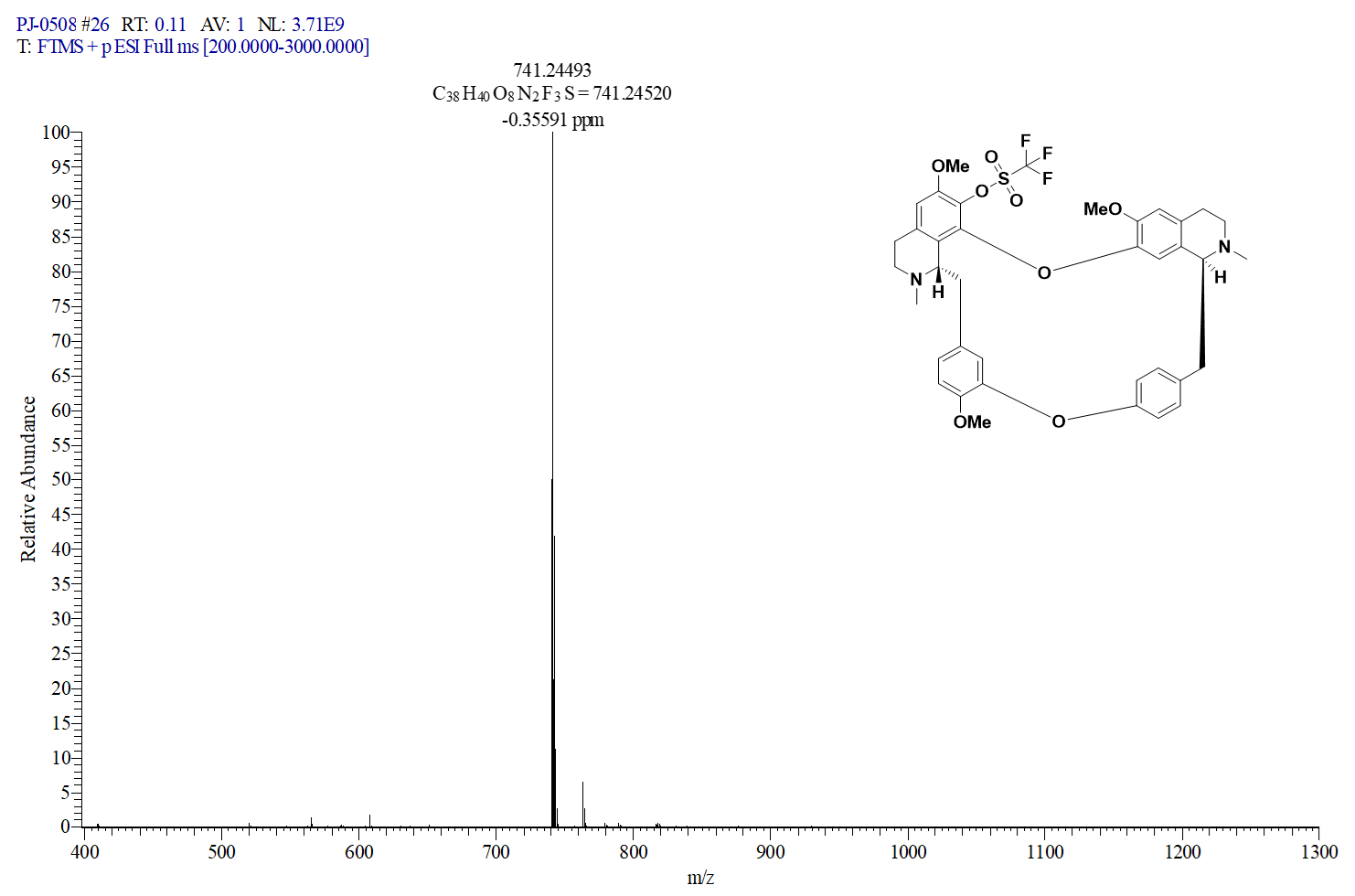


**Compound 3. 7-*O*-(2,4,6-Trimethylbenzenesulfonyl)-tetrandrine: ^1^H-NMR spectrum**


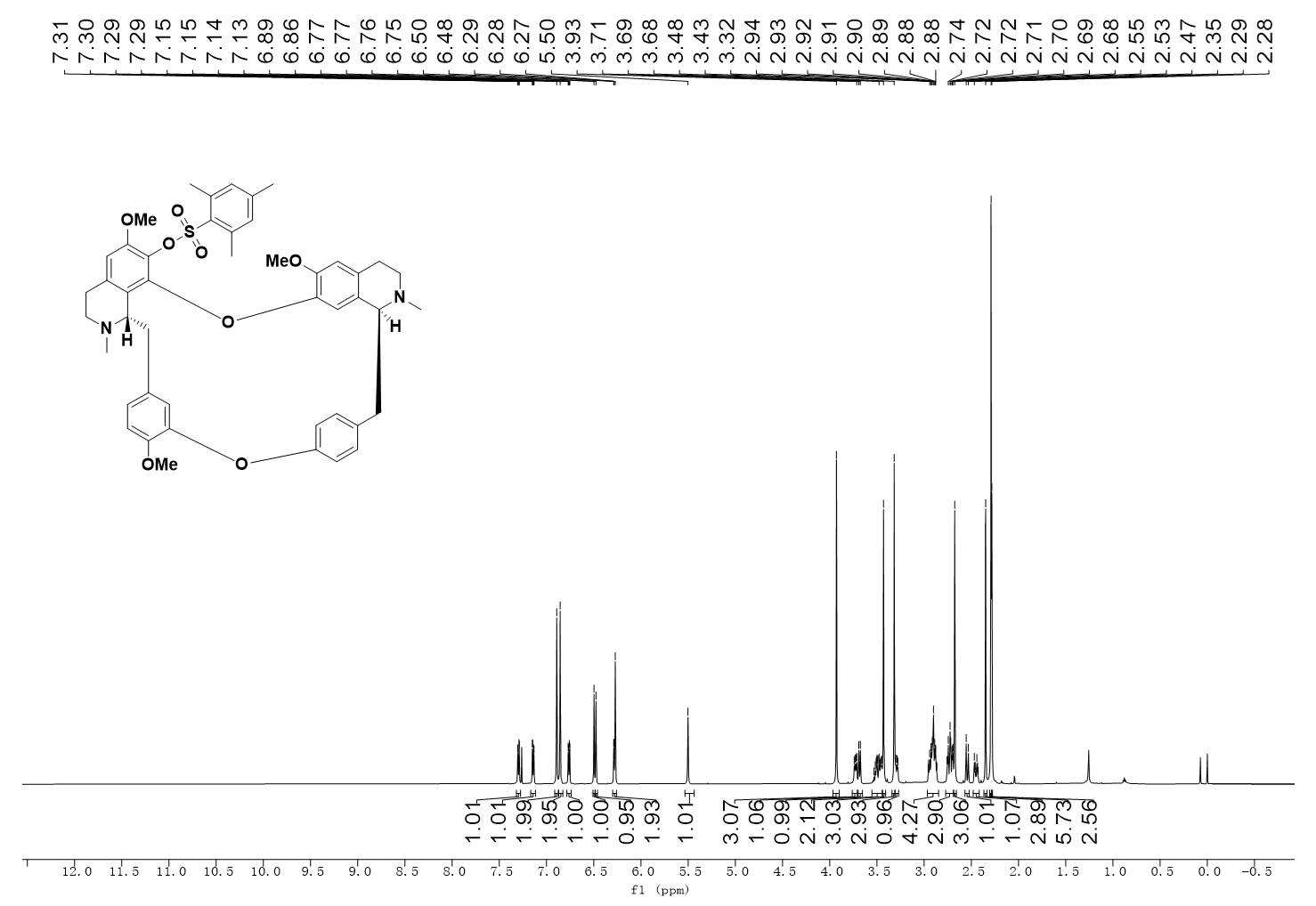


**Compound 3. 7-*O*-(2,4,6-Trimethylbenzenesulfonyl)-tetrandrine: ^13^C-NMR spectrum**


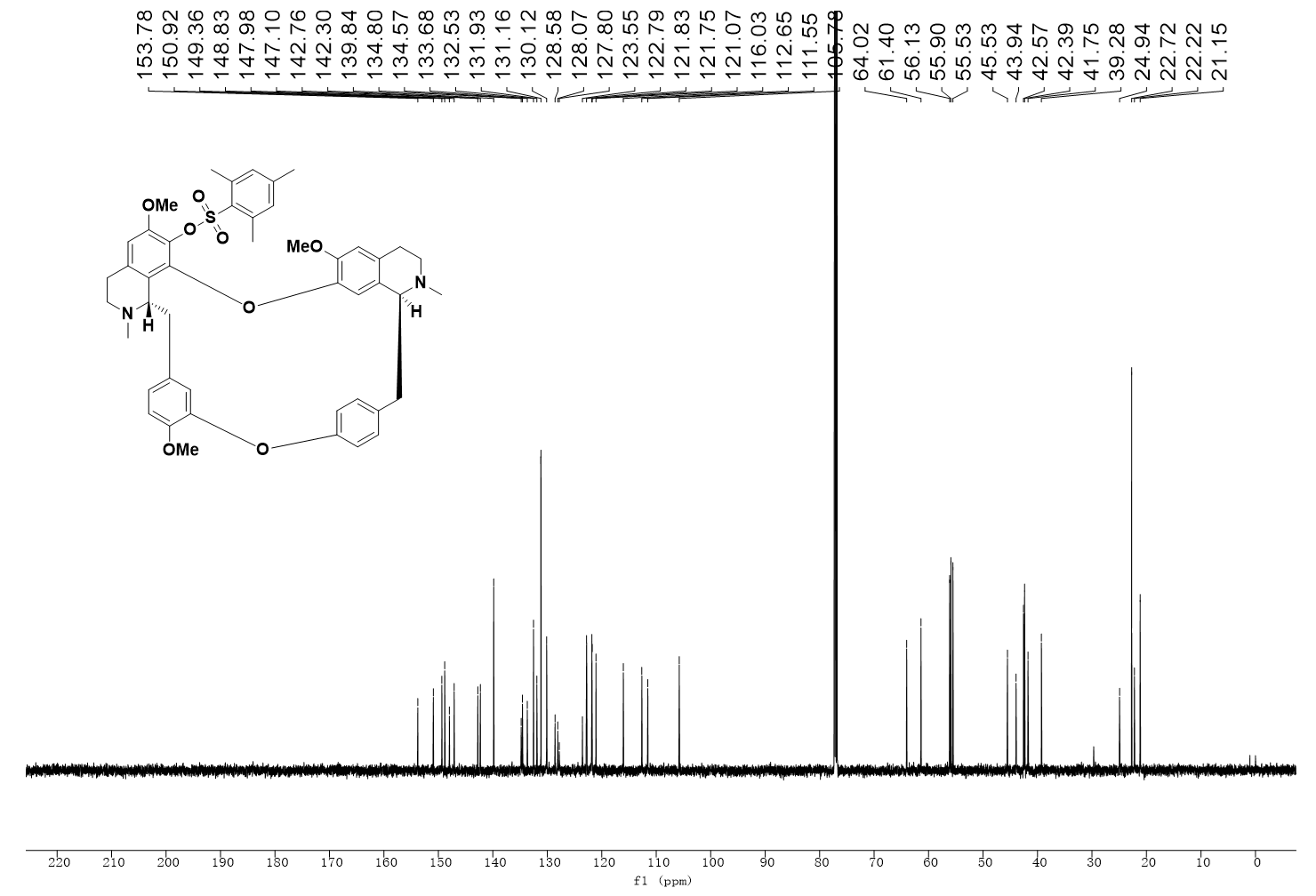


**Compound 3. 7-*O*-(2,4,6-Trimethylbenzenesulfonyl)-tetrandrine: HR-ESIMS spectrum**


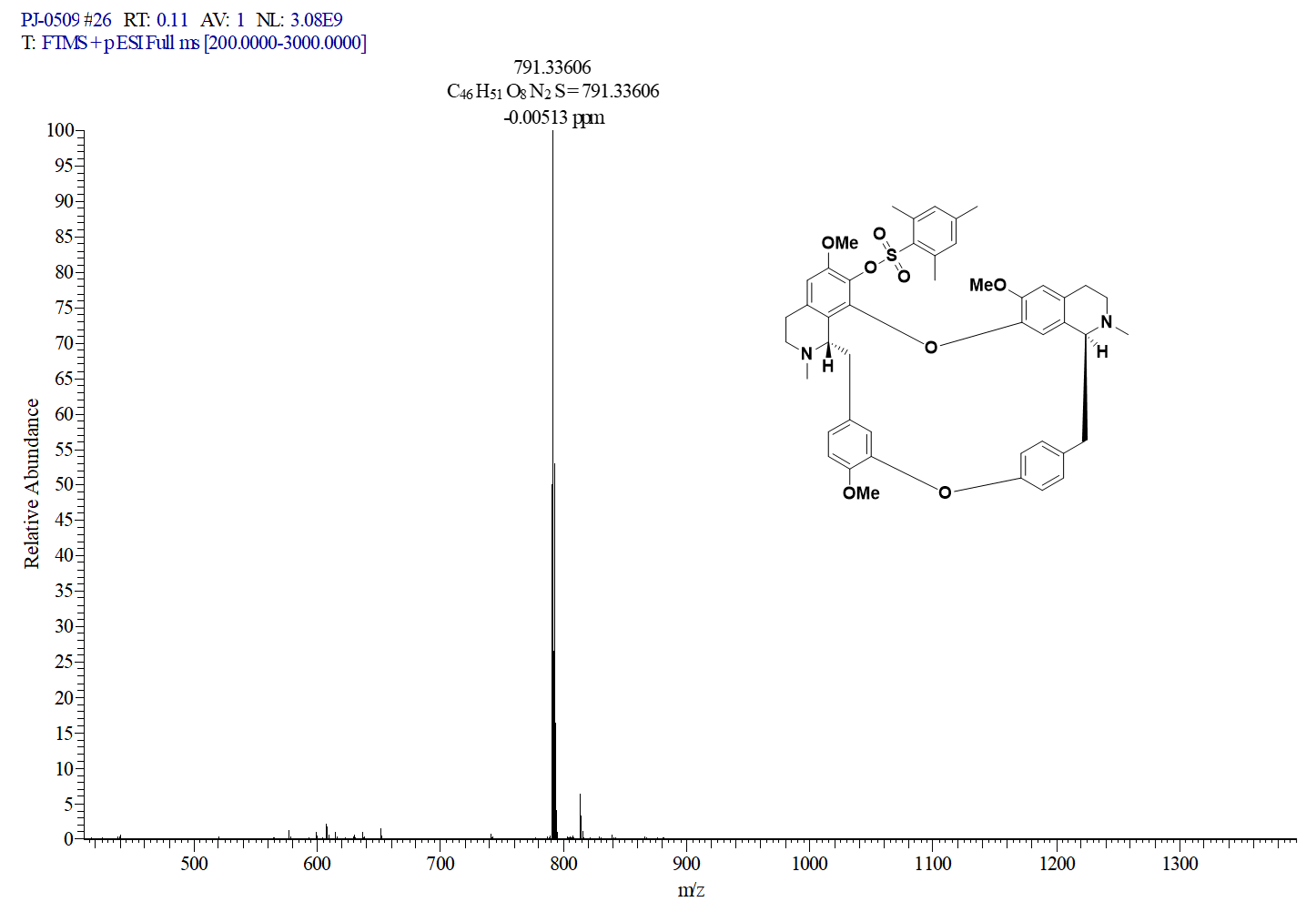


**Compound 4 7-*O*-(2,4,6-Triisopropylbenzenesulfonyl)-tetrandrine: ^1^H-NMR spectrum**


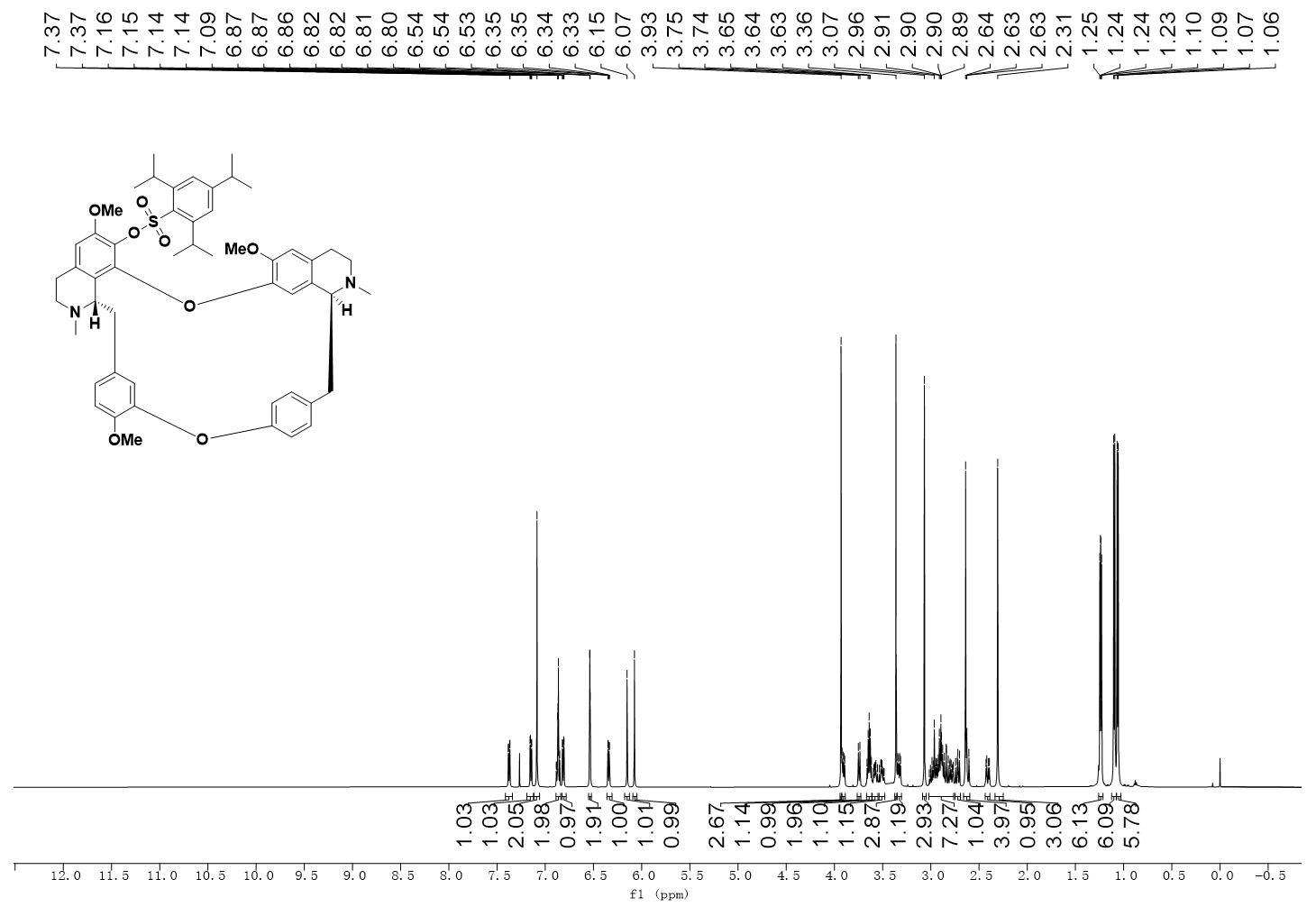


**Compound 4 7-*O*-(2,4,6-Triisopropylbenzenesulfonyl)-tetrandrine: ^13^C-NMR spectrum**


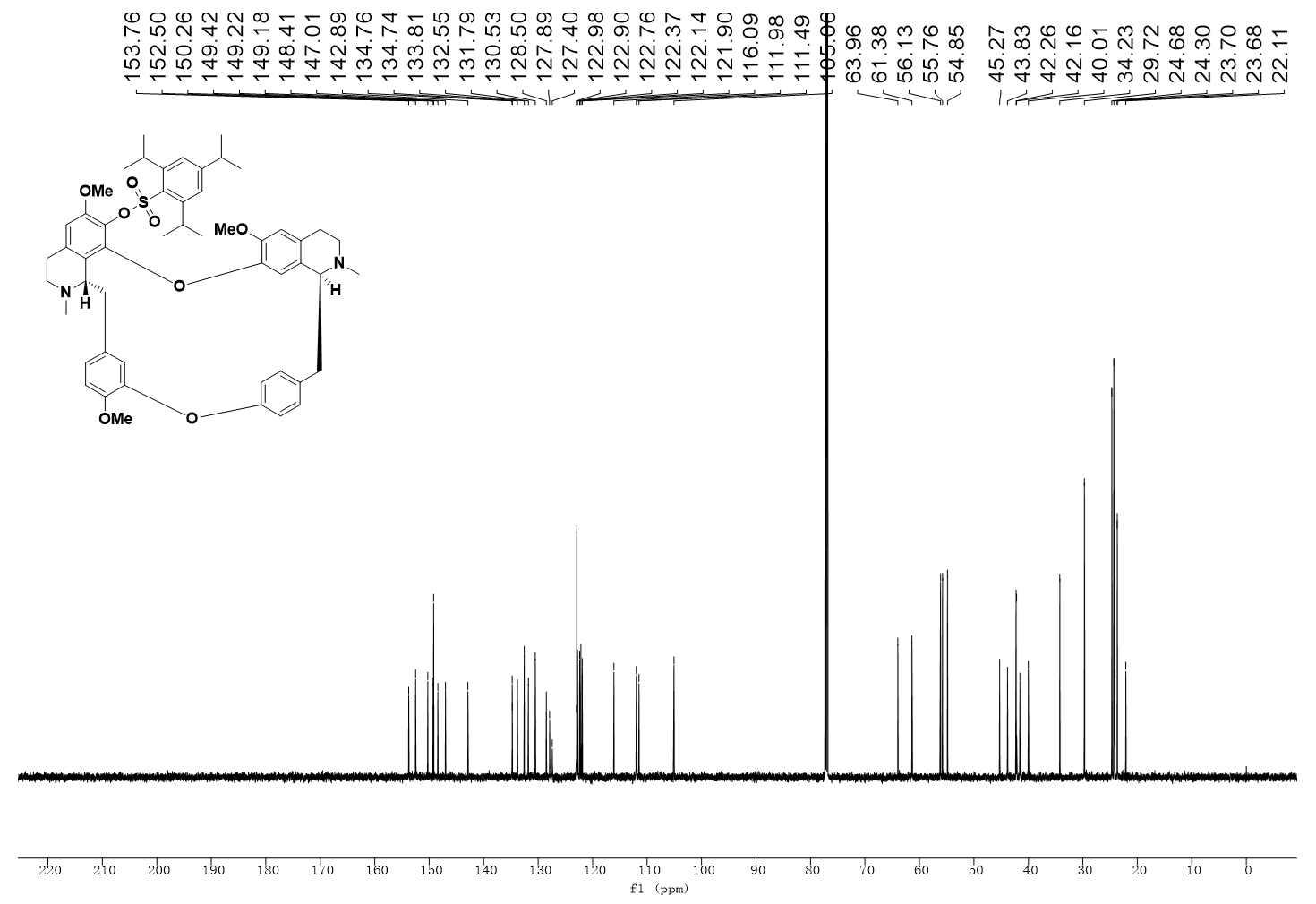


**Compound 4 7-*O*-(2,4,6-Triisopropylbenzenesulfonyl)-tetrandrine: HR-ESIMS spectrum**


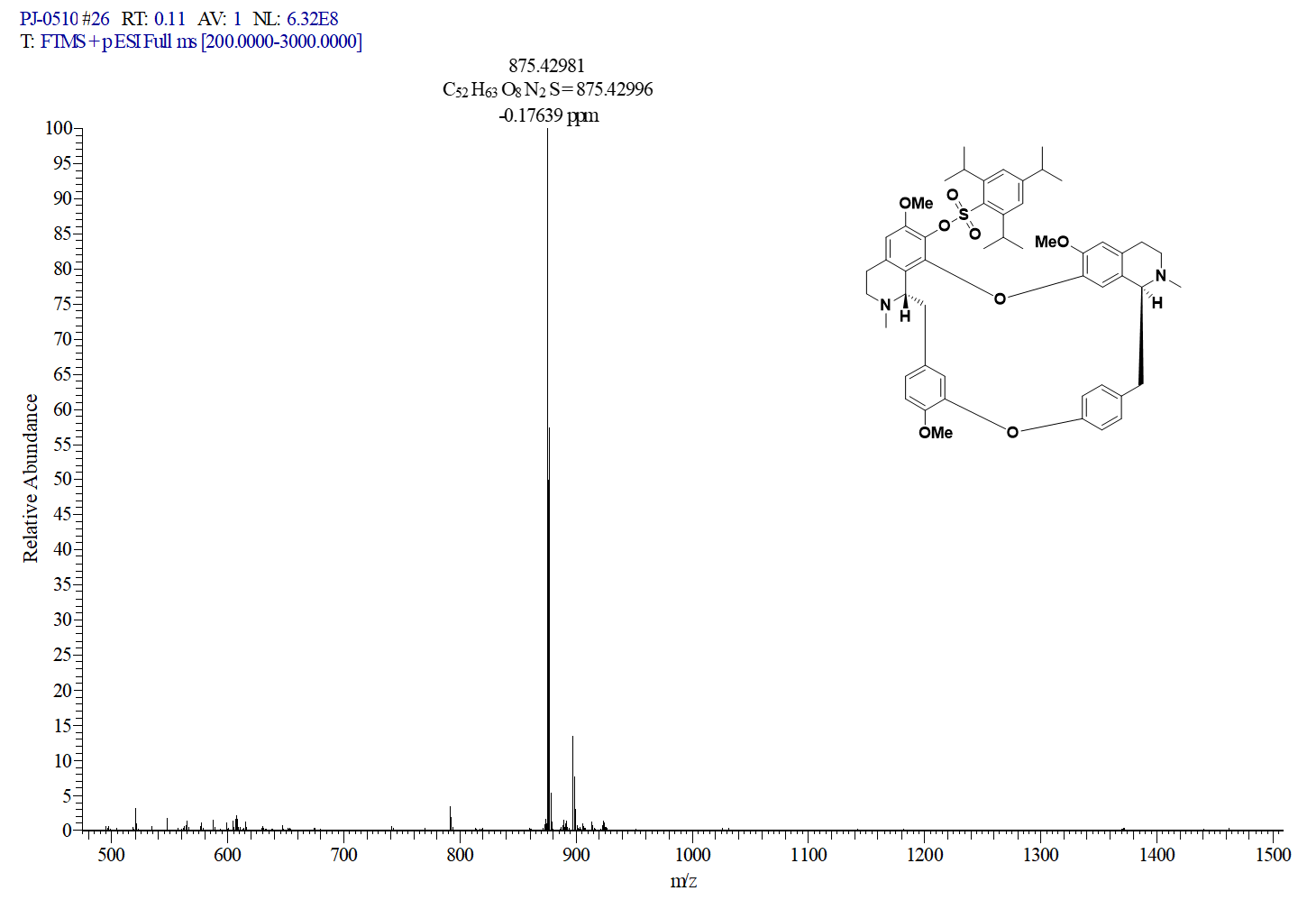


**Compound 5. 7-*O*-(2-(Trifluoromethyl)benzenesulfonyl)-tetrandrine: ^1^H-NMR spectrum**


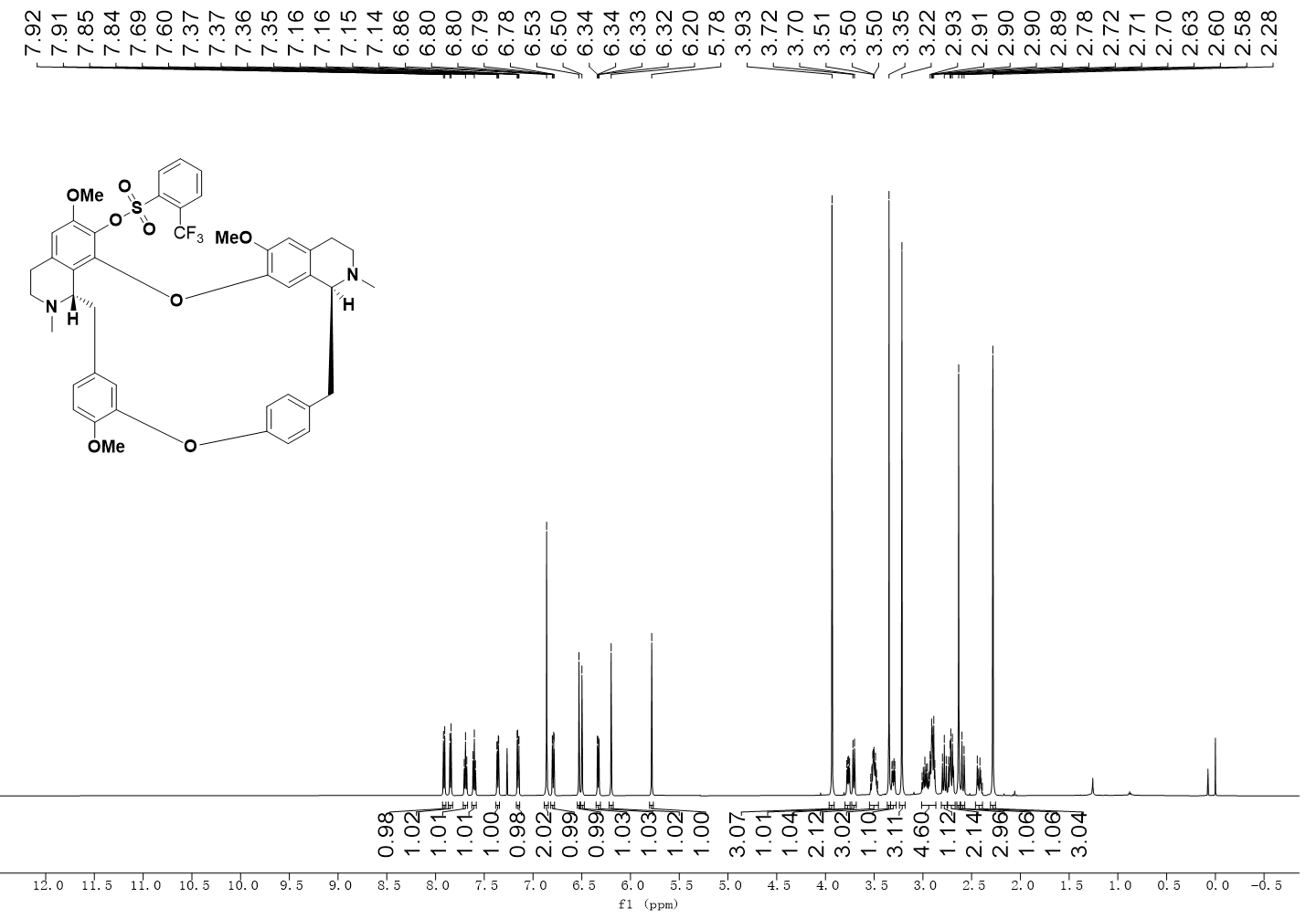


**Compound 5. 7-*O*-(2-(Trifluoromethyl)benzenesulfonyl)-tetrandrine: ^13^C-NMR spectrum**


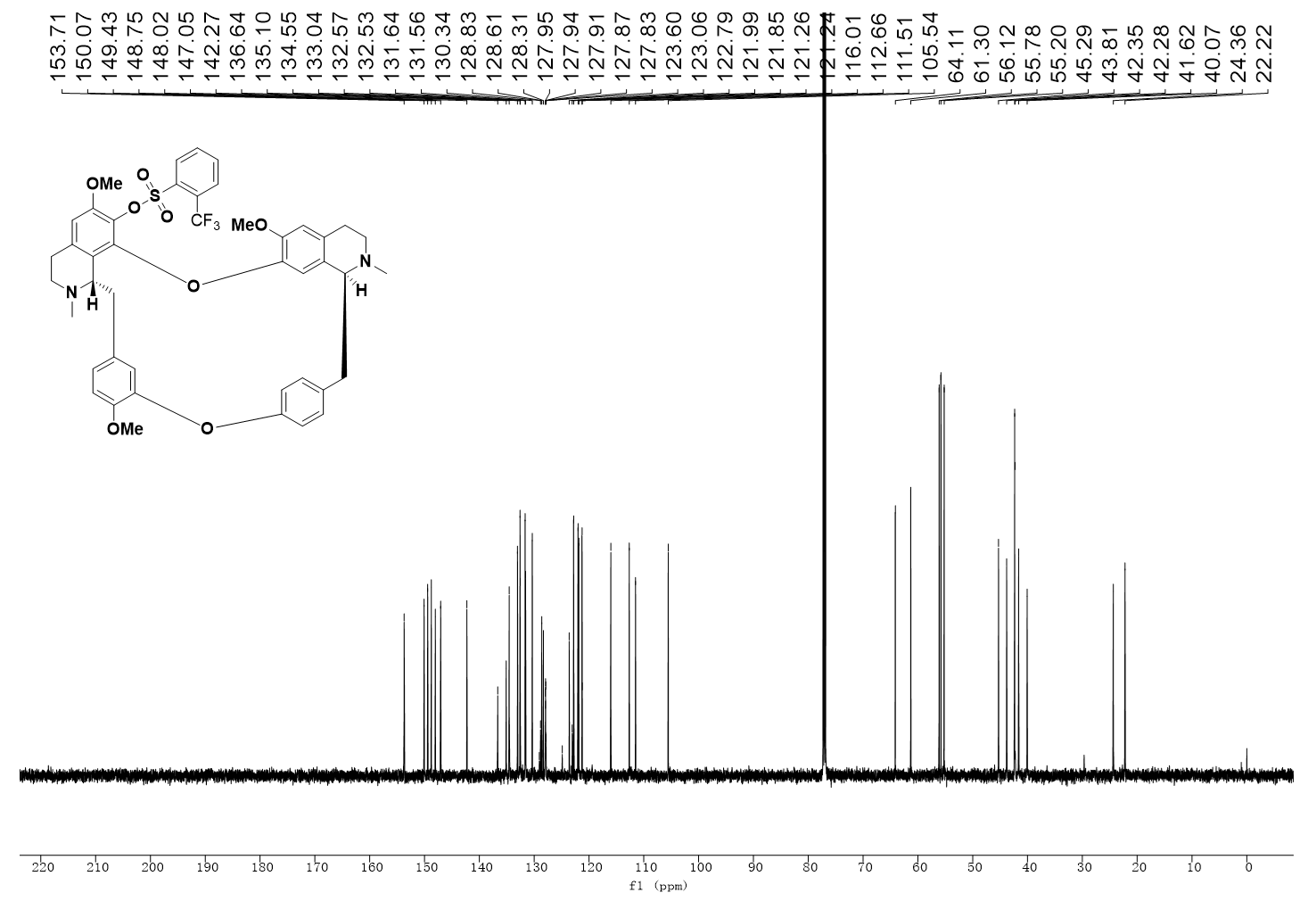


**Compound 5. 7-*O*-(2-(Trifluoromethyl)benzenesulfonyl)-tetrandrine:** **^19^F-NMR spectrum**


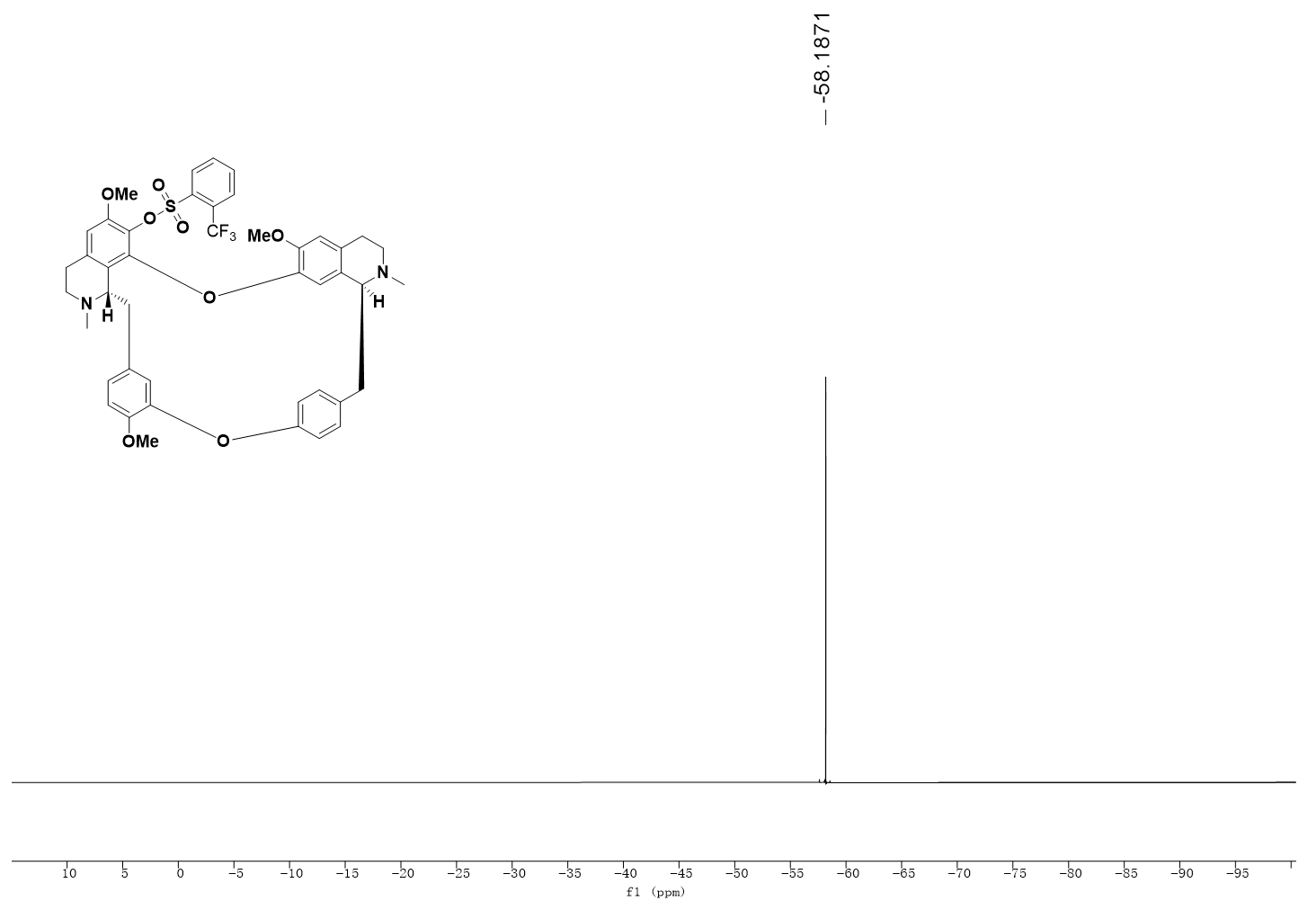


**Compound 5. 7-*O*-(2-(Trifluoromethyl)benzenesulfonyl)-tetrandrine: HR-ESIMS spectrum**


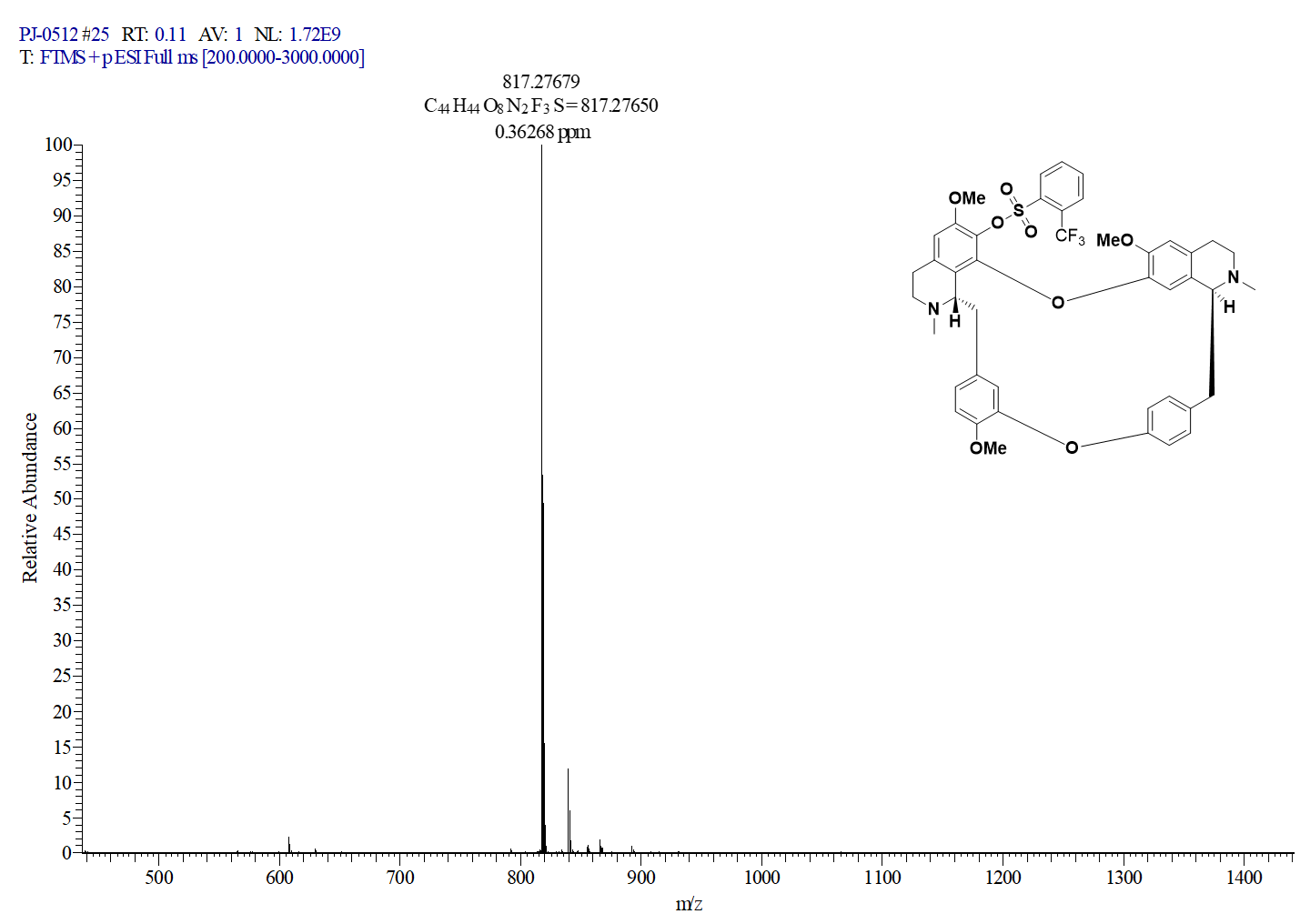


**Compound 6. 7-*O*-(4-(Trifluoromethoxy)benzenesulfonyl)-tetrandrine: ^1^H-NMR spectrum**


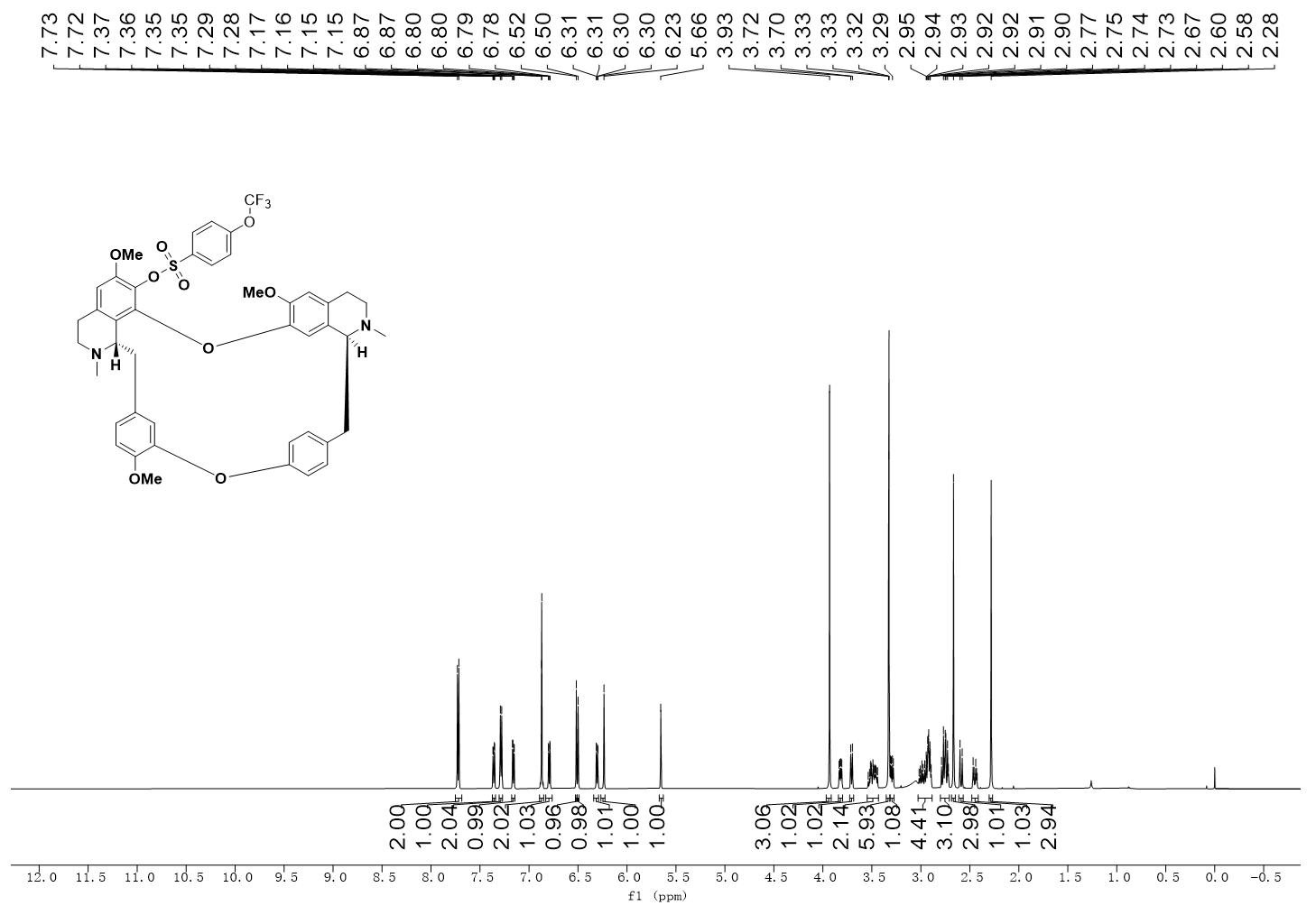


**Compound 6. 7-*O*-(4-(Trifluoromethoxy)benzenesulfonyl)-tetrandrine: ^13^C-NMR spectrum**


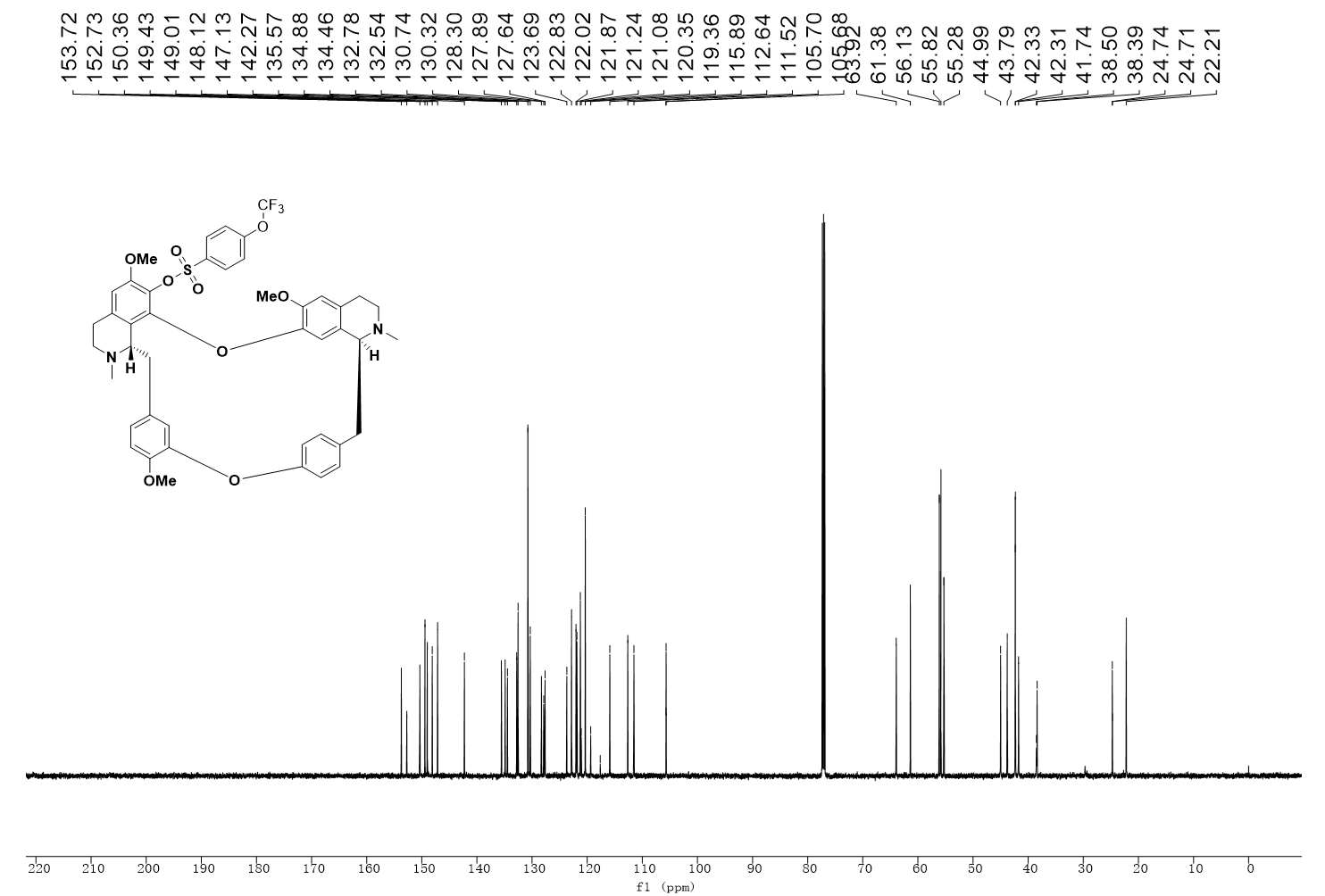


**Compound 6. 7-*O*-(4-(Trifluoromethoxy)benzenesulfonyl)-tetrandrine: ^19^F-NMR spectrum**


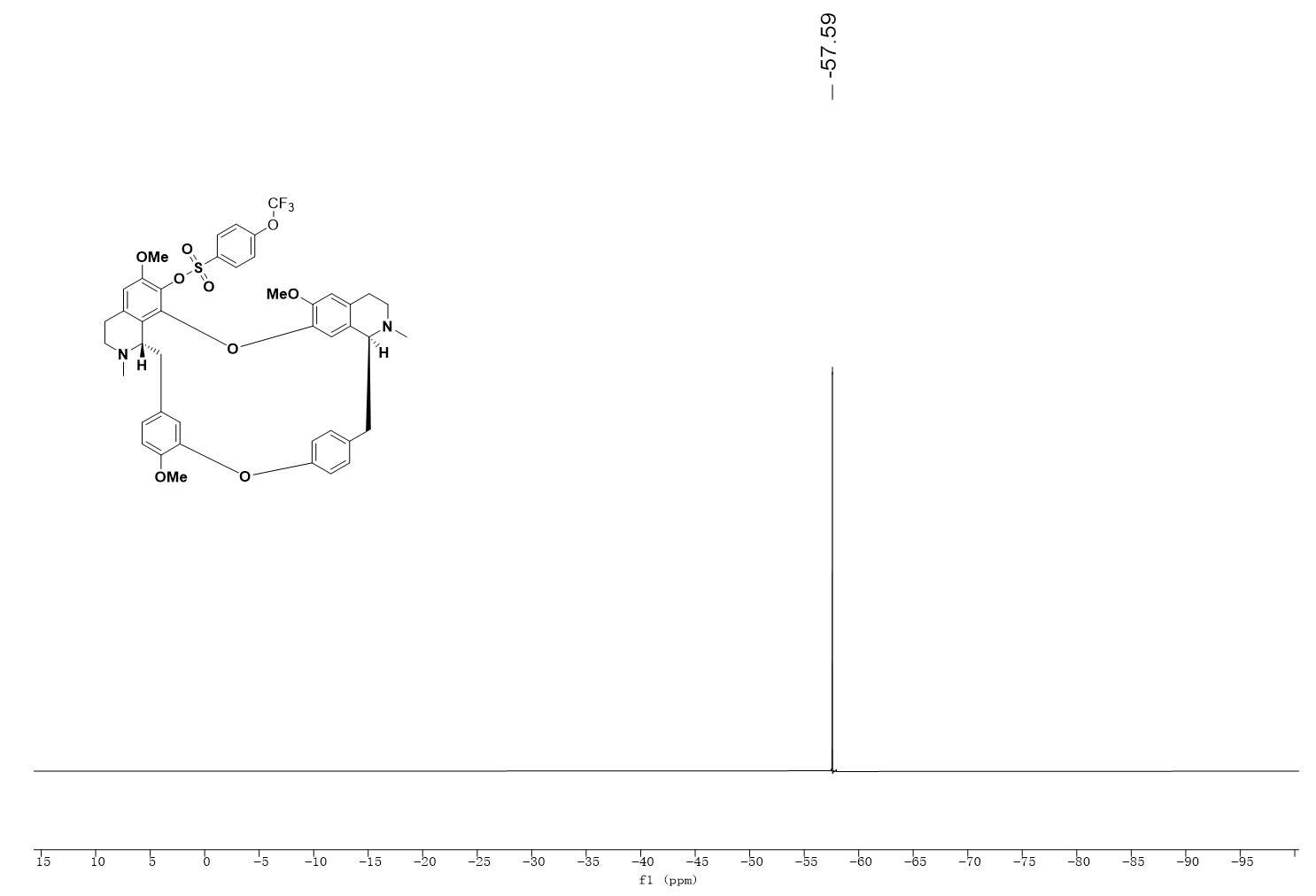


**Compound 6. 7-*O*-(4-(Trifluoromethoxy)benzenesulfonyl)-tetrandrine: HR-ESIMS spectrum**


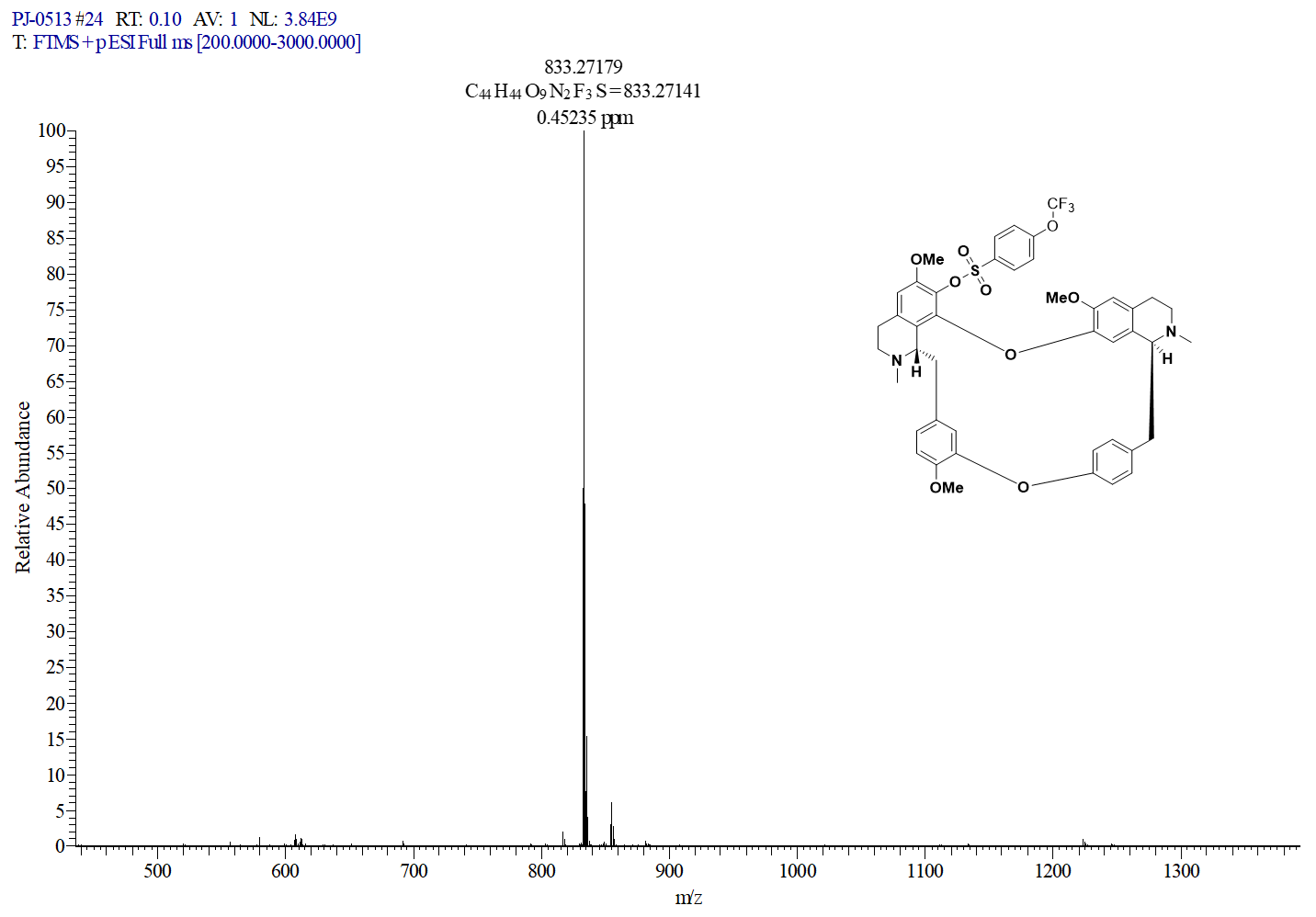


**Compound 7.** **7-*O*-(1-Butanesulfonyl)-tetrandrine: ^1^H-NMR spectrum**


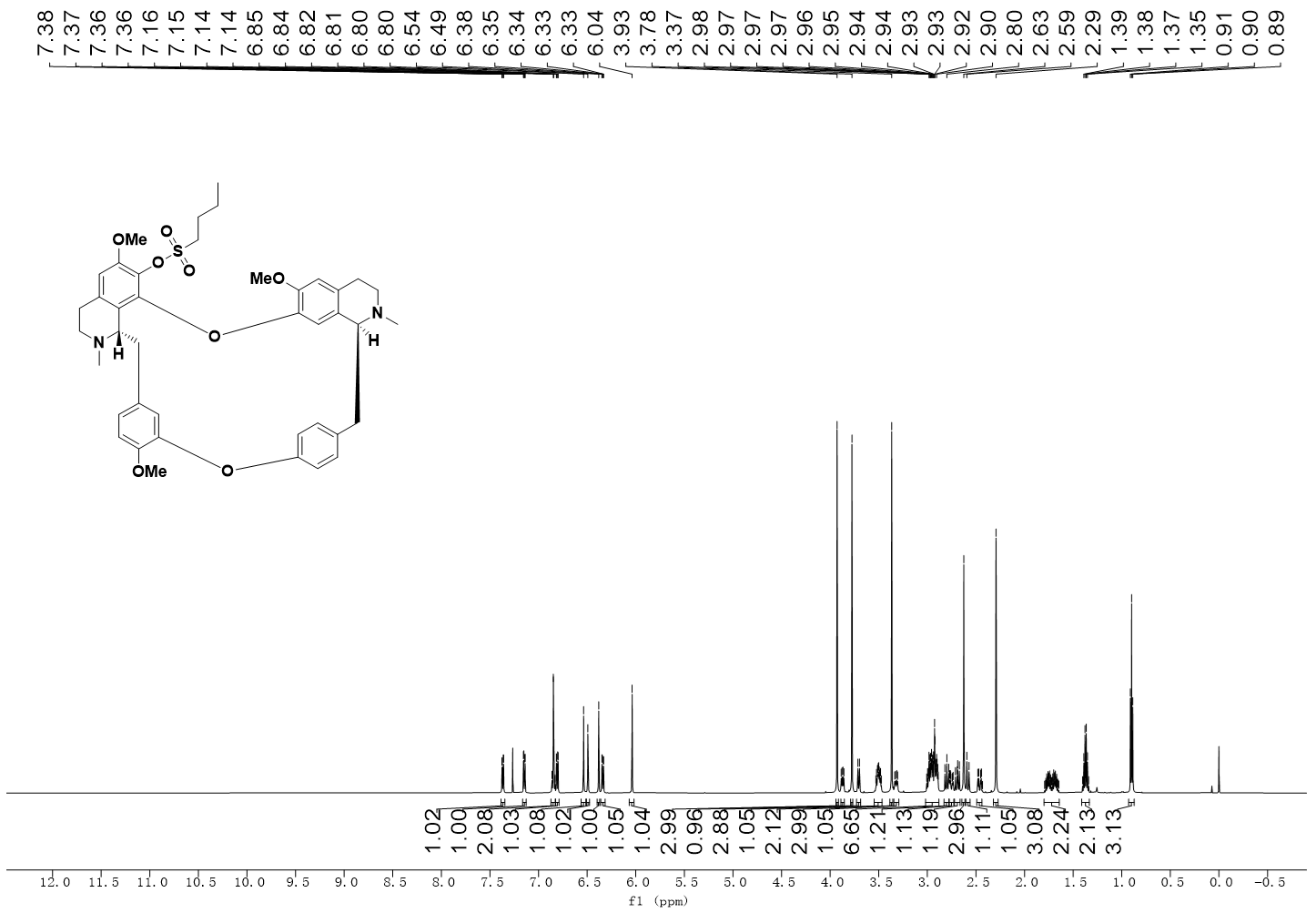


**Compound 7. 7-*O*-(1-Butanesulfonyl)-tetrandrine: ^13^C-NMR spectrum**


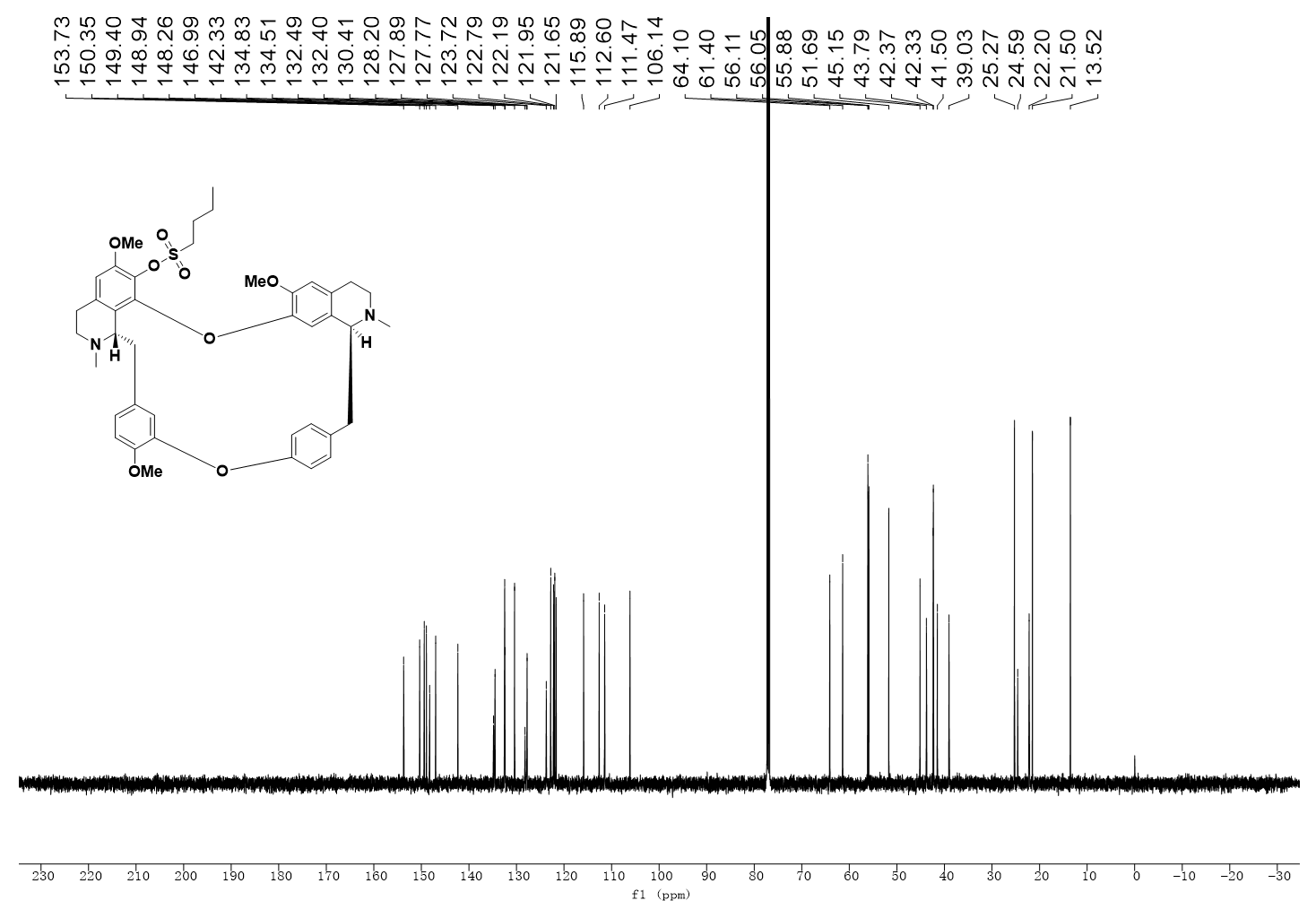


**Compound 7. 7-*O*-(1-Butanesulfonyl)-tetrandrine: HR-ESIMS spectrum**


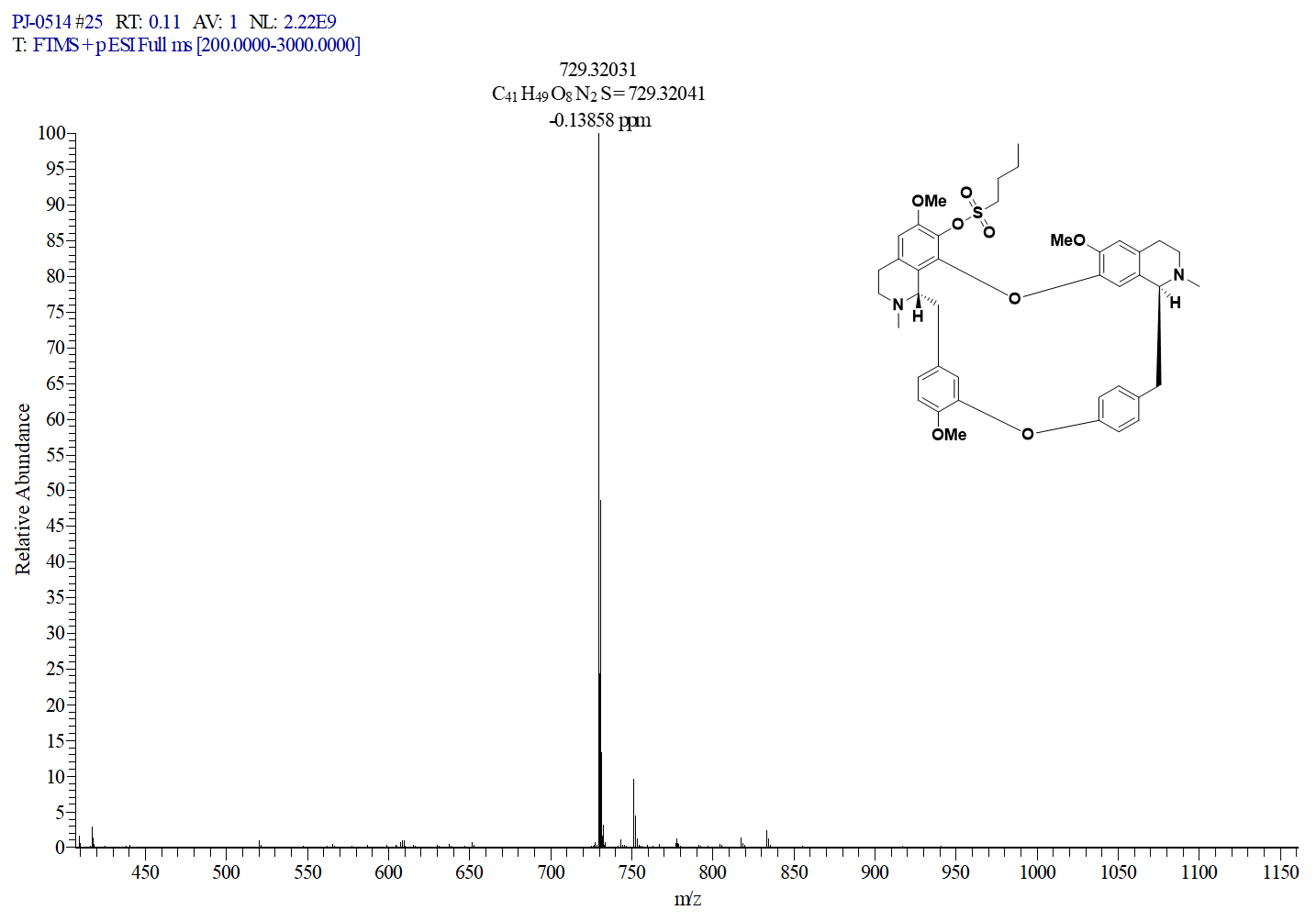


**Compound 8.** **7-*O*-(4-tert-Butylbenzenesulfonyl)-tetrandrine: ^1^H-NMR spectrum**


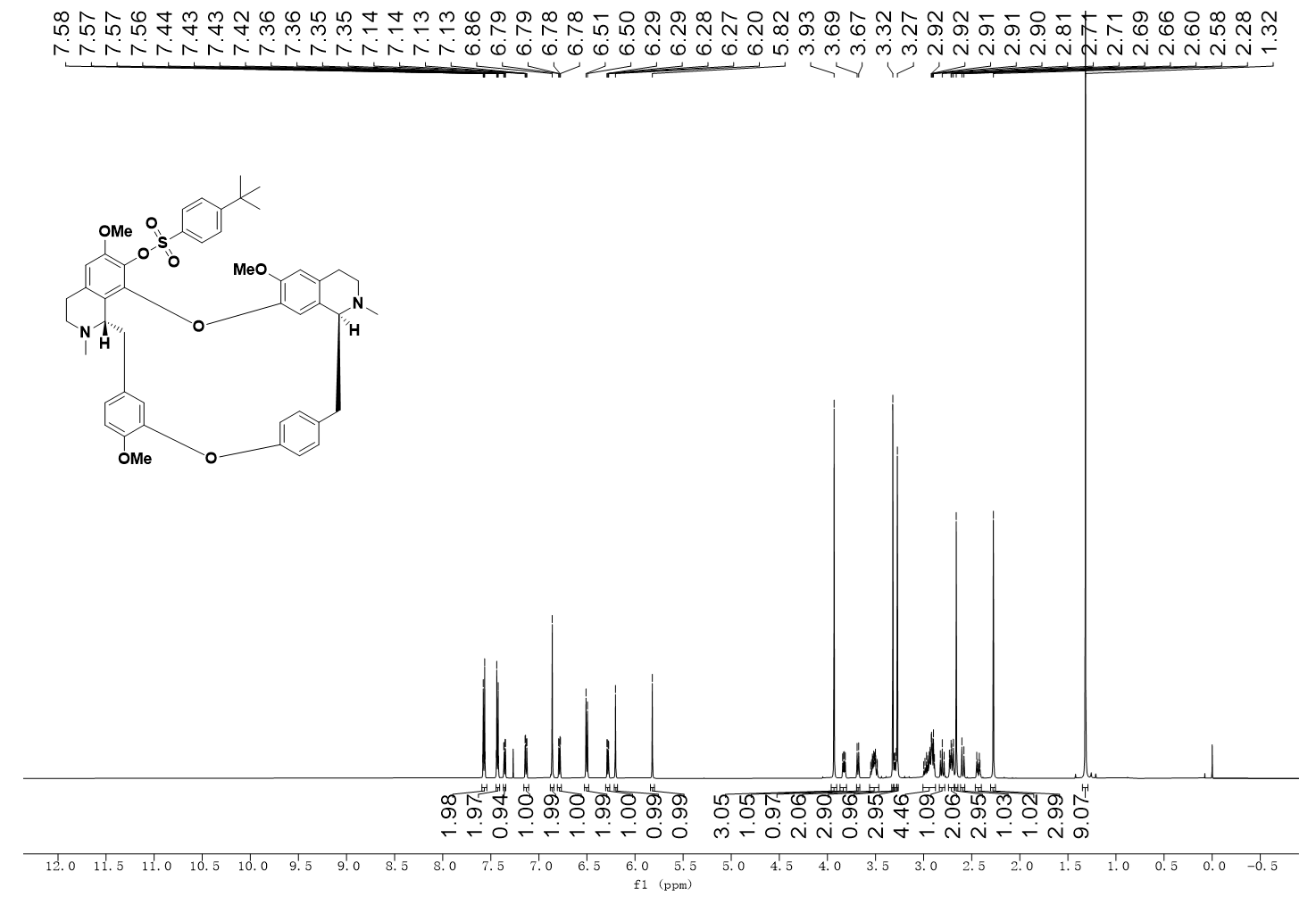


**Compound 8. 7-*O*-(4-tert-Butylbenzenesulfonyl)-tetrandrine: ^13^C-NMR spectrum**


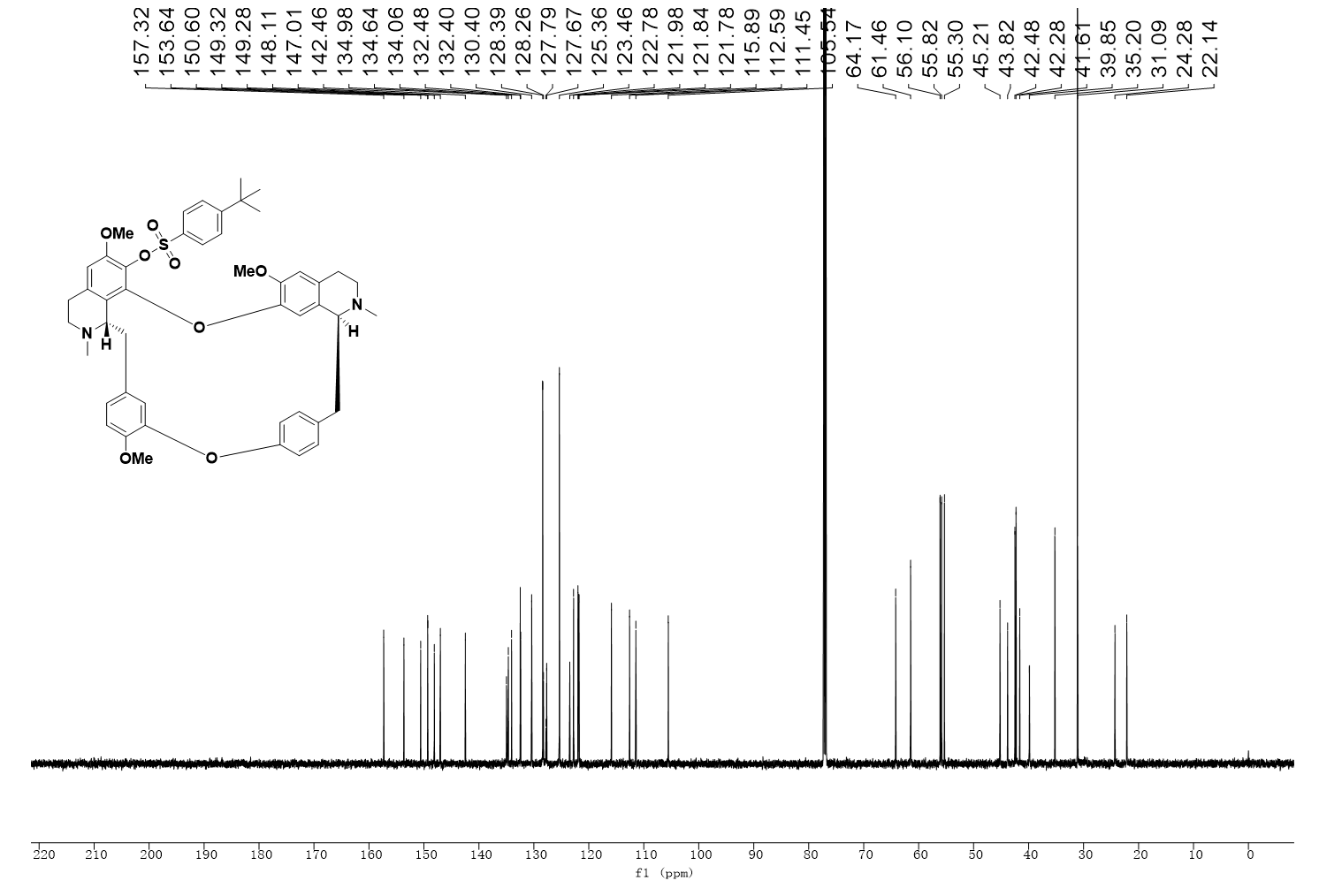


**Compound 8. 7-*O*-(4-tert-Butylbenzenesulfonyl)-tetrandrine: HR-ESIMS spectrum**


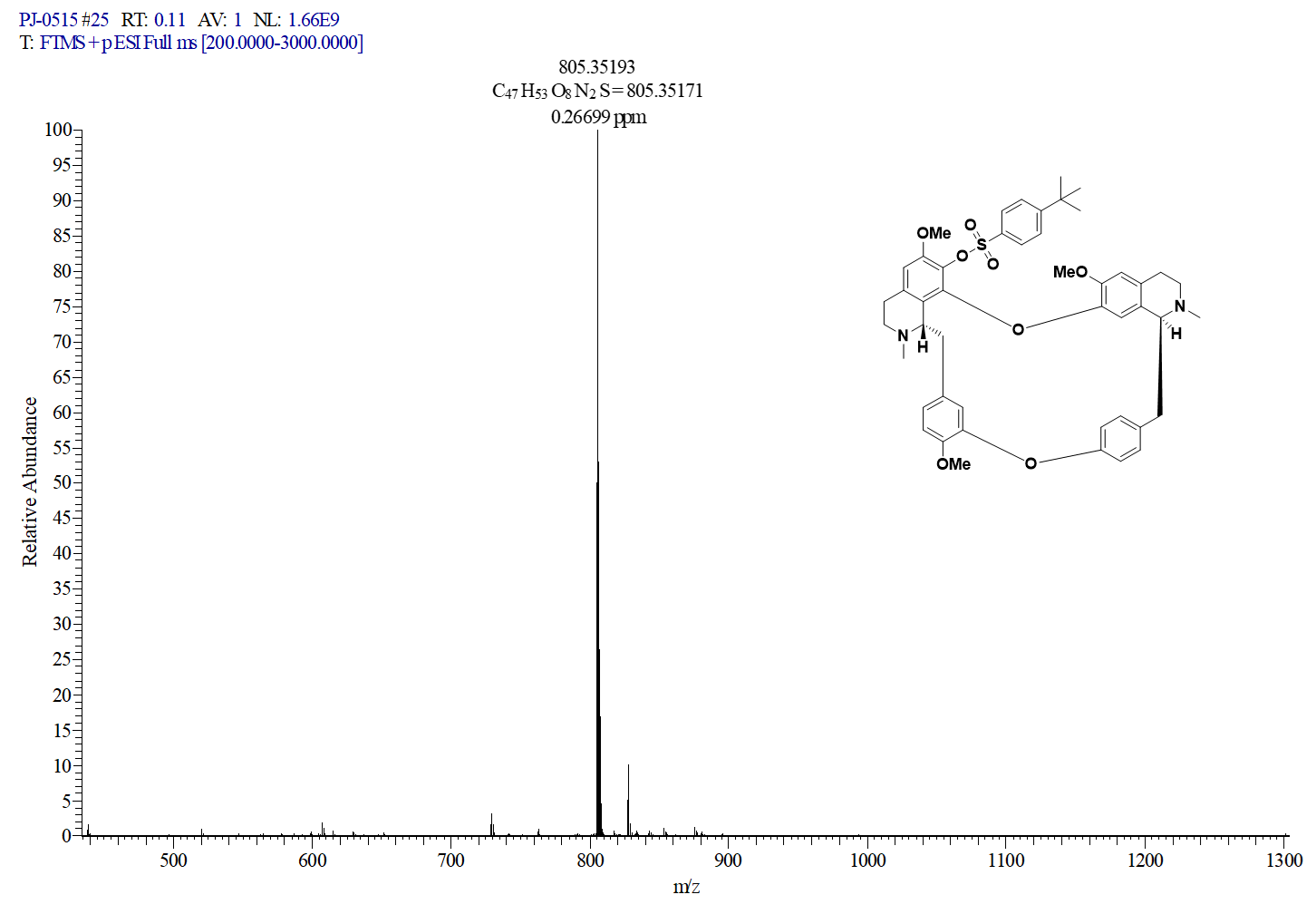


**Compound 9.** **7-*O*-(3-Nitrobenzenesulfonyl)-tetrandrine: ^1^H-NMR spectrum**


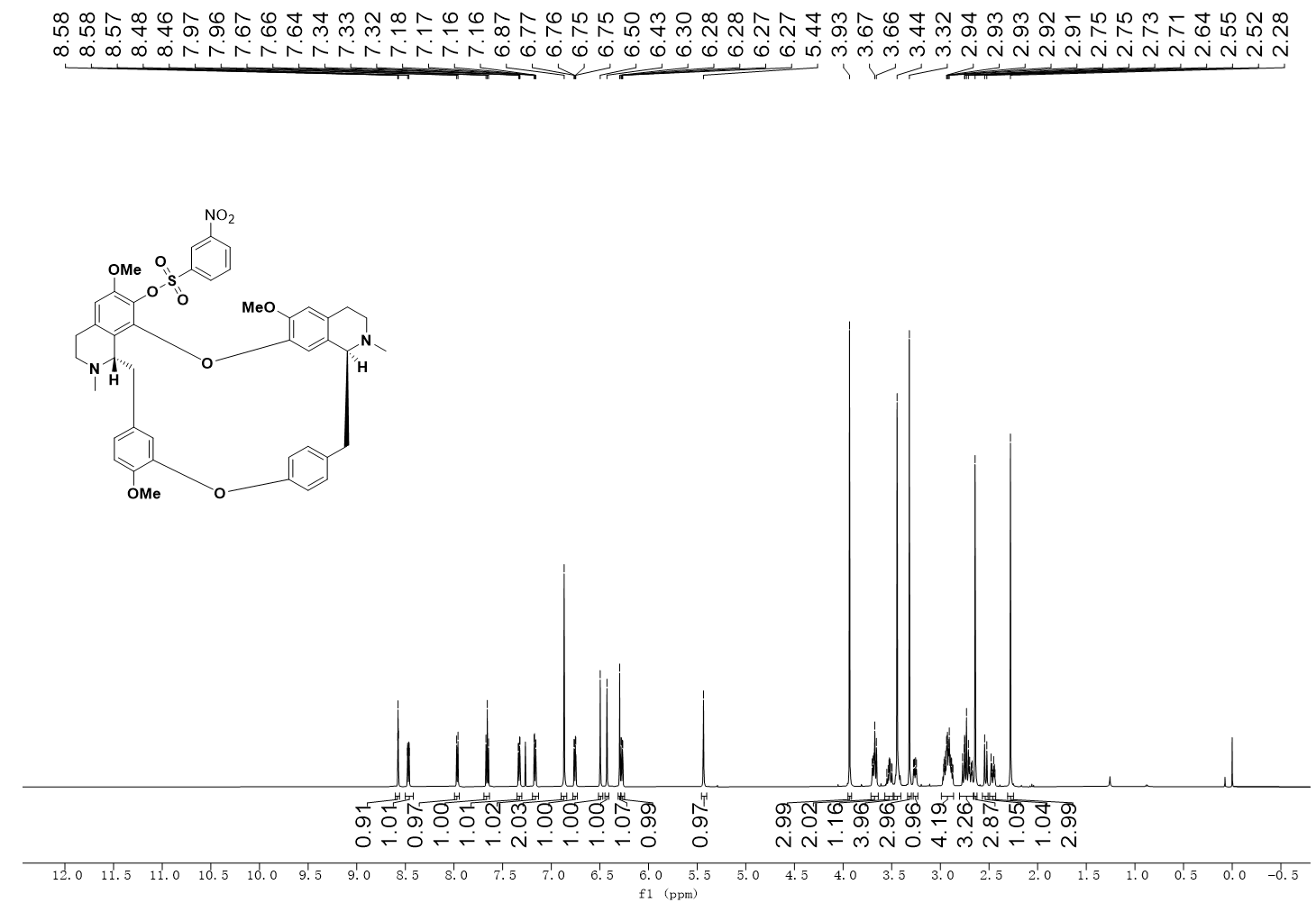


**Compound 9. 7-*O*-(3-Nitrobenzenesulfonyl)-tetrandrine: ^13^C-NMR spectrum**


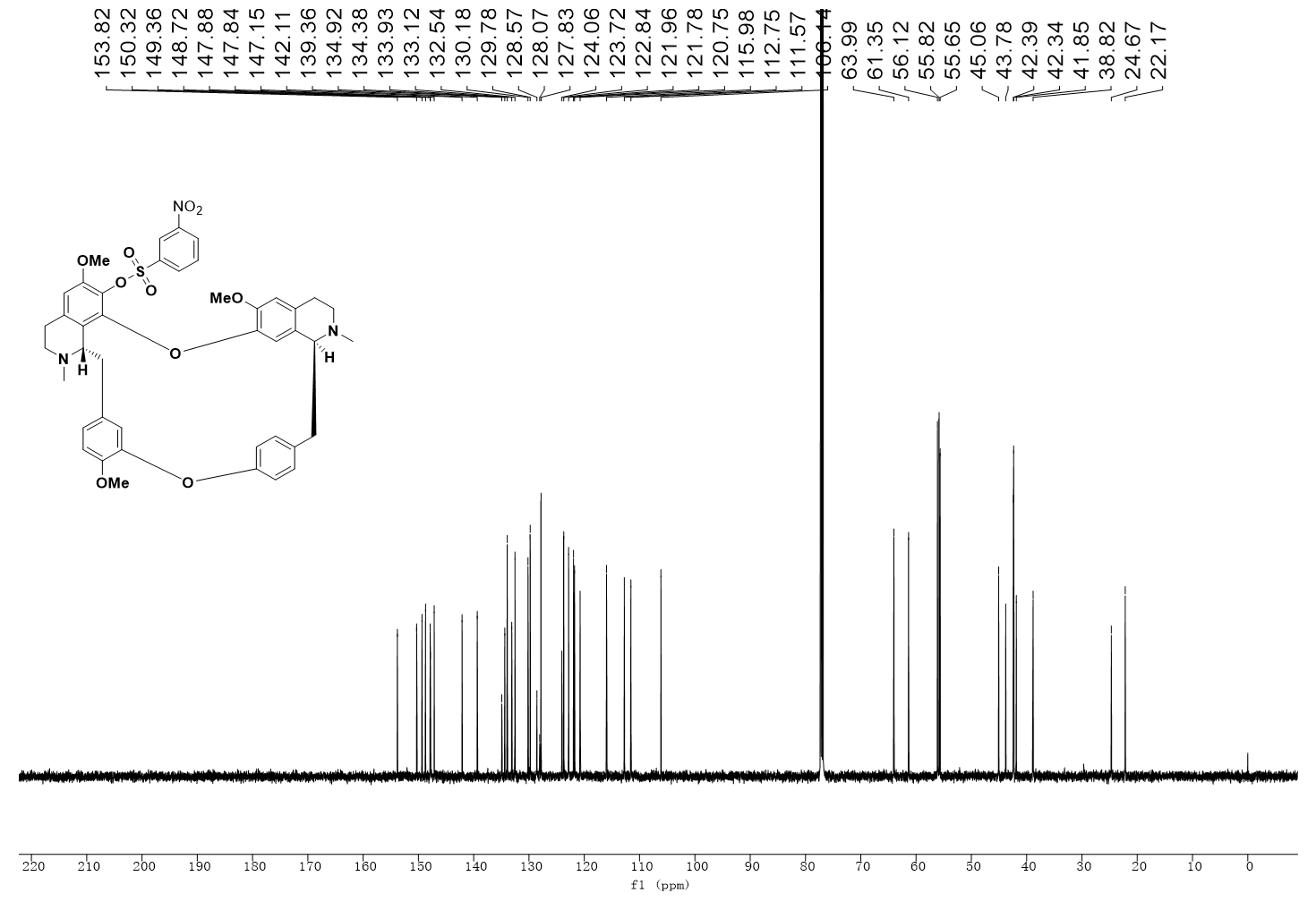


**Compound 9. 7-*O*-(3-Nitrobenzenesulfonyl)-tetrandrine: HR-ESIMS spectrum**


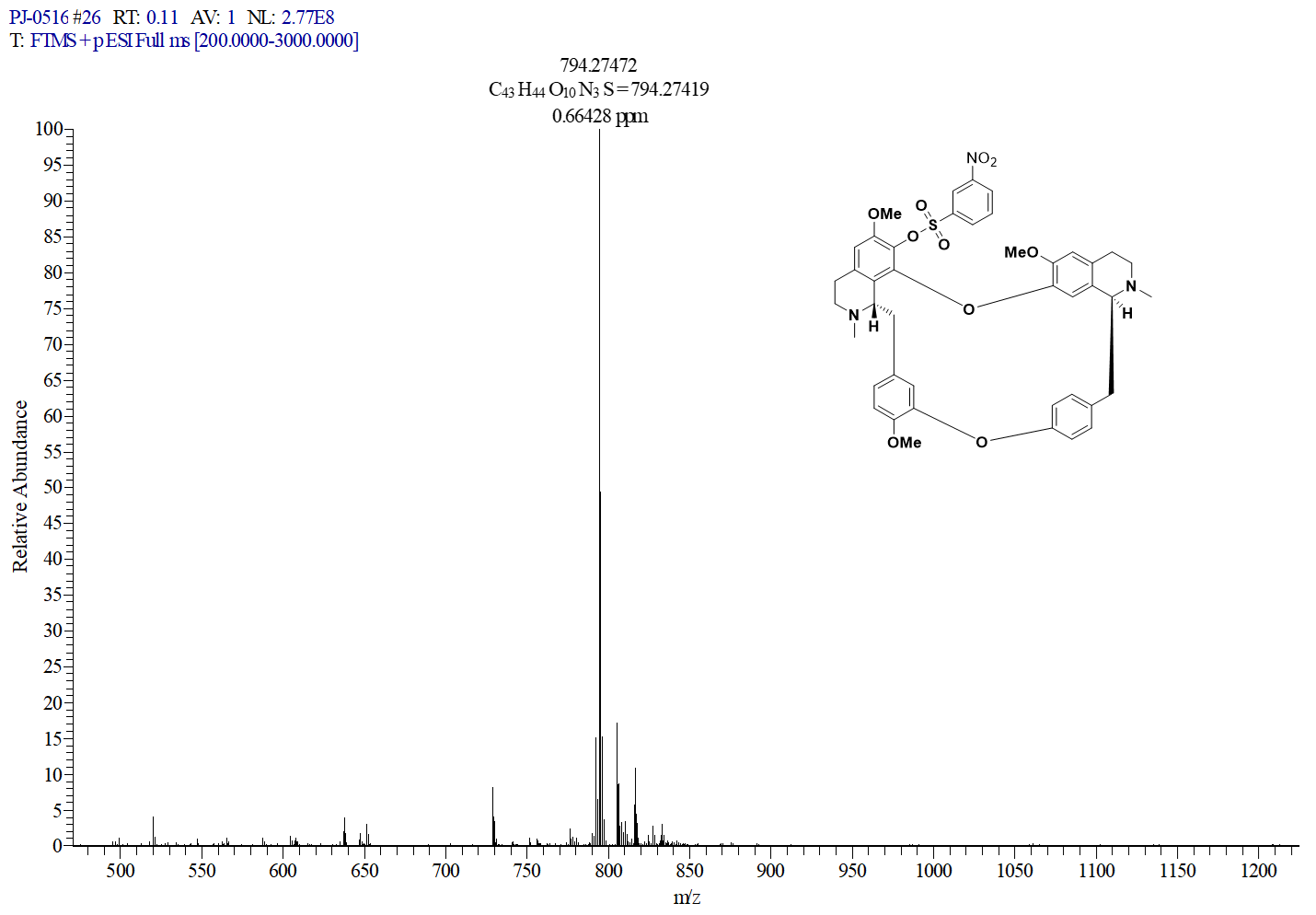


**Compound 10****.** **7-*O*-(3-(Trifluoromethyl)benzenesulfonyl)-tetrandrine: ^1^H-NMR spectrum**


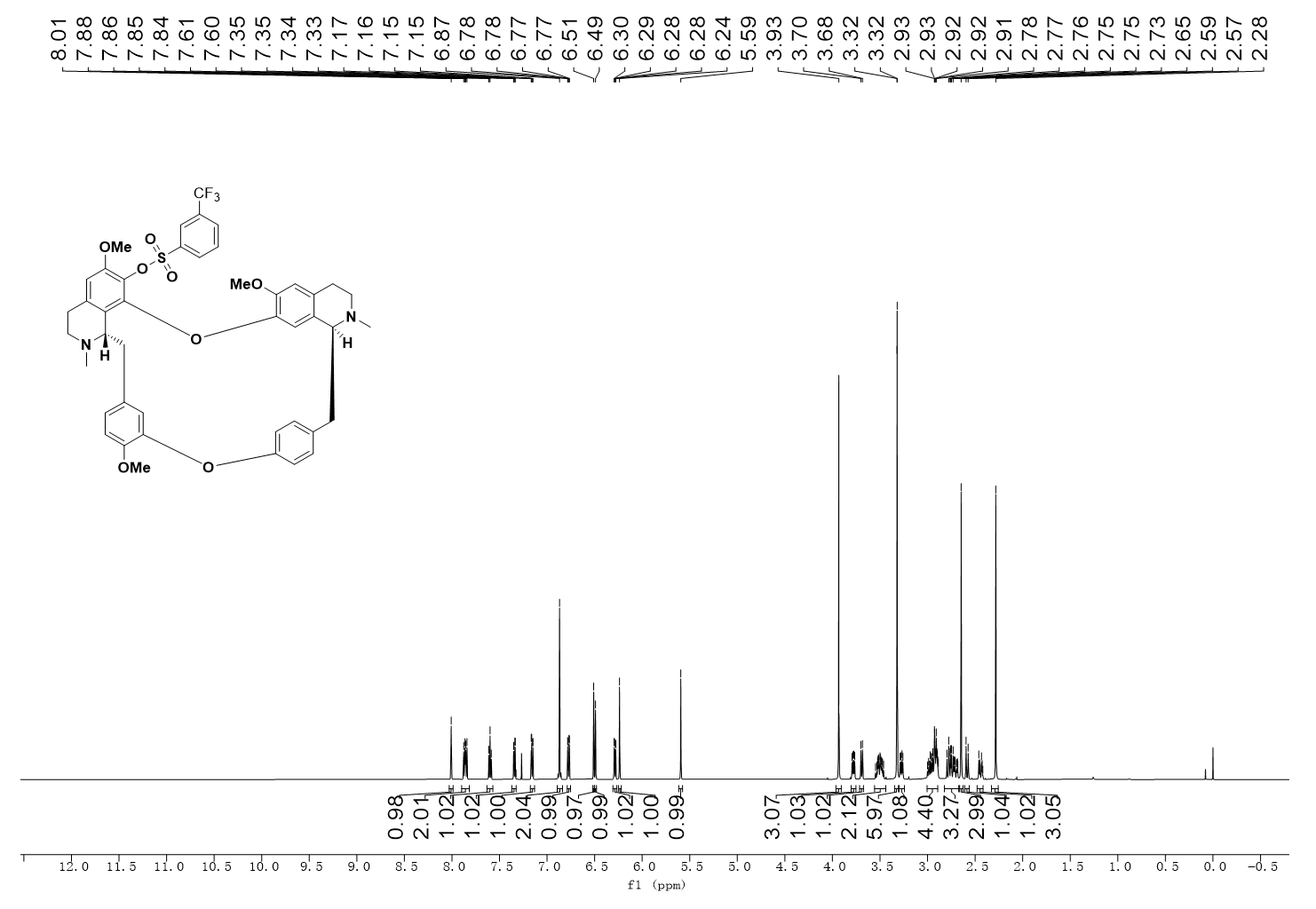


**Compound 10. 7-*O*-(3-(Trifluoromethyl)benzenesulfonyl)-tetrandrine: ^13^C-NMR spectrum**


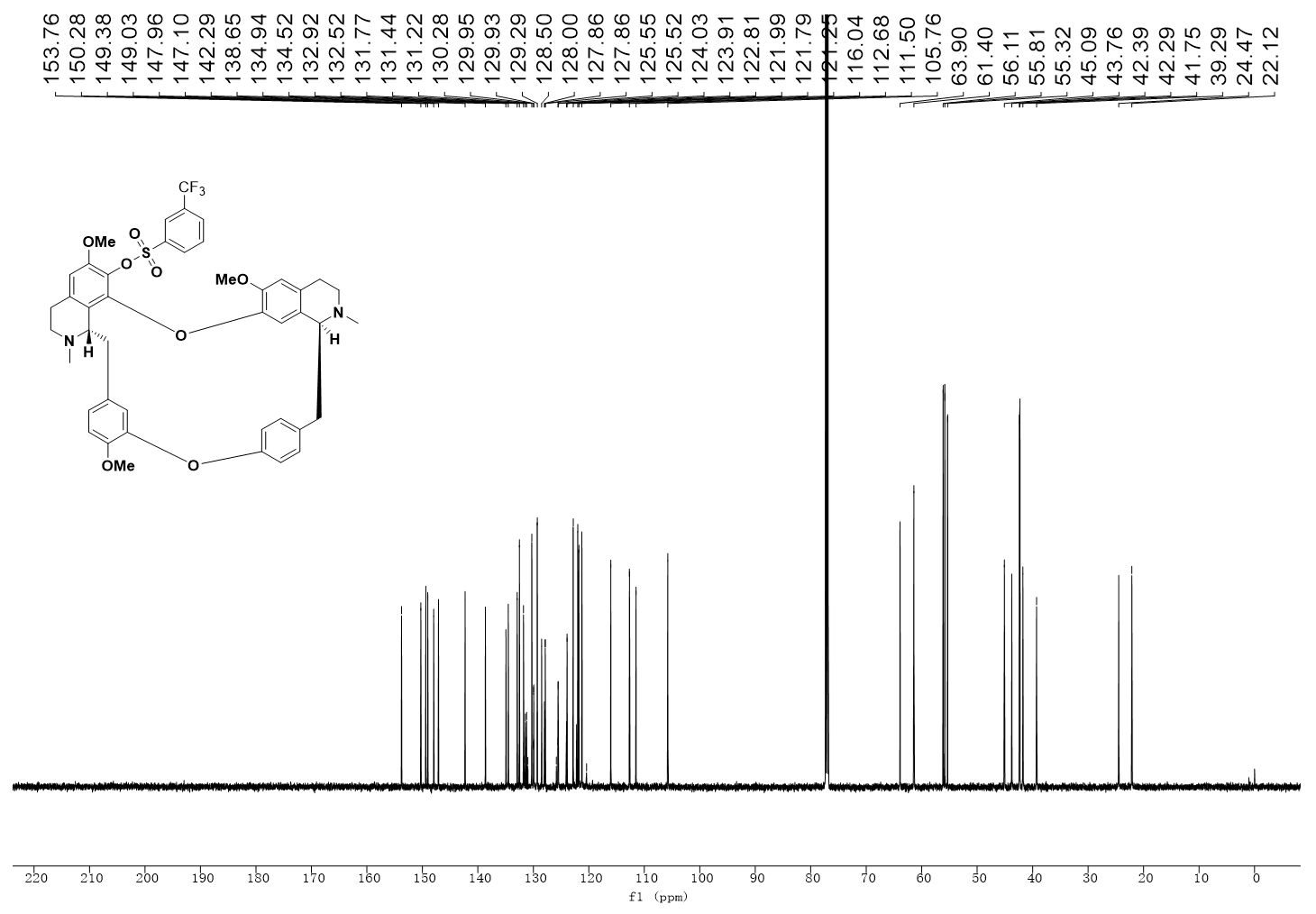


**Compound 10. 7-*O*-(3-(Trifluoromethyl)benzenesulfonyl)-tetrandrine: ^19^F-NMR spectrum**


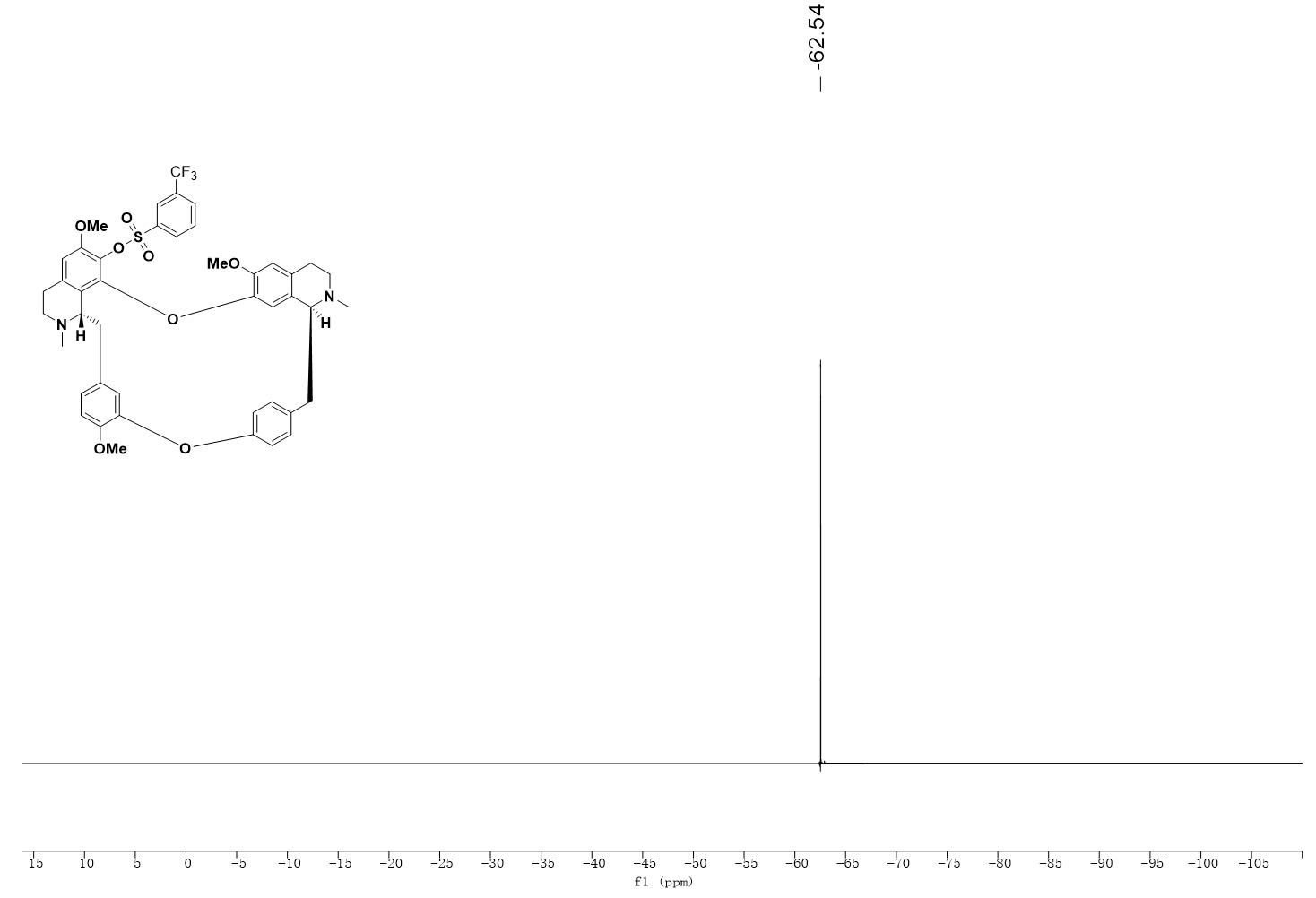


**Compound 10. 7-*O*-(3-(Trifluoromethyl)benzenesulfonyl)-tetrandrine: HR-ESIMS spectrum**


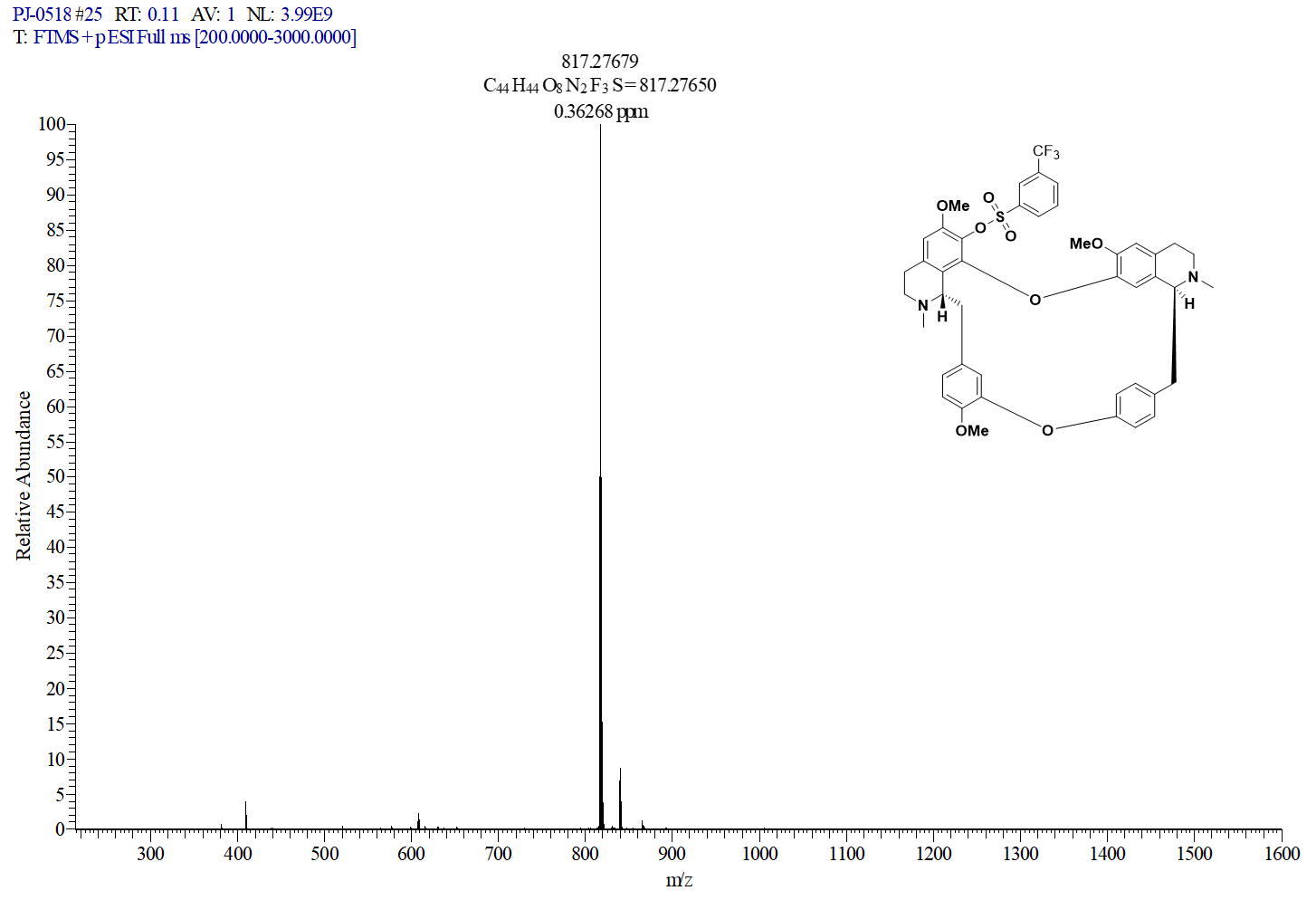


**Compound 11****. 7-*O*-(2-Fluorobenzenesulfonyl)-tetrandrine: ^1^H-NMR spectrum**


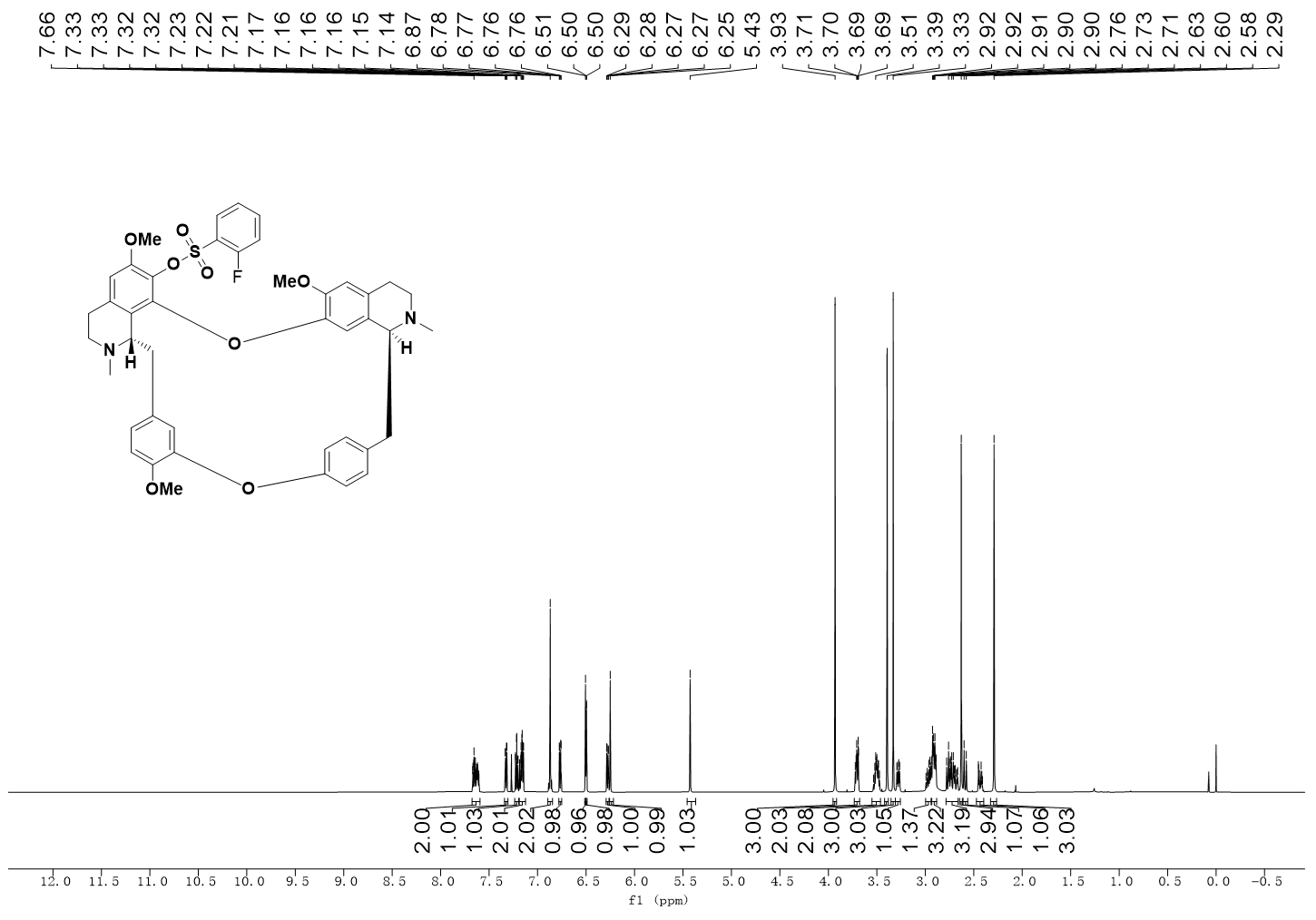


**Compound 11. 7-*O*-(2-Fluorobenzenesulfonyl)-tetrandrine: ^13^C-NMR spectrum**


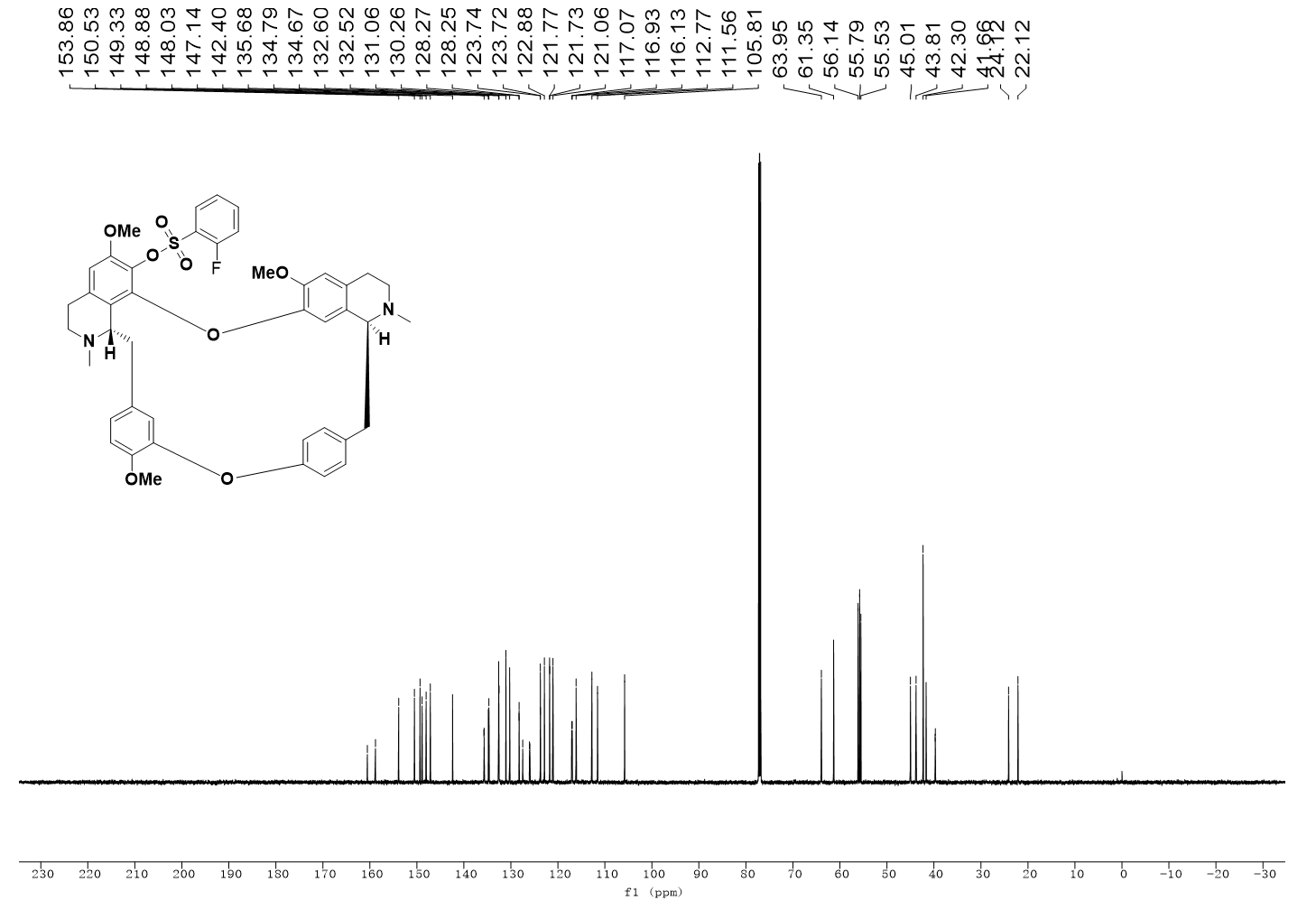


**Compound 11. 7-*O*-(2-Fluorobenzenesulfonyl)-tetrandrine: ^19^F-NMR spectrum**


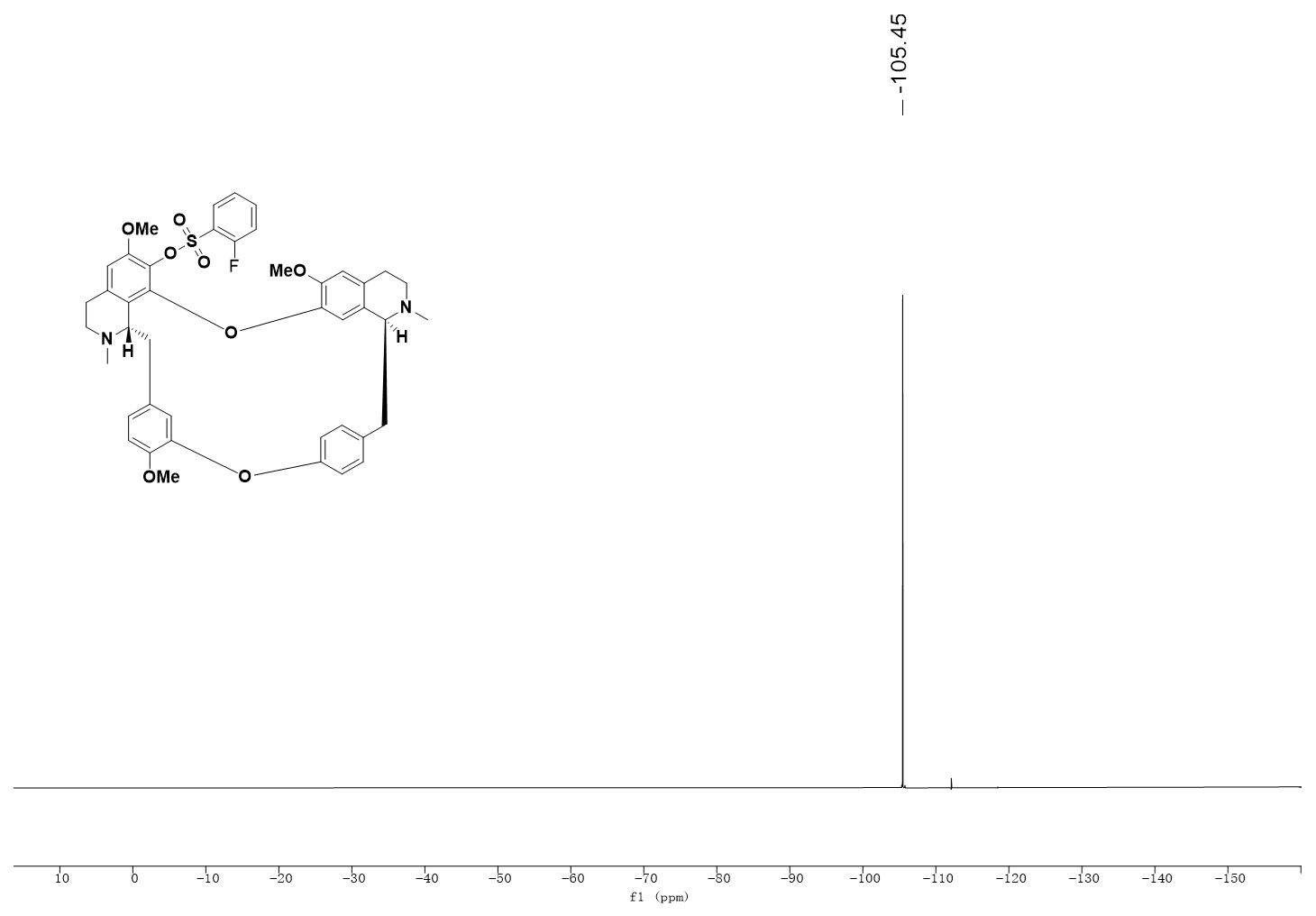


**Compound 11. 7-*O*-(2-Fluorobenzenesulfonyl)-tetrandrine: HR-ESIMS spectrum**


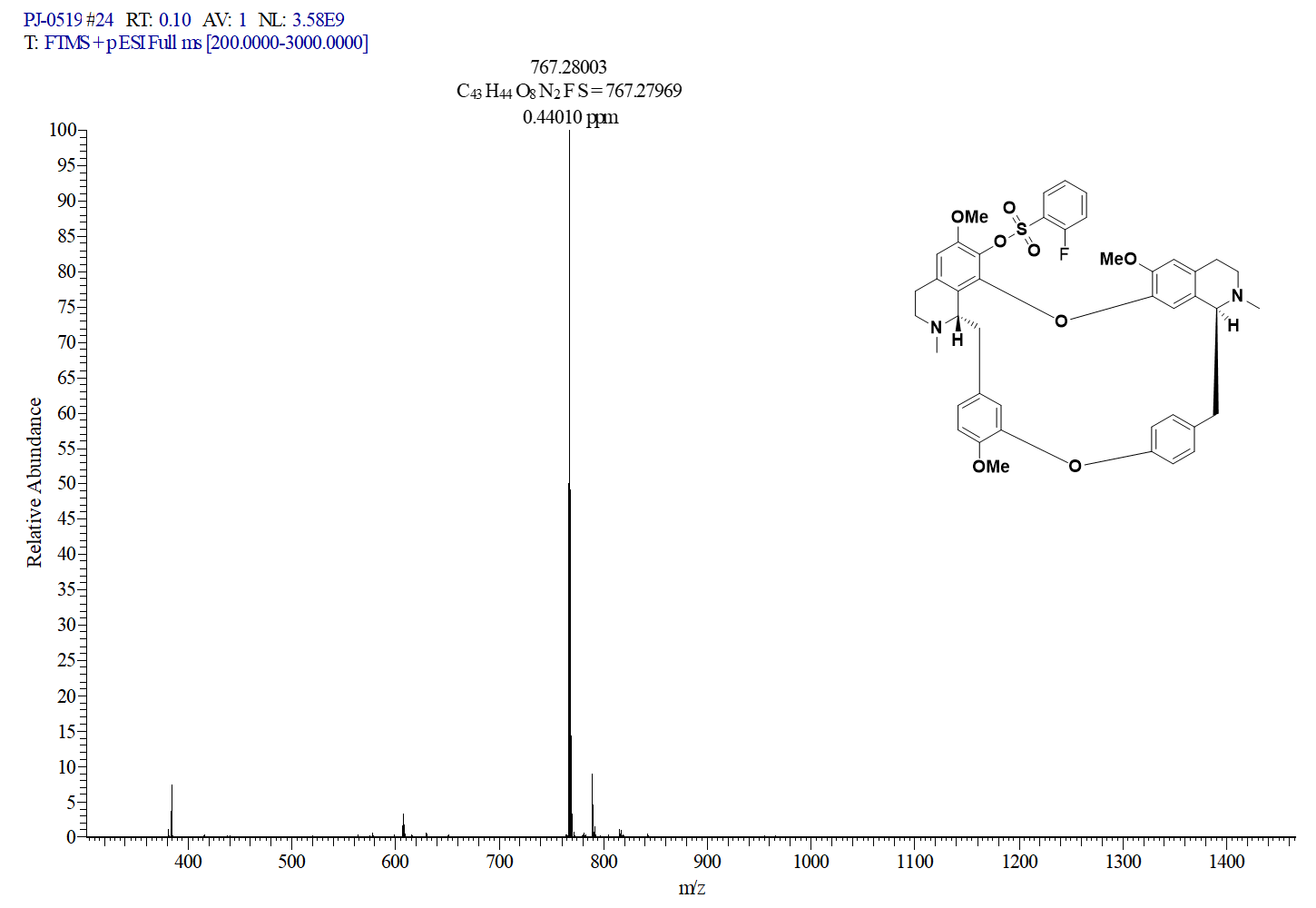


**Compound 12. 7-*O*-(4-Fluorobenzenesulfonyl)-tetrandrine: ^1^H-NMR spectrum**


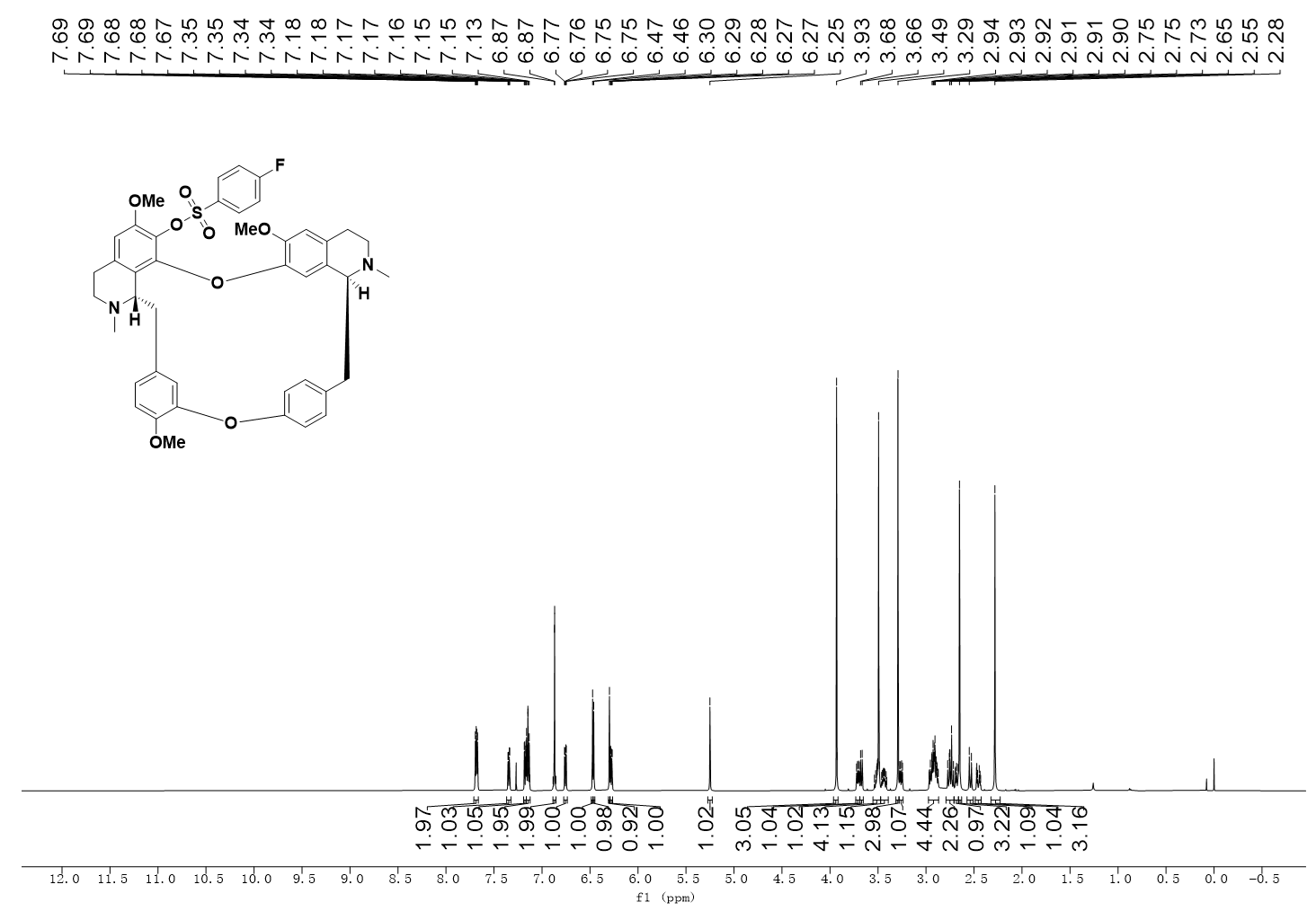


**Compound 12. 7-*O*-(4-Fluorobenzenesulfonyl)-tetrandrine: ^13^C-NMR spectrum**


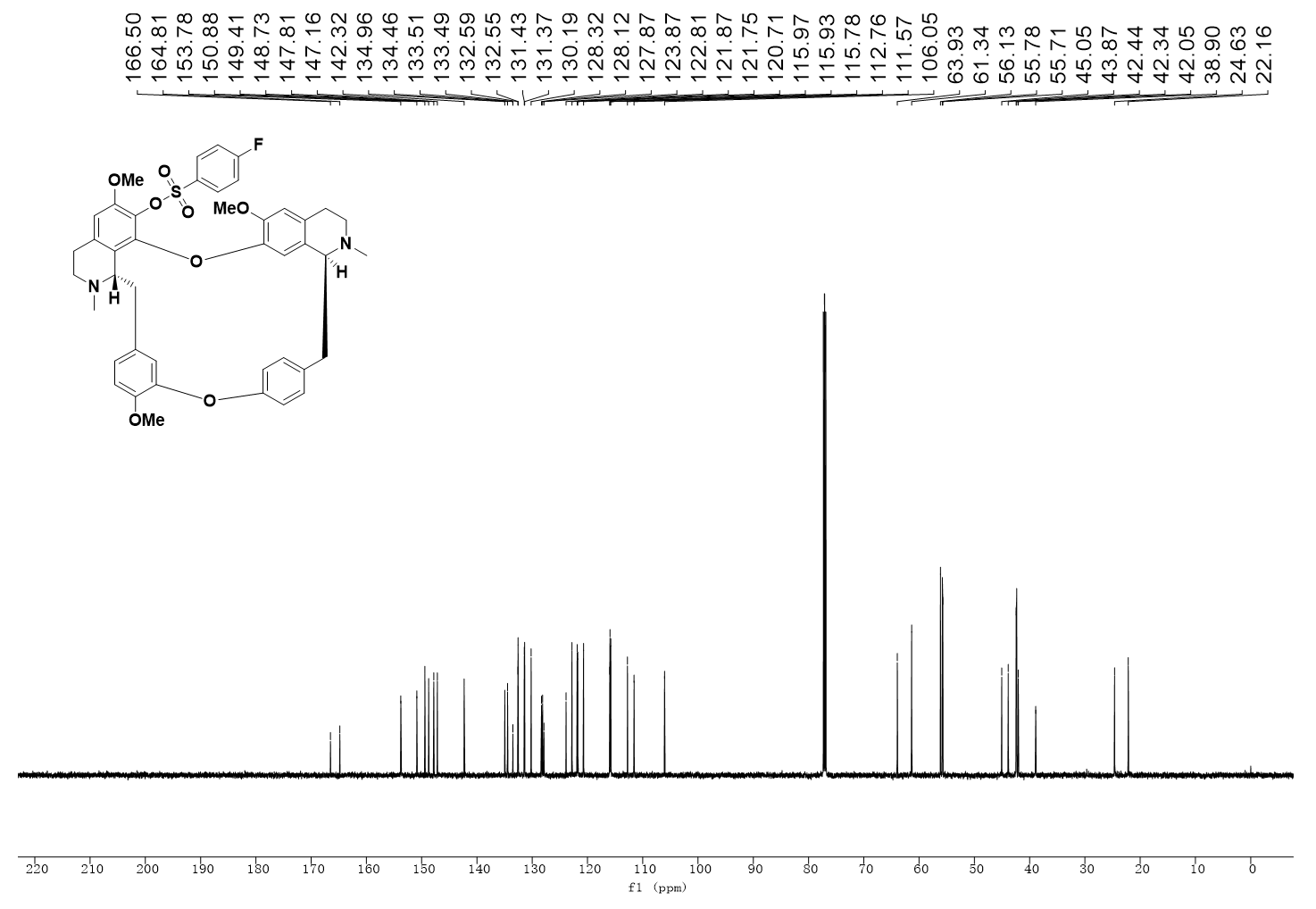


**Compound 12. 7-*O*-(4-Fluorobenzenesulfonyl)-tetrandrine: ^19^F-NMR spectrum**


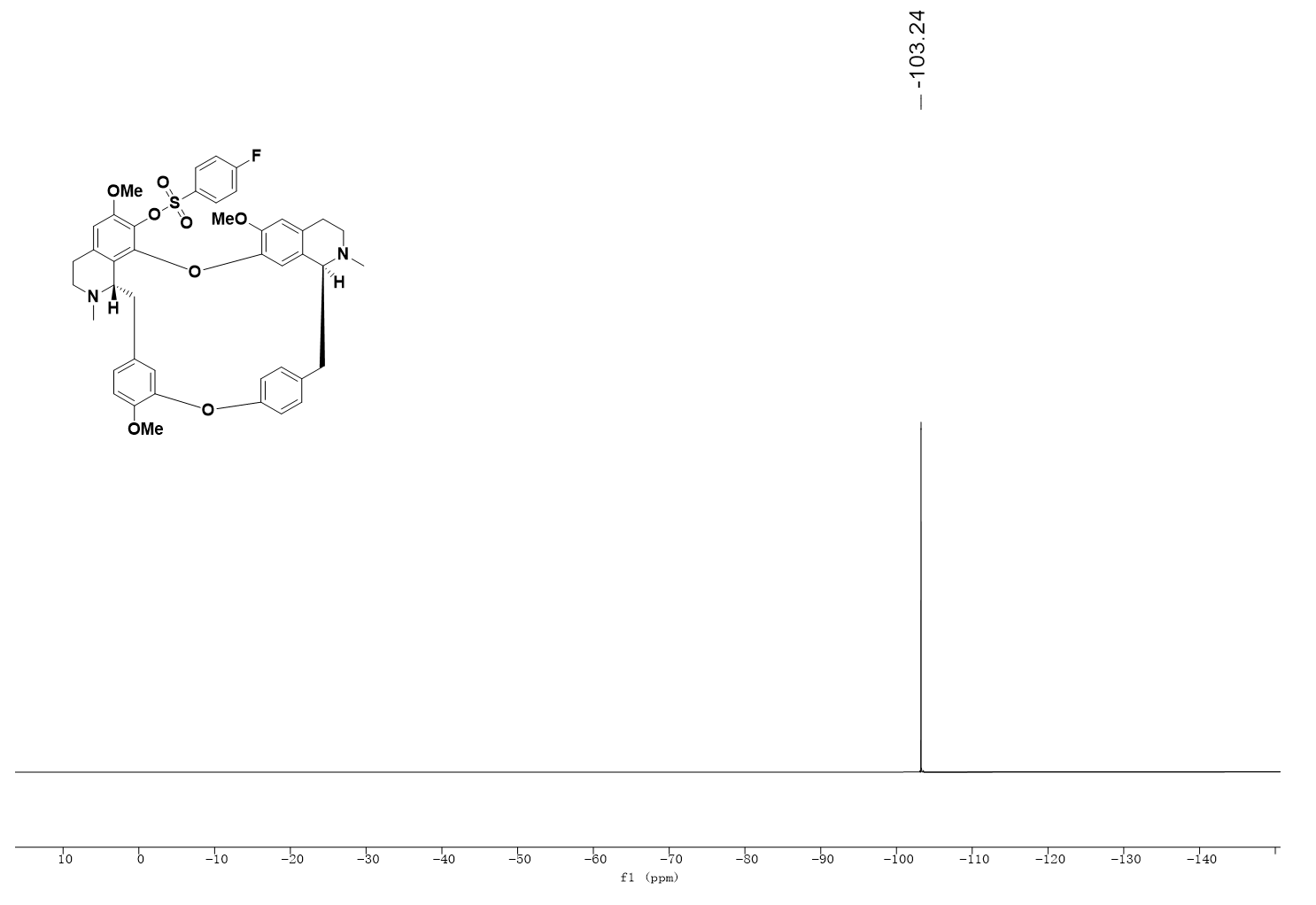


**Compound 12. 7-*O*-(4-Fluorobenzenesulfonyl)-tetrandrine: HR-ESIMS spectrum**


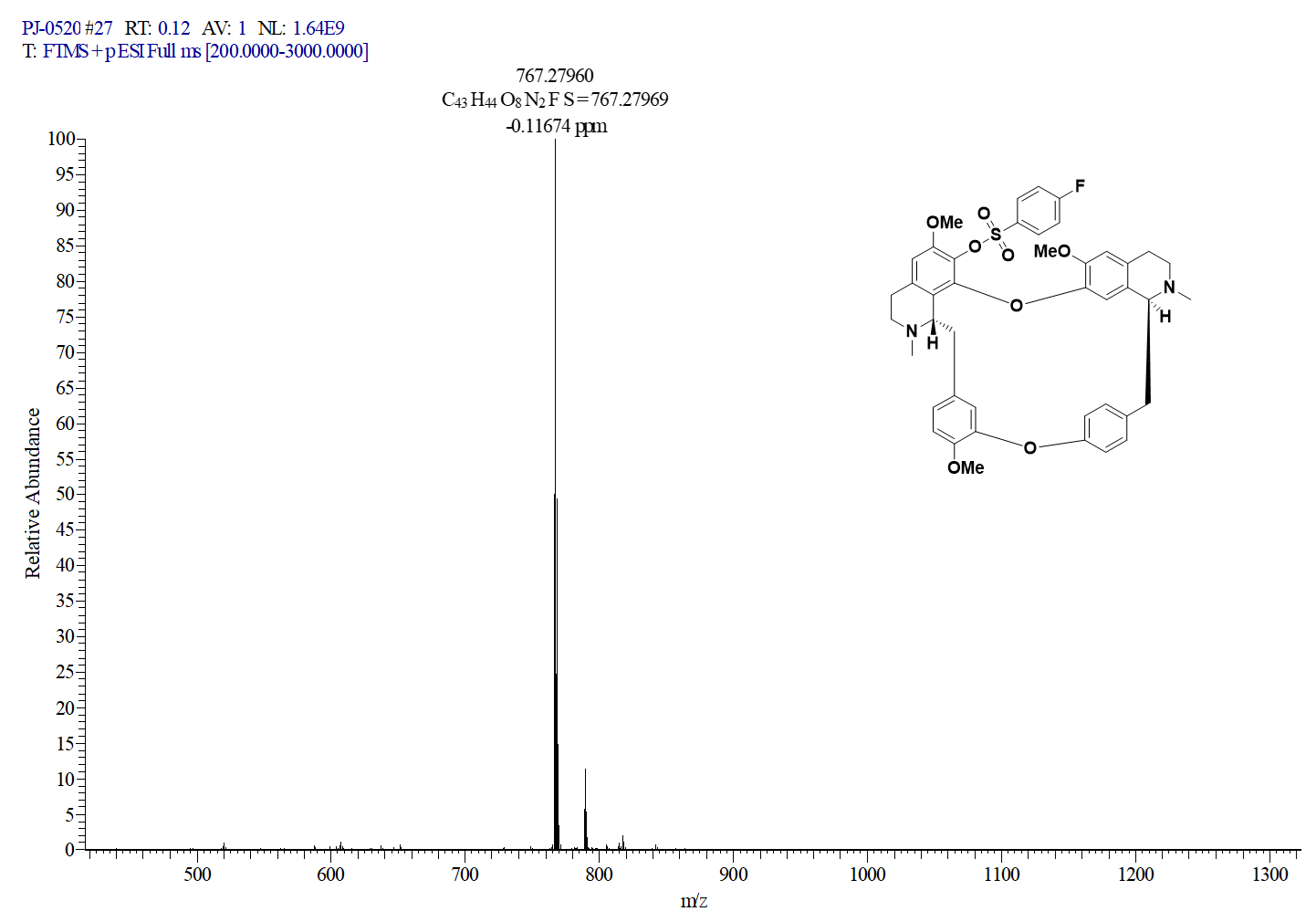


**Compound 13. 7-*O*-(3-Fluorobenzenesulfonyl)-tetrandrine: ^1^H-NMR spectrum**


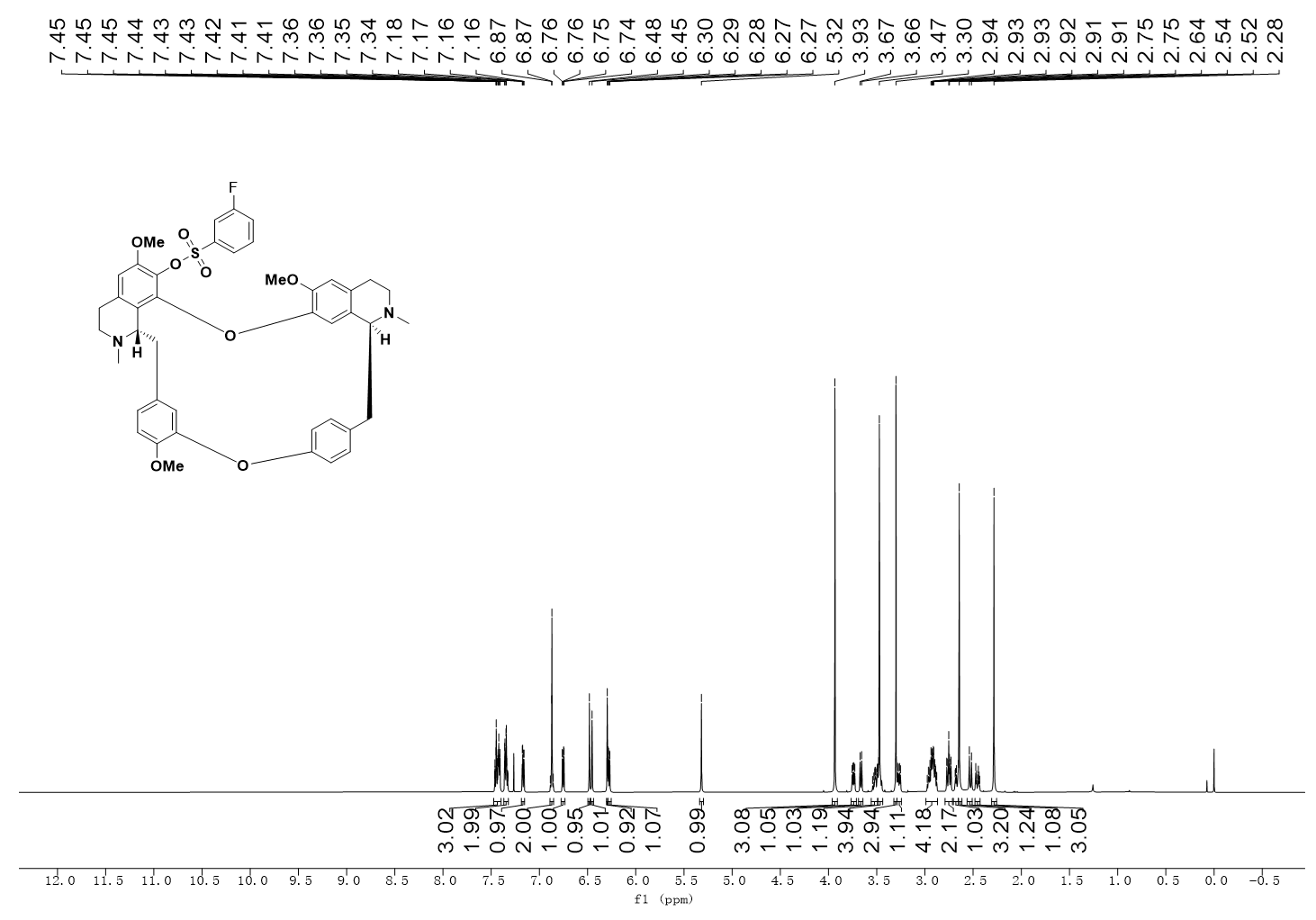


**Compound 13. 7-*O*-(3-Fluorobenzenesulfonyl)-tetrandrine: ^13^C-NMR spectrum**


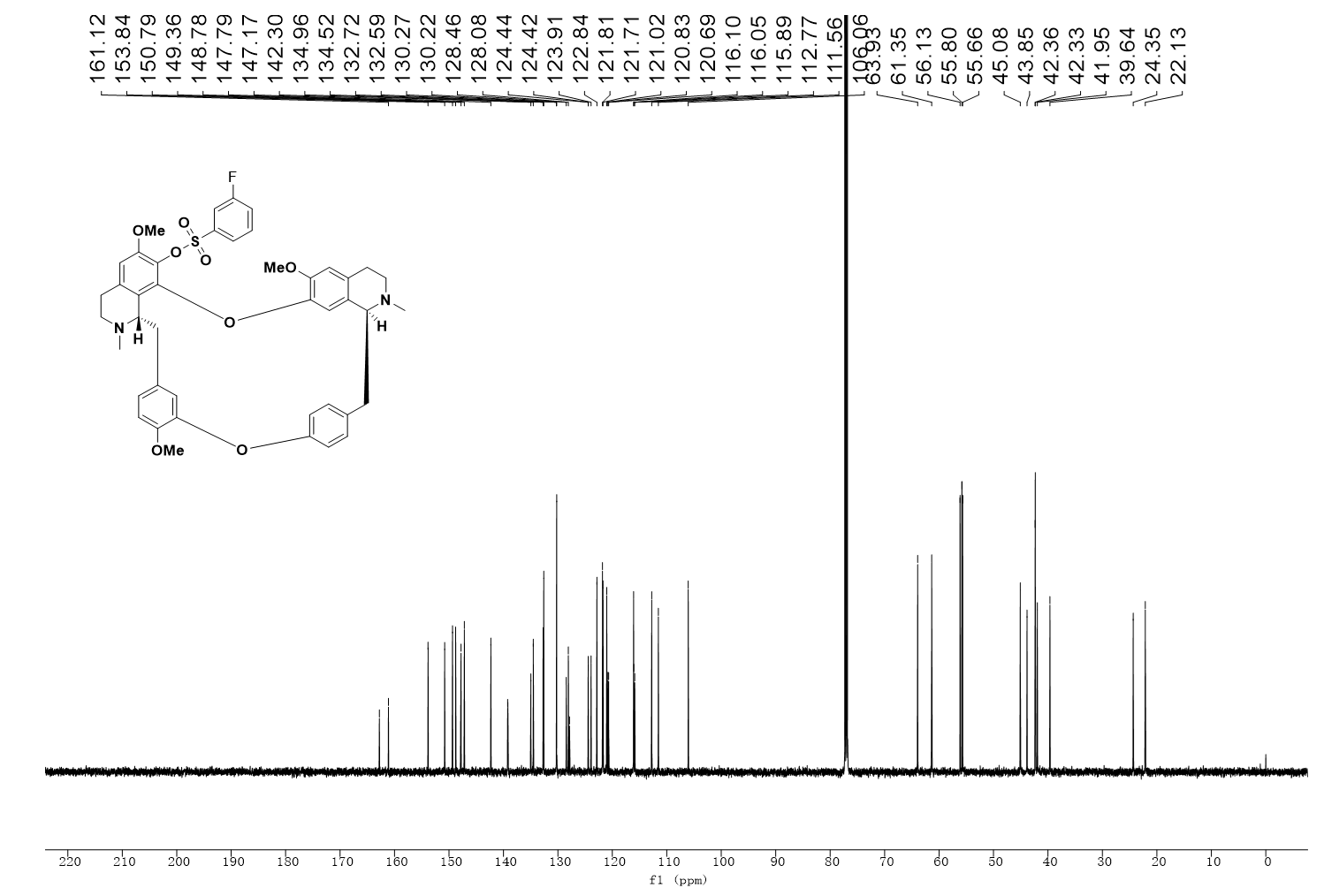


**Compound 13. 7-*O*-(3-Fluorobenzenesulfonyl)-tetrandrine: ^19^F-NMR spectrum**


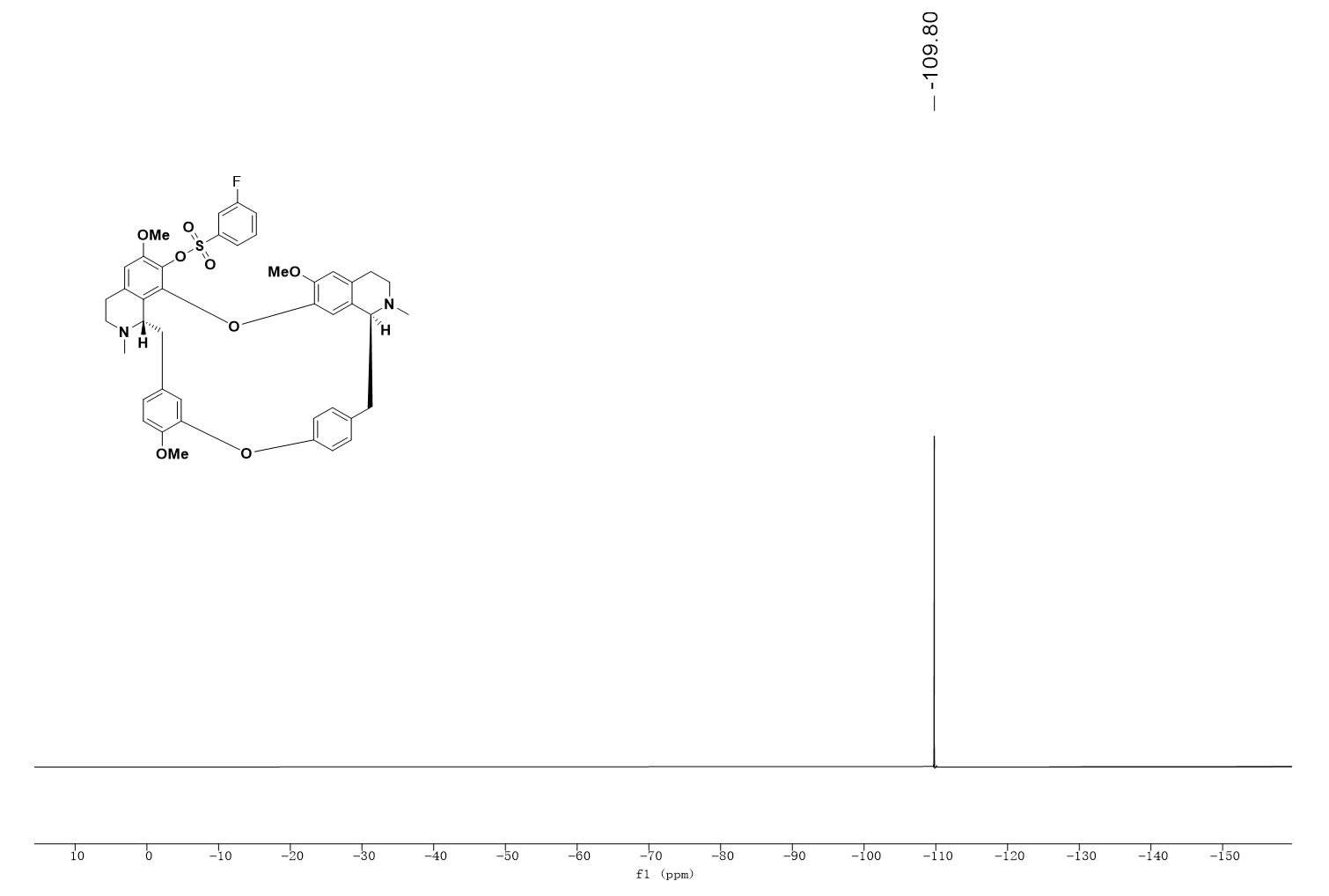


**Compound 13. 7-*O*-(3-Fluorobenzenesulfonyl)-tetrandrine: HR-ESIMS spectrum**


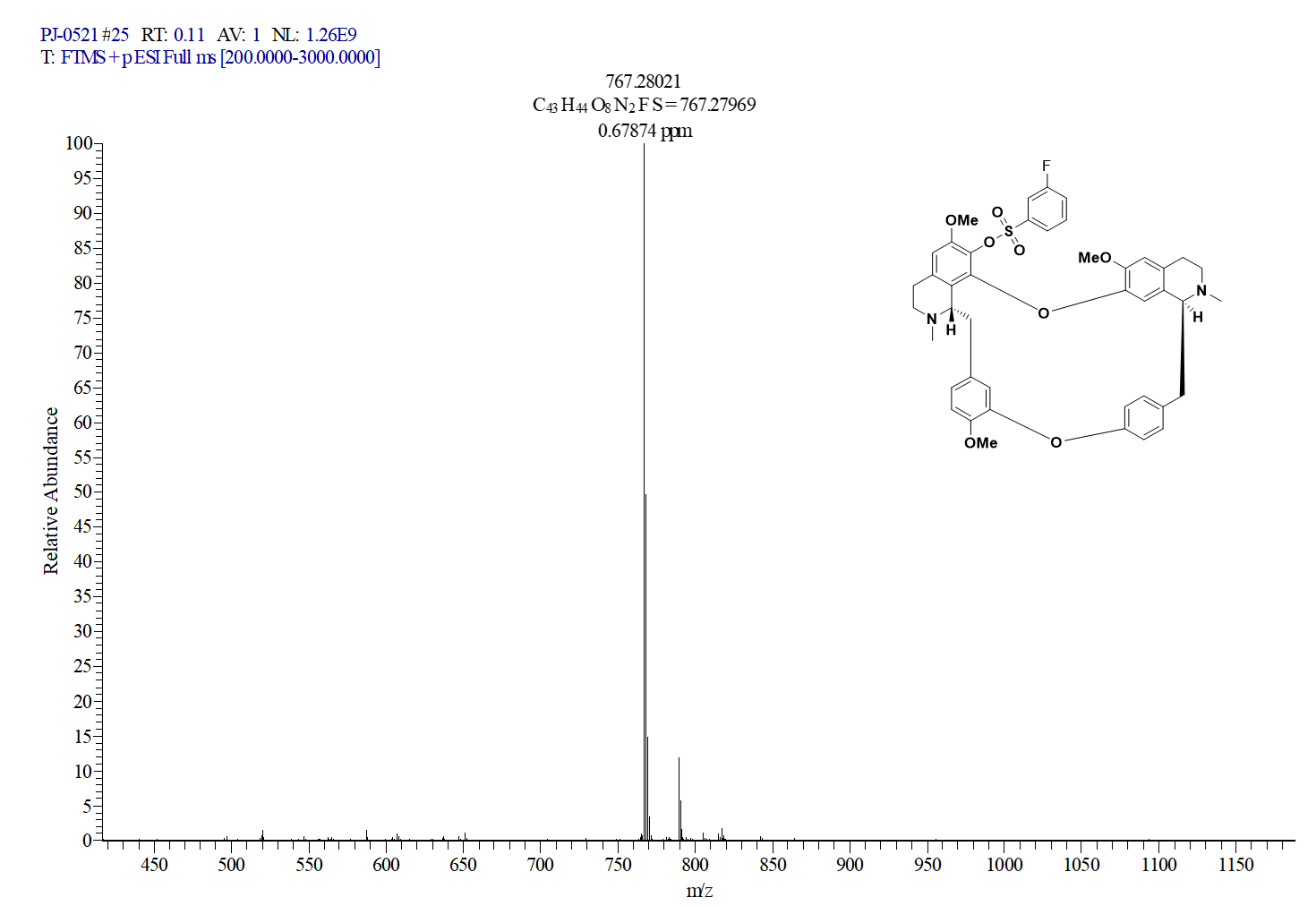


**Compound 14.** **7-*O*-Benzenesulfonyl-tetrandrine: ^1^H-NMR spectrum**


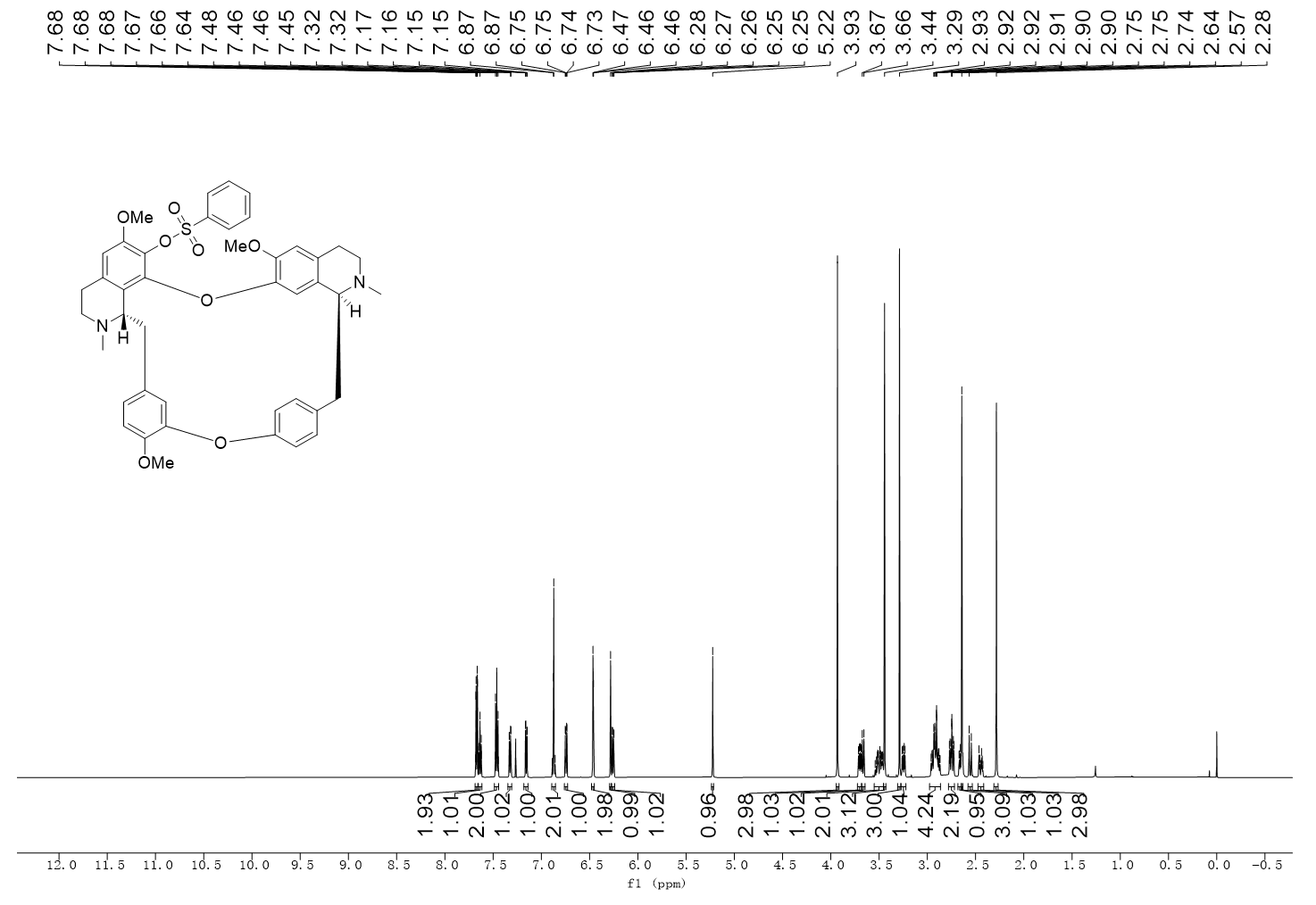


**Compound 14. 7-*O*-Benzenesulfonyl-tetrandrine: ^13^C-NMR spectrum**


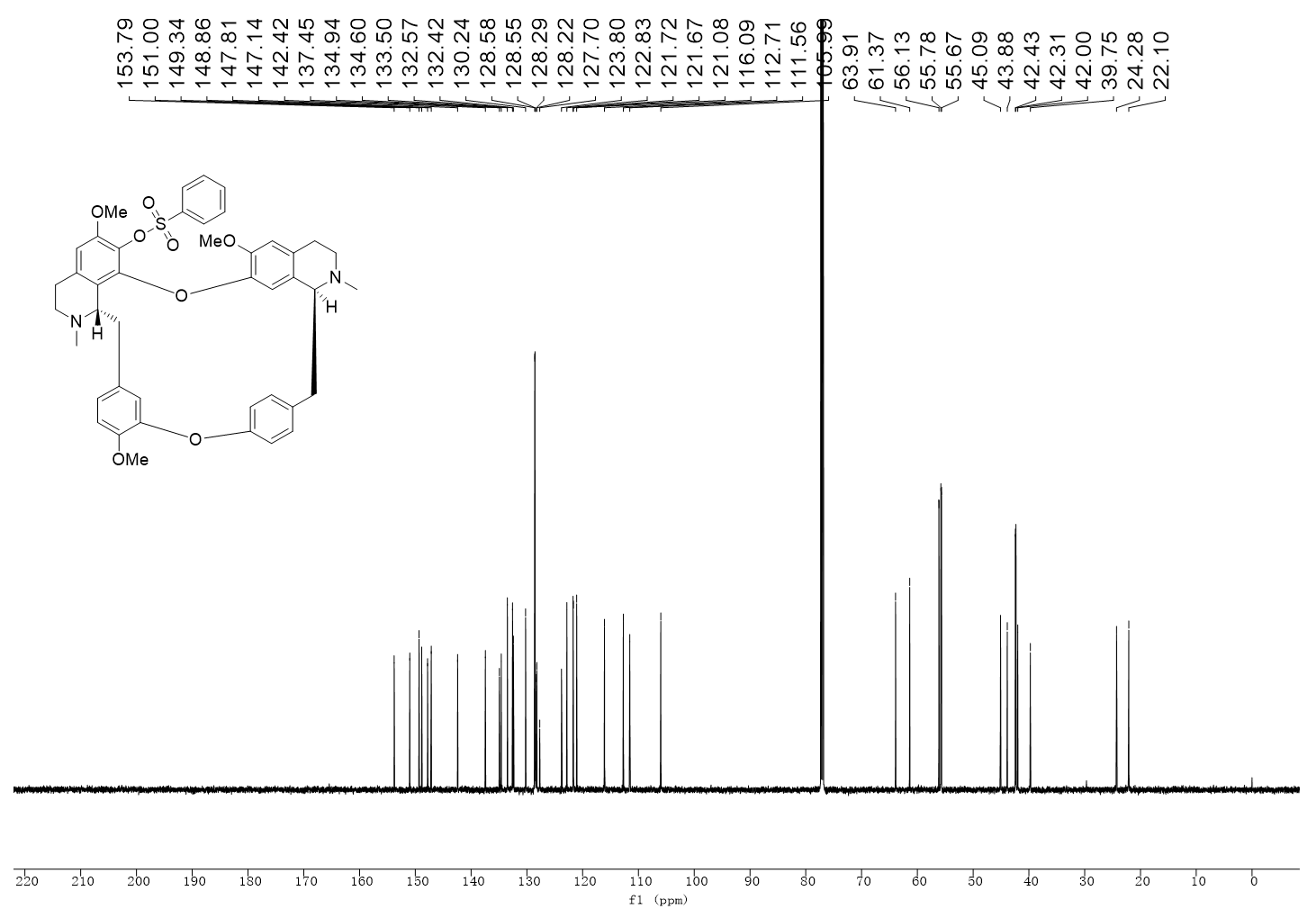


**Compound 14. 7-*O*-Benzenesulfonyl-tetrandrine: HR-ESIMS spectrum**


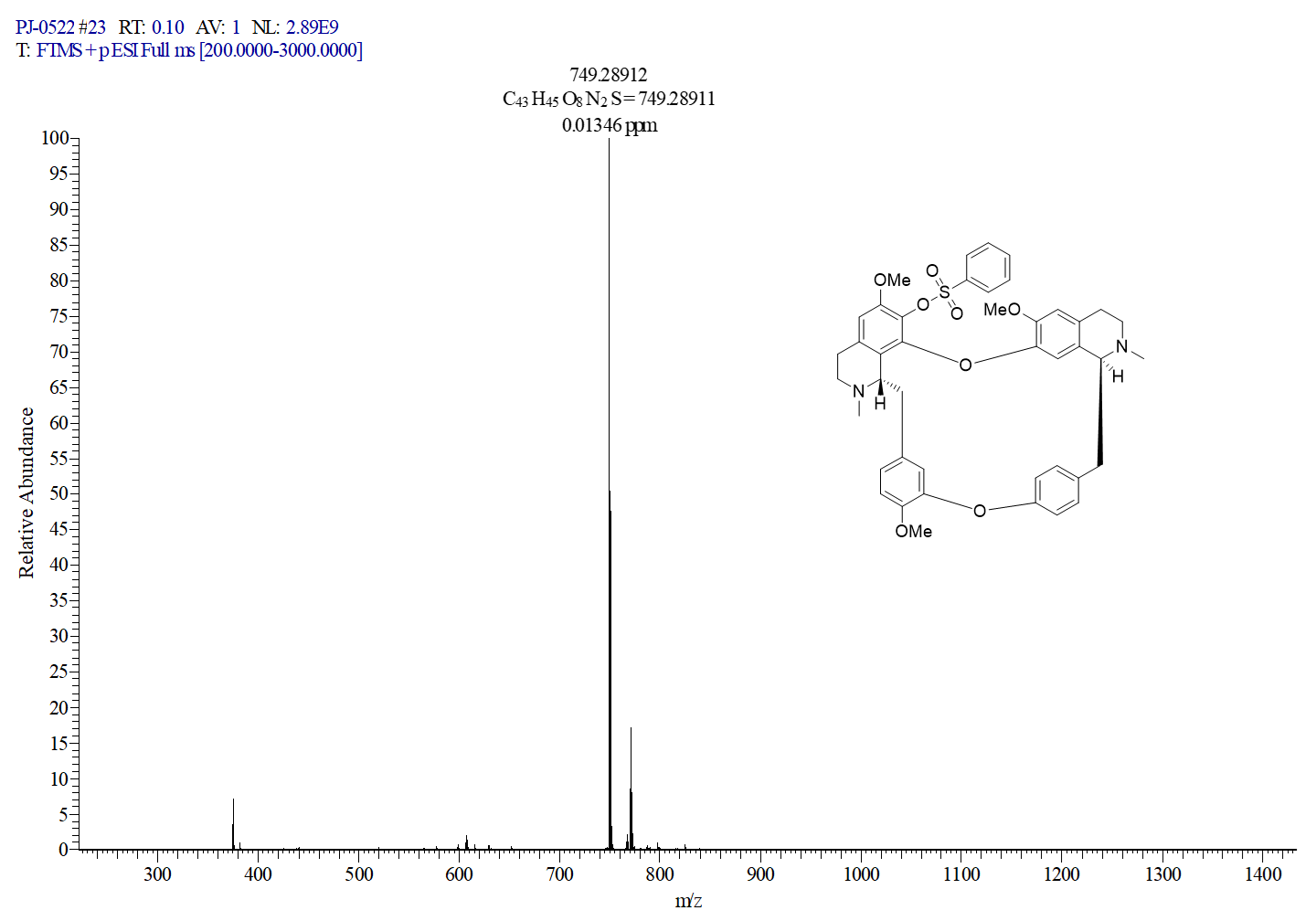


**Compound 15****. 7-*O*-(alpha-Toluenesulfonyl)-tetrandrine: ^1^H-NMR spectrum**


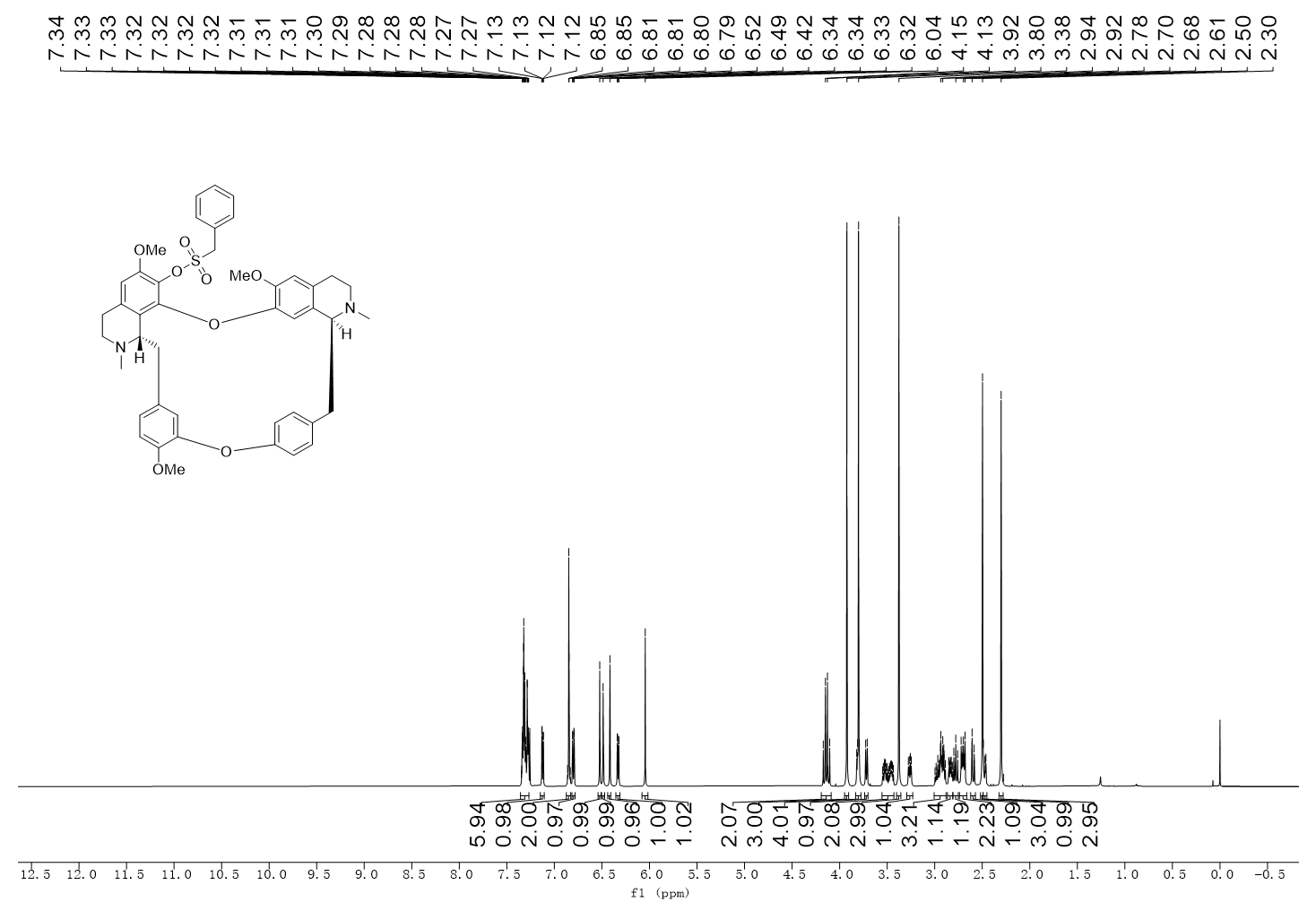


**Compound 15. 7-*O*-(alpha-Toluenesulfonyl)-tetrandrine: ^13^C-NMR spectrum**


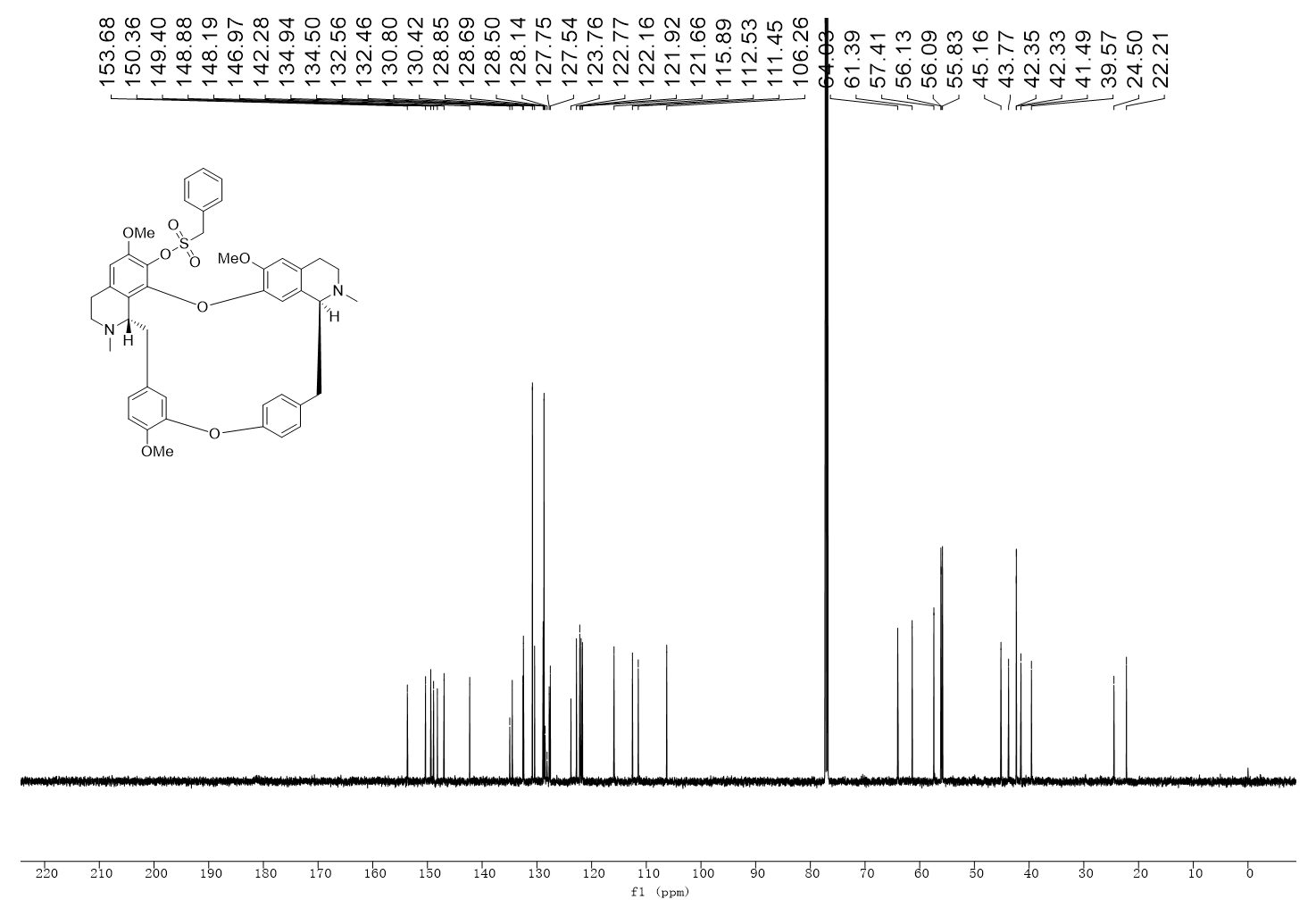


**Compound 15. 7-*O*-(alpha-Toluenesulfonyl)-tetrandrine: HR-ESIMS spectrum**


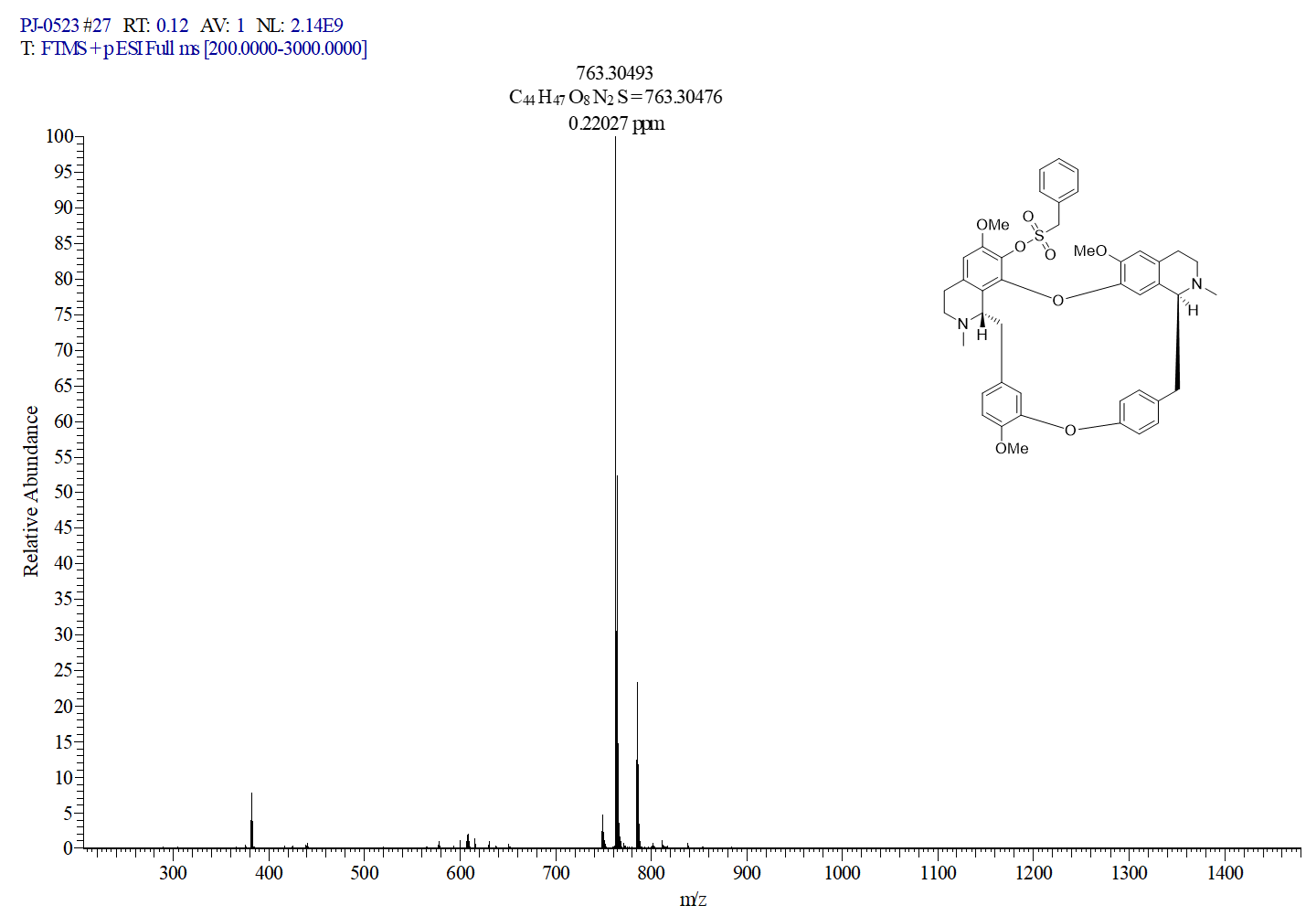


**Compound 16****. 7-*O*-(4-Toluenesulfonyl)-tetrandrine: ^1^H-NMR spectrum**


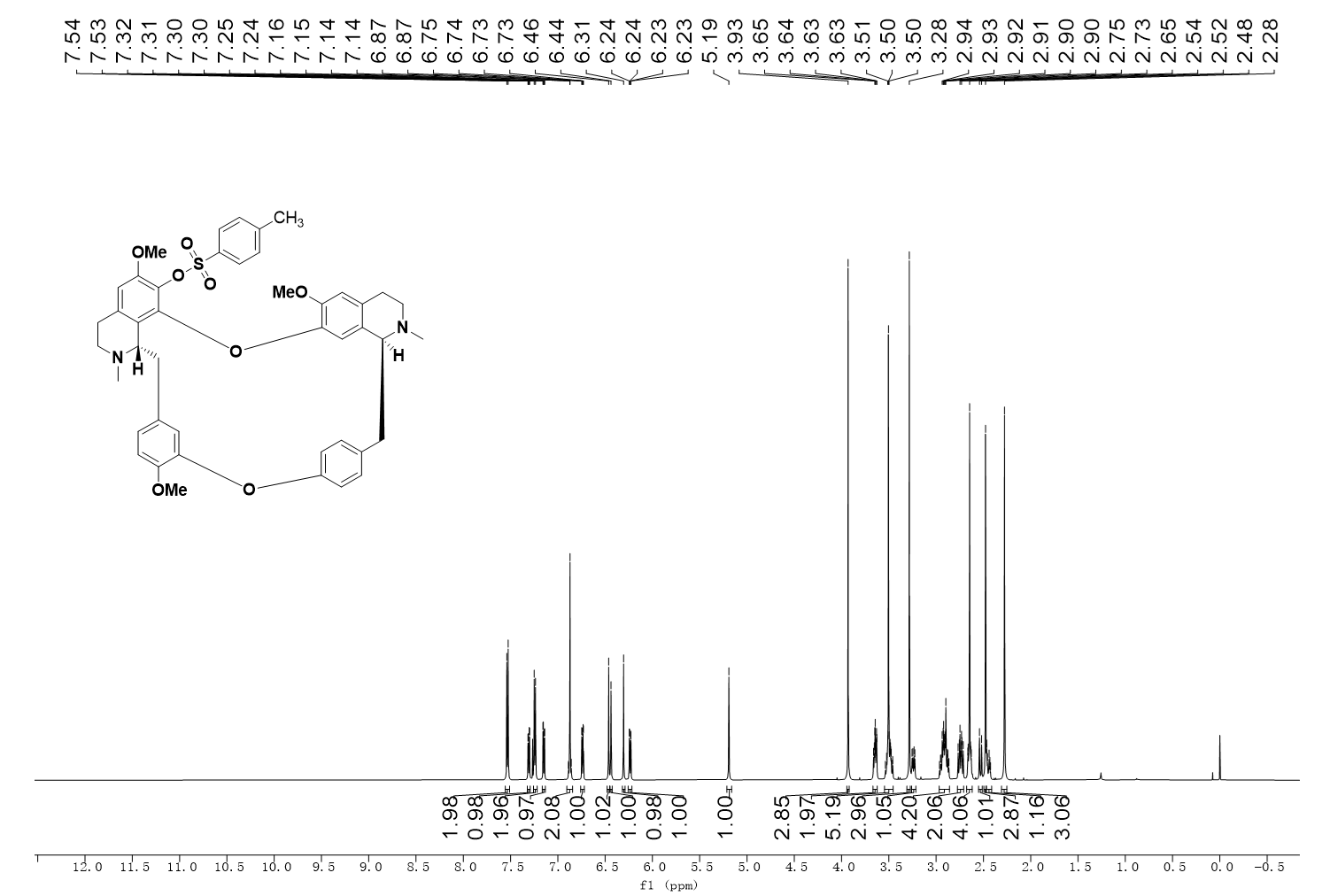


**Compound 16. 7-*O*-(4-Toluenesulfonyl)-tetrandrine: ^13^C-NMR spectrum**


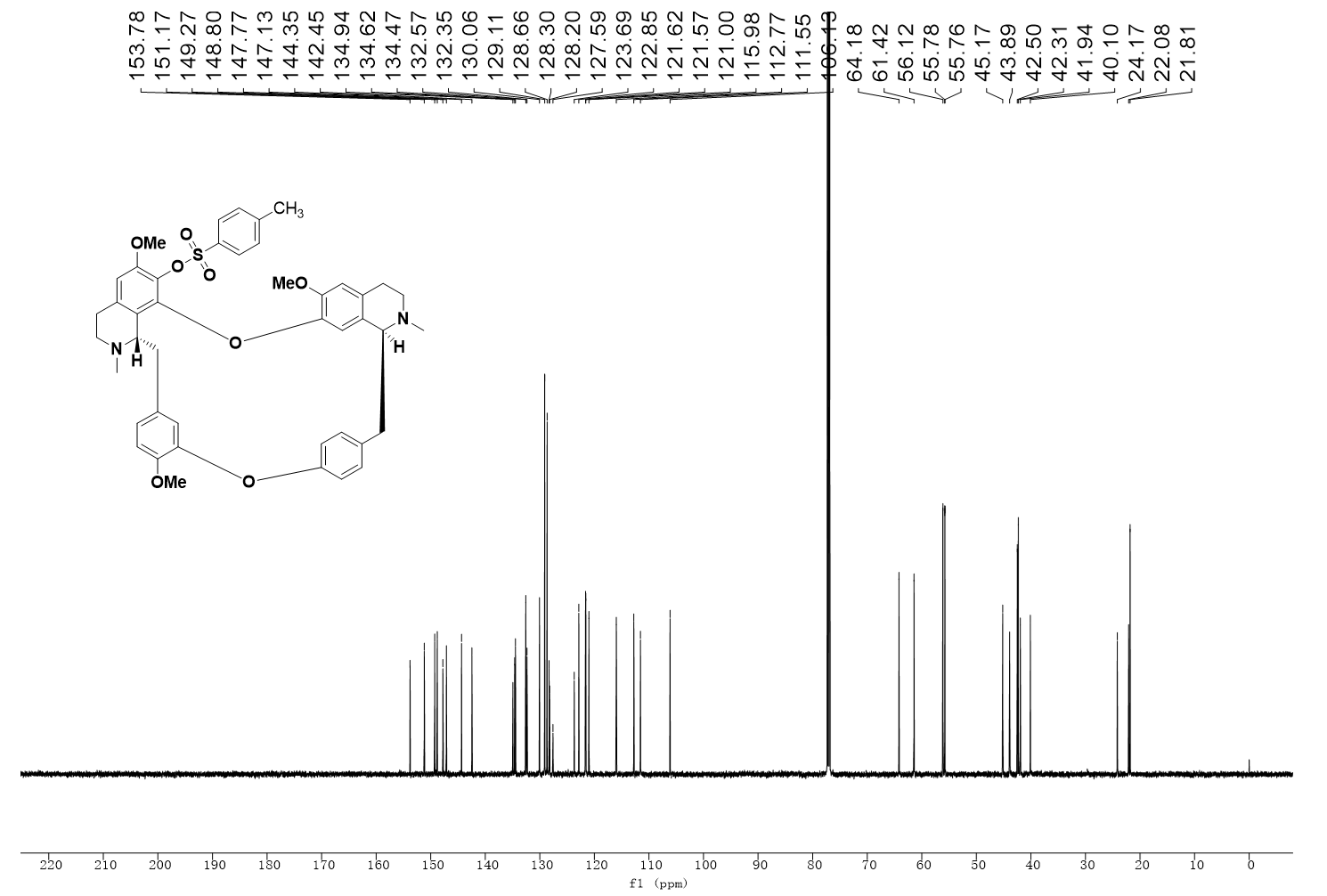


**Compound 16. 7-*O*-(4-Toluenesulfonyl)-tetrandrine: HR-ESIMS spectrum**


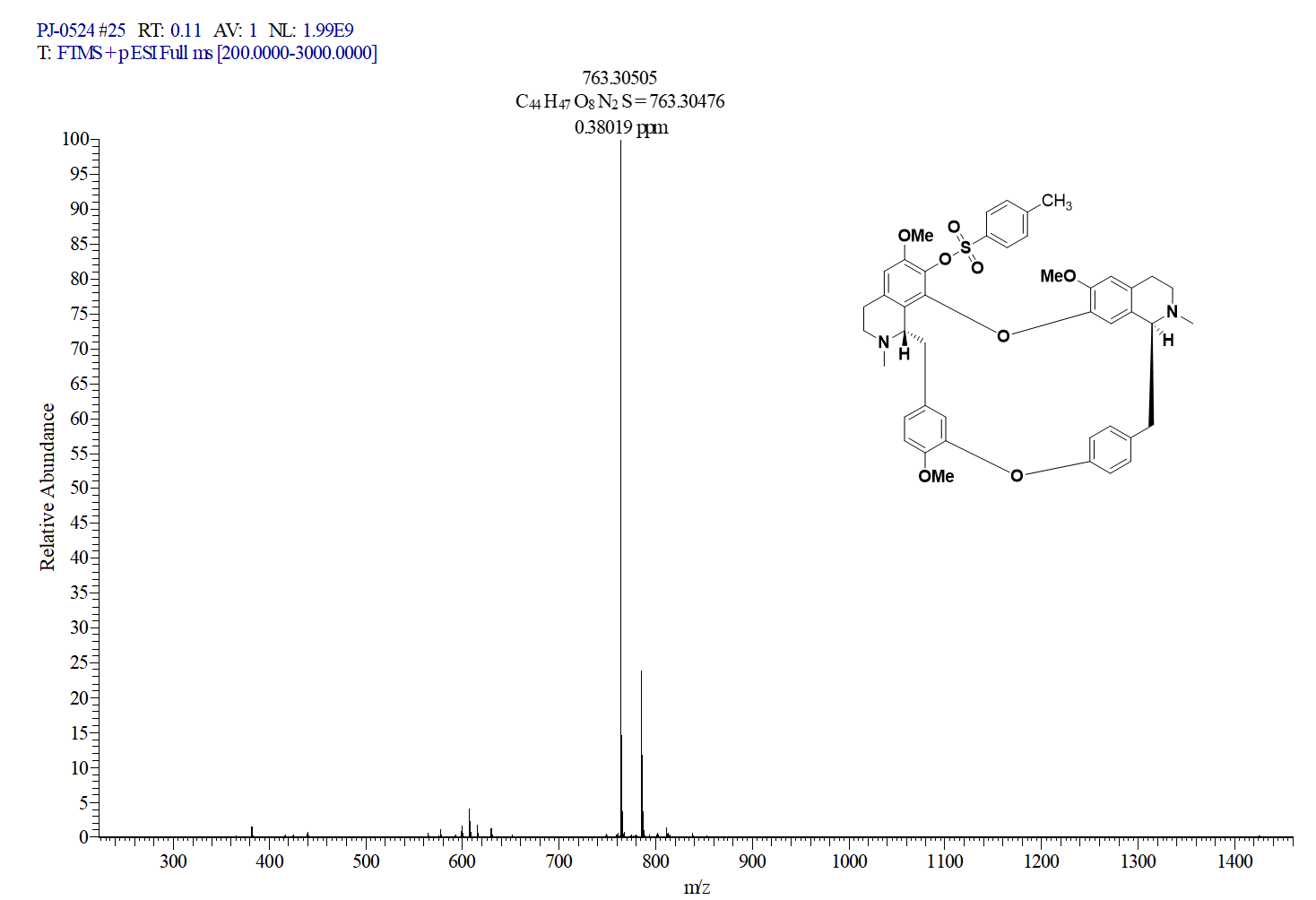


**Compound 17.** **7-*O*-(4-Methoxybenzenesulfonyl)-tetrandrine: ^1^H-NMR spectrum**


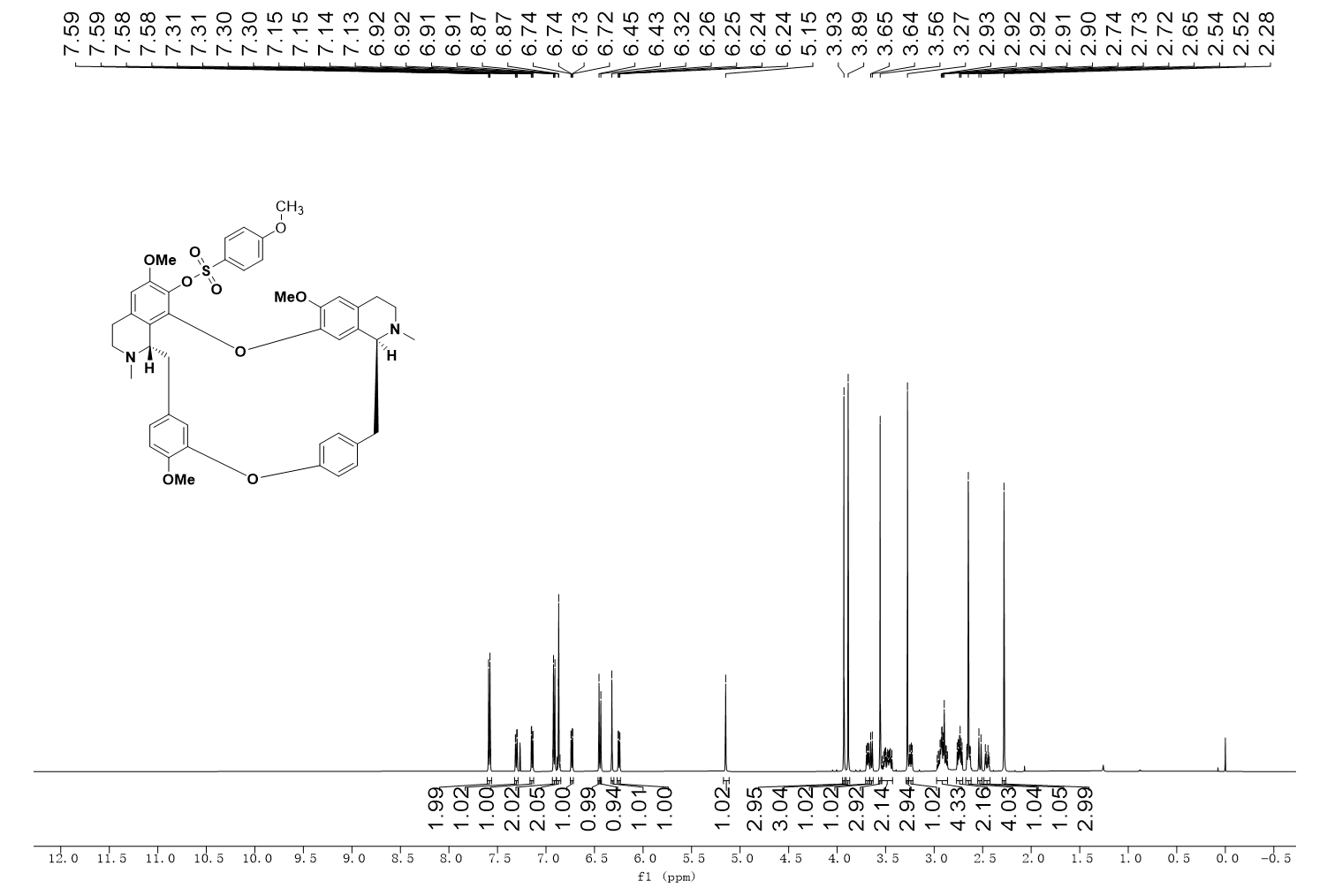


**Compound 17. 7-*O*-(4-Methoxybenzenesulfonyl)-tetrandrine: ^13^C-NMR spectrum**


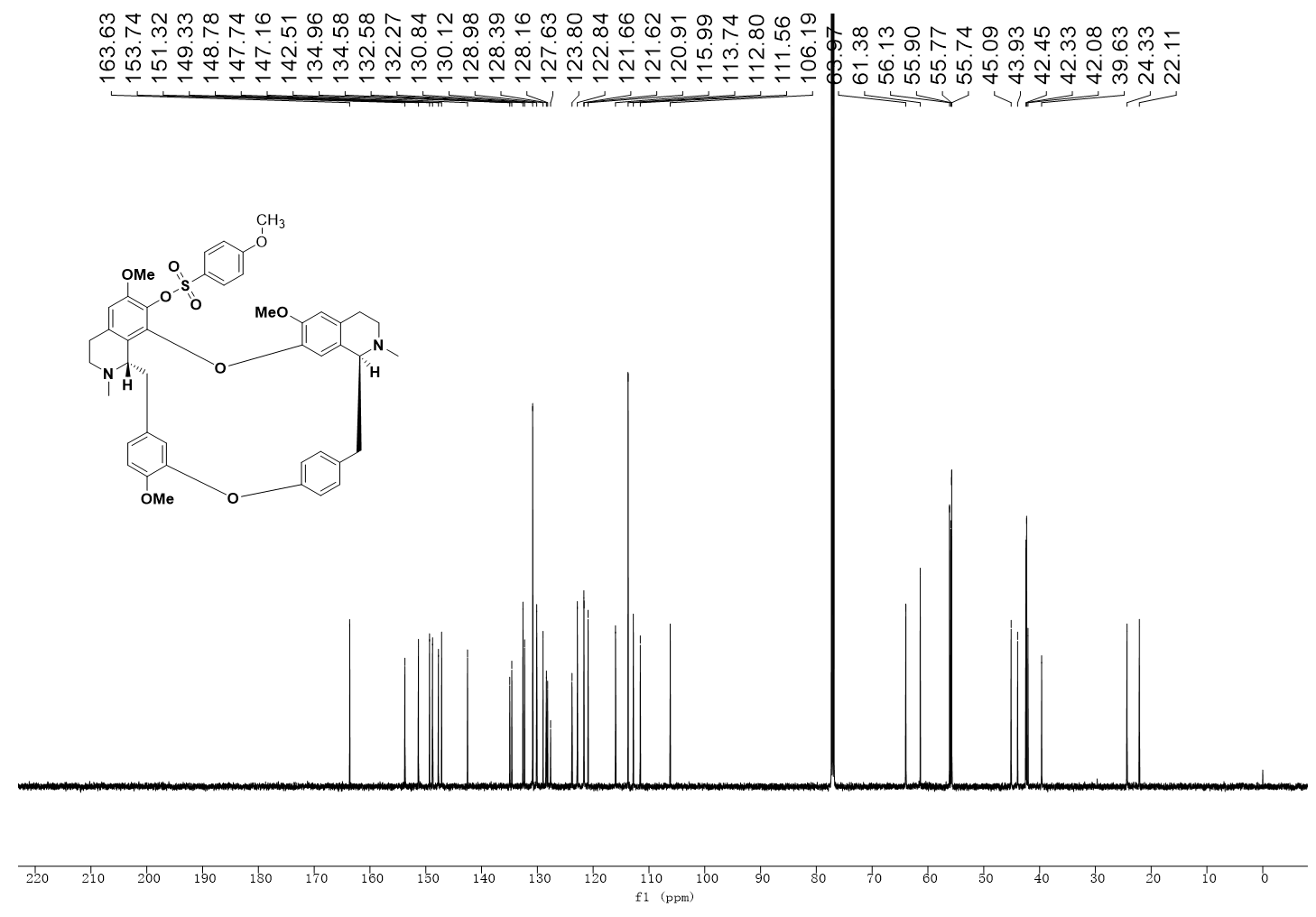


**Compound 17. 7-*O*-(4-Methoxybenzenesulfonyl)-tetrandrine: HR-ESIMS spectrum**


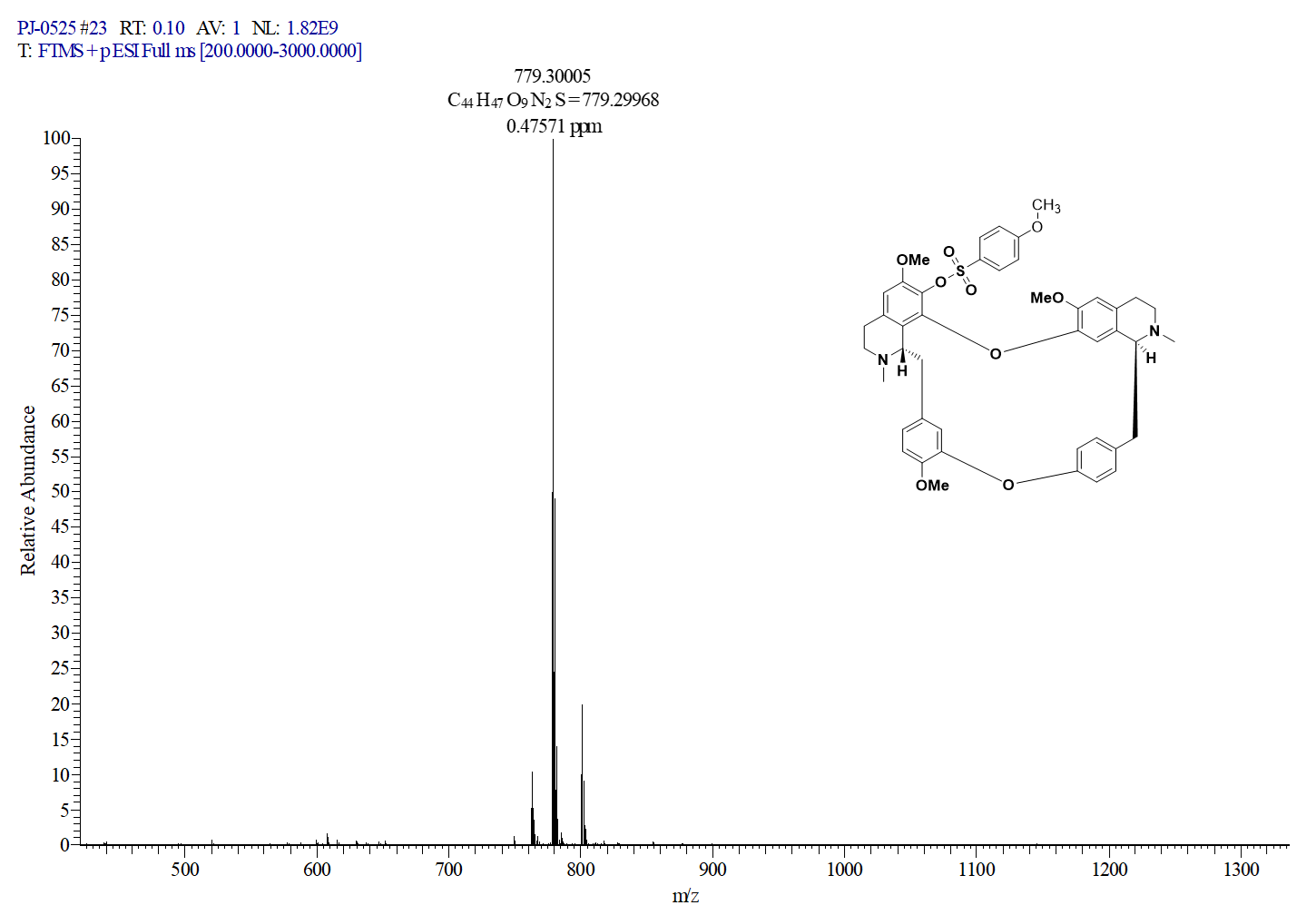


**Compound 18.** **7-*O*-(2-Chlorobenzenesulfonyl)-tetrandrine: ^1^H-NMR spectrum**


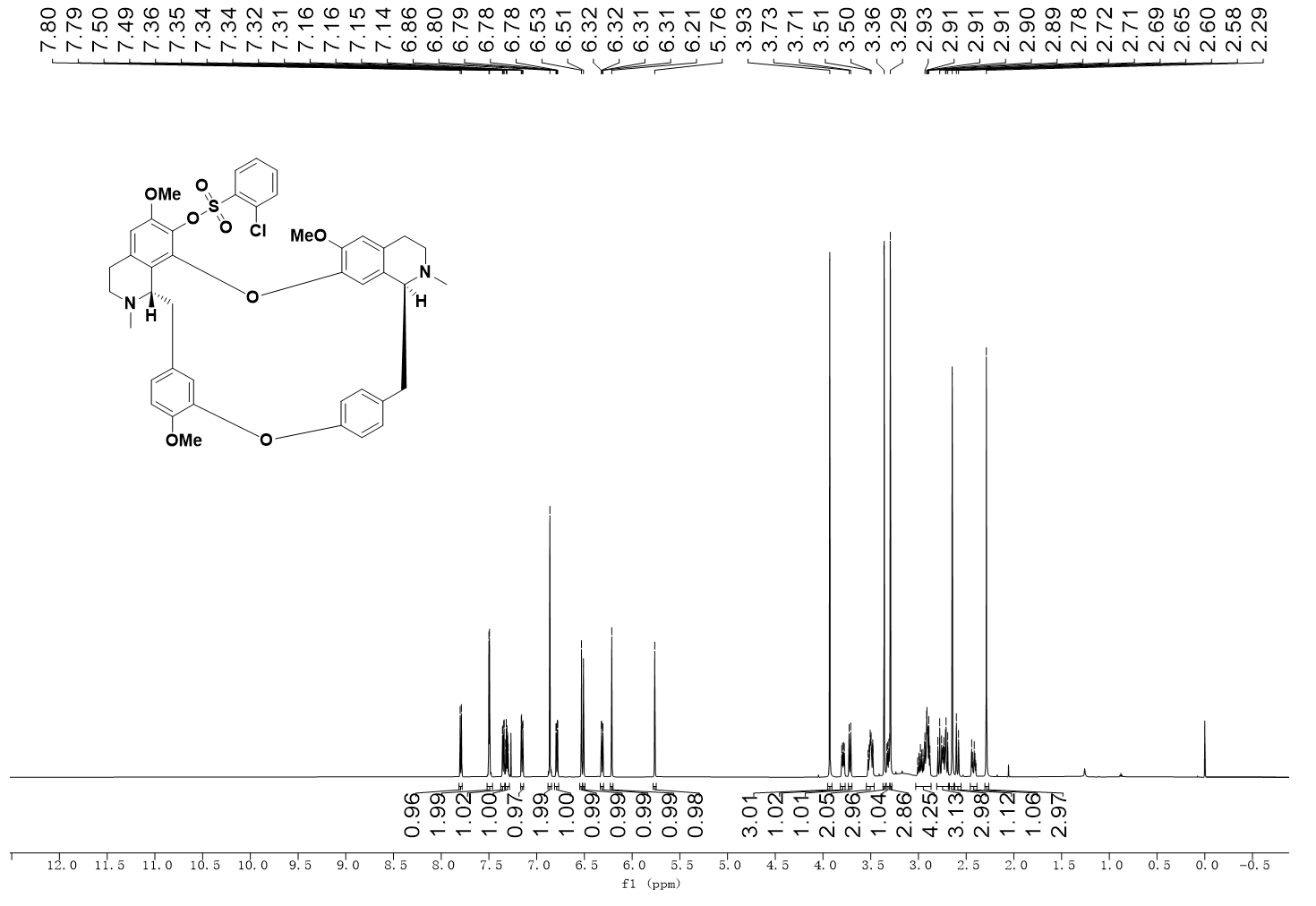


**Compound 18. 7-*O*-(2-Chlorobenzenesulfonyl)-tetrandrine: ^13^C-NMR spectrum**


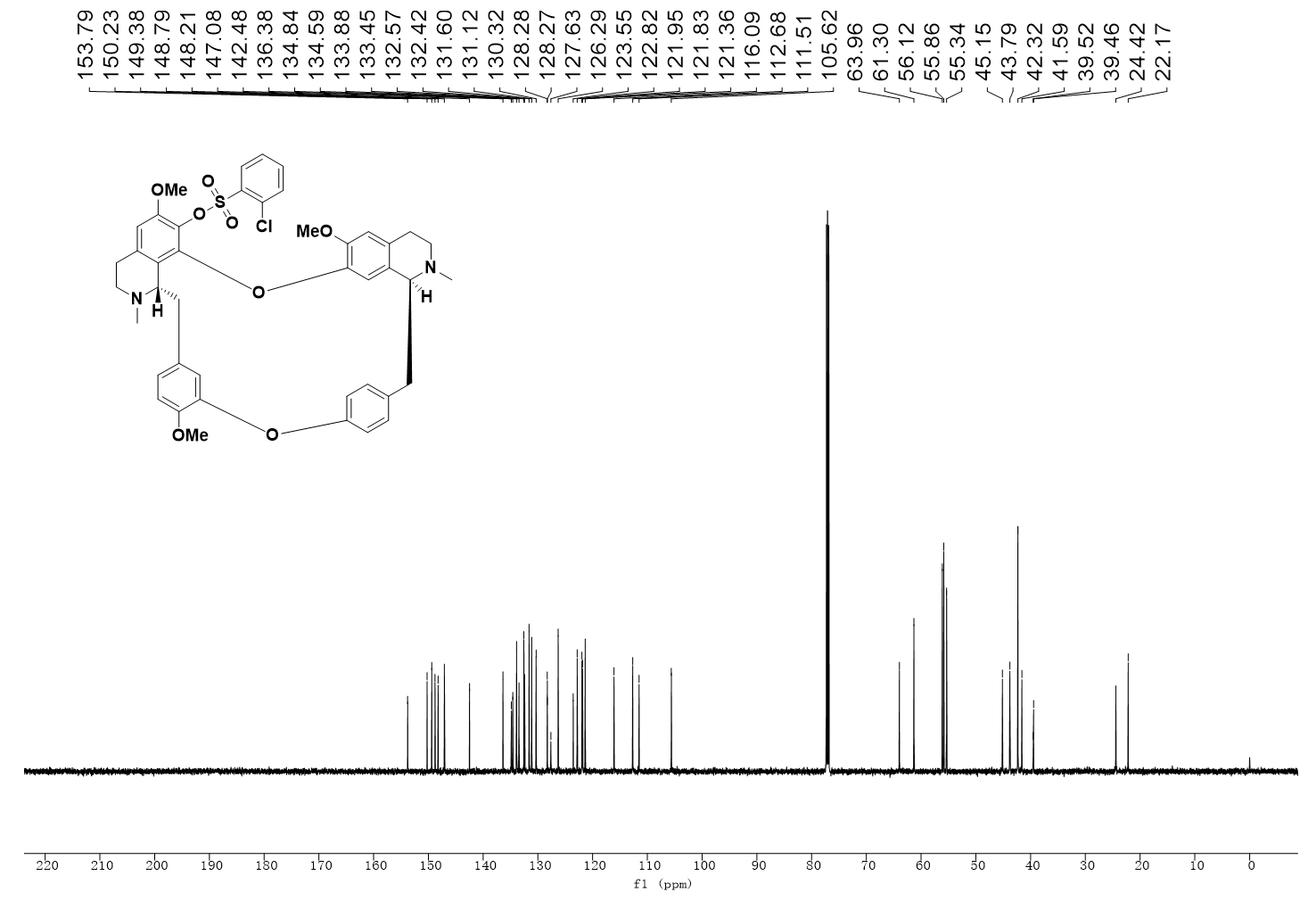


**Compound 18. 7-*O*-(2-Chlorobenzenesulfonyl)-tetrandrine: HR-ESIMS spectrum**


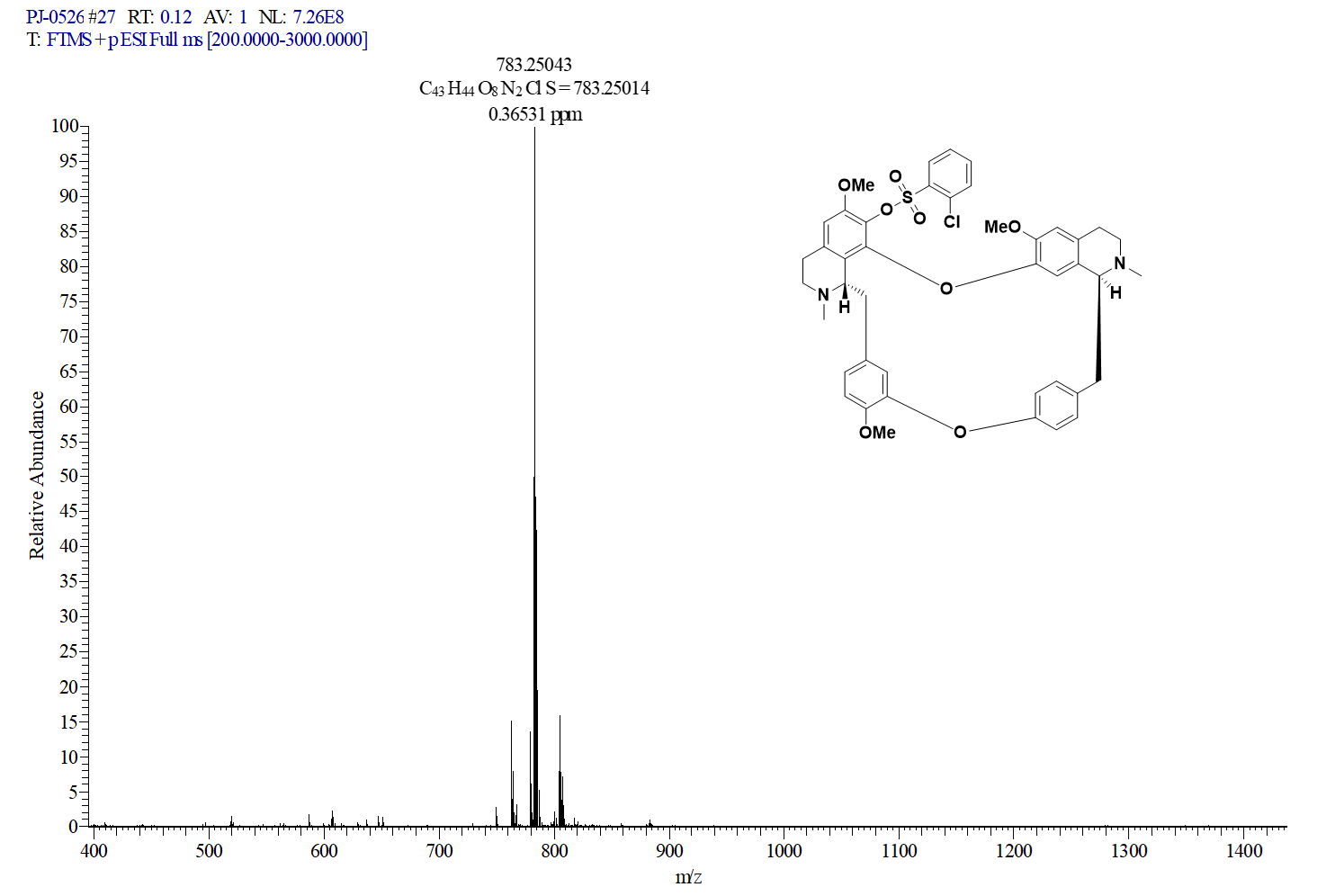


**Compound 19.** **7-*O*-(3-Chlorobenzenesulfonyl)-tetrandrine: ^1^H-NMR spectrum**


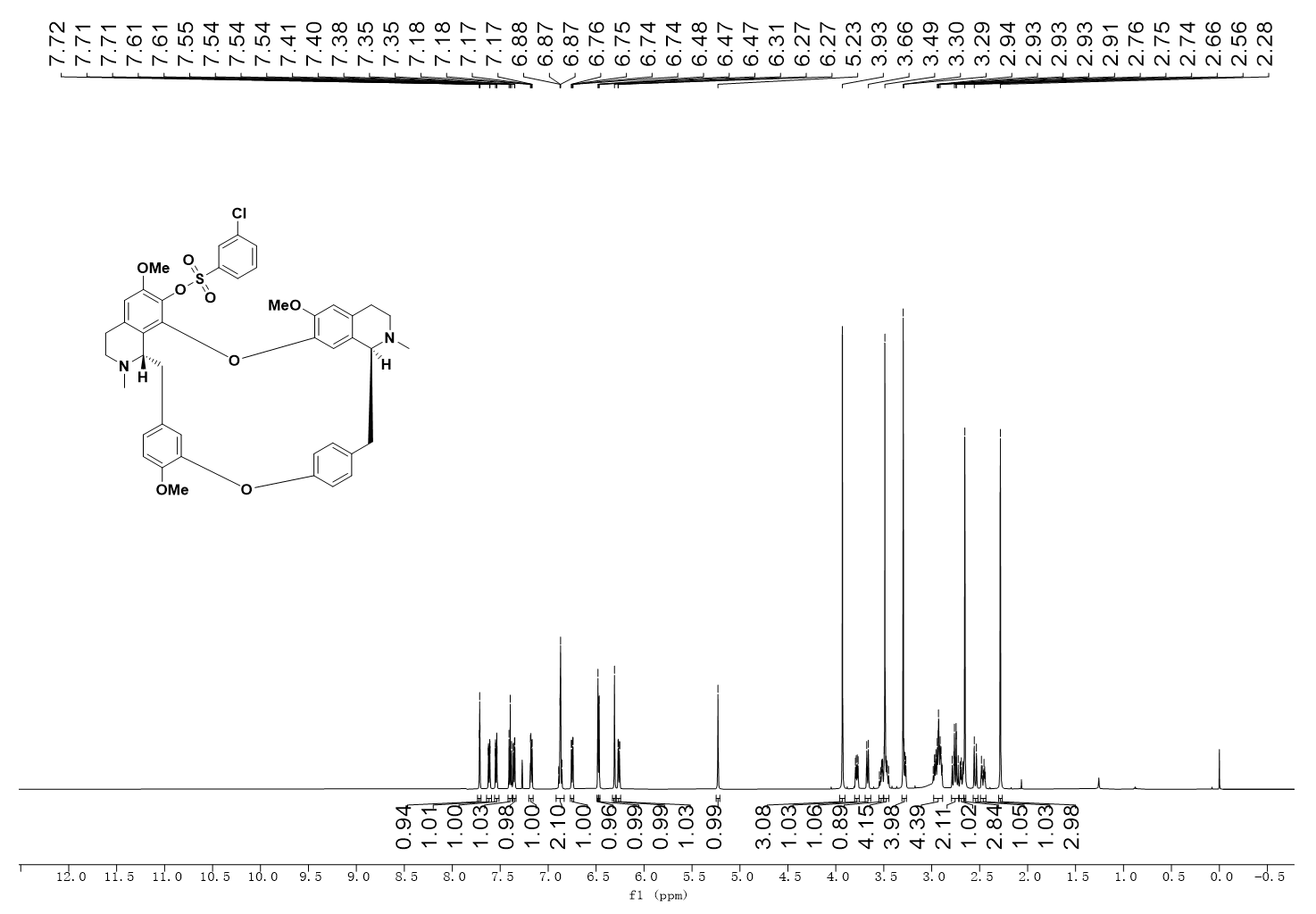


**Compound 19. 7-*O*-(3-Chlorobenzenesulfonyl)-tetrandrine: ^13^C-NMR spectrum**


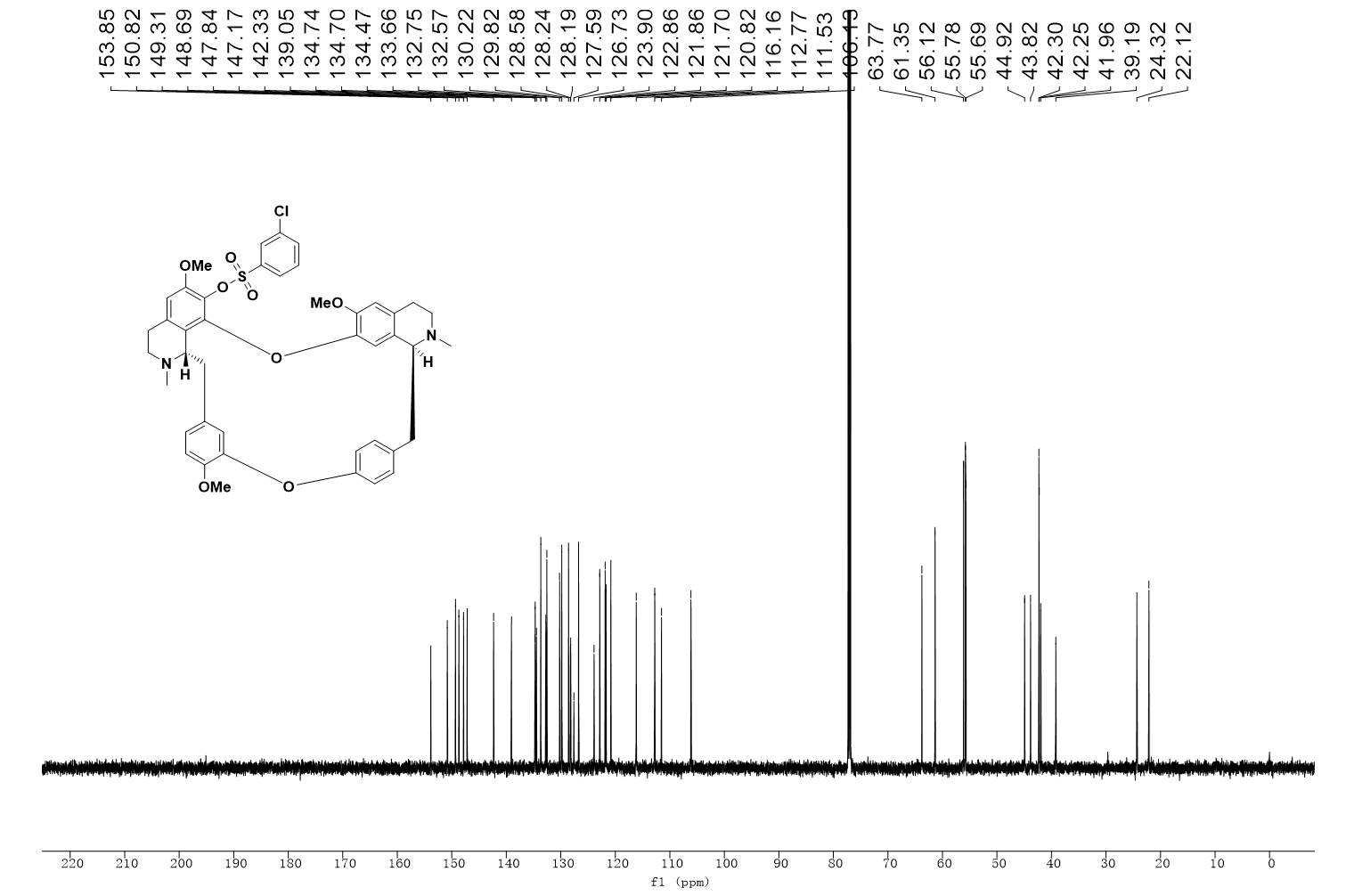


**Compound 19. 7-*O*-(3-Chlorobenzenesulfonyl)-tetrandrine: HR-ESIMS spectrum**


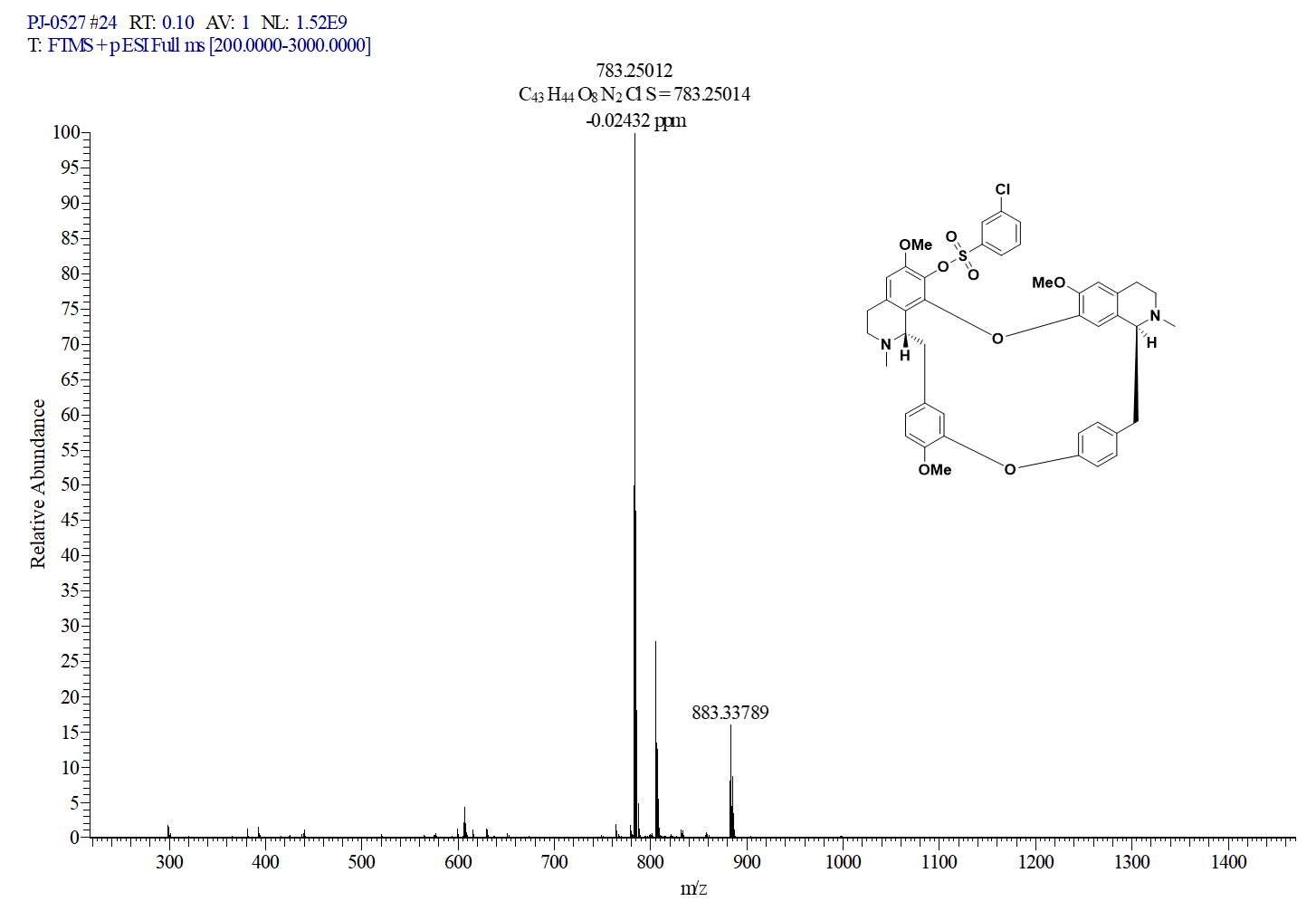


**Compound 20.** **7-*O*-(4-Chlorobenzenesulfonyl)-tetrandrine: ^1^H-NMR spectrum**


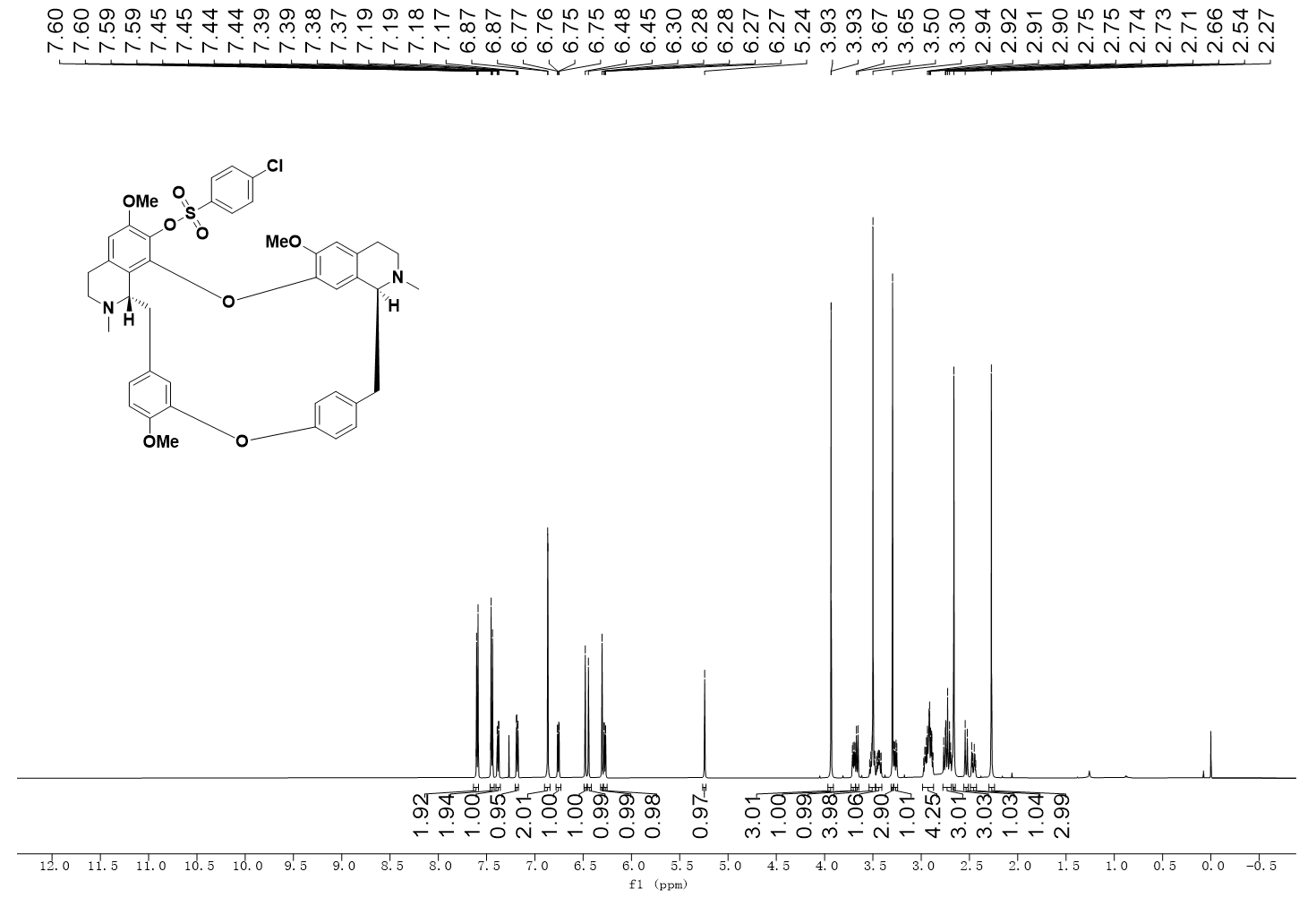


**Compound 20. 7-*O*-(4-Chlorobenzenesulfonyl)-tetrandrine: ^13^C-NMR spectrum**


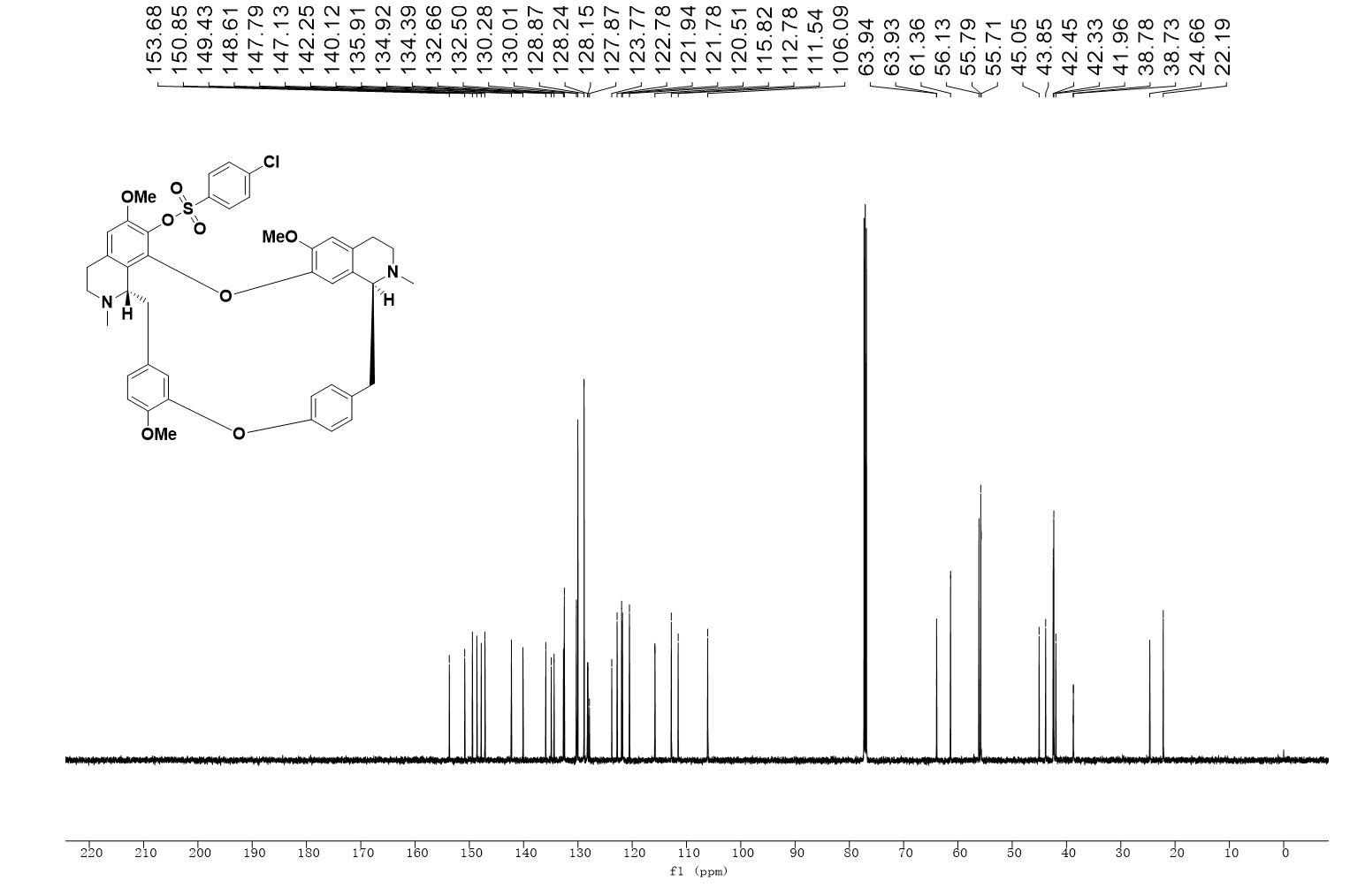


**Compound 20. 7-*O*-(4-Chlorobenzenesulfonyl)-tetrandrine: HR-ESIMS spectrum**


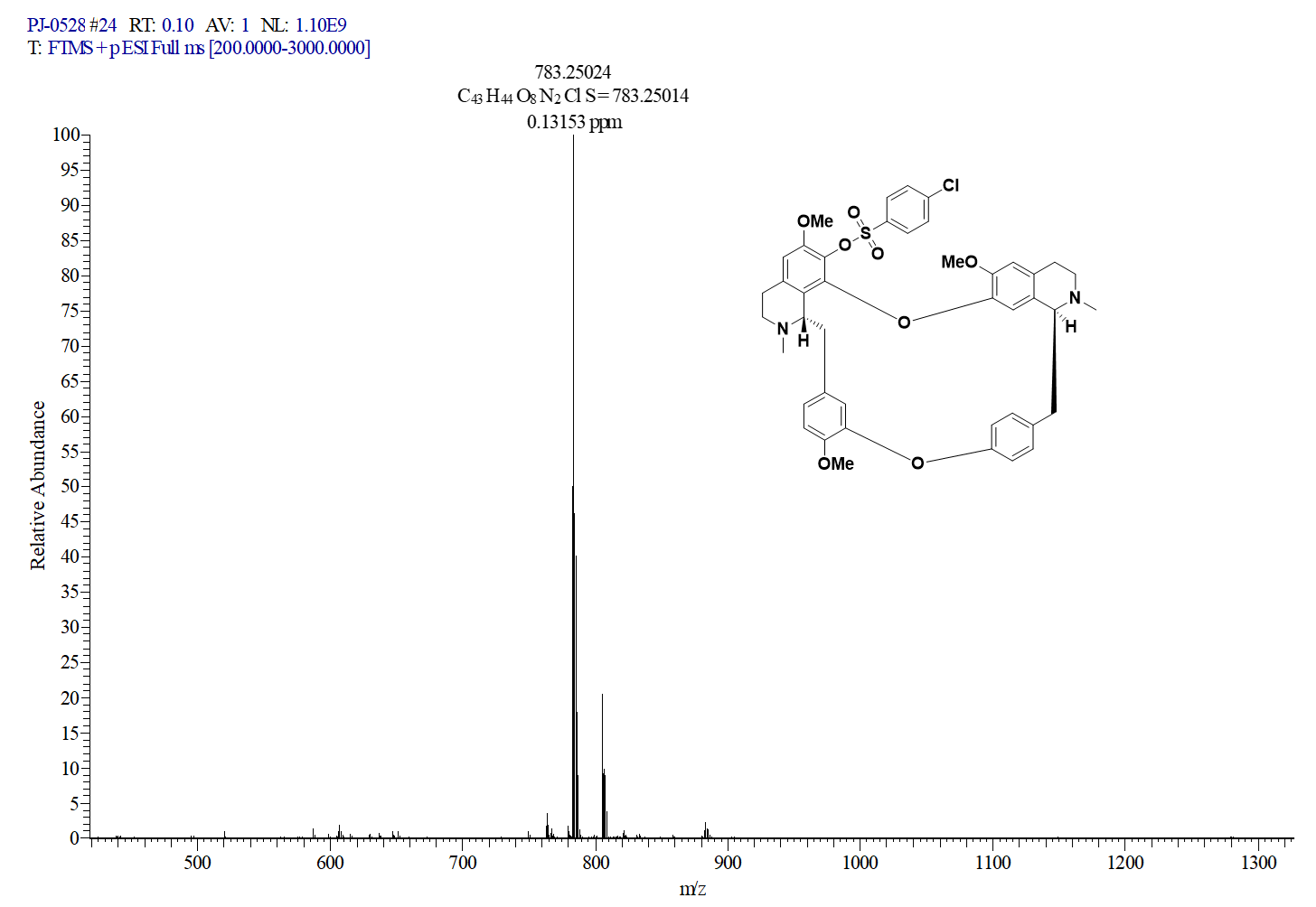


**Compound 21.** **7-*O*-(8-Quinolinesulfonyl)-tetrandrine: ^1^H-NMR spectrum**


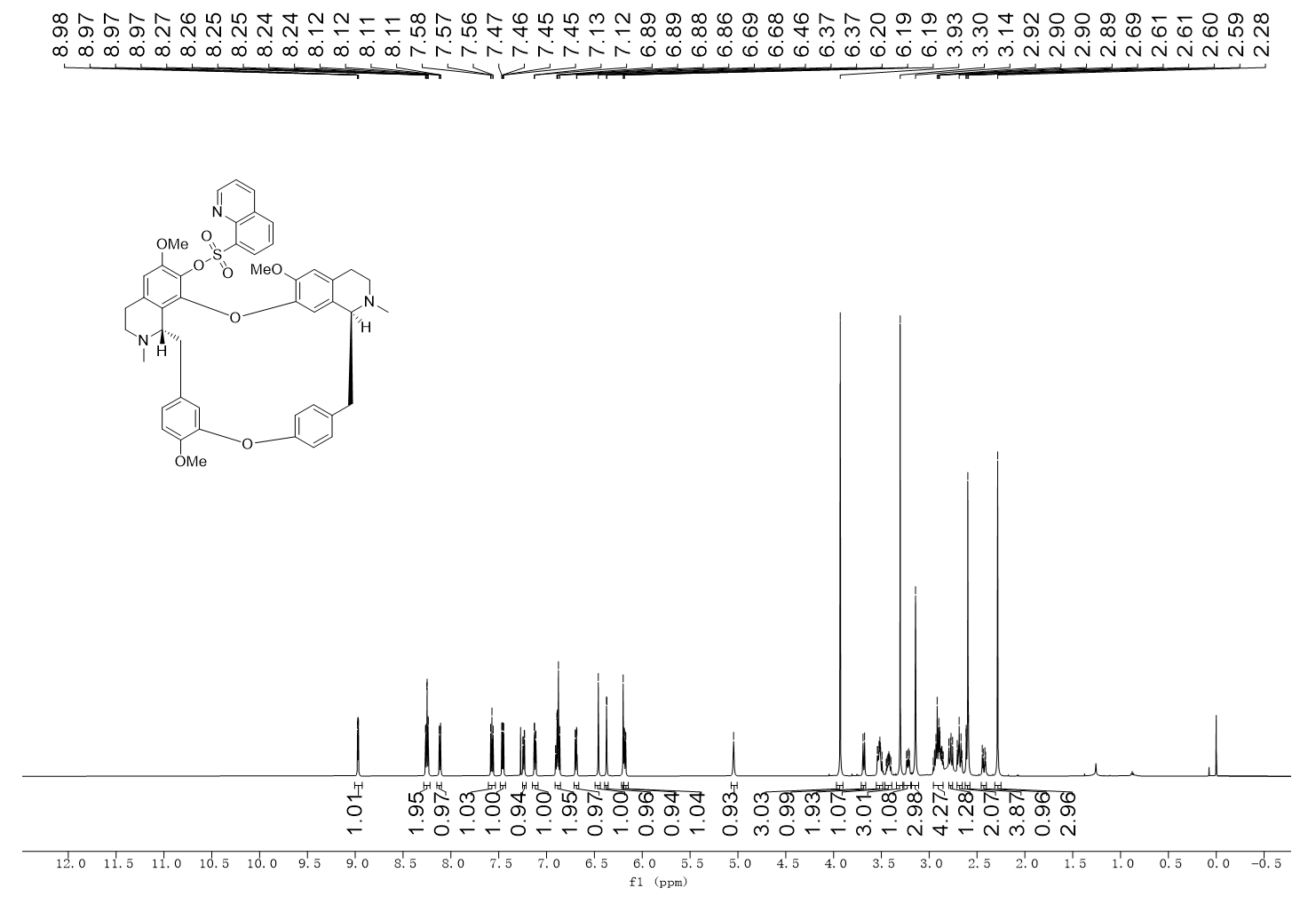


**Compound 21. 7-*O*-(8-Quinolinesulfonyl)-tetrandrine: ^13^C-NMR spectrum**


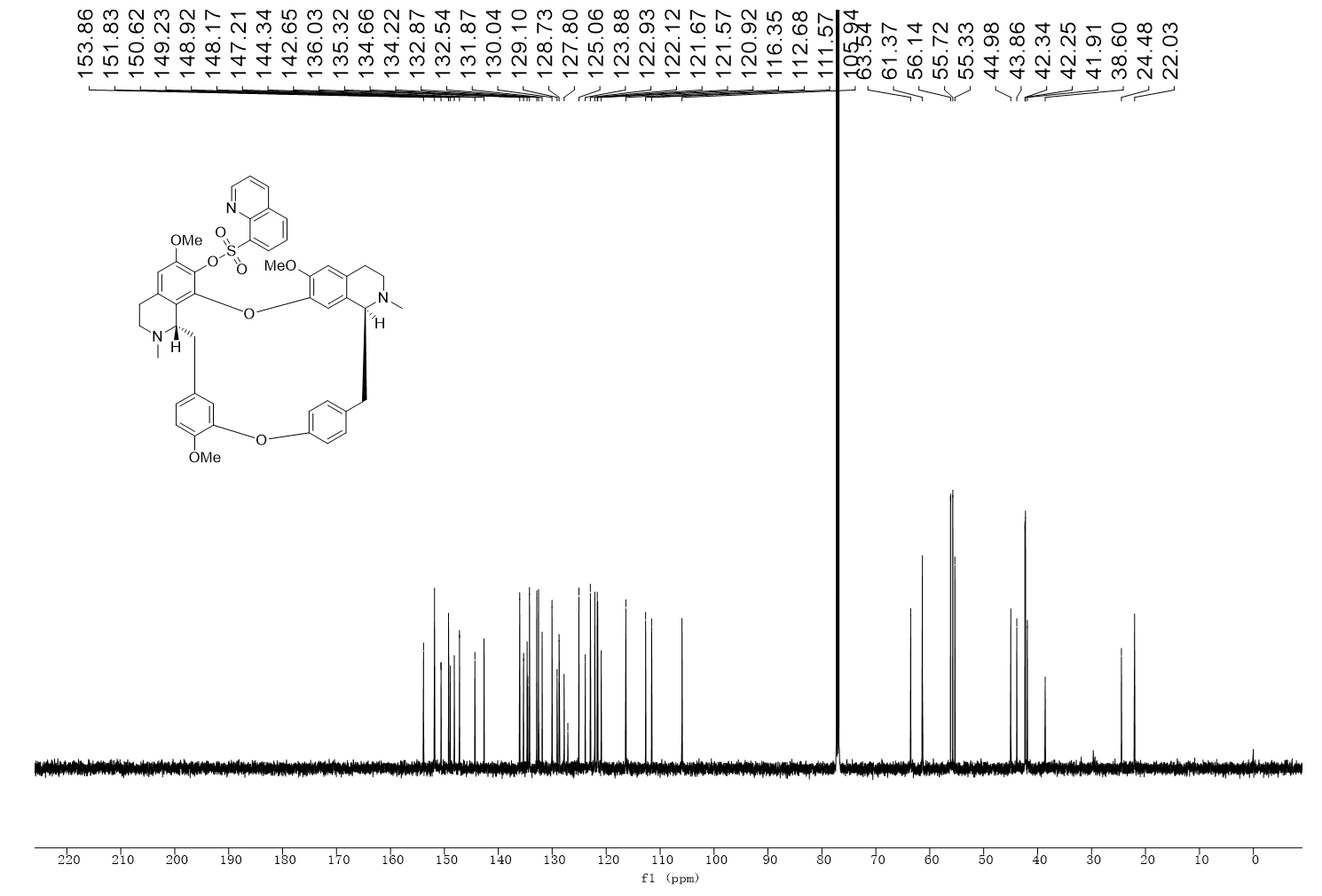


**Compound 21. 7-*O*-(8-Quinolinesulfonyl)-tetrandrine: HR-ESIMS spectrum**


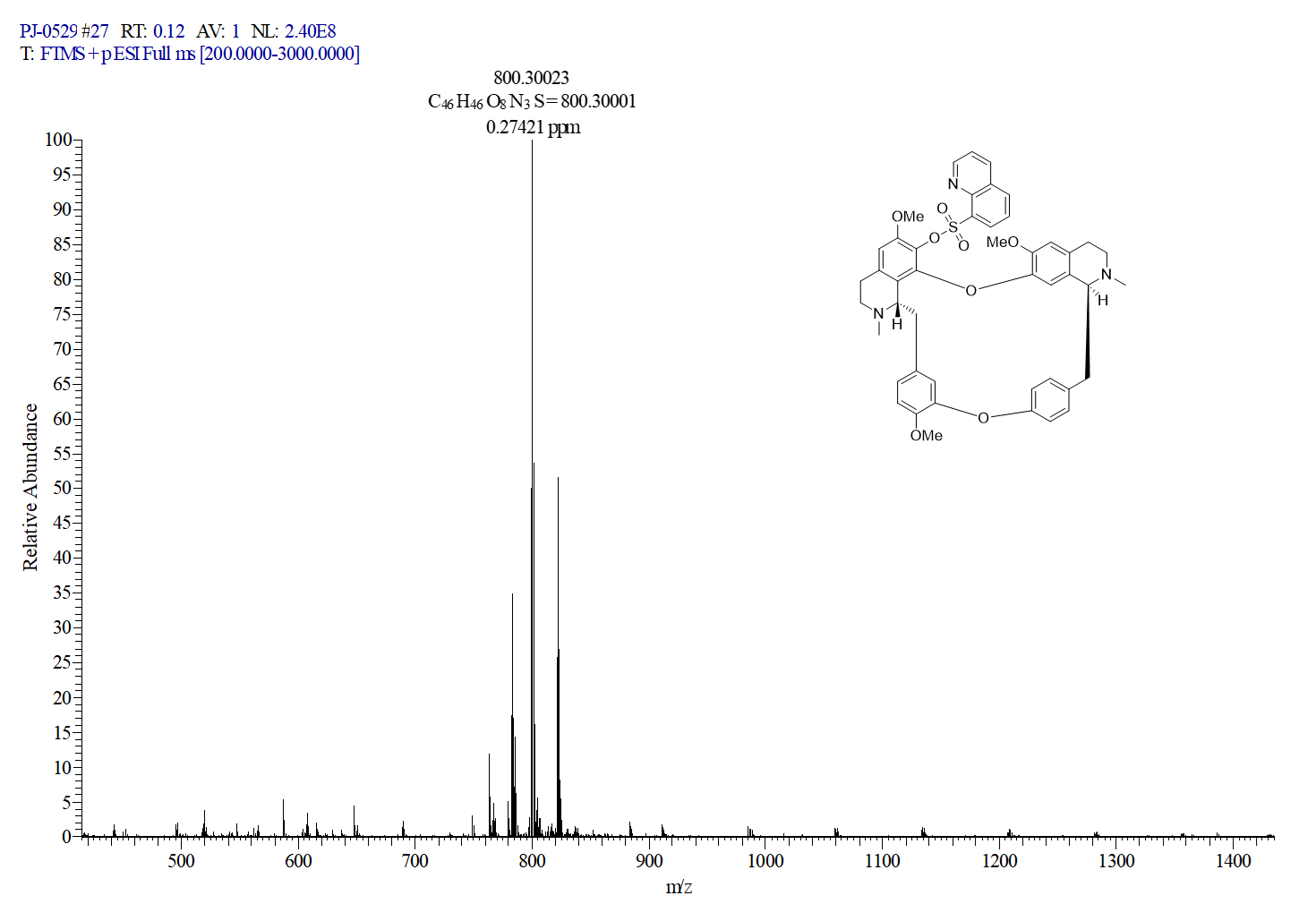


**Compound 22.** **7-*O*-(2-Bromobenzenesulfonyl)-tetrandrine: ^1^H-NMR spectrum**


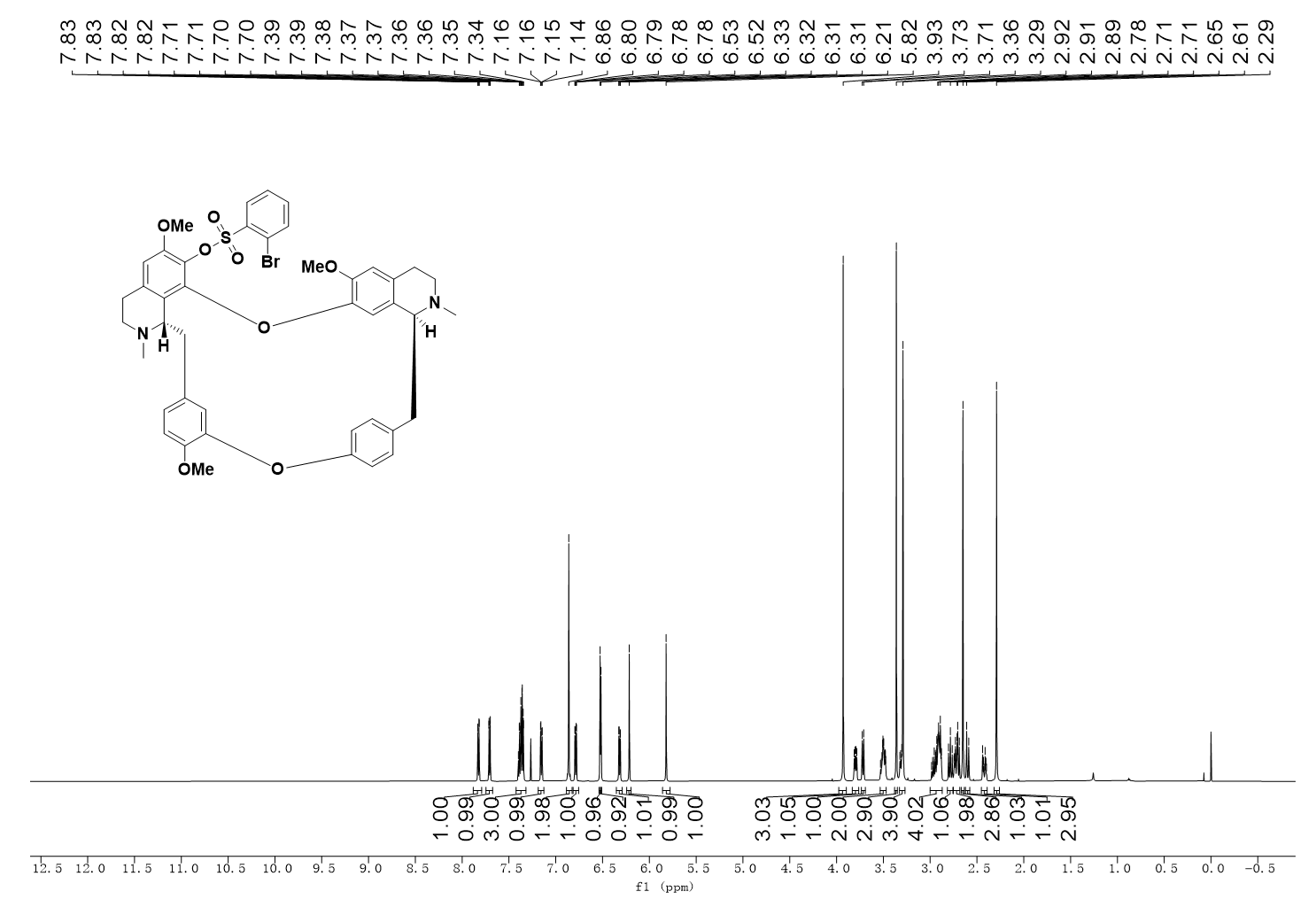


**Compound 22. 7-*O*-(2-Bromobenzenesulfonyl)-tetrandrine: ^13^C-NMR spectrum**


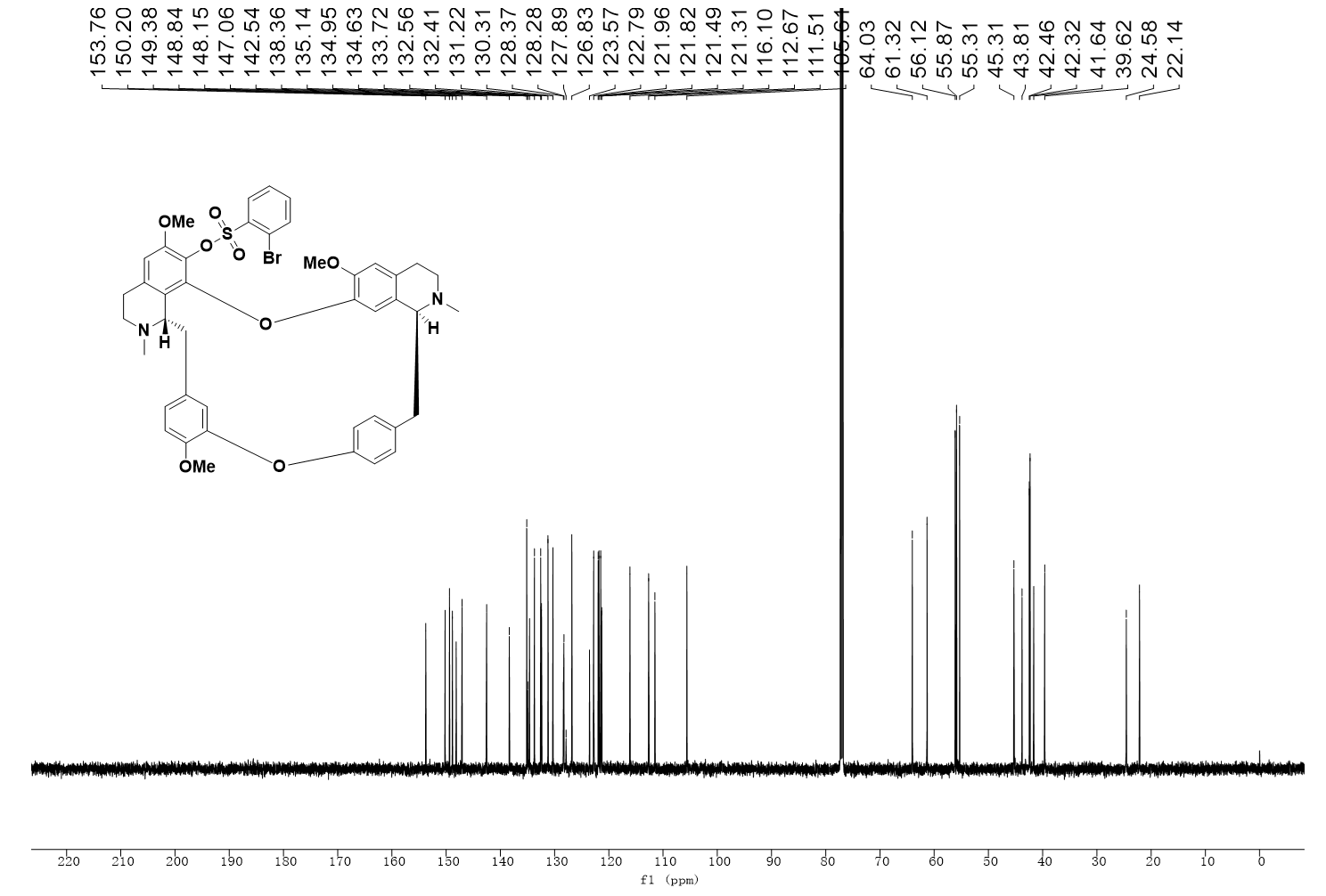


**Compound 22. 7-*O*-(2-Bromobenzenesulfonyl)-tetrandrine: HR-ESIMS spectrum**


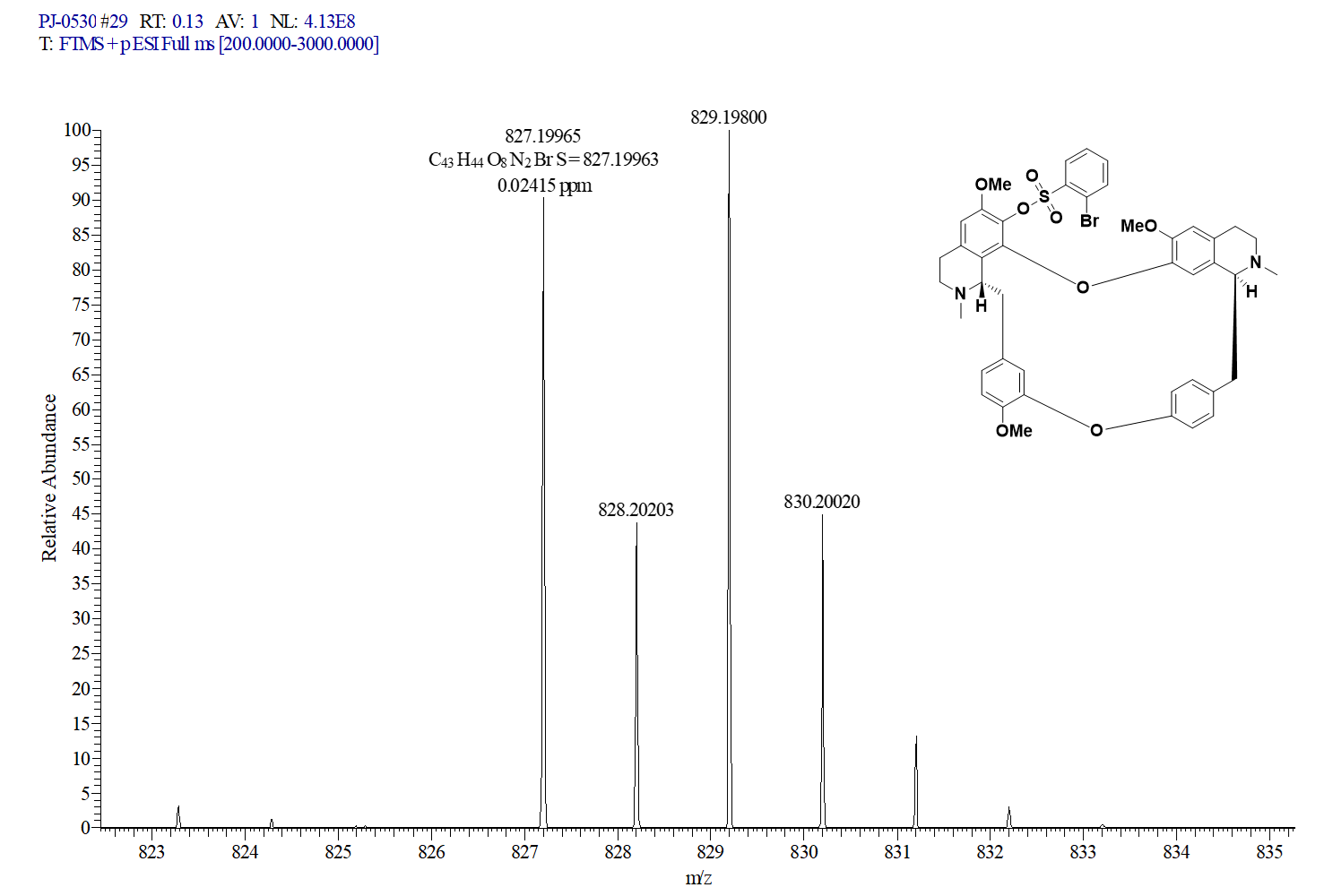


**Compound 23.** **7-*O*-(3-Bromobenzenesulfonyl)-tetrandrine: ^1^H-NMR spectrum**


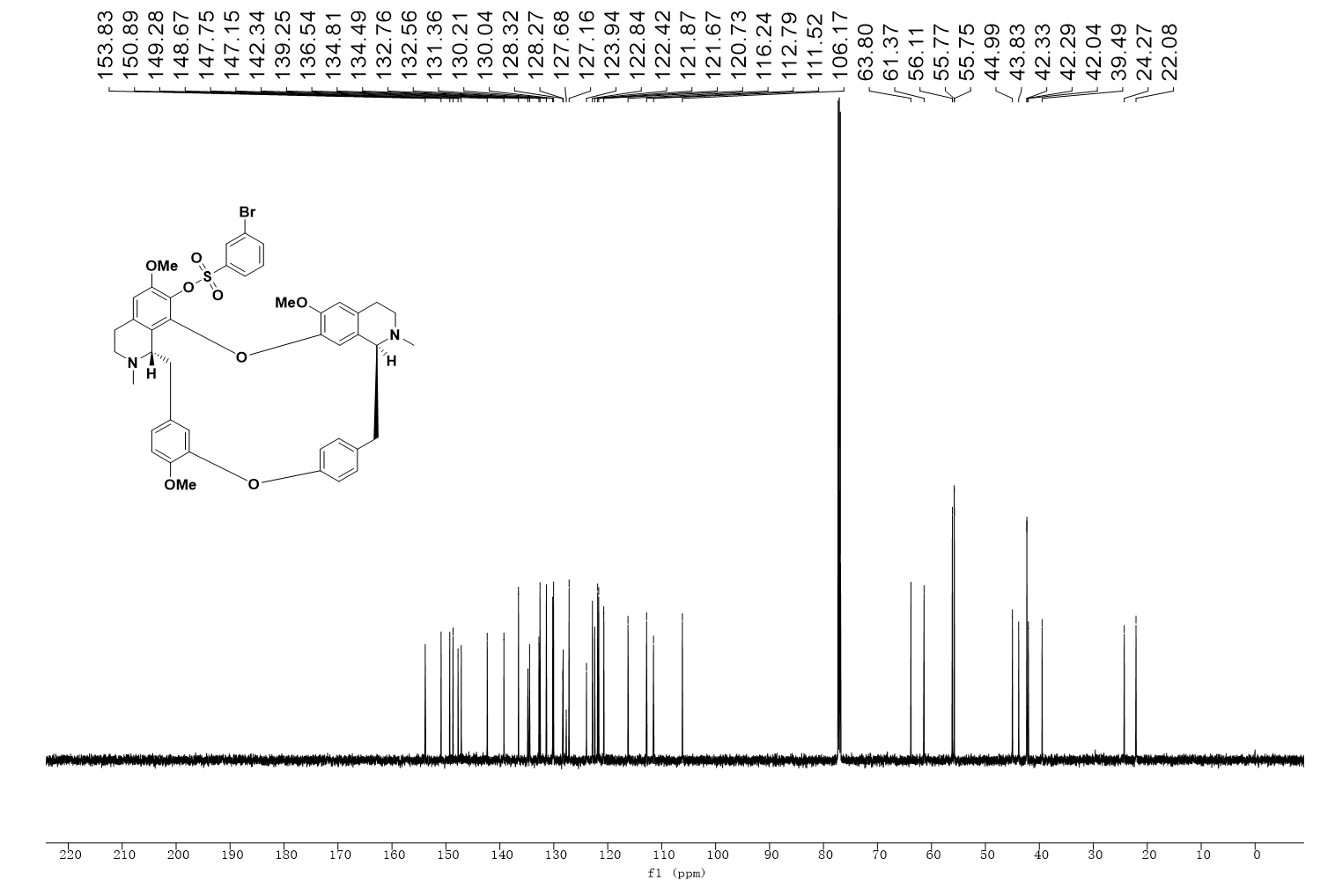


**Compound 23. 7-*O*-(3-Bromobenzenesulfonyl)-tetrandrine: ^13^C-NMR spectrum**


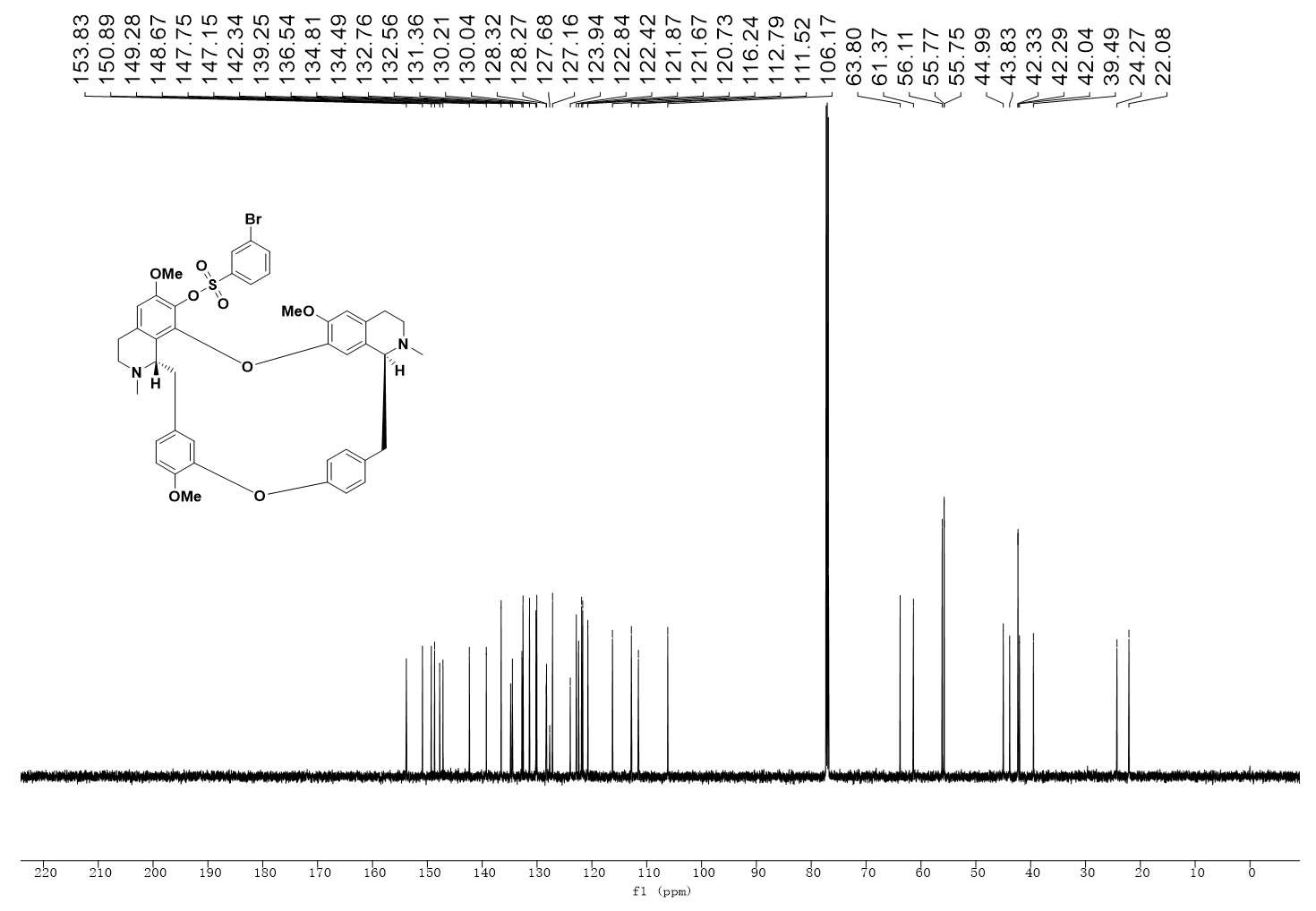


**Compound 23. 7-*O*-(3-Bromobenzenesulfonyl)-tetrandrine: HR-ESIMS spectrum**


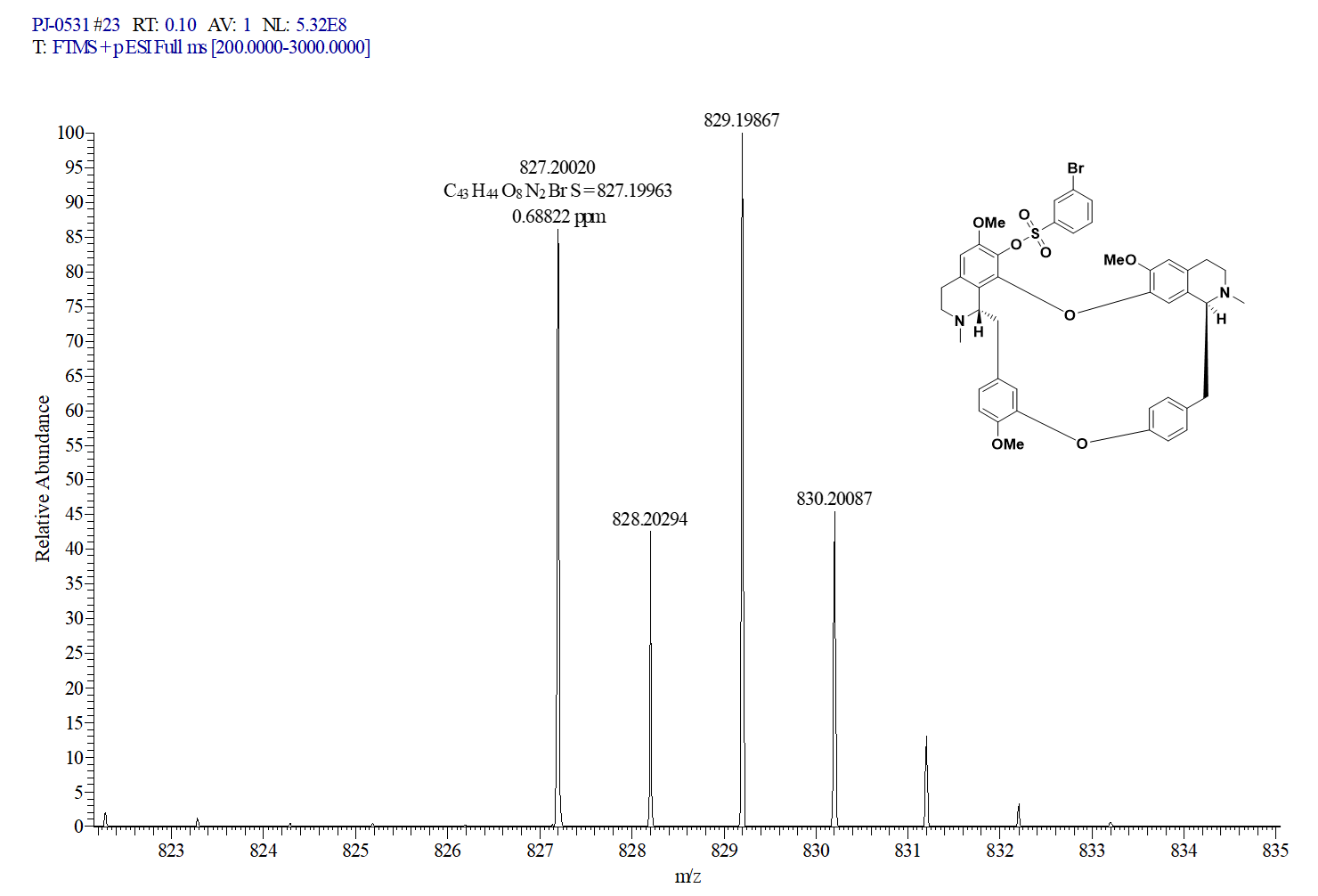


**Compound 24.** **7-*O*-(4-Bromobenzenesulfonyl)-tetrandrine: ^1^H-NMR spectrum**


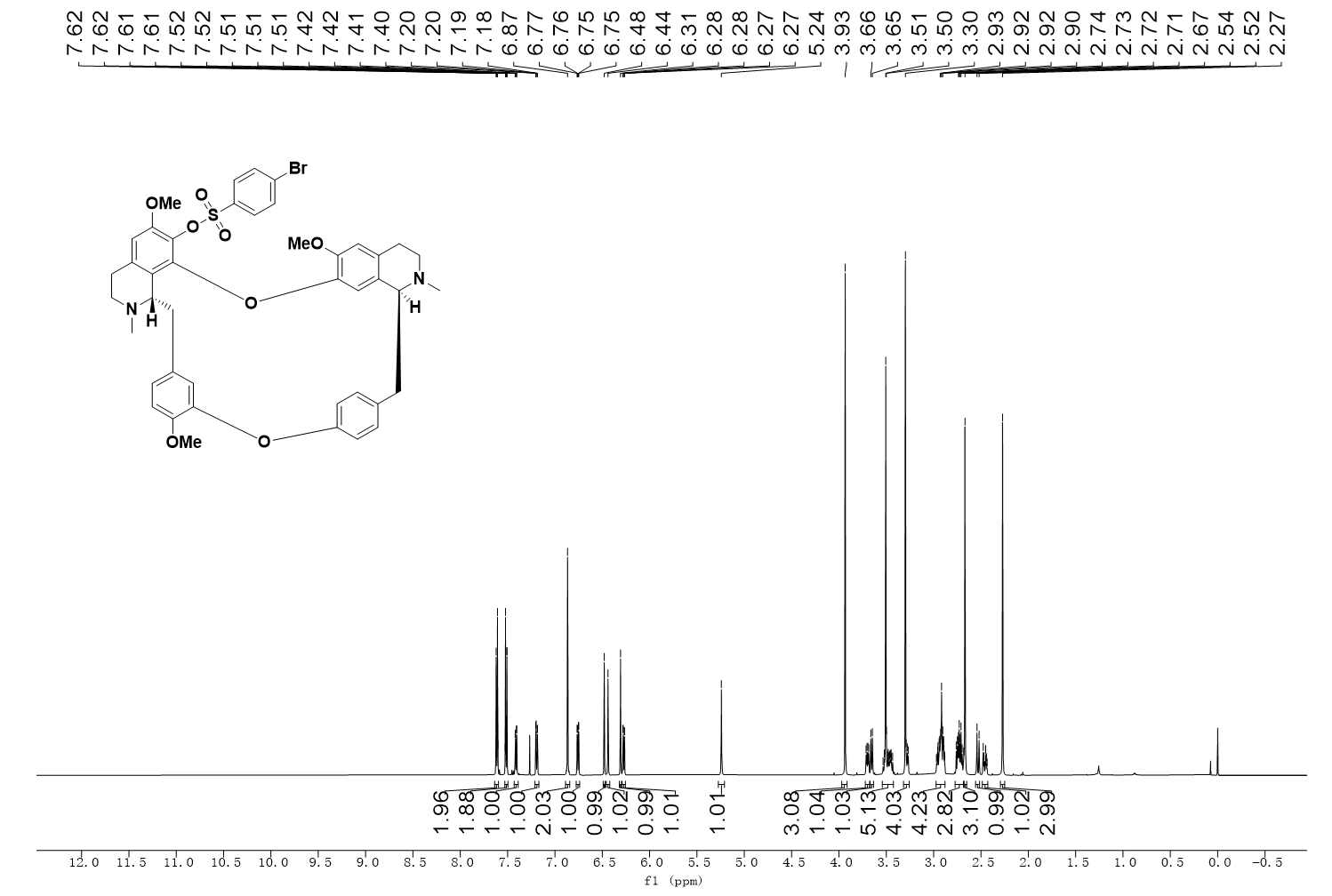


**Compound 24. 7-*O*-(4-Bromobenzenesulfonyl)-tetrandrine: ^13^C-NMR spectrum**


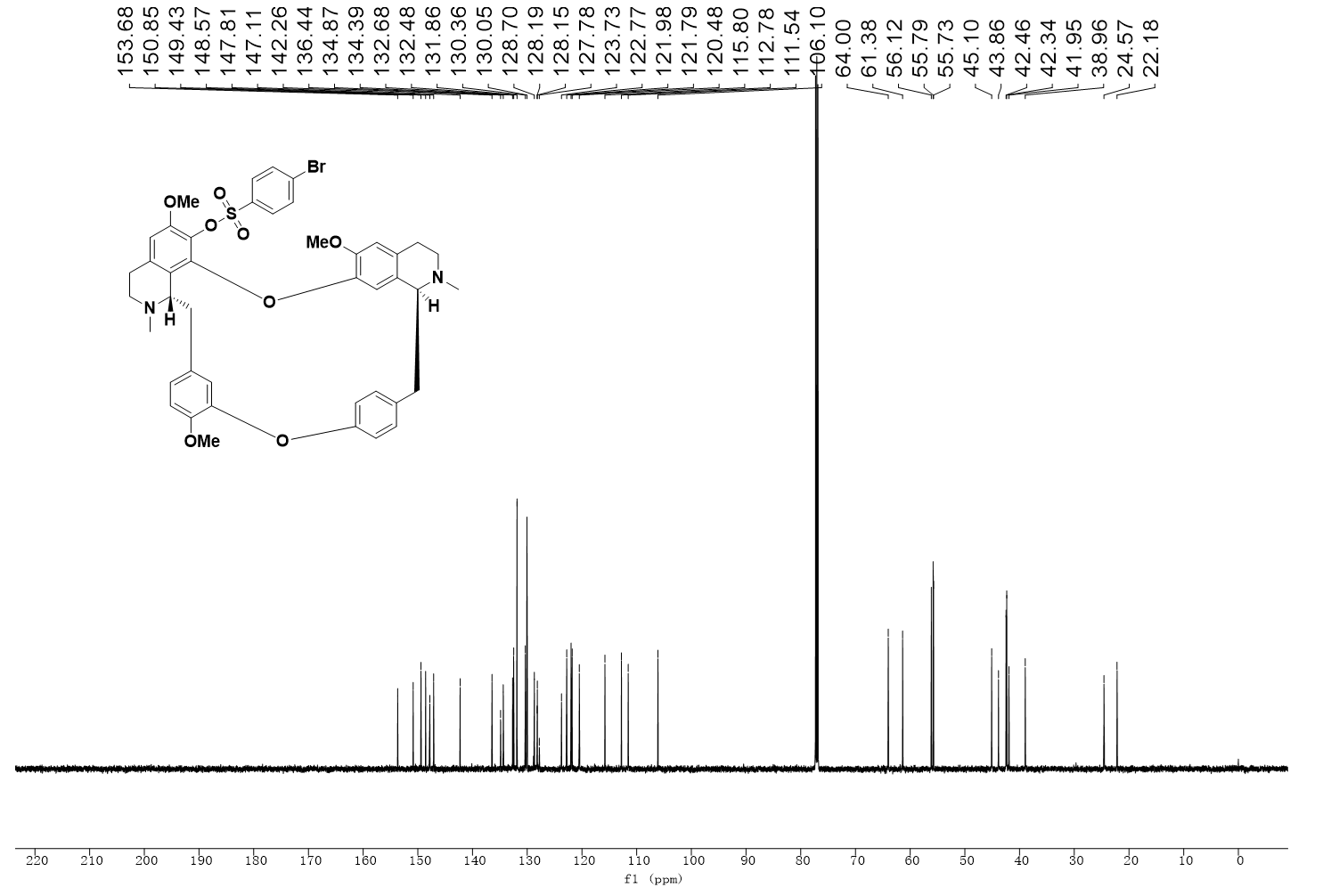


**Compound 24. 7-*O*-(4-Bromobenzenesulfonyl)-tetrandrine: HR-ESIMS spectrum**


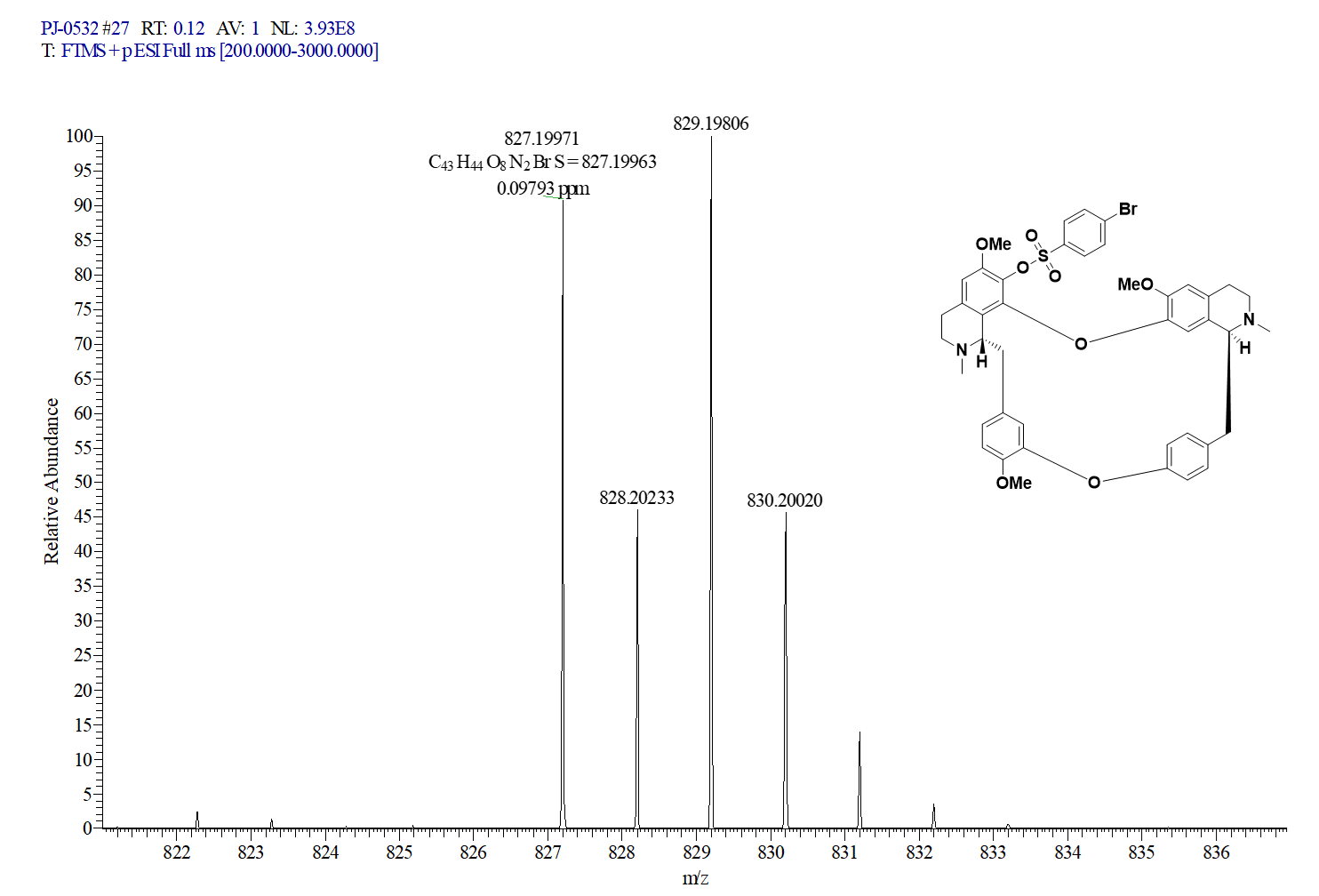


**Compound 25.** **7-*O*-(4-Acetamidobenzenesulfonyl)-tetrandrine: ^1^H-NMR spectrum**


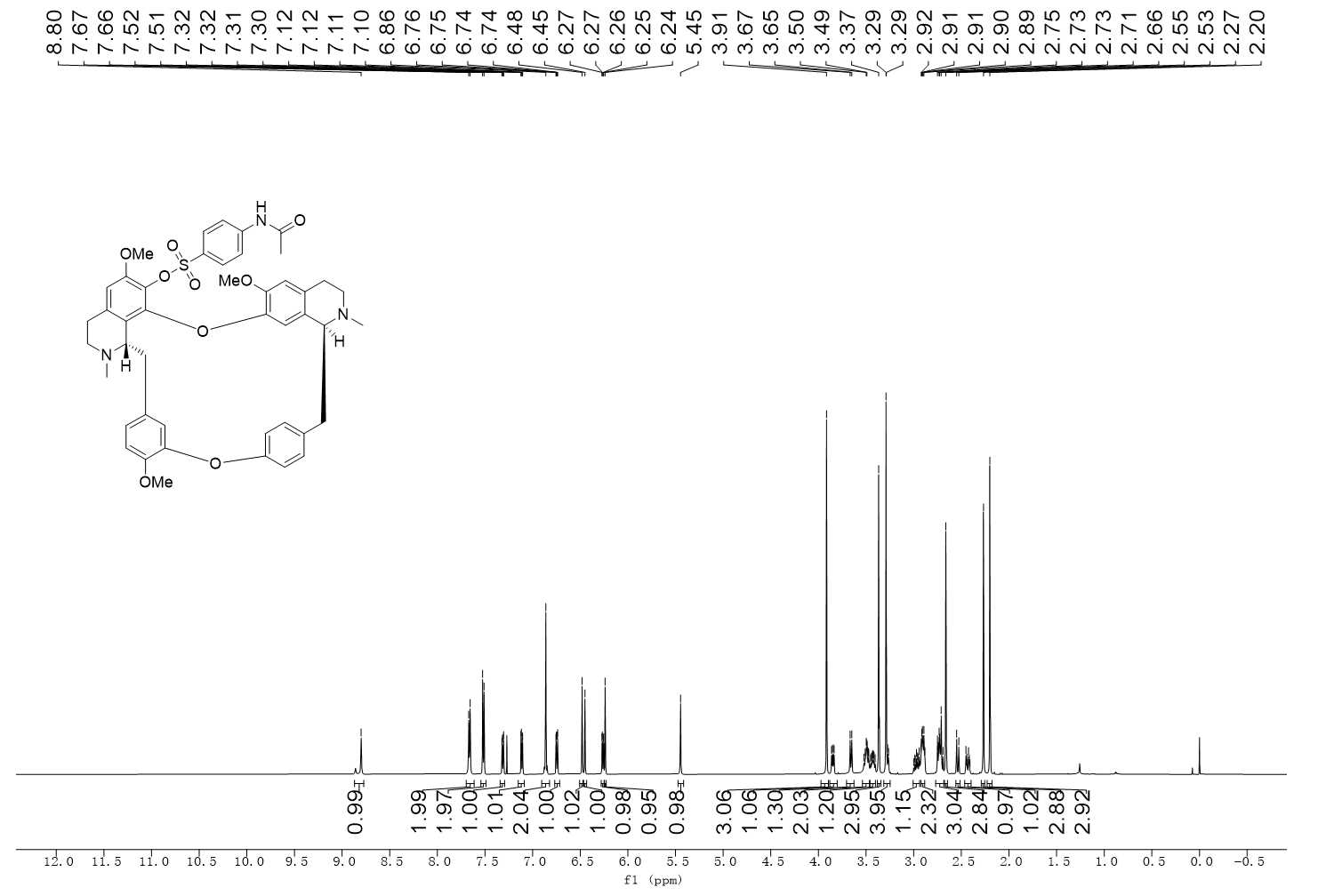


**Compound 25. 7-*O*-(4-Acetamidobenzenesulfonyl)-tetrandrine: ^13^C-NMR spectrum**


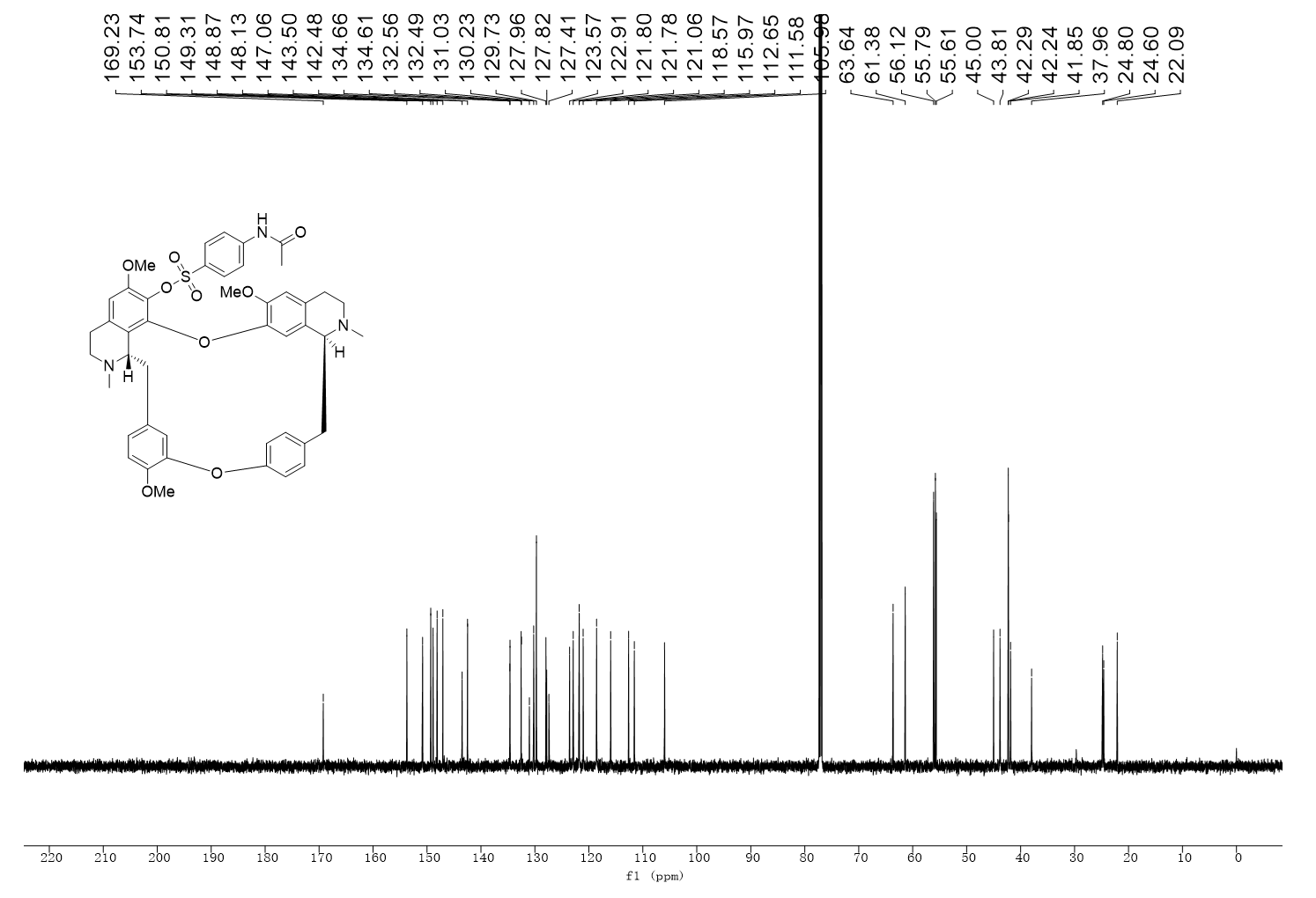


**Compound 25. 7-*O*-(4-Acetamidobenzenesulfonyl)-tetrandrine: HR-ESIMS spectrum**


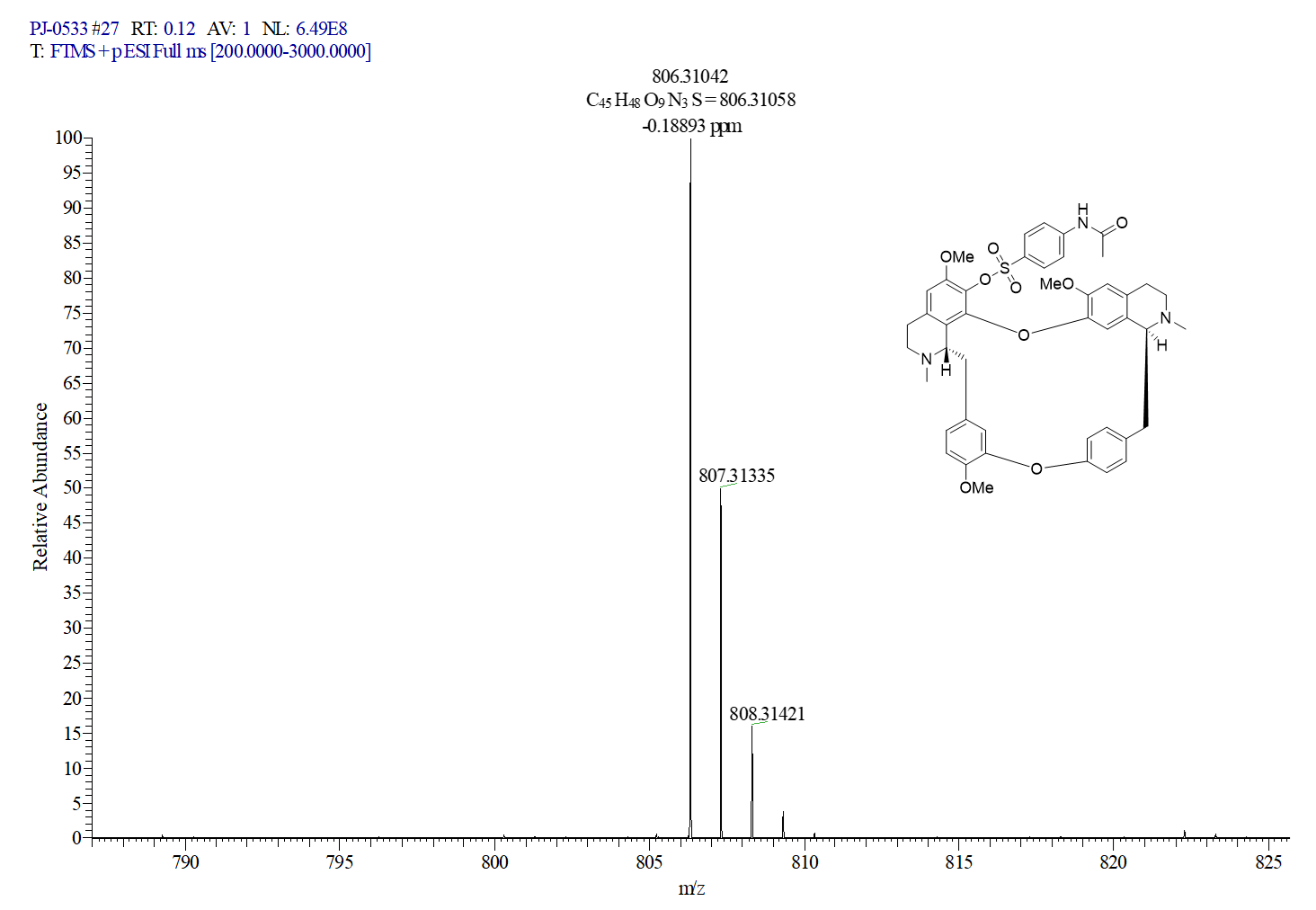


**Compound 26.** **7-*O*-(Pyridine-3-sulfonyl)-tetrandrine: ^1^H-NMR spectrum**


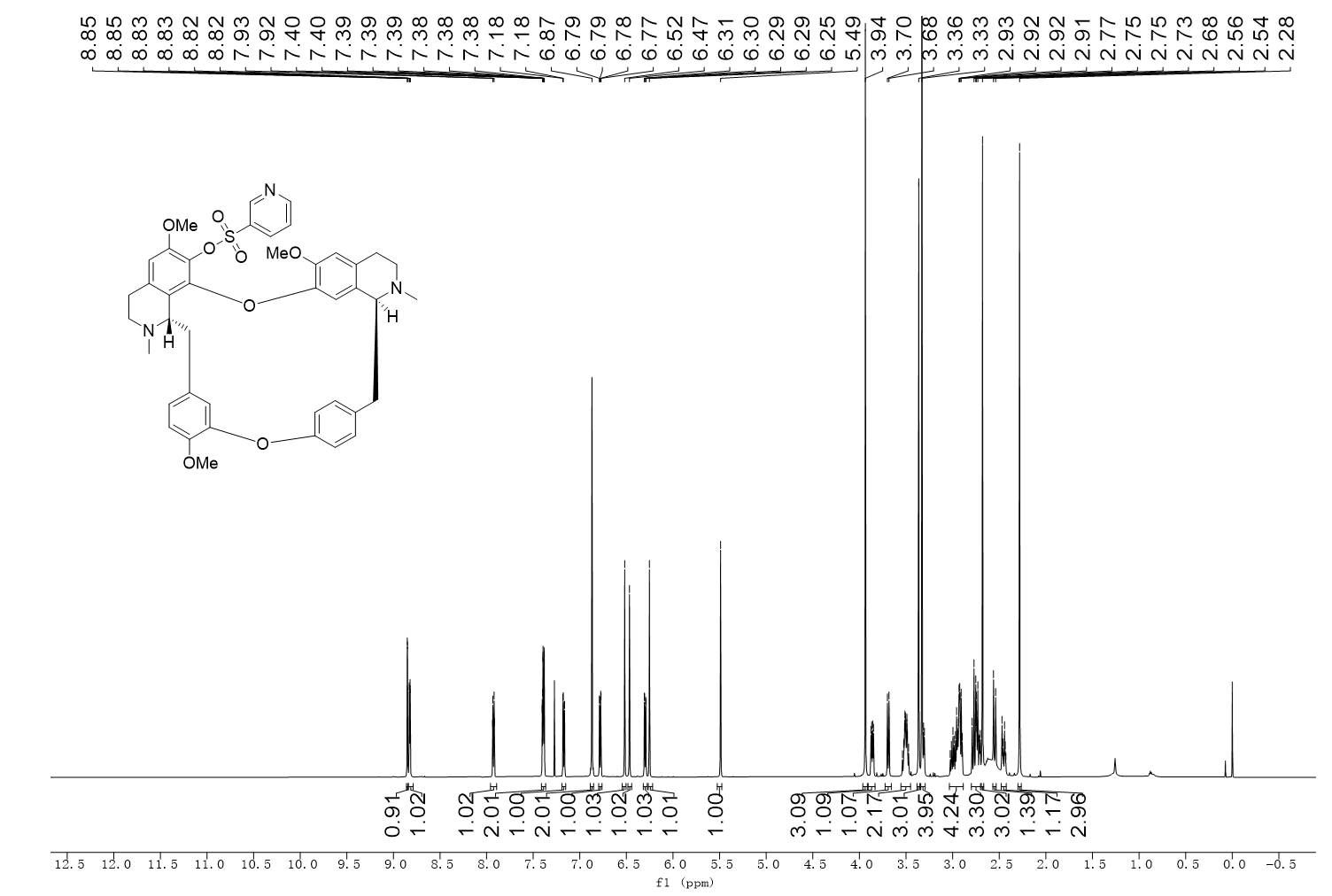


**Compound 26. 7-*O*-(Pyridine-3-sulfonyl)-tetrandrine: ^13^C-NMR spectrum**


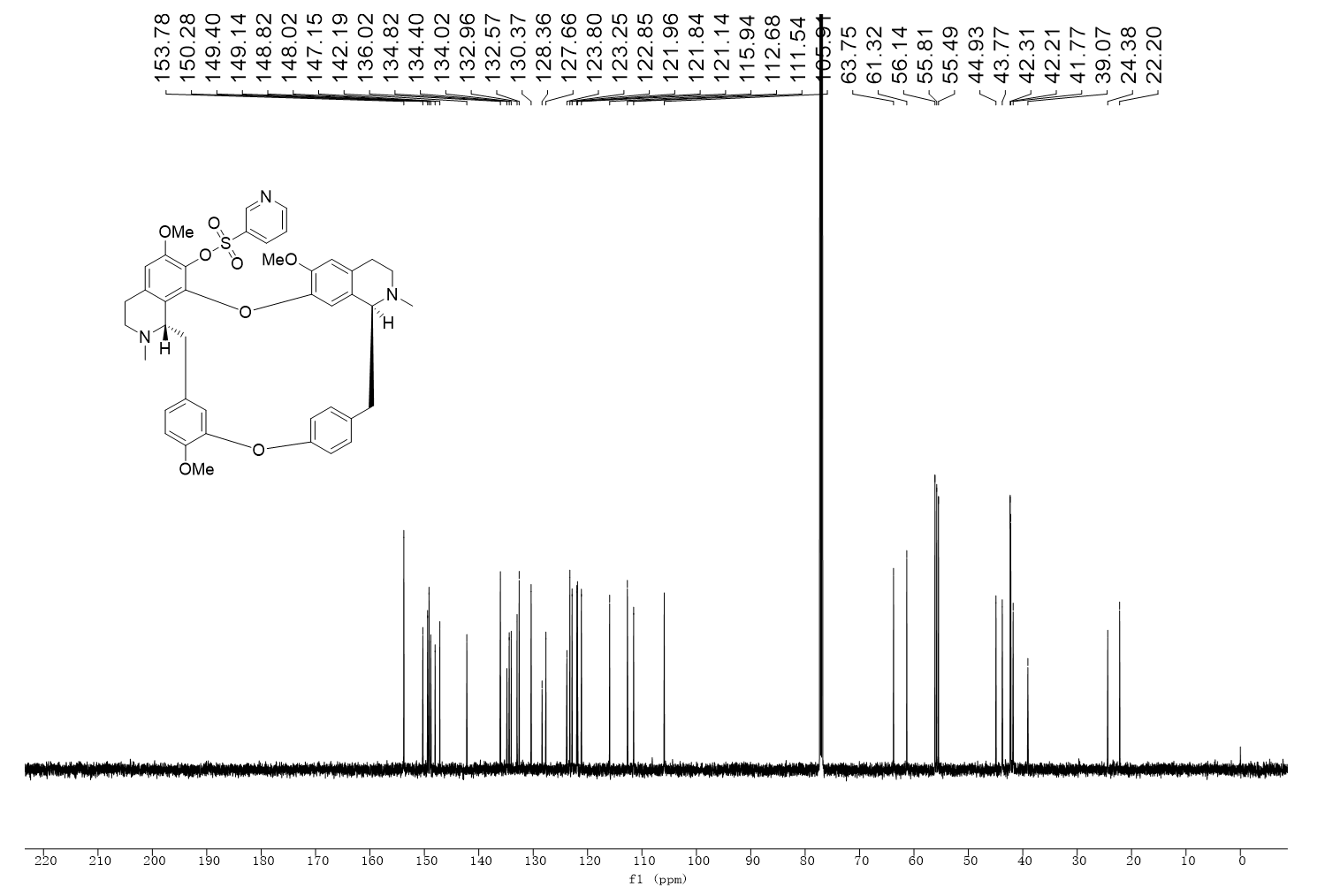


**Compound 26. 7-*O*-(Pyridine-3-sulfonyl)-tetrandrine: HR-ESIMS spectrum**


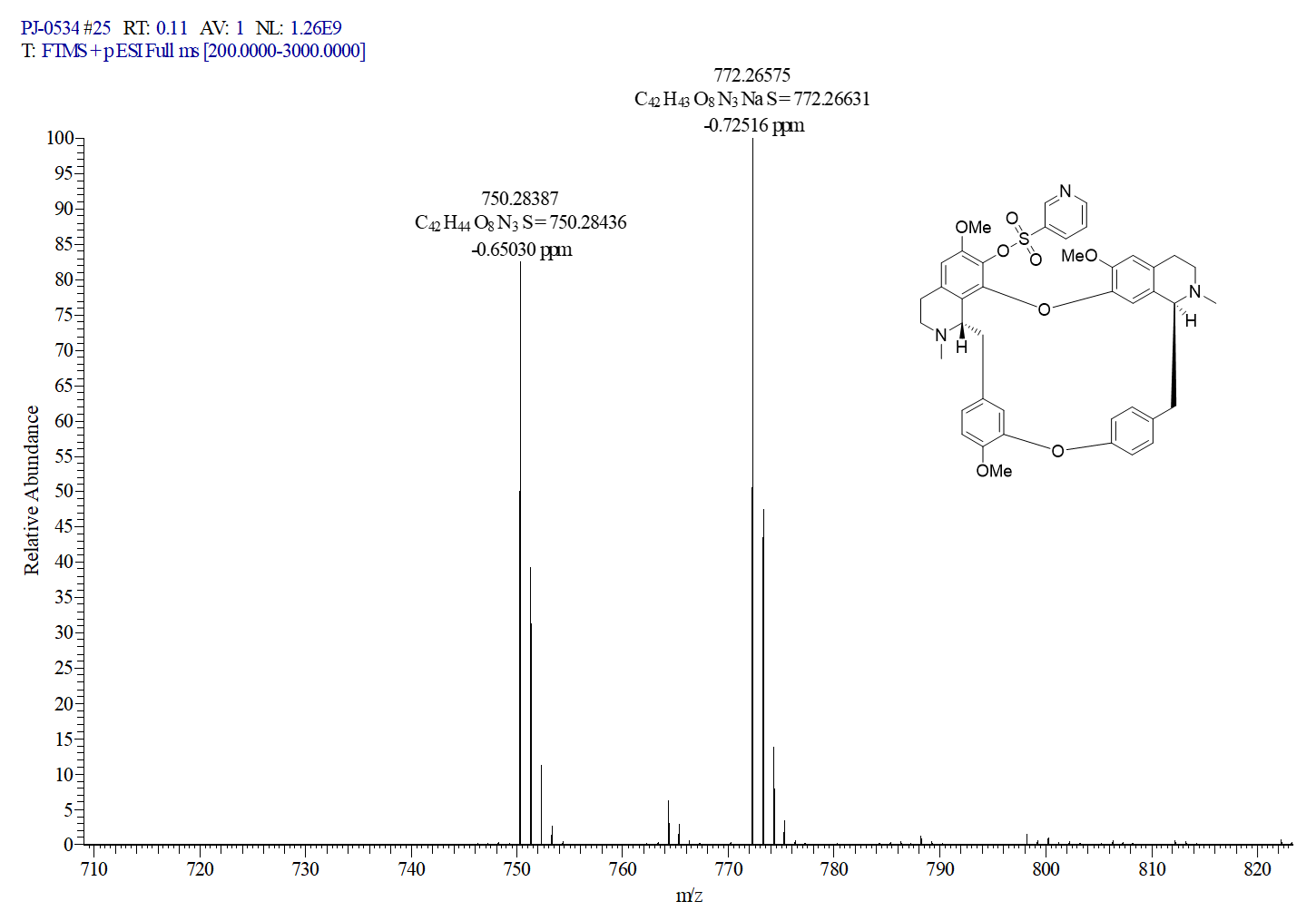


**Compound 27.** **7-*O*-(2-Chloropyridine-5-sulfonyl)-tetrandrine: ^1^H-NMR spectrum**


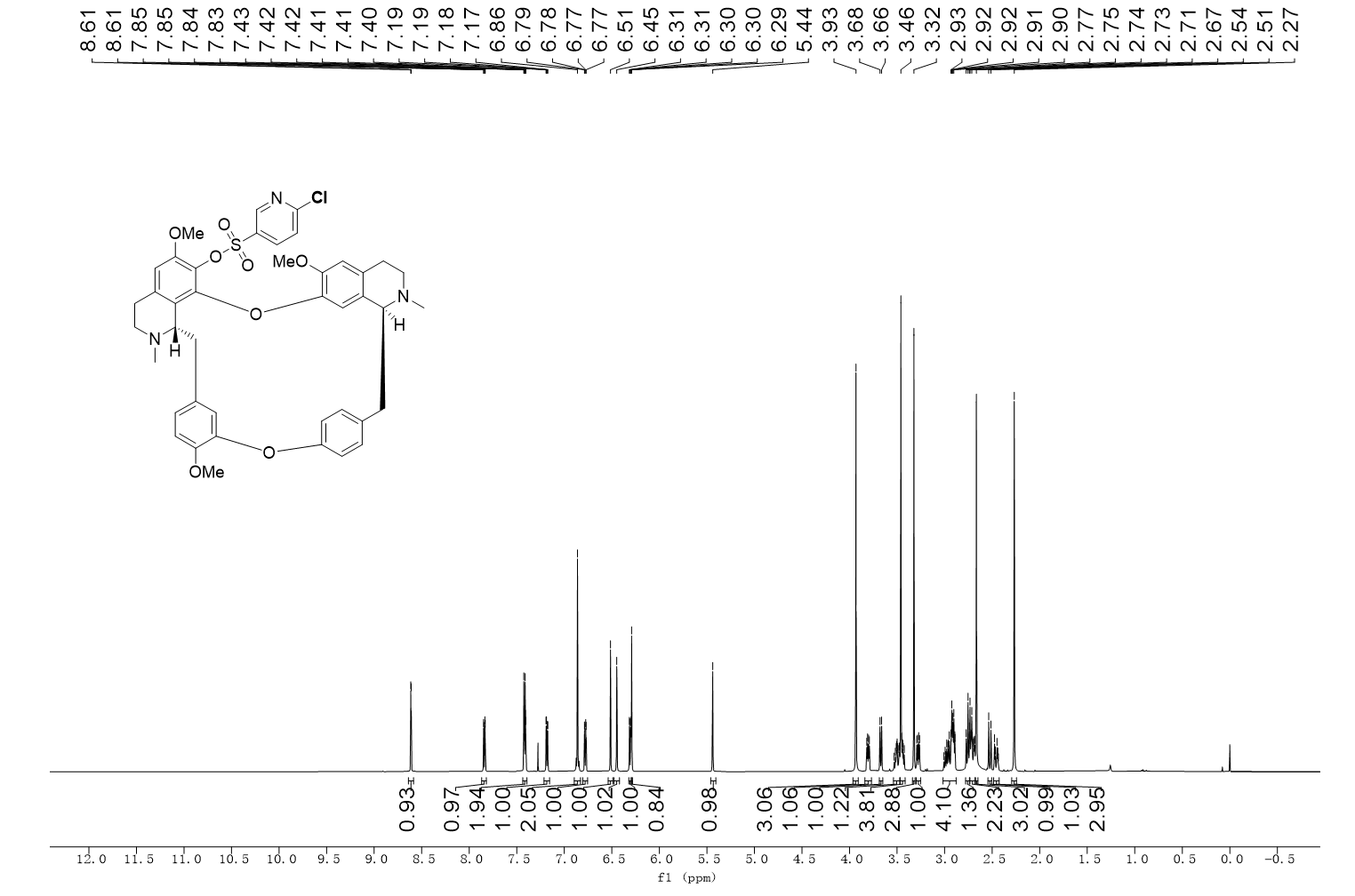


**Compound 27. 7-*O*-(2-Chloropyridine-5-sulfonyl)-tetrandrine: ^13^C-NMR spectrum**


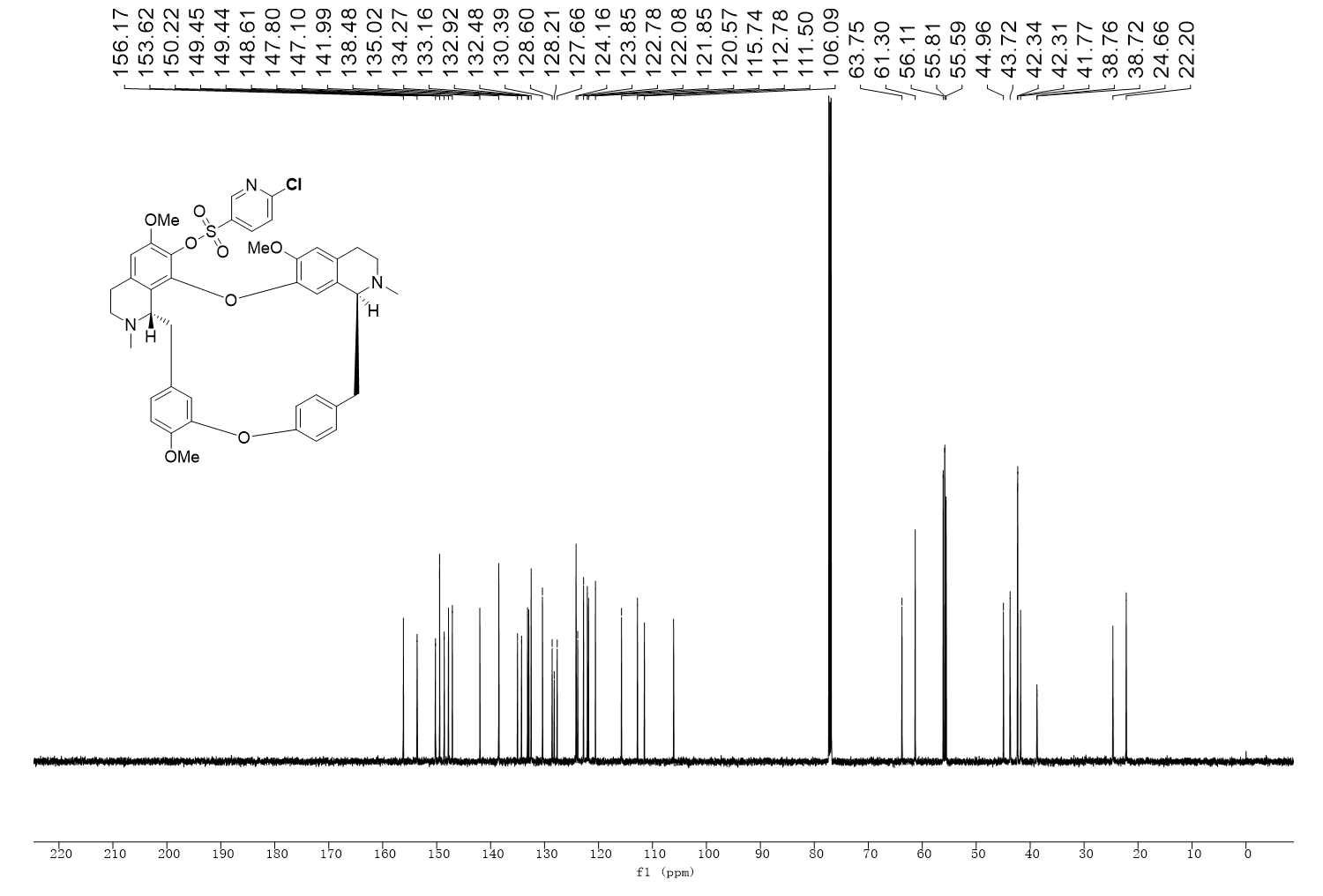


**Compound 27. 7-*O*-(2-Chloropyridine-5-sulfonyl)-tetrandrine: HR-ESIMS spectrum**


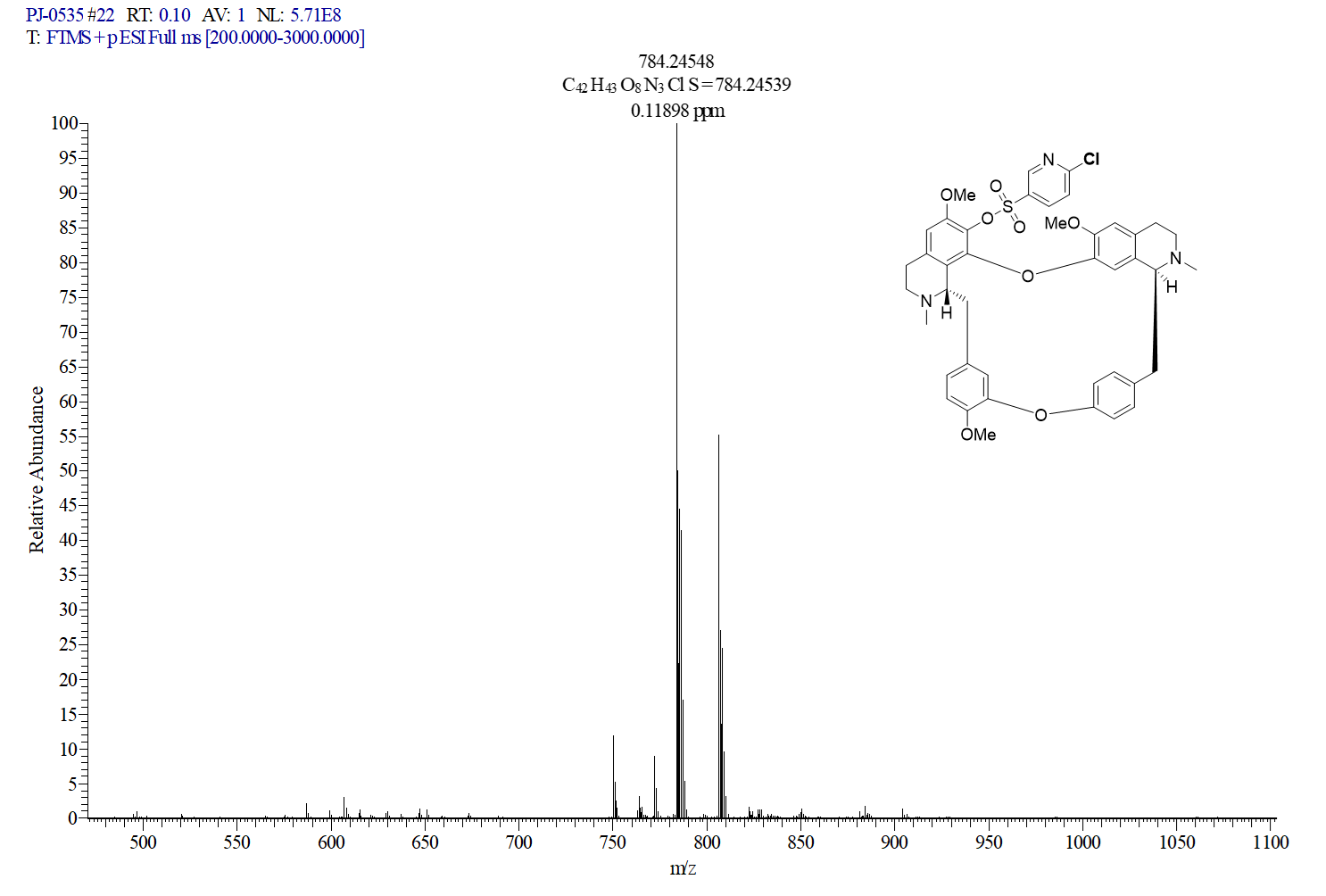


**Compound 28.** **7-*O*-(2-Thiophenesulfonyl)-tetrandrine: ^1^H-NMR spectrum**


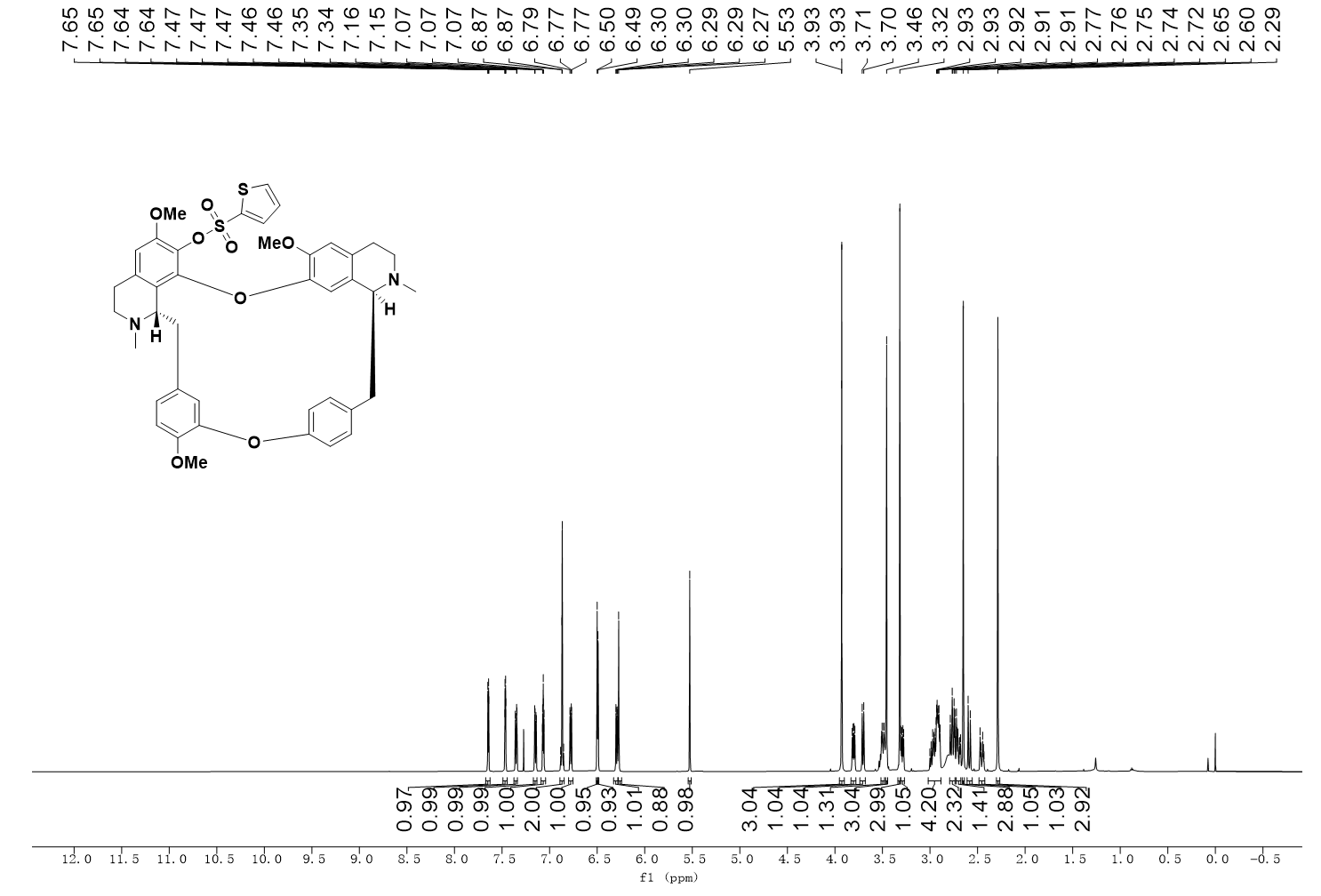


**Compound 28. 7-*O*-(2-Thiophenesulfonyl)-tetrandrine: ^13^C-NMR spectrum**


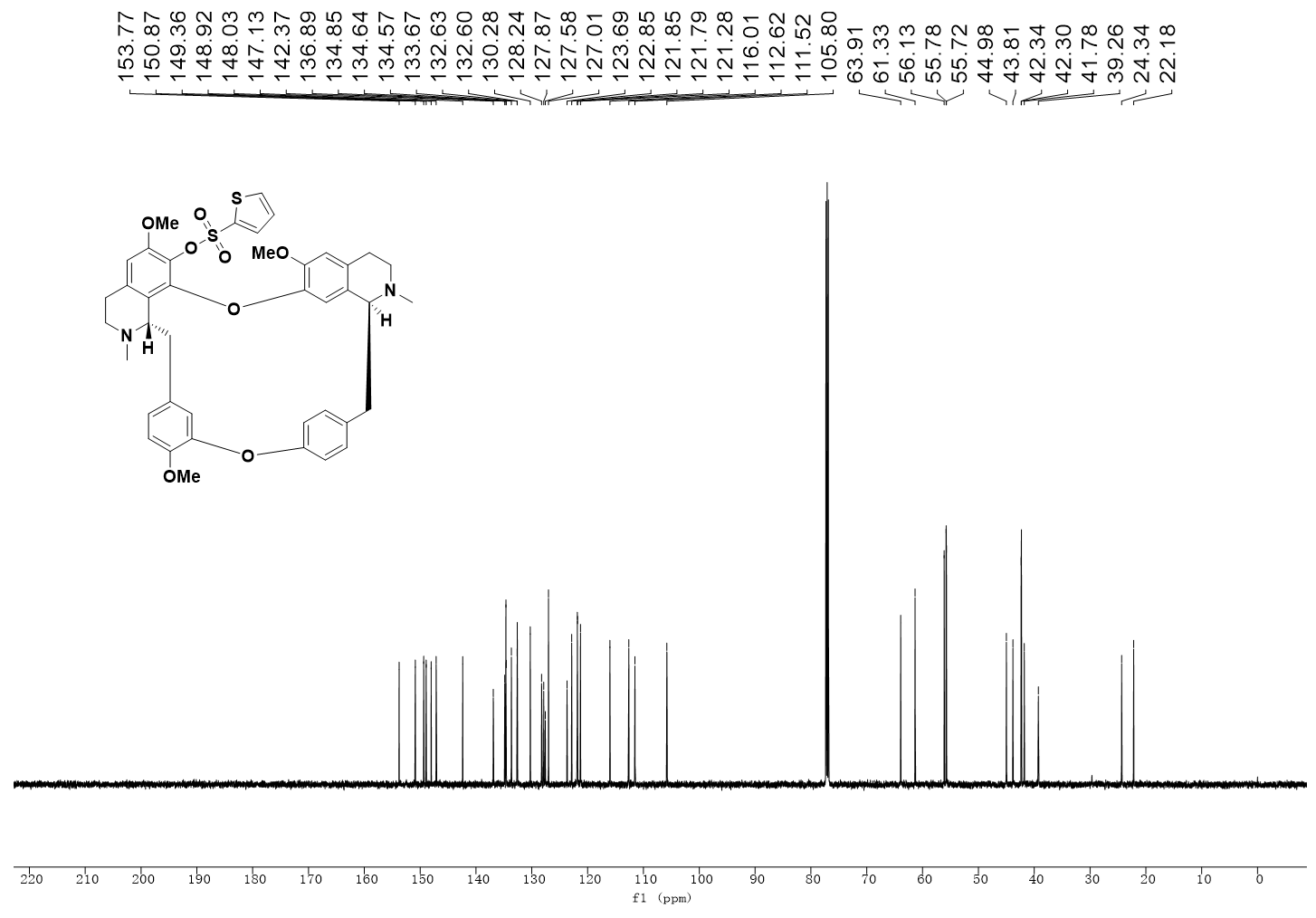


**Compound 28. 7-*O*-(2-Thiophenesulfonyl)-tetrandrine: HR-ESIMS spectrum**


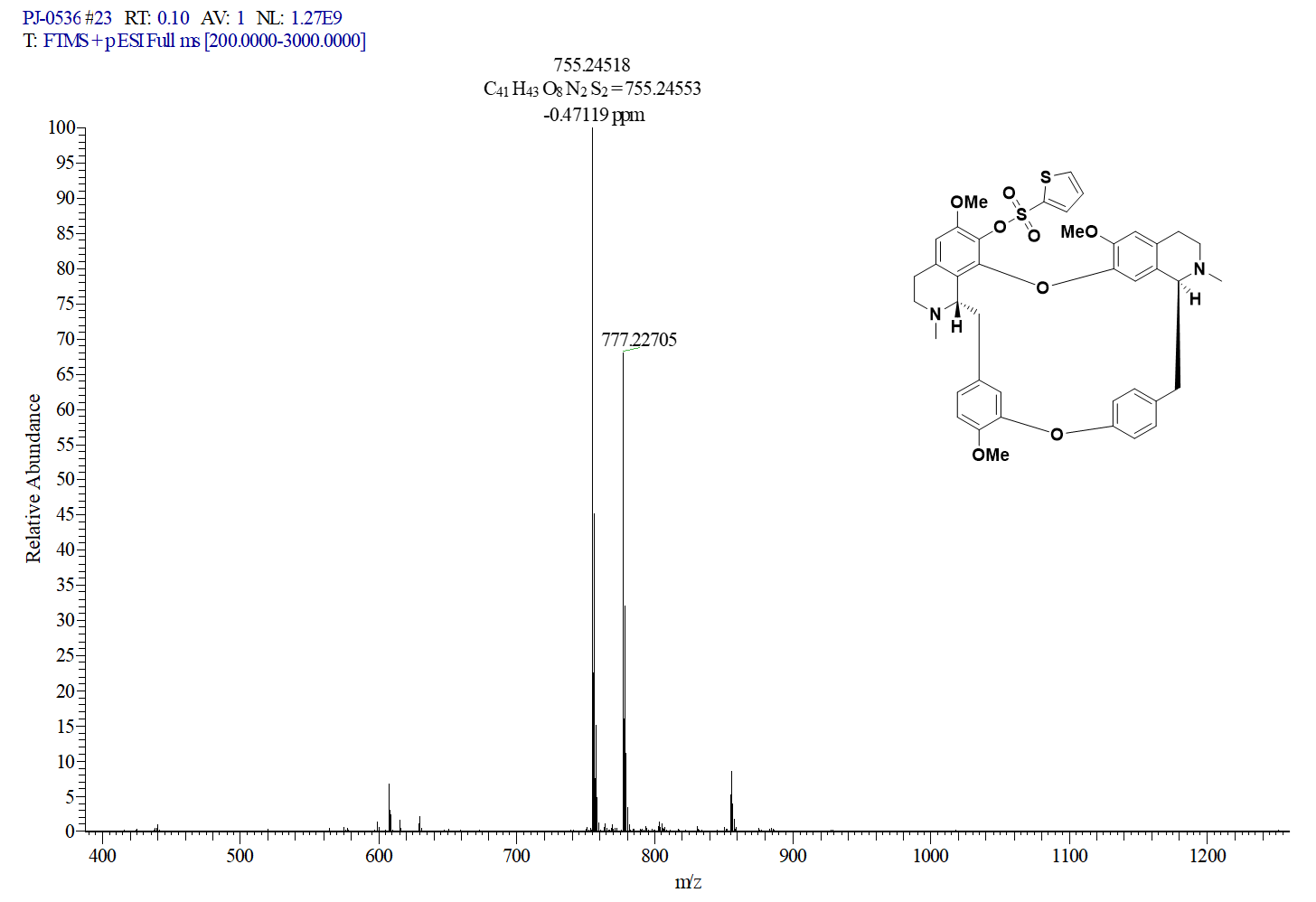


**Compound 29.** **7-*O*-(5-Chlorothiophene-2-sulfonyl)-tetrandrine: ^1^H-NMR spectrum**


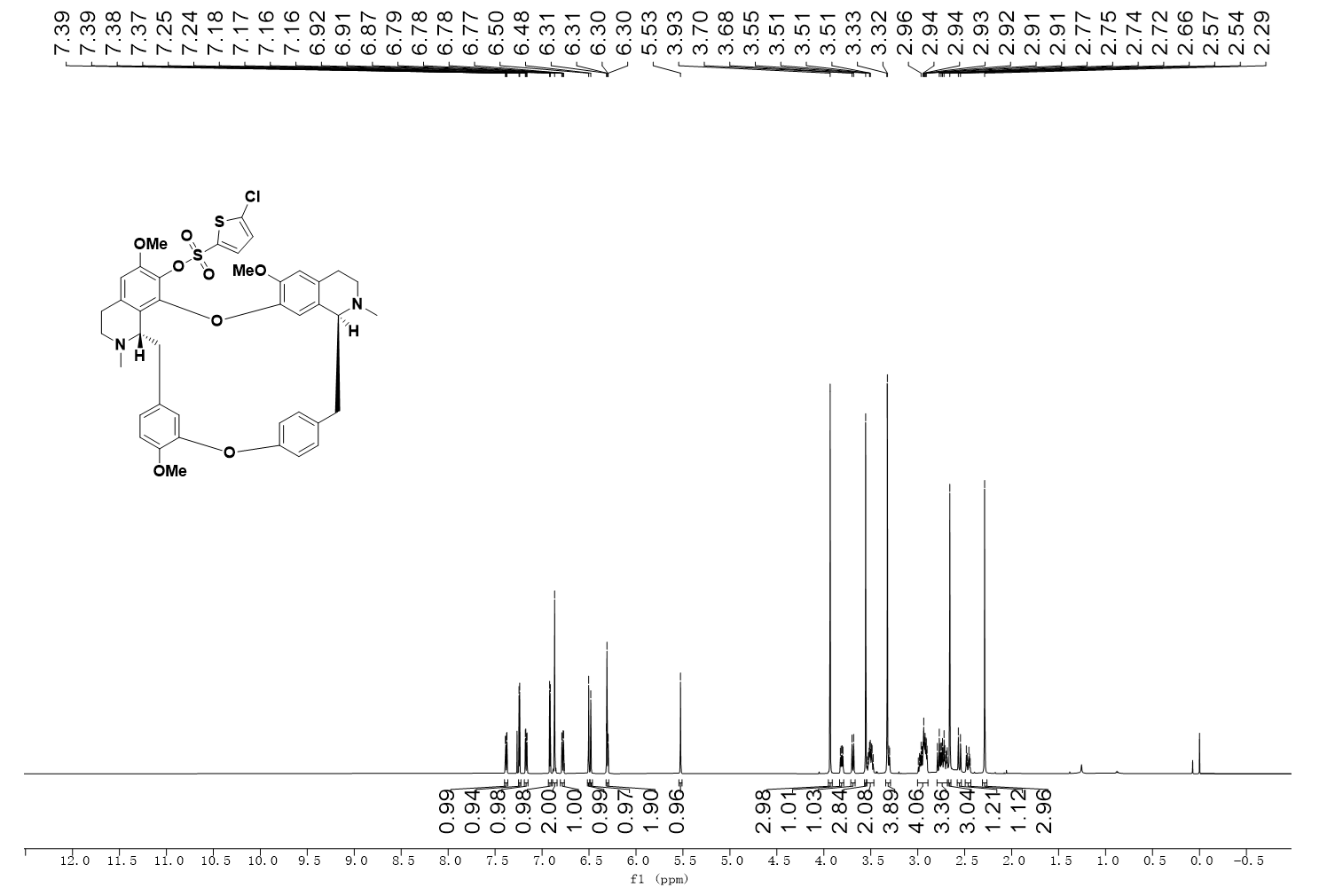


**Compound 29. 7-*O*-(5-Chlorothiophene-2-sulfonyl)-tetrandrine: ^13^C-NMR spectrum**


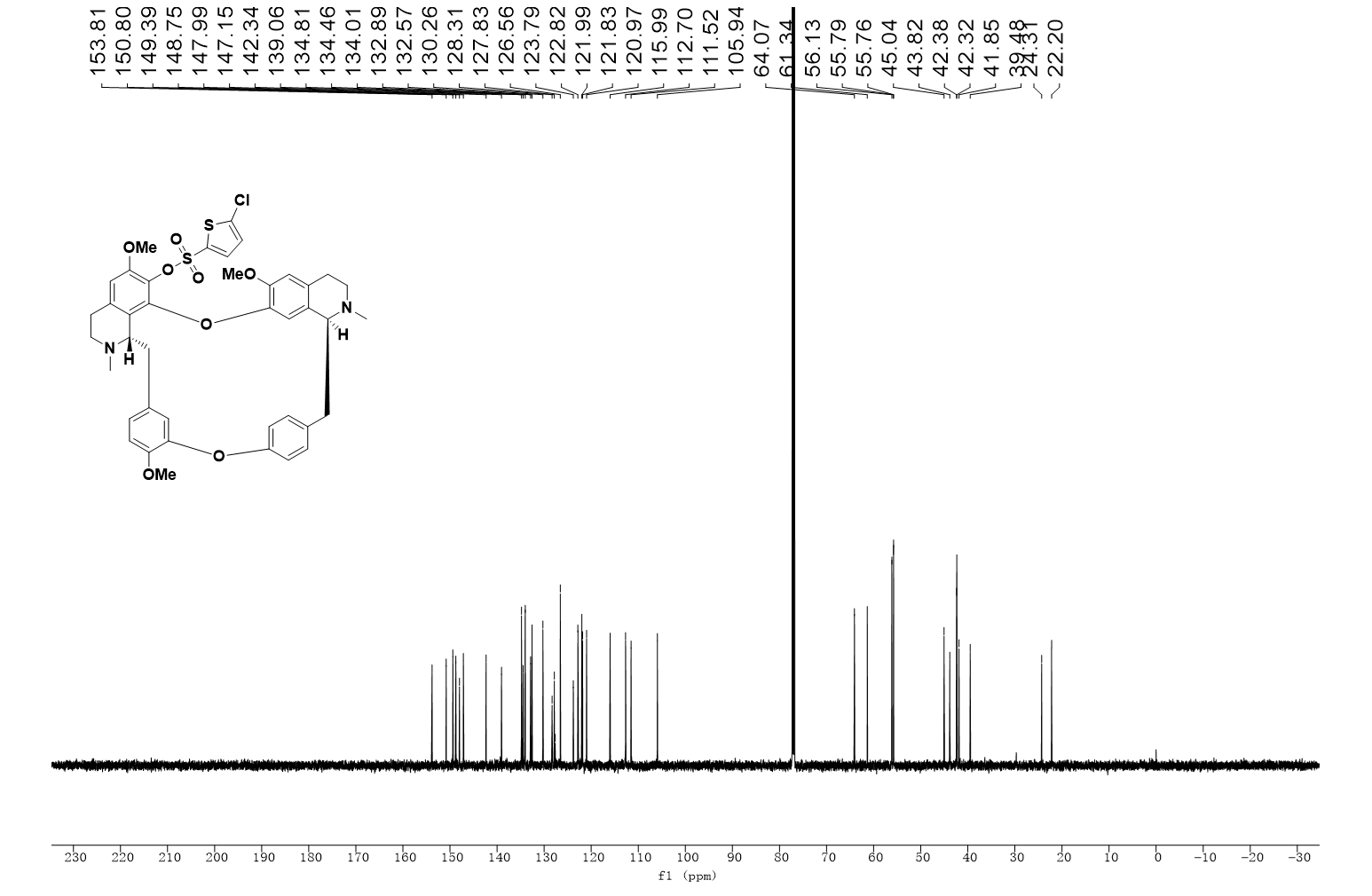


**Compound 29. 7-*O*-(5-Chlorothiophene-2-sulfonyl)-tetrandrine: HR-ESIMS spectrum**


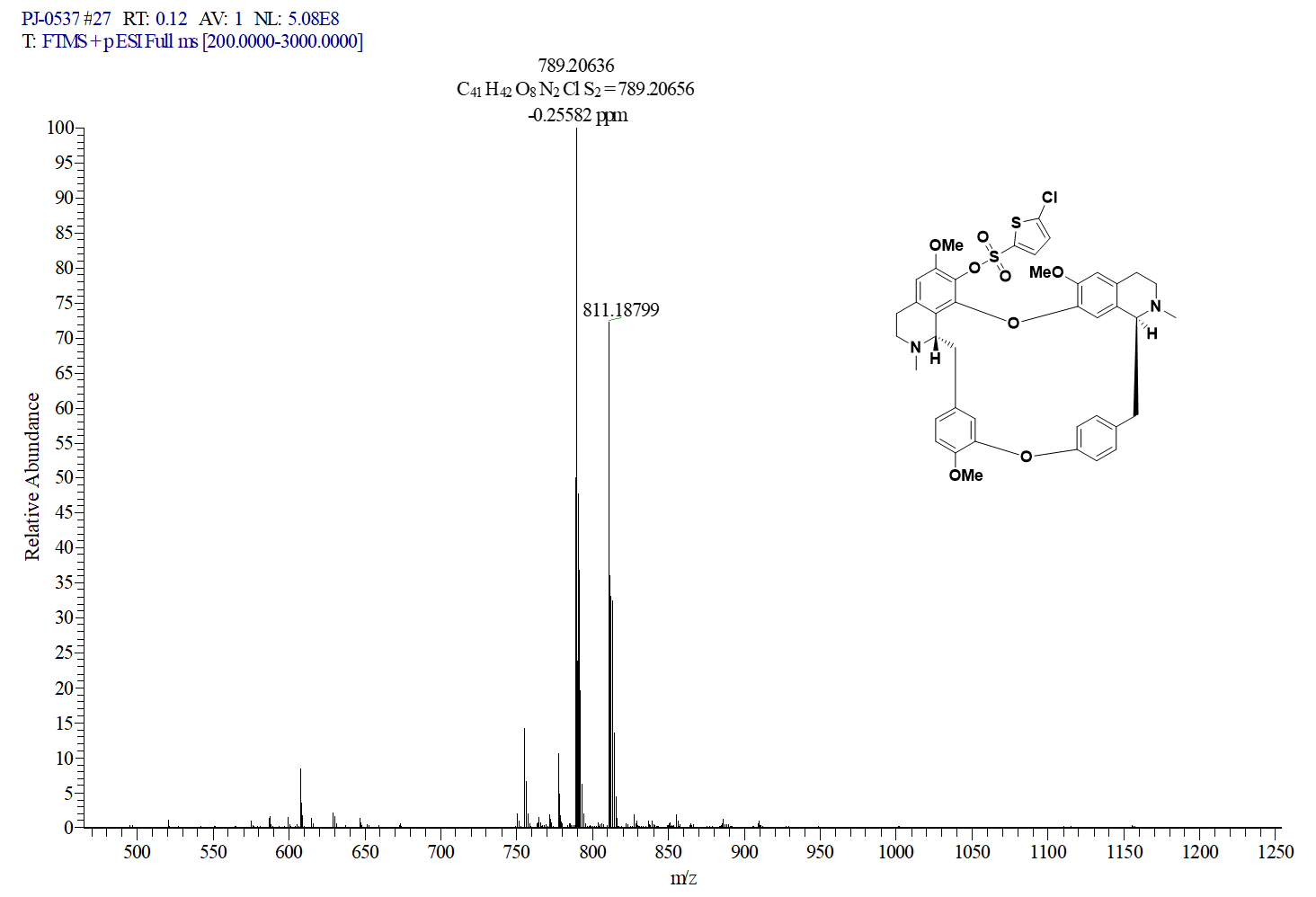


**Compound 30.** **7-*O*-(Biphenyl-4-sulfonyl)-tetrandrine: ^1^H-NMR spectrum**


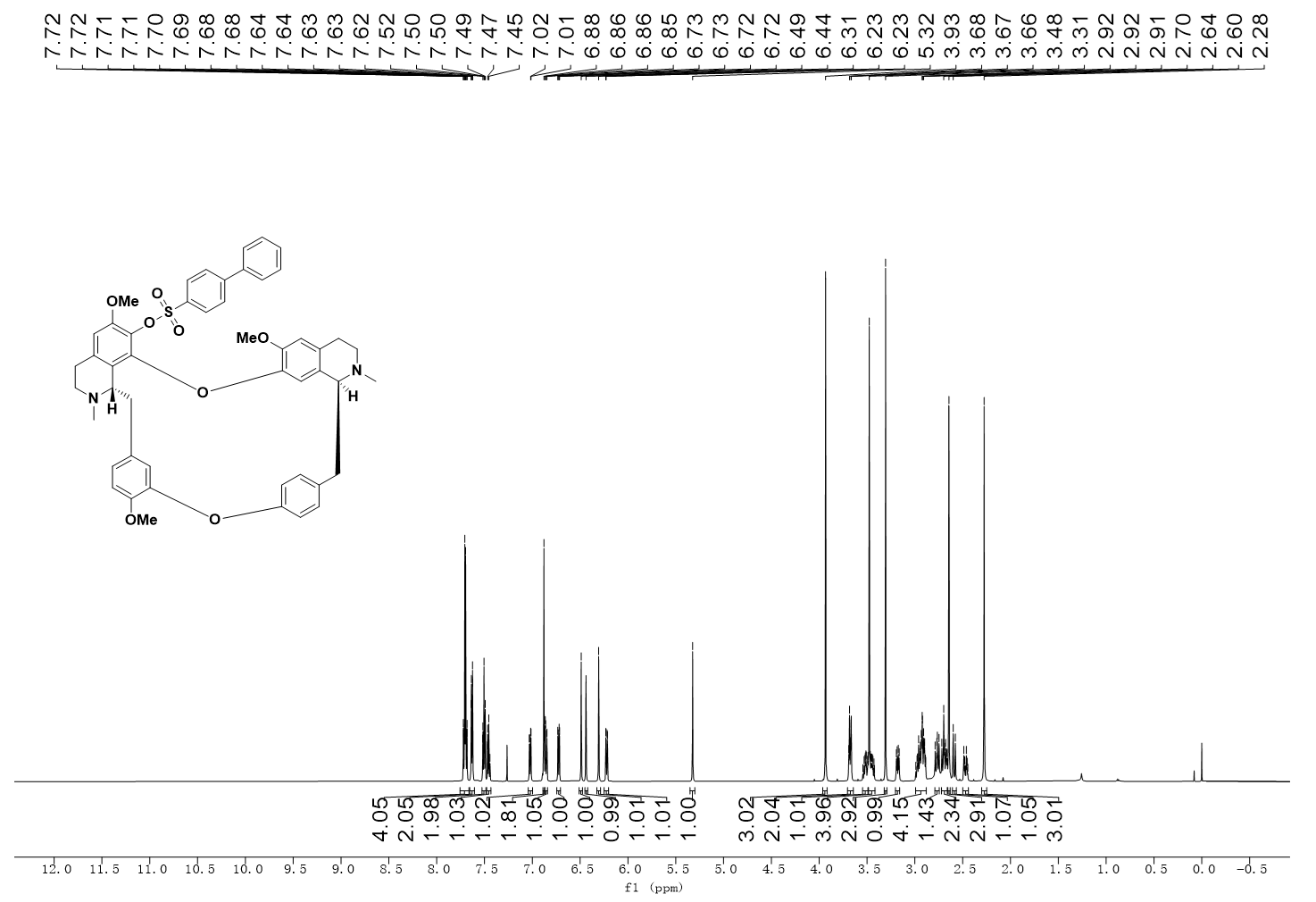


**Compound 30. 7-*O*-(Biphenyl-4-sulfonyl)-tetrandrine: ^13^C-NMR spectrum**


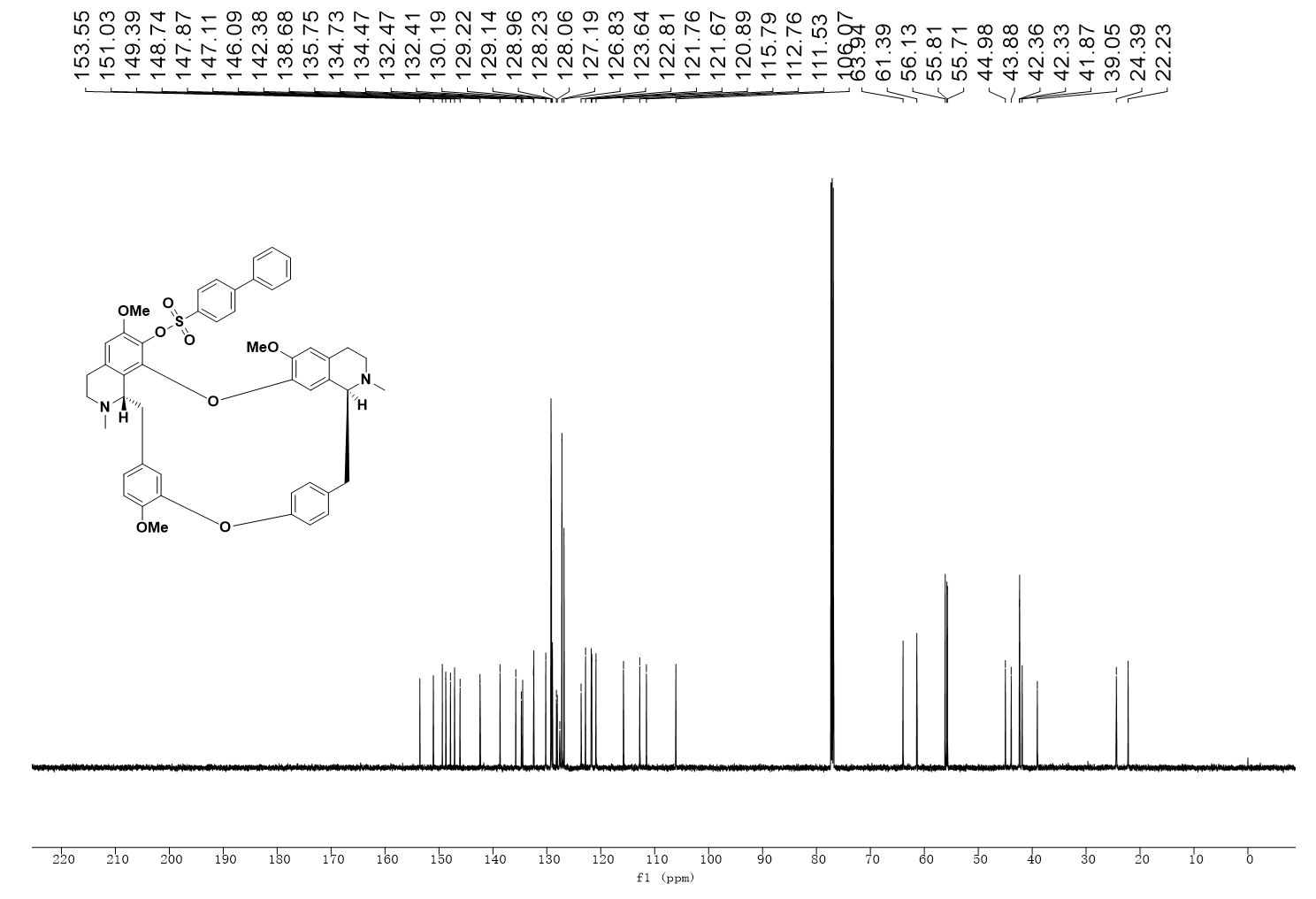


**Compound 30. 7-*O*-(Biphenyl-4-sulfonyl)-tetrandrine: HR-ESIMS spectrum**


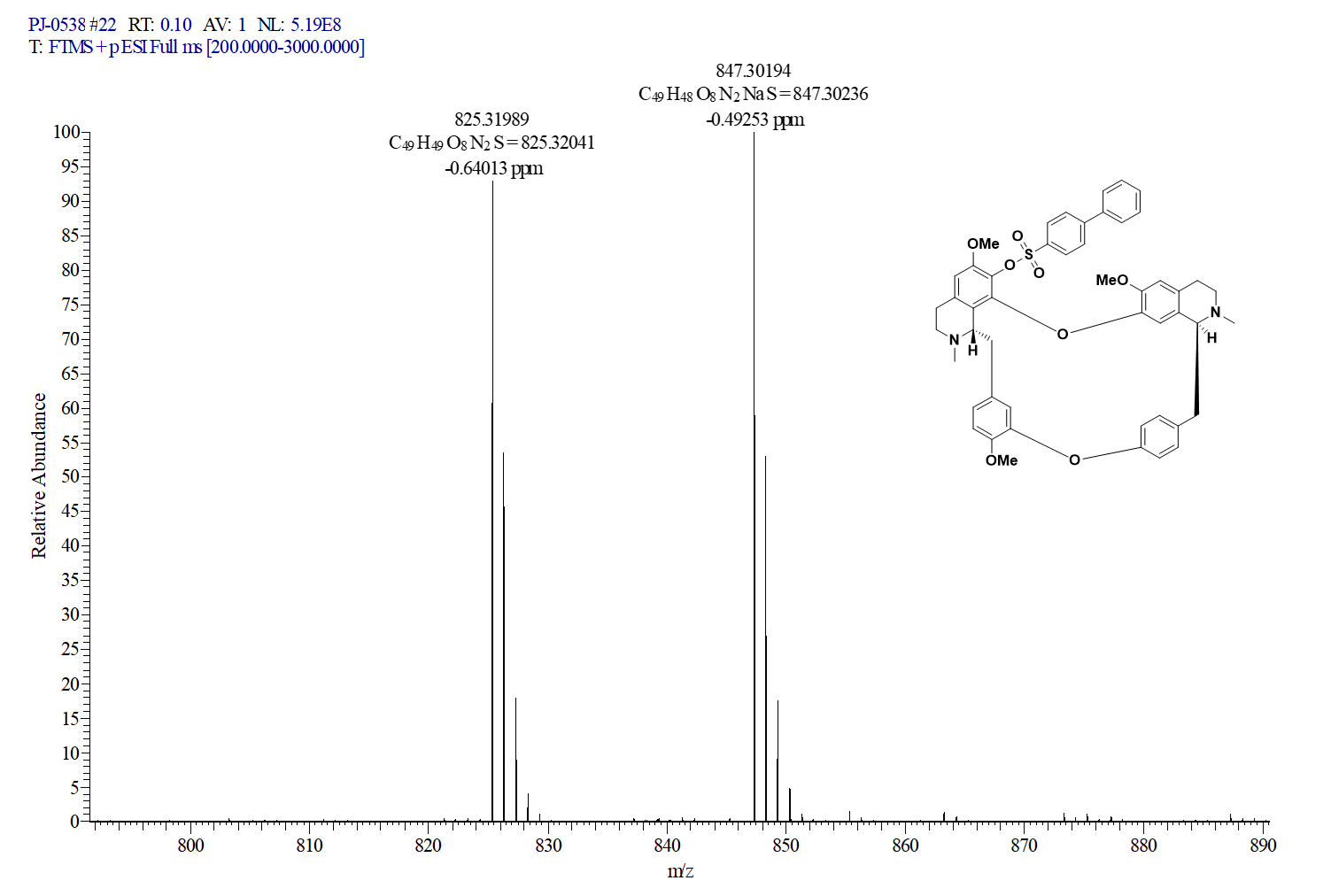


**Compound 31.** **7-*O*-(4-Cyanobenzenesulfonyl)-tetrandrine: ^1^H-NMR spectrum**


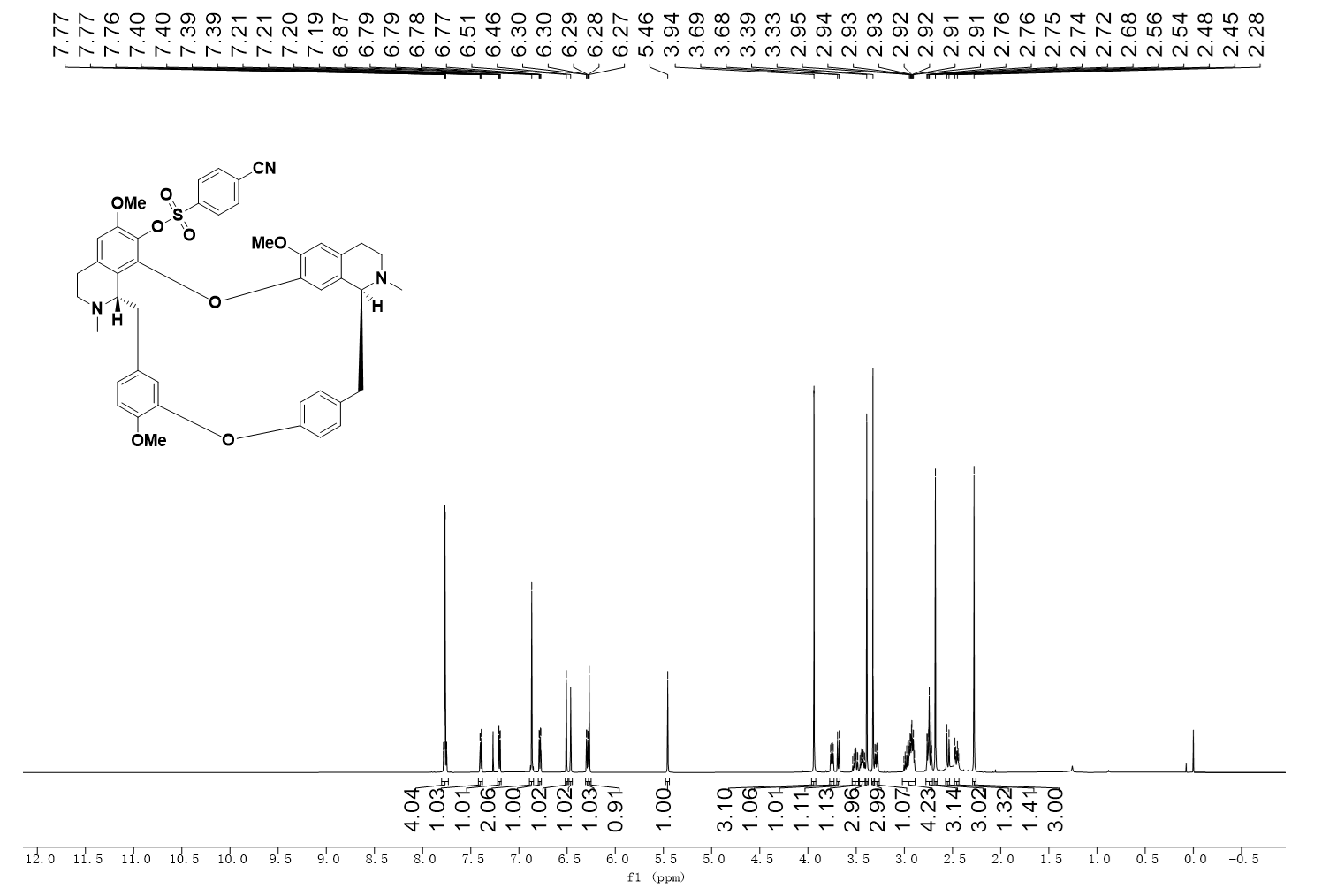


**Compound 31. 7-*O*-(4-Cyanobenzenesulfonyl)-tetrandrine: ^13^C-NMR spectrum**


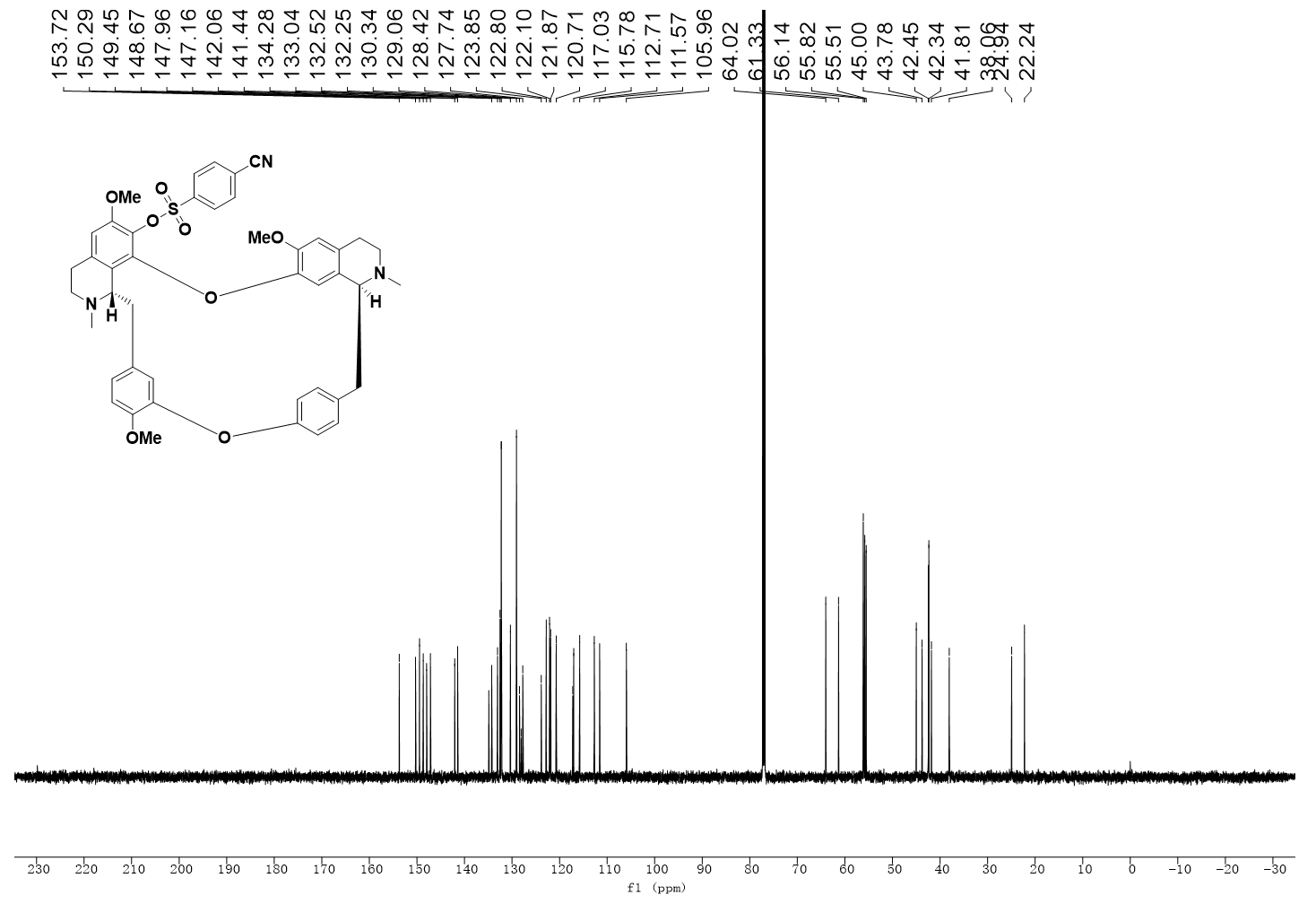


**Compound 31. 7-*O*-(4-Cyanobenzenesulfonyl)-tetrandrine: HR-ESIMS spectrum**


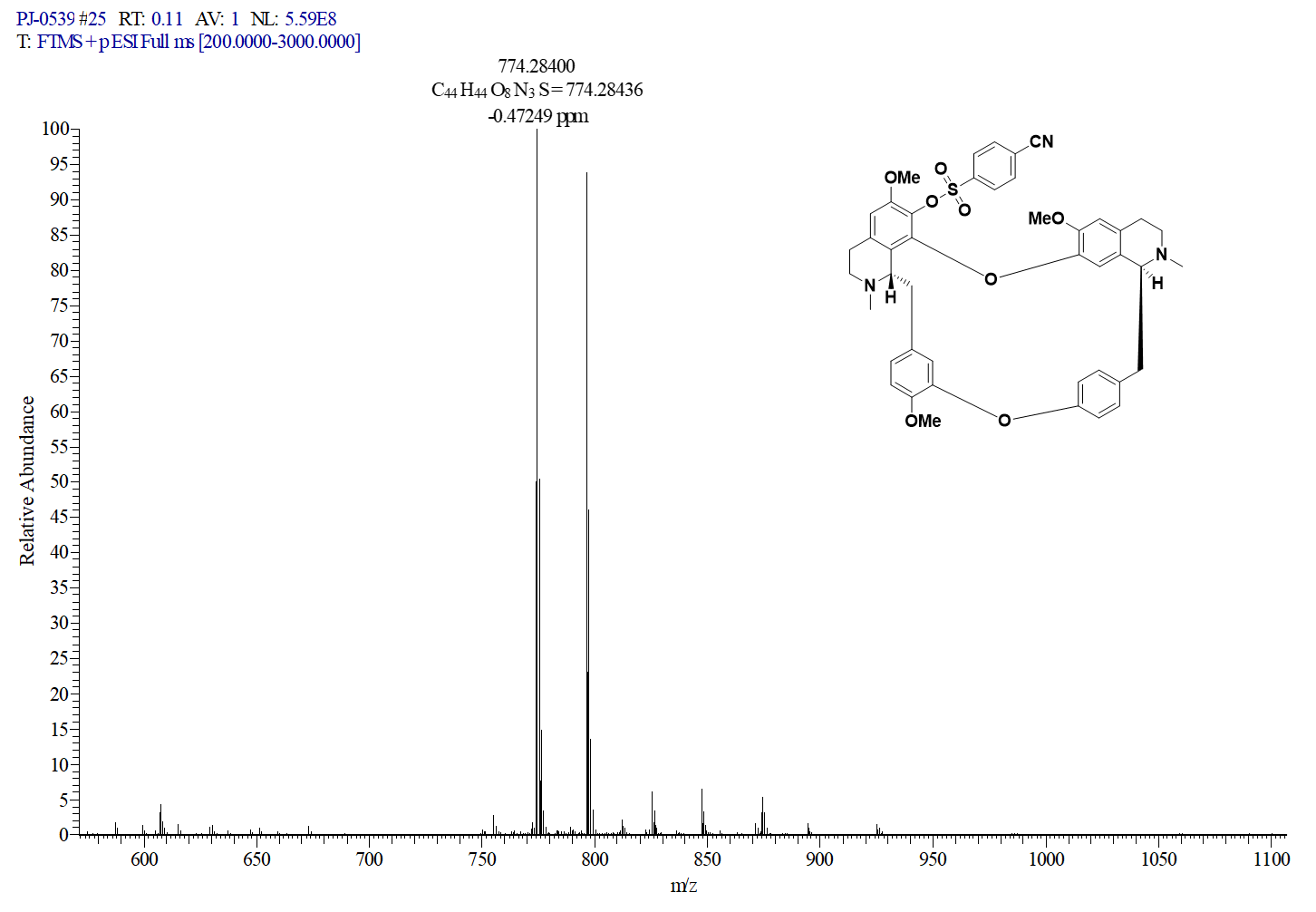


**Compound 32.** **7-*O*-(3-Cyanobenzenesulfonyl)-tetrandrine: ^1^H-NMR spectrum**

**Compound 32. 7-*O*-(3-Cyanobenzenesulfonyl)-tetrandrine: ^13^C-NMR spectrum**

**Compound 32. 7-*O*-(3-Cyanobenzenesulfonyl)-tetrandrine: HR-ESIMS spectrum**

**Compound 33.** **7-*O*-(Cyclopropanesulfonyl)-tetrandrine: ^1^H-NMR spectrum**

**Compound 33. 7-*O*-(Cyclopropanesulfonyl)-tetrandrine****: ^13^C-NMR spectrum**

**Compound 33. 7-*O*-(Cyclopropanesulfonyl)-tetrandrine: HR-ESIMS spectrum**

**Compound 34. 7-*O*-(2-Phthalimidoethanesulfonyl)-tetrandrine: ^1^H-NMR spectrum**

**Compound 34. 7-*O*-(2-Phthalimidoethanesulfonyl)-tetrandrine: ^13^C-NMR spectrum**

**Compound 34. 7-*O*-(2-Phthalimidoethanesulfonyl)-tetrandrine: HR-ESIMS spectrum**

**Compound 35. 7-*O*-(1-Dodecanesulfonyl)-tetrandrine: ^1^H-NMR spectrum**

**Compound 35. 7-*O*-(1-Dodecanesulfonyl)-tetrandrine: ^13^C-NMR spectrum**

**Compound 35. 7-*O*-(1-Dodecanesulfonyl)-tetrandrine: HR-ESIMS spectrum**

**Compound 36.** **7-*O*-(1-Propanesulfonyl)-tetrandrine: ^1^H-NMR spectrum**

**Compound 36. 7-*O*-(1-Propanesulfonyl)-tetrandrine: ^13^C-NMR spectrum**

**Compound 36. 7-*O*-(1-Propanesulfonyl)-tetrandrine: HR-ESIMS spectrum**

**Compound 37.** **7-*O*-(2-Naphthalenesulfonyl)-tetrandrine: ^1^H-NMR spectrum**

**Compound 37. 7-*O*-(2-Naphthalenesulfonyl)-tetrandrine: ^13^C-NMR spectrum**

**Compound 37. 7-*O*-(2-Naphthalenesulfonyl)-tetrandrine: HR-ESIMS spectrum**

**Compound 38.** **7-*O*-(Ethanesulfonyl)-tetrandrine: ^1^H-NMR spectrum**

**Compound 38. 7-*O*-(Ethanesulfonyl)-tetrandrine: ^13^C-NMR spectrum**

**Compound 38. 7-*O*-(Ethanesulfonyl)-tetrandrine: HR-ESIMS spectrum**
